# Feedback between megathrust earthquake cycle and plate convergence

**DOI:** 10.1038/s41598-023-45753-5

**Published:** 2023-10-30

**Authors:** Juan Martin de Blas, Giampiero Iaffaldano, Andrés Tassara, Daniel Melnick

**Affiliations:** 1 Department of Geosciences and Natural Resource Management, University of Copenhagen, Copenhagen, Denmark; 2 Present Address: Dipartimento di Scienze Chimiche, della Vita e della Sostenibilità Ambientale, Università di Parma, Parma, Italy; 3 Departamento de Ciencias de la Tierra, Universidad de Concepción, Concepción, Chile; 4 Instituto de Ciencias de la Tierra, Universidad Austral de Chile, Valdivia, Chile

**Keywords:** Geodynamics, Seismology, Tectonics

## Abstract

Over million years, convergence between the Nazca and South America tectonic plates results in Andean orogeny. Over decades/centuries, it fuels the earthquake cycle of the Andean megathrust. It is well recognised that, over the geologically-long term of million years, Andean orogeny feeds back onto plate convergence rates, generating temporal changes documented throughout the Neogene. In contrast, no feedback mechanism operated over the geologically-short term by the earthquake cycle is currently contemplated. In fact, it is commonly assumed that the rates of contemporary convergence, which are accurately measured via geodesy, remain steady during the megathrust earthquake cycle. Here we investigate whether the contemporary Nazca/South America plate motion varies over year-/decade-long periods in response to megathrust stress variations associated with the earthquake cycle. We focus on the decade preceding the three largest and most recent ${\varvec{M_w > 8}}$ earthquakes (2010 ${\varvec{M_w = 8.8}}$ Maule, 2014 ${\varvec{M_w = 8.1}}$ Iquique, 2015 ${\varvec{M_w = 8.3}}$ Illapel), and find slowdowns of both Nazca and South America whole-plate motions that exceed the impact of data uncertainty or noise. We show that the torque variations required upon Nazca and South America to generate the slowdowns are consistent with that arising from the buildup of interseismic stress preceding the earthquakes.

## Introduction

Since the advent of the plate tectonics theory^1–4^, the occurrence and recurrence of large earthquakes is studied in the context of relative plate motions and the consequent stress accumulation/release along the plate-boundary zones — a process referred to as earthquake cycle^5^. It is commonly assumed that the motions of tectonic plates^6–8^ remain steady during such cycles^5,9^, which comprise a phase of stress buildup spanning several years/decades (i.e. the interseismic phase), one of sudden release of stress during earthquakes (i.e. the coseismic phase), and a subsequent postseismic relaxation phase^9^. Under this premise, earthquake cycles are fueled by relative plate motions^10,11^, but stresses associated with such cycles are not viewed as potentially feeding back onto whole-plate dynamics and kinematics. Virtually all models of earthquake nucleation^12–15^ and seismic potential of tectonic margins^16–21^ rely on such assumption, particularly when estimating slip deficits. However, recent numerical simulations of plate dynamics^22^ suggest that stress variations during large-earthquakes cycles can in fact impact onto the dynamics of relatively small plates.

**Figure 1:**
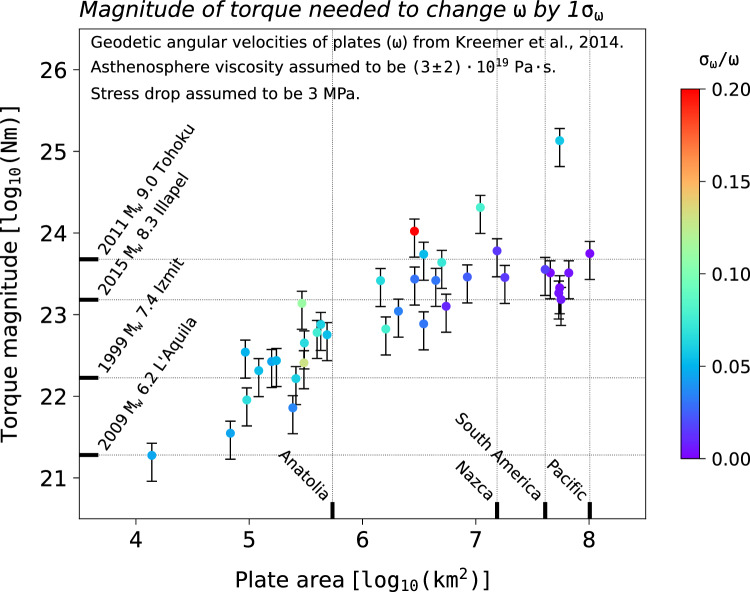
Comparison of torques (i) required to change contemporary plate motions and (ii) provided by the coseismic stress release of large earthquakes. Each dot corresponds to one tectonic plate whose Euler vector is available in the global tectonic model of Kreemer et al.^8^, and shows the magnitude of the torque required in order to change the plate angular velocity ($\omega$) — and thus the plate’s motion — by one standard deviation ($\sigma _{\omega }$). Each plate angular velocity features its own standard deviation, which is calculated from the Euler-vector covariances provided by Kreemer et al.^8^. Dots are coloured according to the ratio $\sigma _{\omega }/\omega$, which, to first-degree, is a proxy for the precision with which GNSS data constrain the motion of the plate at hand. At the lower end of the colour-scale are plates whose angular-velocity standard deviation is small relative to the angular velocity itself. Torque estimates are made assuming that the average viscosity of Earth’s asthenosphere is $(3 \pm 2) \times 10^{19}~\mathrm {Pa \cdot s}$ (uncertainty range is in thin black), and are plotted as a function of the plate area (horizontal axis). Black ticks on the horizontal axis indicate the areas of Anatolia, Nazca, South America and Pacific, for reference. Black ticks on the vertical axis report the magnitudes of the torque associated with the coseismic stress release of four recent, large earthquakes.

The global navigation satellite systems (GNSS) enable scientists to measure contemporary plate motions^23–25^ over few years/decades, and provide an observational basis to explore any links between plate motions and the earthquake cycle. Recent studies of the contemporary motions of the Anatolia^26^ and the Apulia^27^ microplates found that the torques required to generate their recent kinematic changes, evidenced by GNSS data, are consistent with those arising from stress changes during the interseismic and coseismic phases of two of the largest earthquakes ever occurred along their margins: the 1999 $M_W=7.5$ Izmit-Düzce earthquakes for Anatolia, and the 2019 $M_W=6.4$ Dürres earthquake for Apulia. These results beg the question of whether similar dynamics might concern medium-sized plates, thus generating kinematic changes sufficiently large to be detected at the GNSS precision. A first-order estimate of the torque magnitudes involved in the dynamics of medium- to large-sized tectonic plates suggests that it is not geodynamically implausible to pose the question above. Figure 1 shows the magnitude of torques required upon plates whose contemporary Euler vector (i.e. angular velocity $\omega$ and Euler pole) is available from GNSS observations^8^ in order to modify their angular velocity by one standard deviation ($\sigma _{\omega }$). Following previous studies^28^, we derive estimates of the torque variation required to change the motions of plates by differentiating the plate torque-balance equation at two moments in time, and taking into account the viscous resistance — exerted by the asthenosphere at the lithosphere base — that must be overcome in order to modify plate kinematics (see "Methods"). For reference, Fig. 1 y–axis illustrates the torque magnitudes generated by the coseismic stress drop — here assumed to be 3 MPa^29^ — of four large earthquakes of moment magnitude $M_W$ in range between 6 and 9 and whose coseismic slip fields are known from finite-fault solutions^30–33^ available from the SRCMOD public repository^34^ (see "Methods"). Correspondingly, the x–axis shows the area of four tectonic plates of different sizes. This figure suggests that, in principle, large earthquakes hold the potential to generate enough torque to alter the kinematics of medium-sized plates.

Here we focus on testing whether stress variations arising from the earthquake cycle(s) along the the Andean megathrust^35^ influence the dynamics of the Nazca (NZ) and South America (SA) plates. We use GNSS data to constrain temporal variations of the NZ and SA Euler vectors over a period of 10 years preceding three of the largest earthquakes that ruptured the Andean megathrust: the 2010 $\mathrm {M_W = 8.8}$ Maule earthquake^36–38^, the 2014 $\mathrm {M_W = 8.1}$ Iquique earthquake^39^ and the 2015 $\mathrm {M_W = 8.3}$ Illapel earthquake^40^. We investigate whether data evidence any temporal variations of NZ and SA motions in connection with the interseismic phase of the three megathrust earthquakes. Furthermore, we perform statistical tests to assess the robustness of any such inference against the number of observational constraints and the possible presence of data noise. Lastly, we explore whether the torque variations required upon NZ and SA in order to generate any inferred kinematic changes are consistent with those imparted to the plates by interseismic stress changes during the period covered by GNSS data.

## Contemporary whole-plate motions of Nazca and South America

The subduction of NZ underneath SA is responsible for the orogeny and uplift of the Andean mountain range^41^, as well as for the occurrence along the subduction zone of active volcanism and megathrust earthquakes^35,42^ — including some of the largest (i.e. $M_w > 8$) events ever recorded, like the $M_w\!\sim \!9.5$ 1960 Valdivia earthquake^43,44^ and, more recently, the destructive $M_w\!=\!8.8$ 2010 Maule earthquake^36–38^. The contemporary motion between NZ and SA can be measured via geodetic data, and particularly via GNSS position time series. Previous geodetic studies show that NZ converges towards SA in a rapid, oblique fashion at rates as high as 70 mm/yr^7,45–49^. There are, however, significant differences in the convergence rates reported by these studies, which resorted to geodetic measurements spanning variable and often non-overlapping periods of time. In line with the widespread assumption of plate-motion steadiness over the earthquake cycle, these differences have been ascribed to non-tectonic processes, including the impact of data noise. An alternative view might be that these studies sampled the temporally-varying NZ/SA motion over distinct periods of the Andean-megathrust earthquake cycle(s).

### Availability of GNSS data

**Figure 2:**
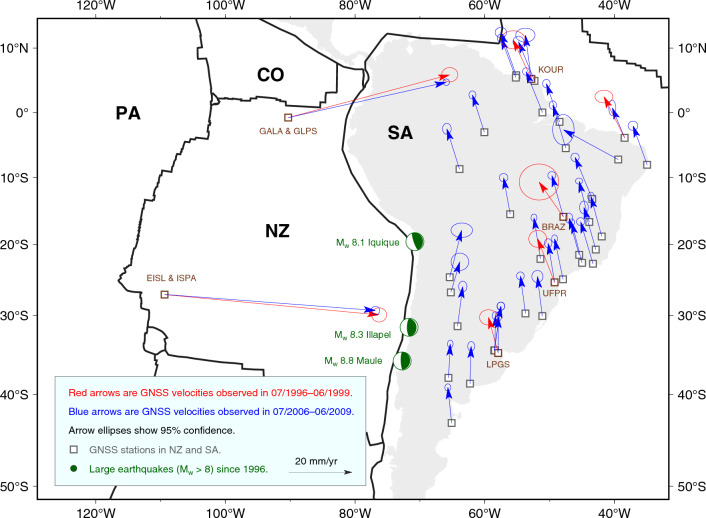
Velocities inferred at GNSS stations (empty squares) from position time series. Velocities are expressed relative to the IGS14 reference frame, and are used to infer the Euler vectors for the Nazca and South America plates during the periods from July 1996 to June 1999 (in red), and from July 2006 to June 2009 (in blue). Please note that not all velocities have been used to constrain the Euler vectors for the latter period in SA, since some sites have been excluded on the basis of their velocity residuals (see Supplementary Fig. 13 for details). For clarity, GNSS-site names for those stations addressed in Fig. 3 are shown in brown. Focal-mechanism solutions (USGS) of the three largest ($M_w>8$) megathrust earthquakes occurred along trench since 1996 (i.e. $M_w =8.8$ 2010 Maule, $M_w = 8.1$ 2014 Iquique, and $M_w = 8.3$ 2015 Illapel earthquakes) are shown as green beach balls. Continents are shown in light grey. Contours of tectonic plates^77^ are shown in solid black.* NZ* Nazca,* SA* South America,* PA* Pacific,* CO* Cocos. This figure has been produced using Generic Mapping Tools version 6^76^.

We utilise publicly-available GNSS data from the Nevada Geodetic Laboratory (NGL) repository^50^ (http://geodesy.unr.edu/) to obtain GNSS station velocities, and analyse the kinematics of NZ and SA prior to the 2010 $\mathrm {M_w = 8.8}$ Maule, the 2014 $\mathrm {M_w = 8.1}$ Iquique, and the 2015 $\mathrm {M_w = 8.3}$ Illapel earthquakes, which ruptured different segments of the Andean megathrust (Fig. 2). NZ hosts only a limited number of islands where geodetic instrumentation may be deployed. Consequently, its spatial coverage of GNSS stations is sparse compared to other settings. Nonetheless, two pairs of continuous GNSS stations, located in Easter Island (EISL, later replaced by ISPA) and the Galapagos Islands (GALA, replaced subsequently by GLPS) (Fig. 2), constrain NZ kinematics prior to 2010. Stations EISL and ISPA are close enough (<5 km) that their data are commonly considered as if collected at a single site. The same holds for GALA and GLPS. These stations have been consistently paired in all previous studies of global plate kinematics^7,8,47^, and represent the entirety of continuously-recording stations whose data are publicly available. Several GNSS stations are deployed within SA, although many of them are located along the subduction zone, making them subject to the impact of plate-boundary deformation^18,38^), and thus may exhibit velocity components that depart from the whole-SA plate motion. Therefore, to determine the motion of the stable portion of SA, we elect to only utilise GNSS stations located more than 700 km away from the Andean margin and other plate boundaries (Fig. 2).

We use GNSS data during two 3-year-long time periods that cover a fraction of interseismic phase leading to the 2010 Maule, 2014 Iquique, and 2015 Illapel earthquakes. The first (i.e. earlier) period extends from July 1996 to June 1999, while the second (i.e. later) one from July 2006 to June 2009. The choice of start/end times is based on the following criteria: (i) periods are longer than 2.5 years — commonly considered the minimum standard to obtain tectonically–meaningful station velocities^51^. (ii) The first period begins at the earliest time when two stations are available in NZ. (iii) The second period ends approximately half a year prior to the occurrence of the 2010 Maule earthquake. (iv) Since our analyses will be based on temporal differences of plate motions, start and end months are the same for earlier and later periods in order to minimise the impact of seasonal signals^52^.

**Figure 3:**
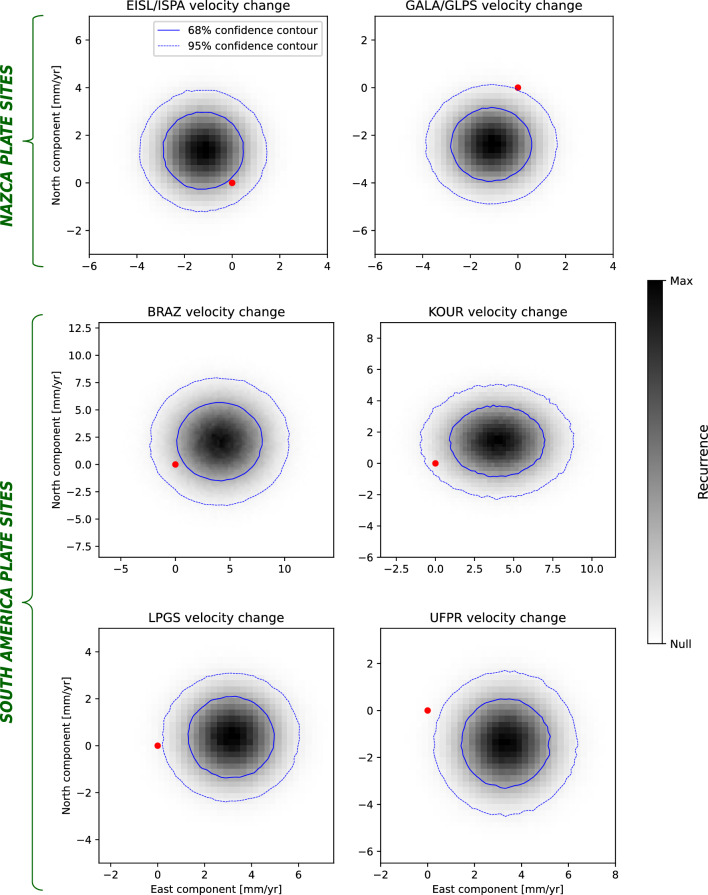
Distributions of temporal change of velocity observed at GNSS stations whose data cover both earlier (1996–1999) and later (2006–2009) periods. Velocities observed at stations EISL and ISPA, and at GALA and GLPS over the two periods are considered as observed at the same site due to proximity of stations ($<5$ km apart, see main text). Distributions are mapped as 2D histograms of the difference between ensembles of station velocities over the earlier and later periods. The latter ones are obtained from the observed mean and standard deviation of the station GNSS velocity. Blue solid/dashed lines show the regions where the most-recurrent 68/95% of the velocity-difference samples fall. Red dots show (0, 0), which would be the centre of the distribution in case of no temporal change of station velocity.

We trim GNSS position time series, and use the MIDAS software^53^ to obtain the station velocities and associated uncertainties (Supp. Files 1–8) relative to the IGS14 reference frame — i.e. the GNSS-only realisation of the International Terrestrial Reference Frame 2014^54,55^. MIDAS implements a Theil-Sen estimator^53^ on position pairs that are 1 year apart from each other, and that do not cross the times of steps in the observed position series arising from technical (e.g. antenna changes) or tectonic (e.g. earthquakes nearby the GNSS station) events. The times of these steps are available from the NGL repository, and are explicitly accounted for in our analyses as optional input to MIDAS. The computational approach implemented in MIDAS has been designed and tested in order to handle the impact of offsets as well as annual/semiannual temporal variations onto the inference of station velocities. In order to corroborate the so-obtained station-velocity inference, we compare station velocities output by MIDAS with those obtained from standard linear trajectory models^56^ (in the following referred to as TMs) of the observed position time series. Specifically, we calculate and compare TMs in two cases: in one, we follow the classical approach of Bevis & Brown^56^ to fitting TMs to observations, and invert for the amplitudes of reference position (offset), linear term (nP = 1), annual and semiannual cycles (nF = 2), as well as steps (Heaviside functions) occurring at the epochs reported in the NGL repository for the specific GNSS station at hand. In a second calculation, we repeat the TM inversion scheme, this time enforcing the amplitude of the linear term to be equal to the station-velocity output by MIDAS, while maintaining the amplitudes of offset, steps and annual/semiannual terms as free parameters of the model. For each GNSS station considered here, TMs are reported and compared in the Supplementary information (Supplementary Figs. 18–100). Across all stations and time intervals considered, we find the following: (i) station velocities obtained in the two cases are systematically similar, with differences being in most cases less than half of the standard deviation associated with the MIDAS velocity. (ii) The TMs obtained in the two cases fit the observed position time series equally well: in fact, the distributions of position residuals (difference between observed position and TM) are virtually the same in the two cases (insets in Supplementary Figs. 18–100). On the basis of these analyses, we take the velocities output by MIDAS as tectonically-meaningful representations of the GNSS-station velocity. For most of the stations whose data cover both earlier and later periods, we observe statistically-significant differences in the inferred velocities across the two periods (Fig. 2), evident also from the distributions of temporal velocity-difference at GNSS stations whose data is available in both earlier and later periods (Fig. 3). This lends support to our working hypothesis.

In order to corroborate the inference of temporal change of station velocities against the possible impact of longer-than-seasonal (i.e. $>1$ year) signals unrelated to the whole-plate motion^53^, we also use position time series for earlier and later periods that are 1 year longer — that is, from July 1996 to June 2000, and from July 2005 to June 2009 (see Supplementary Information). This allows us to test the consistency of the inferences we derive against the potential presence of such signals, which would necessarily make site velocities calculated over variably–long periods to be significantly different. In this case too, we find evidence of temporal change of station velocities. Under the plate–motion approximation^57^, also referred to as Euler-vector approximation, station velocities should be regarded as samples of the the whole-plate motion on the globe, which is described via the use of Euler vectors^58^.

### Inference of Nazca and South America Euler vectors

**Figure 4:**
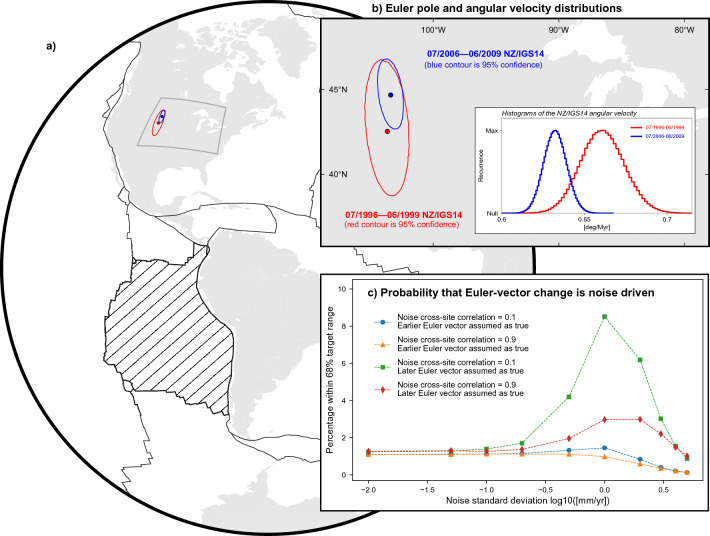
Euler vectors for the motion of NZ relative to IGS14 for the periods July 1996 to June 1999 (in red) and July 2006 to June 2009 (in blue). (**a**) Distribution of Euler-pole ensembles. Contours show 95% confidence region. Continents are in light grey, plate margins^77^ are in solid black. NZ plate is hatched. (**b**) Close-up view of the Euler-pole distributions. Inset shows the distributions of the NZ/IGS14 angular velocities associated with the Euler poles. (**c**) Estimate of the probability that random, cross-site correlated noise in GNSS station velocities (cross-correlation coefficient is taken to be 0.1 and 0.9) is the sole factor determining the difference between Euler vectors inferred for the earlier and later periods (see main text for details). This figure has been produced partially using Generic Mapping Tools version 6^76^.

**Figure 5:**
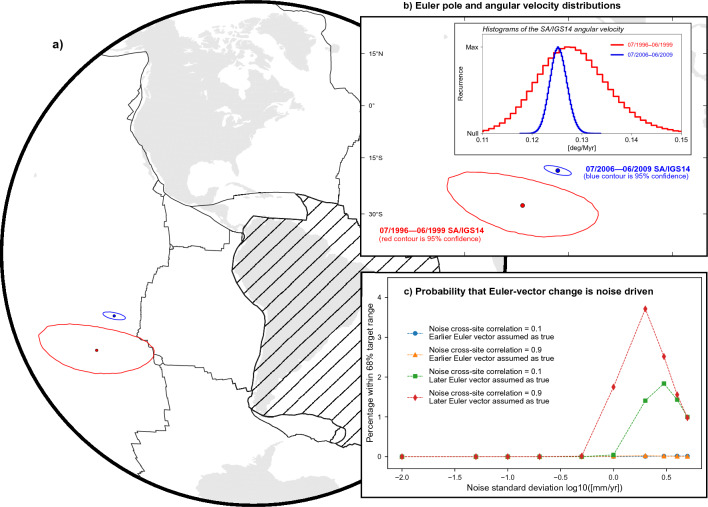
Same as Fig. 4, but for the Euler vector describing the motion of SA relative to IGS14 for the periods July 1996 to June 1999 and July 2006 to June 2009. This figure has been produced partially using Generic Mapping Tools version 6^76^.

We obtain Euler vectors defining the whole-plate motion of NZ and SA relative to IGS14 (indicated as $\vec {\omega }_{NZ/IGS14}$ and $\vec {\omega }_{SA/IGS14}$) over the earlier and later periods via minimisation of the sum of squared velocity misfits^59^. We generate Gaussian distributions comprising $10^6$ samples of GNSS station velocities by using their mean values and standard deviations, and employ these to obtain ensembles of $10^6$ realisations of the Euler vectors. From these, we calculate equally-large ensembles of station velocity-residuals — i.e. the difference between observed station velocity and velocity predicted by the inferred Euler vector at the station position. We use these to assess whether any stations exhibit average residuals larger than their uncertainties at the 95% confidence level, thus suggesting the presence of possibly large velocity components unrelated to the whole-plate motion that warrants exclusion from the Euler-vector calculation. For SA, this results in the exclusion of a few GNSS stations for the 3-year-long and 4-year-long later periods in order to obtain acceptable residuals at all sites used for calculating the Euler vectors (see Supp. Figs. 13 and 14 and Supp. Table 2). We use the ensembles of $\vec {\omega }_{NZ/IGS14}$ and $\vec {\omega }_{SA/IGS14}$ to obtain $10^6$ realisations of the Euler vector describing the motion of NZ relative to fixed SA — i.e. $\vec {\omega }_{NZ/SA} = \vec {\omega }_{NZ/IGS14} - \vec {\omega }_{SA/IGS14}$. Lastly, we calculate average values and associated covariances of Euler-vector ensembles (see Supp. Tables 1–3). The comparison of the distributions of Euler poles and angular velocities over the 3-year-long earlier and later periods evidences a change in both $\vec {\omega }_{NZ/IGS14}$ and $\vec {\omega }_{SA/IGS14}$ (Figs. 4 and 5). The kinematic change experienced by NZ is characterised by a $\sim \! 10\%$ decrease in the angular velocity as well as a shift in the Euler pole location. In fact, mean values of Euler pole and angular velocity for the later period fall outside the 95% confidence region/interval associated with the earlier period. The change experienced by SA consists of a shift in the Euler pole ($\sim 1600~$ km to the northeast), while the angular velocity remains steady at the 68% confidence level (see Fig. 5). Similar inferences hold for Euler vectors calculated using 4-year-long periods — the only exception being that the Euler-pole shift experienced by SA is less pronounced, yet the mean Euler poles for the earlier period falls outside each the 95% confidence region of the one for the later period (see Supplementary Information).

### Robustness of the inference of Euler-vector changes

We perform a test for the impact of random noise in GNSS data, as well as the widely-used F-ratio test^60^: in the former type of test, which we reprise from previous studies^26,27^, we start from the premise that — contrary to the working hypothesis — the Euler vector has not changed through time, and assess the probability that the change inferred from the analyses above is in fact not of geodynamic origin, but owes exclusively to data noise. We regard as *noise* any velocity component measured at GNSS stations that is not related to the whole-plate motion, regardless of whether it has a tectonic origin or not. For example, it has been argued that station GALA could be affected by volcanic deformation^49^, resulting in a velocity deviation of $\sim 2$ mm/yr during the earlier time period. Notably, such a departure would be taken into account in the subsequent noise tests. We start by assuming that one of the Euler vectors — variably, either the one for the earlier or the later periods — represents a faithful realisation of the *true Euler vector*, while the other — which we refer to as *target Euler vector* — differs from the true one only because of the addition of random noise to station observed velocities. We use the true Euler vector to calculate the surface velocities at the locations of stations used to constrain the target Euler vector. We then add a distribution of $10^6$ cross-site correlated Gaussian random-noise samples to these velocities. We use cross-site correlation coefficients of 0.1 and 0.9^61^, while the standard deviation of the noise distributions remains a free parameter ranging from $10^{-2}$ to 5 mm/yr. Next, we use the noisy station velocities to calculate $10^6$ realisations of noisy Euler vectors, and assess what fraction of them falls within the 68% confidence level of the target Euler-vector distribution. Given the size of the ensembles, we interpret this as an estimate of the probability that random noise solely accounts for the inferred difference between the earlier and later Euler vectors. In the case of NZ, these tests indicate that there is a probability of $\sim 8\%$ at most that the inferred Euler-vector change is only apparent and due to data noise (Fig. 4c). In the case of SA, such probability decreases to $\sim 3.5\%$ (Fig. 5c)

In addition to the noise tests, we perform the F-ratio test, which evaluates the statistical significance of a decrease in the chi-squared value ($\chi ^2$) obtained when increasing the number of parameters of a model that is used to fit a set of observations^59,62,63^. In this case, we evaluate the probability that a single Euler vector (3 independent parameters) obtained from all GNSS station–velocities across periods would fit GNSS observations equally well or better than two distinct Euler vectors (6 independent parameters), one for each period. The F-ratio is defined as^60^ $F=[(\chi ^2_{a}-\chi ^2_{1}-\chi ^2_{2})/3]/[(\chi ^2_{1}+\chi ^2_{2})/(N-6)]$, where $\chi ^2_{a}$ is the chi-squared value obtained when using all GNSS station velocities, $\chi ^2_{1}$ is the one obtained when using velocities observed during the earlier period, while $\chi ^2_{2}$ is the one obtained when using velocities observed during the later period. Results from these tests performed on $\vec {\omega }_{NZ/IGS14}$ and $\vec {\omega }_{SA/IGS14}$ are reported in the Supplementary Information (Supp. Table 4). For the case of $\vec {\omega }_{NZ/IGS14}$, the F-ratio test yields high probabilities, which we tend to interpret as a direct reflection of the limited number of observational constraints (2 GNSS stations). In contrast, the F-ratio test for $\vec {\omega }_{SA/IGS14}$, which relies on more observations, yields a very low probability of $2 \cdot 10^{-4}~\%$.

### Temporal changes of NZ whole-plate motion relative to SA

**Figure 6:**
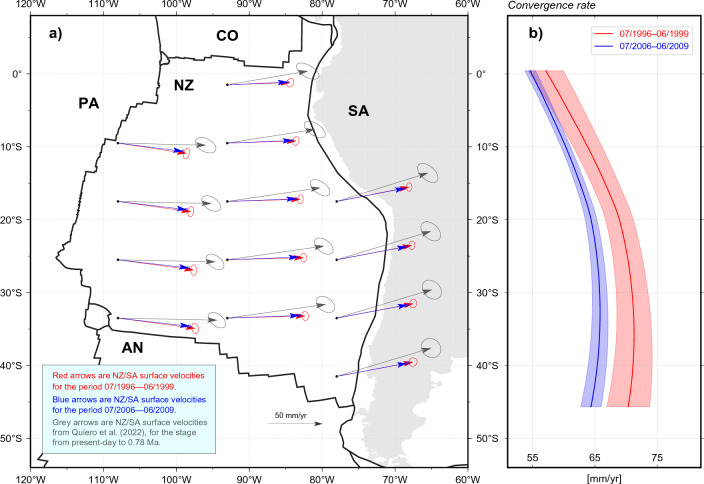
(**a**) Theoretical surface velocities illustrating the change in the NZ plate kinematics relative to SA from the period July 1996—June 1999 (red arrows) to the period July 2006—June 2009 (blue arrows). Surface velocities calculated from the Euler vector inferred by Quiero et al.^66^ from the stage from present-day to 0.78 Ma are shown in grey. Ellipses around velocity arrows show the 95% confidence level. Continents are in light grey, and plate boundaries are shown with thick black lines. (**b**) Convergence rate calculated along the NZ-SA plate boundary (i.e. the Andean Trench), expressed in mm/yr and calculated using the Euler vectors defining the motion of NZ relative to SA for the first (in red) and last (in blue) time periods. Solid lines represent the average values, while the shaded areas indicate the $2\sigma$ (95%) confidence range. This figure has been produced partially using Generic Mapping Tools version 6^76^.

We sample surface velocities at uniformly-distributed locations within NZ from the ensembles of $\vec {\omega }_{NZ/SA}$ for the earlier and later periods. This permits visualising the temporal change of the NZ whole-plate motion relative to SA during the decade preceding the Maule, Iquique and Illapel earthquakes. The plate tectonic approximation^57^ is known to be less accurate in describing surface velocities at locations within tectonic plate-boundary zones, as these experience a degree of deformation that might result in local velocities being slightly different than those implied by Euler vectors^64,65^. While we remain aware of this, we note that any such departure would affect equally the contemporary Euler vectors for the earlier and later periods. On this basis, it is still appropriate to calculate NZ/SA velocities implied by Euler vectors also at locations along the Andean megathrust, and focus on their temporal differences, rather than on the values associated with each Euler vector separately. Whether inside NZ (Fig. 6a) or along the Andean margins (Fig. 6b), we estimate the temporal decrease of NZ/SA convergence rate in the decade preceding 2010 to be as high as 6 mm/yr (or 8% of the earlier velocity) at the 95% confidence level. Even larger differences exist between the contemporary velocities obtained from geodetic observations and past velocities reconstructed over Myr-long stages from pickings of the observed ocean-floor magnetisation: we compare the contemporary NZ whole–plate motions inferred here to those implied by the NZ/SA Euler vector for the stage from present-day to 0.78 Ma, reconstructed in the recent study of Quiero et al.^66^, who used the most-resolved finite-rotation sets available in the literature for the plate circuit connecting NZ to SA via the Nubia, Antarctica and Pacific plates (Fig. 6a). In this case, differences of NZ whole-plate motion are as high as 25 mm/yr (or 38% of the contemporary motion), and are discussed below.

## Torque variations upon Nazca and South America

We test whether the temporal changes of NZ and SA whole-plate motions observed from the earlier to the later periods using independent networks of GNSS stations result from the interseismic stress buildup preceding the Maule, Iquique, and Illapel earthquakes. To this end, we generate and compare parameterised estimates of two types of torque variation: one is the torque variation required to alter the kinematics of NZ and SA to the degree evidenced by their Euler-vector changes. The other type is the torque variation generated upon NZ and SA by the buildup of interseismic stress along the future rupture area during the interval covered by GNSS data, which represents a fraction of the interseismic phase(s) preceding the three earthquakes.

Previous studies^26,28^ parameterised the torque variation required to change Euler vectors by differentiating the generic plate torque-balance equation at two moments in time. This effectively translates, in quantitative terms, the notion that in order to alter the motion of a tectonic plate, a torque variation must overcome the viscous resistance exerted at the plate/asthenosphere interface by the Couette component of the asthenospheric flow^28^. This approach yields a linear relationship linking the vector space of torque variations acting upon a plate to that of the Euler-vector changes experienced by the plate, through an operator that depends on the shape of the plate as well as on the viscosity of the asthenosphere underneath it (see "Methods"). We utilise such a relationship to estimate torque variations needed upon NZ and SA to generate the contemporary changes of their Euler vectors. Specifically, we use the ensembles of Euler vectors for earlier and later periods to generate an ensemble of torque-variation vectors under the assumption that the average viscosity of the asthenosphere is in range from $1\times 10^{19}$ to $3\times 10^{19}$ $\mathrm {Pa \cdot s}$.

Following previous studies^26,27^, the torque variation imparted to a plate by the interseismic stress buildup may be estimated as the opposite of the torque variation associated with the coseismic stress drop of the same earthquake. This signifies the notion that, during the interseismic and coseismic phases of an earthquake, stresses are directed opposite to each other. The coseismic torque variation, in turn, can be parameterised from models of the finite–fault slip during the 2010 Maule, the 2014 Iquique, and the 2015 Illapel earthquakes^32,67,68^, which provide vector fields for the direction of stress drop over the associated rupture areas (see "Methods"). In using models of finite–fault slip, we assume a linear relationship between coseismic slip and stress drop over the rupture area, and cast the maximum stress–drop value (associated with the largest coseismic slip) as a free parameter in range from 1 to 7 MPa — in line with global statistics of large earthquakes^29^. This serves the purpose of assessing the impact that uncertainty about the actual stress-drop value has onto the torque–variation estimate. The uncertainties of other finite-fault model parameters — such as strike, dip, and rake field — are also taken into account (see "Methods"). We visualise distributions of torque-variation ensembles by focusing separately on the geographical location where the torque-variation vector intersects the Earth’s surface (i.e. the torque-variation pole), and of the magnitude of the torque-variation vector (i.e. the vector norm). Since the Maule, Iquique, and Illapel earthquakes occurred at the interface between NZ and SA, the $3{rd}$ Newton’s law of motion implies that the force — and thus the torque — imparted to one plate during the interseismic/coseismic phase of any of these earthquakes is equal and opposite to that imparted to the other plate during the same phase. Hence, the poles of the torque-variation imparted to NZ and SA are antipodal.

A precise estimate of what portion of the total interseismic torque variation is imparted to a plate during a fraction of the interseismic phase may be complicated by the fact that the length of the earthquake cycle, as well as how the interseismic stress builds up through time are generally unknown. While models of the idealised earthquake cycle often assume that the interseismic stress grows linearly through time^5^, observational evidence^11,42,69^ suggests that this is not a realistic model assumption. To mitigate the impact of such uncertainty, we resort to historical catalogues for the occurrence of past earthquakes that ruptured the same sections of the Andean megathrust as those ruptured by the 2010 Maule, the 2014 Iquique, and the 2015 Illapel earthquakes^35,70^. These earthquakes occurred, respectively, 175, 133, and 67 years before them. We take those are representative values of the earthquake recurrence times, and elect to calculate the magnitude of the torque variations imparted to NZ and SA in two end-member cases that represent opposite extremes of a spectrum of possibilities: in one case we assume that the whole interseismic stress buildup occurs over the same interval covered by GNSS observations — i.e. from 1996 to 2009. In the other case, we take a pro-rata of the whole interseismic stress buildup equal to the ratio between the interval covered by GNSS data and the assumed recurrence times indicated above. These estimates represent lower and upper limits to the actual stress buildup between 1996 and 2009, and thus arguably encompass the true, unknown scenario.

**Figure 7:**
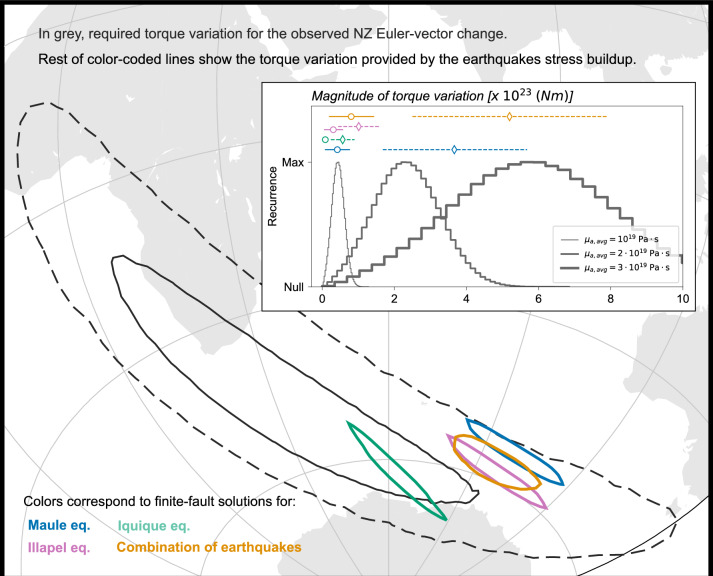
Comparison of the distributions of ensembles of torque variation (i) required upon NZ to explain the GNSS–observed Euler–vector change (in dark grey) and (ii) imparted to NZ by the interseismic buildup of stress preceding the 2010 Maule, 2014 Iquique, and 2015 Illapel earthquakes (in colours, including the torque variation resulting from their superimposition). The main map shows the distributions of torque-variation poles (contours show the regions where the most-recurrent 95% of poles fall, continents are in light grey), while the inset displays the distributions/ranges of torque-variation magnitude. In the inset, dashed-coloured lines show the range of torque-variation magnitudes obtained when considering the entire interseismic stress buildup phase. Instead, solid-coloured lines show the fraction of that would be built up during the period covered by GNSS observation if the interseismic stress grew linearly through time — calculated by multiplying the total interseismic torque-variation by the ratio between the 10–yr and the recurrence time of each considered earthquake (see main text for details). Grey histograms show the distributions of torque-variation magnitudes required to explain the NZ kinematic change, and are calculated using an average asthenosphere viscosity of $10^{19}$ $\mathrm {Pa \cdot s}$ (thin grey line), $2 \times 10^{19}$ $\mathrm {Pa \cdot s}$ (medium-thick grey line) or $3 \times 10^{19}$ $\mathrm {Pa \cdot s}$ (thick grey line). This figure has been produced partially using Generic Mapping Tools version 6^76^.

**Figure 8:**
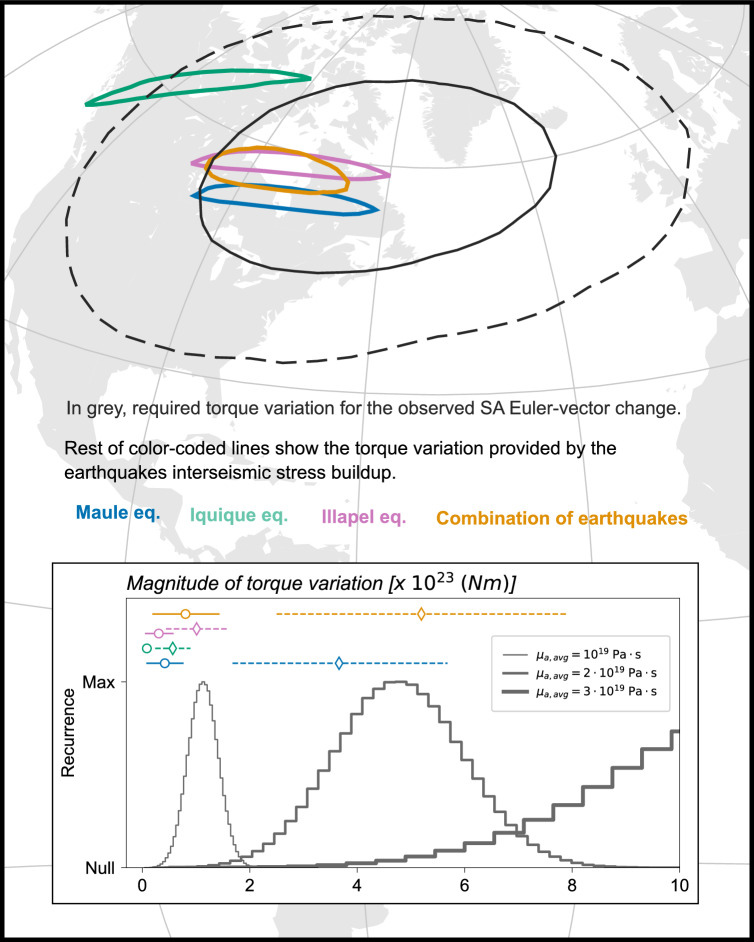
Same as Fig. 7, but showing the comparison between the torque-variation ensembles required upon, and imparted to, SA. This figure has been produced partially using Generic Mapping Tools version 6^76^.

We compare distributions of torque-variation ensembles required upon, and imparted to, NZ in Fig. 7; while Fig. 8 shows the same comparison for SA. For each plate, the overlap of distributions of required and imparted torque-variation poles indicates that the force required to cause the plate-motion slowdown is oriented in the same direction of the force generated by the earthquakes’ interseismic stress buildup upon each plate. Furthermore, despite the impact that parameter uncertainties and trade-off between parameters have onto the torque-variation estimates (i.e. asthenosphere viscosity, stress-drop value, fraction of interseismic stress buildup over the GNSS observation interval), there is agreement also between the distributions of magnitude of required and imparted torque-variations — for the latter one, solid/dashed lines in the insets of Figs. 7, 8 refer to the cases of pro-rata/whole interseismic stress described above. This indicates that indeed the buildup of interseismic stress occurred along the Andean megathrust between 1996 and 2009 is capable of slowing down the convergence motion of NZ and SA during the same interval of time. We note that variations of parameter values within their uncertainties, as well as trade-offs between parameters that we accounted for would affect only the magnitude of the torque variation, and not the location of the torque-variation pole, by a degree (separation between dashed and solid lines) that would still support the plausibility of the working hypothesis. These inferences also hold when considering NZ and SA Euler-vector changes obtained from GNSS data collected over 4-year-long periods (see Supplementary Information).

## Discussion

The existence of contemporary changes in the NZ and SA motions offers an additional perspective to study and interpret differences between the geodetic and geologically-recent NZ/SA velocities illustrated above (Fig. 6a). One interpretation of such differences is to regard the contemporary NZ/SA motion as a point-in-time sample of a longer-term, temporally-variable kinematic pattern that is, at least in part, controlled by stress variations associated with the megathrust earthquake cycle. In this view, the geologically-recent NZ/SA motion — i.e. the one inferred for the stage from present-day to 0.78 Ma (see supplementary data of Quiero et al.^66^) — represents an estimate of the motion averaged over tens of thousands earthquake cycles that, combined together, rupture multiple times the entire Andean megathrust. It seems thus logical to ask the following question: how does the torque variation needed to explain the difference between contemporary and geologically-recent NZ/SA Euler vectors compare to the torque variation one may expect from temporal stress variations during megathrust earthquake cycles? To this end, we use the same parameterisation above, and estimate that the former torque variation is in ranged from $2 \times 10^{23}$ to $8 \times 10^{23}$ $\mathrm {N\cdot m}$. Dividing by Earth’s radius — i.e. the arm of the torque variation — and an assumed rupture area of around 18000 $\mathrm {km^2}$ — i.e. estimated rupture of the 2010 $M_w = 8.8$ Maule earthquake — yields a stress in range from 1 to 7 MPa. This remains well aligned to global catalogues of large earthquakes^29^.

**Figure 9:**
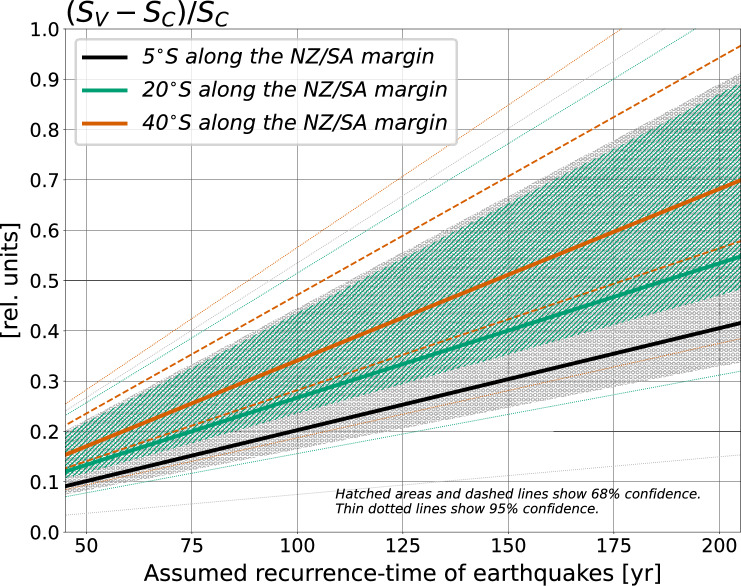
Relative difference between baseline slip estimated at three latitudes along the NZ/SA margins assuming (i) steadiness of NZ/SA convergence rate through time (slip indicated as $S_C$), and (ii) temporal variability of convergence rate (slip indicated as $S_V$). $S_C$ is estimated as the product between average convergence rate between 1996 and 2009 (see Fig. 6) and earthquake recurrence time (taken as a free parameter, see horizontal axis). $S_V$ is estimated similarly, but the convergence rate is assumed to vary through time by up to 6 mm/yr in 10 years, in line with the difference between 1996–1999 and 2006–2009 convergence rates inferred here from GNSS data. 68% and 95% confidence ranges have been calculated from ensembles of convergence rate estimates.

An alternative choice to finite-fault slip models in order to determine interseismic torque-variations is the use of Andean subduction-zone coupling models^18,21,71,72^, which estimate the degree of locking along the megathrust, and are utilised to identify sections experiencing significant accumulation of stress. These models, however, assume plate-motion steadiness, which this study challenges. In fact, the plausibility of the working hypothesis tested here has implications for estimates of slip deficit along megathrust segments^21^. It is standard practice to compare the amount of slip occurred during large earthquakes with a baseline expectation calculated from relative plate motions, and to consider any discrepancy as a proxy for the seismic potential of megathrust segments. It is also standard practice to calculate slip baseline values as the product between local plate motion — i.e. the plate convergence across the specific megathrust segment — and the time interval since the occurrence of the last great earthquake along the same segment. Scientists calculate contemporary plate-convergence values by pooling together GNSS time–series collected over decade-long periods, without necessarily requiring temporal overlap among time–series^7,8,47^. In fact, such a criterion is often deliberately — and understandably, given the limited temporal extent of some GNSS observations — relaxed in order to be able to constrain Euler vectors, and thus plate convergence, from a larger pool of GNSS data. This practice carries the implicit assumption that — contrary to what this study documents — there are no temporal variations of plate motions during the period for which GNSS data are available. Under this premise, we assess by how much could slip baseline values calculated assuming temporal steadiness of NZ/SA convergence — in the following referred to as $S_C$ — depart from values that instead account for temporal variations as high as those reported here — referred to as $S_V$. We first estimate $S_C$ using NZ/SA convergence rates calculated as average of rates inferred from GNSS data for the periods 1996–1999 and 2006–2009. Next, we estimate $S_V$ using a convergence rate that has been systematically slowing down during the assumed recurrence–time of megathrust earthquakes, at a slowdown-rate of about 2–6 mm/yr every 10 years (see Fig. 6b). In these estimates, the recurrence time of megathrust earthquakes is a free parameter that we take in range between 50 and 200 years, in line with the time intervals between the latest great earthquakes that ruptured the Chilean segment of the Andean megathrust and the previous ones reported in the same region in seismic catalogues^35,70^. Figure 9 shows the ratio $(S_V-S_C)/S_C$ as a function of the assumed recurrence time, calculated at three latitudes along the Andean margin. This analysis suggests that current slip baseline estimates, which are predicated on the notion of NZ/SA convergence steadiness through the earthquake cycle, might depart by as much as 70% from estimates that, instead, would explicitly account for temporal variability of convergence during the earthquake cycle. We leave to future studies to explore what are the implications of the above onto slip–deficit estimates. Nonetheless, we speculate that the feedback between megathrust earthquake cycle and plate convergence documented here might serve as basis for alternative models of hazard assessment.

## Methods

The methods presented below are reprised from previous studies (see references). They are reported here for completeness and reproducibility.

### Torque variations required to change plate motions

In order to calculate the torque variation required to modify the kinematics of a plate, we use analytical equations derived in previous studies^22,28^. They result from posing the torque-balance condition at two moments in time ($t_1$ and $t_2$), and computing the difference between them^28^. Doing so simplifies the problem by reducing the torque terms solely to those that have actually changed from $t_1$ to $t_2$. The resulting equation links the torque variation $\Delta \vec {M}$, experienced by a tectonic plate of basal area *S*, to the resulting change of Euler vector $\Delta \vec {\omega }$ that describes the plate motion on the globe:

1 $$\begin{aligned} \Delta \vec {M} = \int _{S} \frac{\mu _a}{H_a} \cdot \vec {r} \times [\Delta \vec {\omega } \times \vec {r}] \cdot dS \end{aligned}$$

where $\mu _a$ and $H_a$ are the viscosity and thickness of the asthenosphere, respectively, $\vec {r}$ is the position vector, *S* is the plate basal area and $\Delta \vec {\omega } = \vec {\omega }(t_2) - \vec {\omega } (t1)$. Since $\vec {r}$ and $\Delta \vec {\omega }$ are mutually independent, the integral can be written as the result of a linear map

2 $$\begin{aligned} \int _{S} \frac{\mu _a}{H_a} \cdot \vec {r} \times [\Delta \vec {\omega } \times \vec {r}] \cdot dS = \begin{pmatrix} \int _{S} \frac{\mu _a}{H_a}(y^2+z^2)dS &{} -\int _{S} \frac{\mu _a}{H_a}xydS &{} -\int _{S} \frac{\mu _a}{H_a}xzdS \\ -\int _{S} \frac{\mu _a}{H_a}xydS &{} \int _{S} \frac{\mu _a}{H_a}(x^2+z^2)dS &{} -\int _{S} \frac{\mu _a}{H_a}yzdS \\ -\int _{S} \frac{\mu _a}{H_a}xzdS &{} -\int _{S} \frac{\mu _a}{H_a}yzdS &{} \int _{S} \frac{\mu _a}{H_a}(x^2+y^2)dS \end{pmatrix} \cdot \Delta \vec {\omega } \end{aligned}$$

or

3 $$\begin{aligned} \underbrace{{\begin{pmatrix} \Delta M_x \\ \Delta M_y \\ \Delta M_z \end{pmatrix}}}_{\Delta \vec {M}} = \underbrace{\begin{pmatrix} \int _{S} \frac{\mu _a}{H_a} (y^2+z^2)dS &{} -\int _{S} \frac{\mu _a}{H_a} xydS &{} -\int _{S} \frac{\mu _a}{H_a} xzdS \\ -\int _{S} \frac{\mu _a}{H_a} xydS &{} \int _{S} \frac{\mu _a}{H_a} (x^2+z^2)dS &{} -\int _{S} \frac{\mu _a}{H_a} yzdS \\ -\int _{S} \frac{\mu _a}{H_a} xzdS &{} -\int _{S} \frac{\mu _a}{H_a} yzdS &{} \int _{S} \frac{\mu _a}{H_a} (x^2+y^2)dS \end{pmatrix}}_{\textbf{P}} \underbrace{{\begin{pmatrix} \Delta \omega _x \\ \Delta \omega _y \\ \Delta \omega _z \end{pmatrix}}}_{\Delta \vec {\omega }} \end{aligned}$$

where the operator ${\textbf{P}}$ connects the vector space of Euler-vector changes experienced by a tectonic plate to that of torque variations imparted to the plate. Equation (3) can also be written on a simplified form

4 $$\begin{aligned} \Delta \vec {M} = {\textbf{P}} \Delta \vec {\omega } \end{aligned}$$

${\textbf{P}}$ depends on the viscosity and thickness of the asthenosphere underneath the plate. To calculate the asthenosphere thickness, we utilise inferences from recent studies of long–wavelength glacial rebound data^73,74^. They establish that, at the global scale, the cube of the asthenosphere thickness ($H_a$) is proportional to the viscosity contrast between the asthenosphere ($\mu _a$) and the lower part of the upper mantle ($\mu _m$). That is, $H_a = a \cdot (\mu _a/\mu _m)^{1/3}$, with the average value of *a* being $4.73 \cdot 10^5~\textrm{m}$. In our calculations, we set $\mu _m$ to $1.5 \cdot 10^{21}~\mathrm {Pa \cdot s}$. Furthermore, we implement lateral variations of the asthenosphere viscosity mapped by the tomography model PM_v2_2012 by Priestly & McKenzie^75^ (see Table 1 and Eqs. (1), (15), and (17) in their study). The Supplementary Information reports figures illustrating the depth–averaged asthenosphere viscosity underneath NZ and SA mapped when assuming a global average of the asthenosphere viscosity equal to $10^{19}~\mathrm {Pa \cdot s}$, $2 \cdot 10^{19}~\mathrm {Pa \cdot s}$, and $3 \cdot 10^{19}~\mathrm {Pa \cdot s}$.

### Torque variations imparted to plates by the interseismic stress buildup

We estimate the torque variation imparted to a tectonic plate by the interseismic buildup of stress following the methodology proposed by Martin de Blas et al.^26^ and Iaffaldano et al.^27^. The approach builds on the notion that the torque variation associated with the entire interseismic phase preceding an earthquake is equal in magnitude but opposite in direction to that associated with the coseismic stress drop during the earthquake. The latter one is estimated by assuming a linear relationship between the coseismic slip and stress drop on the rupture area. The maximum stress drop $\Delta \sigma _{max}$, which is associated with a maximum coseismic slip $s_{max}$, is a free parameter of the model. The torque variation can be thus estimated as:

5 $$\begin{aligned} \Delta \vec {M}_{C} = \int _{\Sigma _r}{\vec {r} \times \left[ \Delta \sigma _{max} \cdot \frac{s(\vec {r})}{s_{max}} \cdot {{\hat{u}}}_r (\vec {r}) \right] \, d\Sigma _r } \end{aligned}$$

where $\Sigma _r$ is the rupture area, $s(\vec {r})$ is the slip at the rupture-area location (relative to Earth’s centre) $\vec {r}$, and ${{\hat{u}}}_r (\vec {r})$ is the rake direction at position $\vec {r}$. We take $\Delta \sigma _{max}$ in range from 1 to 7 MPa, in line with global statistics^11,29^. The torque variation associated with the entire interseismic stress buildup is then:

6 $$\begin{aligned} \Delta \vec {M}_{I} = - \Delta \vec {M}_{C} = - \int _{\Sigma _r}{\vec {r} \times \left[ \Delta \sigma _{max} \cdot \frac{s(\vec {r})}{s_{max}} \cdot {{\hat{u}}}_r (\vec {r}) \right] \, d\Sigma _r } \end{aligned}$$

Equation (6) effectively represents an upper-limit to the torque variation that could be imparted to a tectonic plate during part of the interseismic phase preceding an earthquake. A lower-limit could be estimated as well under the assumption that the interseismic stress grows linearly through time. The fraction of $\Delta \vec {M}_{I}$ accrued over an interval of time $\Delta t_w$ shorter than the length of the interseismic phase $\gamma$ may be calculated as

7 $$\begin{aligned} \Delta \vec {M}_{I} = -\frac{\Delta t_w}{\gamma } \int _{\Sigma _r}{\vec {r} \times \left[ \Delta \sigma _{max} \cdot \frac{s(\vec {r})}{s_{max}} \cdot {{\hat{u}}}_r (\vec {r}) \right] \, d\Sigma _r } \end{aligned}$$

In some of our calculations, we make the assumption above, and set $\Delta t_w=10~$yr (i.e. the period of time covered by GNSS data). We also set $\gamma$ to be 175 years for the Maule earthquake, 133 years for the Iquique earthquake and 67 for the Illapel earthquake (see main text for details). We take values of other parameters in Eqs. (6) and (7) — i.e. $s(\vec {r})$, $\Sigma _r$, and ${{\hat{u}}}_r (\vec {r})$ — from models of finite-fault rupture of the 2010 Maule, 2014 Iquique, and 2015 Illapel earthquakes^32,67,68^, which are inferred from teleseismic wave observations and are available via the public SRCMOD repository^34^ (see also http://equake-rc.info/srcmod/).

Lastly, the torque variation arising from simultaneous buildup of interseismic stress associated with the three earthquakes can be estimated as the summation of the contributions from each earthquake separately:

8 $$\begin{aligned} \Delta \vec {M}_{I,tot} = \sum _{i=1}^{3} \; -\frac{\Delta t_w}{\gamma _i} \int _{\Sigma _{r,i}}{\vec {r} \times \left[ \Delta \sigma _{max,i} \cdot \frac{s(\vec {r})}{s_{max,i}} \cdot {{\hat{u}}}_r (\vec {r}) \right] \, d\Sigma _{r,i} } \end{aligned}$$

where the index $i=1,2,3$ refers to the 2010 Maule, the 2014 Iquique, and the 2015 Illapel earthquakes, respectively.

### Supplementary Information

Supplementary Information 1.

Supplementary Information 2.

Supplementary Information 3.

Supplementary Information 4.

Supplementary Information 5.

Supplementary Information 6.

Supplementary Information 7.

Supplementary Information 8.

Supplementary Information 9.

## Data Availability

All data and methods used in this study are described and/or referenced herein. GNSS data used here are openly available from the Nevada Geodetic Laboratory archive (http://geodesy.unr.edu/)^50^. GNSS surface velocities were obtained using the MIDAS software^53^. Some of the figures were generated using Generic Mapping Tools version 6^76^ (https://www.generic-mapping-tools.org/).

## References

[1] Wilson JT (1965). A new class of faults and their bearing on continental drift. Nature.

[2] McKenzie DP, Parker RL (1967). The North Pacific: An example of tectonics on a sphere. Nature.

[3] Morgan WJ (1968). Rises, trenches, great faults, and crustal blocks. J. Geophys. Res..

[4] Le Pichon X (1968). Sea-floor spreading and continental drift. J. Geophys. Res..

[5] Govers R, Furlong K, van de Wiel L, Herman M, Broerse T (2018). The geodetic signature of the earthquake cycle at subduction zones: Model constraints on the deep processes. Rev. Geophys..

[6] DeMets C, Gordon RG, Argus DF (2010). Geologically current plate motions. Geophys. J. Int..

[7] Argus DF (2010). The angular velocities of the plates and the velocity of Earth’s centre from space geodesy. Geophys. J. Int..

[8] Kreemer C, Blewitt G, Klein EC (2014). A geodetic plate motion and global strain rate model. Geochem. Geophys. Geosyst..

[9] Scholz CH (2019). The Mechanics of Earthquakes and Faulting.

[10] Stein S, Wysession M (2003). An Introduction to Seismology, Earthquakes, and Earth Structure.

[11] Kanamori H, Brodsky EE (2004). The physics of earthquakes. Rep. Prog. Phys..

[12] Lapusta N, Rice JR, Ben-Zion Y, Zheng G (2000). Elastodynamic analysis for slow tectonic loading with spontaneous rupture episodes on faults with rate- and state-dependent friction. J. Geophys. Res. Solid Earth.

[13] Lyakhovsky V, Ben-Zion Y, Agnon A (2001). Earthquake cycle, fault zones, and seismicity patterns in a rheologically layered lithosphere. J. Geophys. Res. Solid Earth.

[14] Bizzarri A, Cocco M (2006). A thermal pressurization model for the spontaneous dynamic rupture propagation on a three-dimensional fault: 1. Methodological approach. J. Geophys. Res. Solid Earth.

[15] Jiang J, Bock Y, Klein E (2021). Coevolving early afterslip and aftershock signatures of a San Andreas fault rupture. Sci. Adv..

[16] Klinger Y (2000). Slip rate on the Dead Sea transform fault in northern Araba valley (Jordan). Geophys. J. Int..

[17] Fialko Y (2006). Interseismic strain accumulation and the earthquake potential on the southern San Andreas fault system. Nature.

[18] Moreno M (2011). Heterogeneous plate locking in the South-Central Chile subduction zone: Building up the next great earthquake. Earth Planet. Sci. Lett..

[19] Nocquet J-M (2017). Supercycle at the Ecuadorian subduction zone revealed after the 2016 Pedernales earthquake. Nat. Geosci..

[20] Herman MW, Govers R (2020). Locating fully locked asperities along the South America subduction megathrust: A new physical interseismic inversion approach in a Bayesian framework. Geochem. Geophys. Geosyst..

[21] Graham SE, Loveless JP, Meade BJ (2021). A global set of subduction zone earthquake scenarios and recurrence intervals inferred from geodetically constrained block models of interseismic coupling distributions. Geochem. Geophys. Geosyst..

[22] Martin de Blas J, Iaffaldano G (2019). Using rigid microplate motions to detect the stress buildup preceding large earthquakes: A feasibility test based on synthetic models. J. Geophys. Res. Solid Earth.

[23] Dixon TH (1991). An introduction to the global positioning system and some geological applications. Rev. Geophys..

[24] Segall P, Davis JL (1997). GPS applications for geodynamics and earthquake studies. Annu. Rev. Earth Planet. Sci..

[25] Bock Y, Melgar D (2016). Physical applications of GPS geodesy: A review. Rep. Prog. Phys..

[26] Martin de Blas J, Iaffaldano G, Calais E (2022). Have the 1999 Izmit-Düzce earthquakes influenced the motion and seismicity of the Anatolian microplate?. Geophys. J. Int..

[27] Iaffaldano G, Martin de Blas J, Dali Udbø ÍB (2022). Decadal change of the Apulia microplate motion preceding the MW 6.4, 26 November 2019 Durrës (Albania) earthquake. Earth Planet. Sci. Lett..

[28] Iaffaldano G, Bunge H-P (2015). Rapid plate motion variations through geological time: Observations serving geodynamic interpretation. Annu. Rev. Earth Planet. Sci..

[29] Allmann B, Shearer P (2009). Global variations of stress drop for moderate to large earthquakes. J. Geophys. Res..

[30] Gallovič F, Imperatori W, Mai PM (2015). Effects of three-dimensional crustal structure and smoothing constraint on earthquake slip inversions: Case study of the $$Mw$$ 6.3 2009 L’Aquila earthquake. J. Geophys. Res. Solid Earth.

[31] Reilinger RE (2000). Coseismic and Postseismic Fault Slip for the 17 August 1999, M = 7.5, Izmit, Turkey Earthquake. Science.

[32] Hayes GP (2017). The finite, kinematic rupture properties of great-sized earthquakes since 1990. Earth Planet. Sci. Lett..

[33] Hayes GP (2011). Rapid source characterization of the 2011 M w 9.0 off the Pacific coast of Tohoku Earthquake. Earth Planets Space.

[34] Mai PM, Thingbaijam KKS (2014). SRCMOD: An online database of finite-fault rupture models. Seismol. Res. Lett..

[35] Ruiz S, Madariaga R (2018). Historical and recent large megathrust earthquakes in Chile. Tectonophysics.

[36] Madariaga R, Métois M, Vigny C, Campos J (2010). Central Chile finally breaks. Science.

[37] Vigny C (2011). The 2010 $$M_w$$ 8.8 Maule megathrust earthquake of Central Chile, monitored by GPS. Science.

[38] Moreno M (2010). Toward understanding tectonic control on the Mw 8.8 2010 Maule Chile earthquake. Earth Planet. Sci. Lett..

[39] Jara J (2018). Kinematic study of Iquique 2014 Mw 8.1 earthquake: Understanding the segmentation of the seismogenic zone. Earth Planet. Sci. Lett..

[40] Ruiz S (2016). The seismic sequence of the 16 September 2015 Mw 8.3 Illapel, Chile, earthquake. Seismol. Res. Lett..

[41] Sobolev SV, Babeyko AY (2005). What drives orogeny in the Andes?. Geology.

[42] Melnick D (2017). The super-interseismic phase of the megathrust earthquake cycle in Chile. Geophys. Res. Lett..

[43] Cifuentes IL (1989). The 1960 Chilean earthquakes. J. Geophys. Res..

[44] Barrientos SE, Ward SN (1990). The 1960 Chile earthquake: Inversion for slip distribution from surface deformation. Geophys. J. Int..

[45] Angermann D, Klotz J, Reigber C (1999). Space-geodetic estimation of the Nazca-South America Euler vector. Earth Planet. Sci. Lett..

[46] Norabuena EO, Dixon TH, Stein S, Harrison CGA (1999). Decelerating Nazca-South America and Nazca-Pacific plate motions. Geophys. Res. Lett..

[47] Sella GF, Dixon TH, Mao A (2002). REVEL: A model for recent plate velocities from space geodesy. J. Geophys. Res..

[48] Kendrick E (2003). The Nazca-South America Euler vector and its rate of change. J. S. Am. Earth Sci..

[49] Jarrin P (2022). Current motion and deformation of the Nazca plate: New constraints from GPS measurements. Geophys. J. Int..

[50] Blewitt G, Hammond W, Kreemer C (2018). Harnessing the GPS data explosion for interdisciplinary science. EOS.

[51] Blewitt G, Lavallée D (2002). Effect of annual signals on geodetic velocity. J. Geophys. Res..

[52] Blewitt G, Lavallée D, Clarke P, Nurutdinov K (2001). A new global mode of earth deformation: Seasonal cycle detected. Science.

[53] Blewitt G, Kreemer C, Hammond W, Gazeaux J (2016). MIDAS robust trend estimator for accurate GPS station velocities without step detection. J. Geophys. Res..

[54] Altamimi Z, Rebischung P, Métivier L, Collilieux X (2016). ITRF2014: A new release of the International Terrestrial Reference Frame modeling nonlinear station motions: ITRF2014. J. Geophys. Res. Solid Earth.

[55] Rebischung P, Altamimi Z, Ray J, Garayt B (2016). The IGS contribution to ITRF2014. J. Geodesy.

[56] Bevis M, Brown A (2014). Trajectory models and reference frames for crustal motion geodesy. J. Geodesy.

[57] Gordon RG (1998). The plate tectonic approximation: Plate nonrigidity, diffuse plate boundaries, and global plate reconstructions. Annu. Rev. Earth Planet. Sci..

[58] Cox A, Hart RB (1986). Plate Tectonics: How it Works.

[59] Nocquet J-M, Calais E, Altamimi Z, Sillard P, Boucher C (2001). Intraplate deformation in western Europe deduced from an analysis of the International Terrestrial Reference Frame 1997 (ITRF97) velocity field. J. Geophys. Res. Solid Earth.

[60] Stein S, Gordon RG (1984). Statistical tests of additional plate boundaries from plate motion inversions. Earth Planet. Sci. Lett..

[61] Wdowinski S, Bock Y, Zhang J, Fang P, Genrich J (1997). Southern California permanent GPS geodetic array: Spatial filtering of daily positions for estimating coseismic and postseismic displacements induced by the 1992 Landers earthquake. J. Geophys. Res. Solid Earth.

[62] Calais E, Dong L, Wang M, Shen Z, Vergnolle M (2006). Continental deformation in Asia from a combined GPS solution. Geophys. Res. Lett..

[63] Symithe S, Calais E, de Chabalier JB, Robertson R, Higgins M (2015). Current block motions and strain accumulation on active faults in the Caribbean. J. Geophys. Res. Solid Earth.

[64] Norabuena E (1998). Space geodetic observations of Nazca-South America convergence across the Central Andes. Science.

[65] Wang K, Hu Y, He J (2012). Deformation cycles of subduction earthquakes in a viscoelastic earth. Nature.

[66] Quiero F, Tassara A, Iaffaldano G, Rabbia O (2022). Growth of Neogene Andes linked to changes in plate convergence using high-resolution kinematic models. Nat. Commun..

[67] Hayes GP (2013). Seismotectonic framework of the 2010 February 27 Mw 8.8 Maule, Chile earthquake sequence. Geophys. J. Int..

[68] Hayes GP (2014). Continuing megathrust earthquake potential in Chile after the 2014 Iquique earthquake. Nature.

[69] Meltzner AJ (2015). Time-varying interseismic strain rates and similar seismic ruptures on the Nias-Simeulue patch of the Sunda megathrust. Quat. Sci. Rev..

[70] Lomnitz C (2004). Major earthquakes of Chile: A historical survey, 1535–1960. Seismol. Res. Lett..

[71] Métois M, Vigny C, Socquet A (2016). Interseismic coupling, megathrust earthquakes and seismic swarms along the Chilean subduction zone (38$$\,^{\circ }$$-18$$\,^{\circ }$$S). Pure Appl. Geophys..

[72] Saillard M (2017). From the seismic cycle to long-term deformation: Linking seismic coupling and quaternary coastal geomorphology along the Andean megathrust. Tectonics.

[73] Paulson A, Richards MA (2009). On the resolution of radial viscosity structure in modelling long-wavelength postglacial rebound data. Geophys. J. Int..

[74] Richards MA, Lenardic A (2018). The Cathles parameter (Ct): A geodynamic definition of the asthenosphere and implications for the nature of plate tectonics. Geochem. Geophys. Geosyst..

[75] Priestley K, McKenzie D (2013). The relationship between shear wave velocity, temperature, attenuation and viscosity in the shallow part of the mantle. Earth Planet. Sci. Lett..

[76] Wessel P (2019). The generic mapping tools version 6. Geochem. Geophys. Geosyst..

[77] Matthews KJ (2016). Global plate boundary evolution and kinematics since the late Paleozoic. Glob. Planet. Change.

