# Author Correction: Feedback between megathrust earthquake cycle and plate convergence

**DOI:** 10.1038/s41598-023-47731-3

**Published:** 2023-11-24

**Authors:** Juan Martin de Blas, Giampiero Iaffaldano, Andrés Tassara, Daniel Melnick

**Affiliations:** 1 Department of Geosciences and Natural Resource Management, University of Copenhagen, Copenhagen, Denmark; 2 Present Address: Dipartimento di Scienze Chimiche, della Vita e della Sostenibilità Ambientale, Università di Parma, Parma, Italy; 3 Departamento de Ciencias de la Tierra, Universidad de Concepción, Concepción, Chile; 4 Instituto de Ciencias de la Tierra, Universidad Austral de Chile, Valdivia, Chile

Correction to: *Scientific Reports* 10.1038/s41598-023-45753-5, published online 30 October 2023

The Acknowledgements section in the original version of this Article was incomplete.

“This work is part of a project funded by the Independent Research Fund Denmark (grant number: 0135 00351B). The authors gratefully acknowledge Marcos Moreno for his insights and discussions, which contributed to the improvement of this manuscript. JMB acknowledges Valentina Espinoza for her invaluable inputs in multiple aspects of this study.”

now reads:

“This work is part of a project funded by the Independent Research Fund Denmark (grant number: 0135 00351B). AT and DM acknowledge support from the Chilean ANID Milenio Grant NCN19_167—Millennium Nucleus CYCLO. The authors gratefully acknowledge Marcos Moreno for his insights and discussions, which contributed to the improvement of this manuscript. JMB acknowledges Valentina Espinoza for her invaluable inputs in multiple aspects of this study.”

The original Article has been corrected.

