# Supplementary material for: Feedback between megathrust earthquake cycle and plate convergence
Source: Sci Rep. 2023 Oct 30;13:18623. doi: 10.1038/s41598-023-45753-5 (PMC10616103; doi:10.1038/s41598-023-45753-5)
Supplement: Supplementary file 1 — Supplementary Information 1. [file 41598_2023_45753_MOESM1_ESM.pdf]

Feedback between megathrust earthquake cycle and plate convergence

Juan Martin de Blas[1,*], Giampiero Iaffaldano[1], Andrés Tassara[2], and Daniel Melnick[3]

[1]Department of Geosciences and Natural Resource Management, University of Copenhagen, Denmark.
[2]Departamento de Ciencias de la Tierra, Universidad de Concepción, Chile.
[3]Instituto de Ciencias de la Tierra, Universidad Austral de Chile, Valdivia, Chile.
[*]jmb@ign.ku.dk

Supplementary Material

Supplementary files list

Supplementary File 1. Velocities of continuously-recording GNSS stations used to obtain the NZ Euler vectors (relative to IGS14) for the 3-yr time period from July 1996 to June 1999. Columns are: (1) Acronyms of sites within NZ, (2–3) longitude and latitude of site locations (in decimal degrees), (4–5) station East/North velocities (v_e/v_n) relative to IGS14 in mm/yr, and (6–7) standard deviations (σ) of the East/North velocities in mm/yr.

Supplementary File 2. Same as Supplementary File 1, but for the 4-yr-long period from July 1996 to June 2000.

Supplementary File 3. Same as Supplementary File 1, but for the 3-yr-long period from July 2006 to June 2009.

Supplementary File 4. Same as Supplementary File 1, but for the 4-yr-long period from July 2005 to June 2009.

Supplementary File 5. Velocities of continuously-recording GNSS stations used to obtain the SA Euler vectors (relative to IGS14) for the 3-yr time period from July 1996 to June 1999. Columns are: (1) Acronyms of sites within SA, (2–3) longitude and latitude of site locations (in decimal degrees), (4–5) station East/North velocities (v_e/v_n) relative to IGS14 in mm/yr, and (6–7) standard deviations (σ) of the East/North velocities in mm/yr.

Supplementary File 6. Same as Supplementary File 5, but for the 4-yr-long period from July 1996 to June 2000.

Supplementary File 7. Same as Supplementary File 5, but for the 3-yr-long period from July 2006 to June 2009.

Supplementary File 8. Same as Supplementary File 5, but for the 4-yr-long period from July 2005 to June 2009.

Supplementary tables

Supplementary Table 1. Euler vectors ($\vec{\omega}$) describing the NZ/IGS14 motions for the two first and last time periods considered on this study. We present the Euler pole location (in decimal degrees), the angular velocity (in $^\circ$ Myr^{-1}), the Euler vector Cartesian elements, and the associated covariance matrix C values (in 10^{-9} rad^2 Myr^{-2}).

Time period	lon	lat	ω	ω_x	ω_y	ω_z	c_{xx}	c_{xy}	c_{xz}	c_{yy}	c_{yz}	c_{zz}
[yrs]	[$^\circ E$]	[$^\circ N$]	[$^\circ$ Myr^{-1}]	[10^{-2} $^\circ$ Myr^{-1}]			[10^{-9} rad^2 Myr^{-2}]					
1996.50–1999.49	-103.644	42.578	0.661	-11.47	-47.27	44.70	14.3	21.7	6.3	142.4	35.6	22.9
2006.50–2009.49	-103.382	44.680	0.632	-10.40	-43.73	44.46	3.4	4.1	0.7	37.0	5.9	3.9
1996.50–2000.49	-103.083	42.436	0.660	-11.02	-47.41	44.51	13.0	21.2	6.7	117.9	33.2	21.3
2005.50–2009.49	-102.846	45.140	0.630	-9.87	-43.31	44.64	2.4	2.7	0.5	25.7	4.1	2.7

Supplementary Table 2. Euler vectors ($\vec{\omega}$) describing the SA/IGS14 motions for the two first and last time periods considered on this study. We present the Euler pole location (in decimal degrees), the angular velocity (in $^\circ$ Myr^{-1}), the Euler vector Cartesian elements, and the associated covariance matrix C values (in 10^{-9} rad^2 Myr^{-2}).

Time period	lon	lat	ω	ω_x	ω_y	ω_z	c_{xx}	c_{xy}	c_{xz}	c_{yy}	c_{yz}	c_{zz}	Stations excluded
[yrs]	[$^\circ E$]	[$^\circ N$]	[$^\circ$ Myr^{-1}]	[10^{-2} $^\circ$ Myr^{-1}]			[10^{-9} rad^2 Myr^{-2}]						
1996.50–1999.49	-133.259	-27.867	0.127	-7.67	-8.17	-5.93	50.4	-46.7	-18.5	63.2	21.5	22.6	None
2006.50–2009.49	-123.111	-18.560	0.125	-6.49	-9.95	-3.99	1.8	-1.8	-0.8	2.5	1.0	0.7	BOMJ, BRFT, BUE1, BUE2, CRA1, KOUR, RWSN, TUCU, UCOR, UNSA
1996.50–2000.49	-124.605	-23.563	0.122	-6.35	-9.21	-4.88	37.0	-36.8	-15.2	45.7	17.2	13.5	None
2005.50–2009.49	-122.449	-19.949	0.122	-6.17	-9.71	-4.18	6.8	-7.5	-5.5	9.2	6.4	4.9	BUE1, KOUR, PRMA, RWSN TUCU, UCOR, UNSA

Supplementary Table 3. Euler vectors ($\vec{\omega}$) describing the NZ/SA motions for the two first and last time periods considered on this study. We present the Euler pole location (in decimal degrees), the angular velocity (in $^\circ$ Myr^{-1}), the Euler vector Cartesian elements, and the associated covariance matrix C values (in 10^{-9} rad^2 Myr^{-2}).

Time period	lon	lat	ω	ω_x	ω_y	ω_z	c_{xx}	c_{xy}	c_{xz}	c_{yy}	c_{yz}	c_{zz}
[yrs]	[$^\circ E$]	[$^\circ N$]	[$^\circ$ Myr^{-1}]	[10^{-2} $^\circ$ Myr^{-1}]			[10^{-9} rad^2 Myr^{-2}]					
1996.50–1999.49	-95.546	52.180	0.641	-3.79	-39.11	50.63	64.7	-25.0	-12.2	205.5	56.9	45.4
2006.50–2009.49	-96.614	54.925	0.592	-3.92	-33.79	48.44	5.1	2.3	-0.1	39.4	6.9	4.6
1996.50–2000.49	-96.962	52.067	0.626	-4.67	-38.21	49.38	50.0	-15.7	-8.6	163.7	50.4	34.7
2005.50–2009.49	-96.289	55.296	0.594	-3.70	-33.60	48.81	9.2	-4.8	-5.0	34.8	10.5	7.6

Supplementary Table 4. Results of the F-ratio test performed on pairs of Euler vectors from Supplementary Tables 1 and 2.

Plate	Euler vector 1	Euler vector 2	F-ratio value	Probability [%]
NZ/IGS14	07/1996–06/1999	07/2006–06/2009	1.32	45.75
NZ/IGS14	07/1996–06/2000	07/2005–06/2009	0.77	60.81
SA/IGS14	07/1996–06/1999	07/2006–06/2009	12.81	$2 \cdot 10^{-4}$
SA/IGS14	07/1996–06/2000	07/2005–06/2009	2.17	10.34

Supplementary Table 5. Entries of the linear operator **P** in equation 3. Units are 10^{39} Pa · s · m^3.

Plate	μ_a	$\mathbf{P}_{11}$	$\mathbf{P}_{12}$	$\mathbf{P}_{13}$	$\mathbf{P}_{22}$	$\mathbf{P}_{23}$	$\mathbf{P}_{33}$
NZ	$1 \cdot 10^{19}$ Pa · s	4.3	-0.3	-0.1	0.8	-1.3	3.8
NZ	$2 \cdot 10^{19}$ Pa · s	22.7	-1.1	-0.3	4.0	-6.9	19.8
NZ	$3 \cdot 10^{19}$ Pa · s	56.5	-2.2	-0.5	9.9	-17.2	49.5
SA	$1 \cdot 10^{19}$ Pa · s	5.2	2.6	1.4	4.8	-1.5	6.7
SA	$2 \cdot 10^{19}$ Pa · s	18.9	10.2	6.4	22.8	-5.8	27.4
SA	$3 \cdot 10^{19}$ Pa · s	47.1	26.9	17.4	63.0	-14.9	72.3

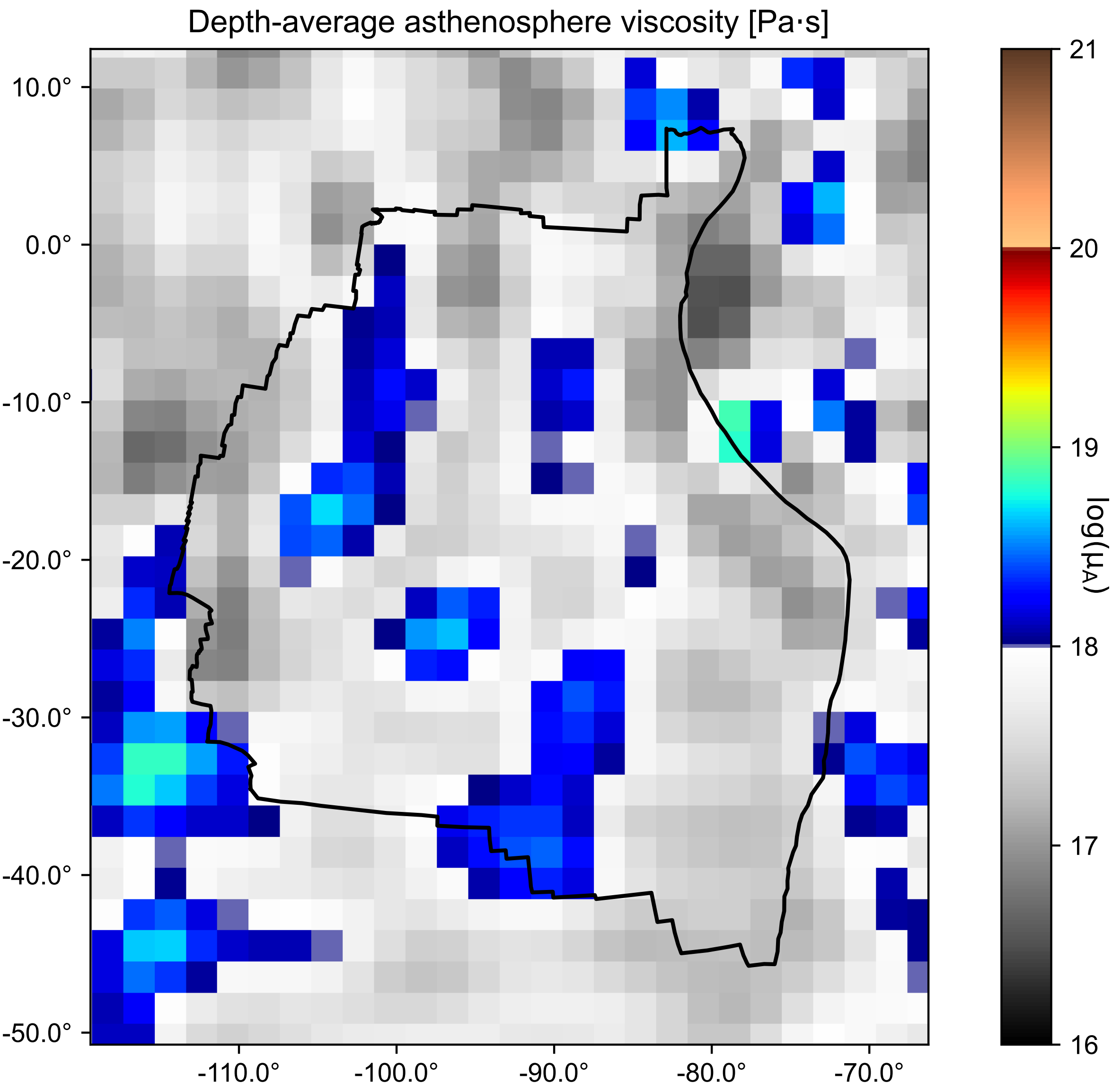

Supplementary Figure 1. Depth–averaged asthenosphere viscosity μ_a underneath NZ, for the case where the global average of μ_a is set to $1 \cdot 10^{19}$ Pa $\cdot$ s. Lateral variations of μ_a are inferred from lateral temperature variations in the seismic tomography model of Priestley & McKenzie[1]. NZ plate boundaries are in thick black.

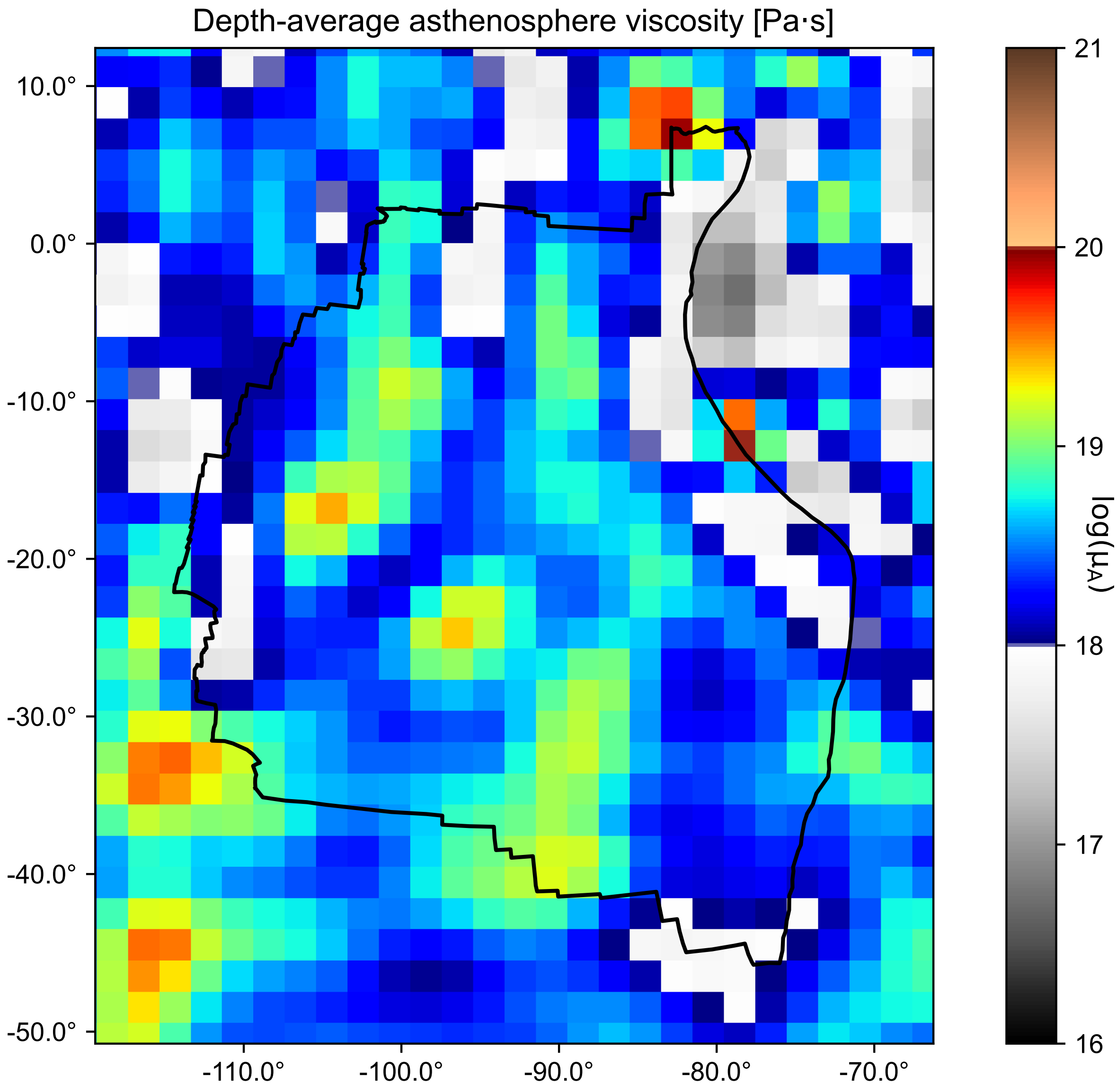

Supplementary Figure 2. As Supplementary Figure 1, for the case where the global average of μ_a is set to $2 \cdot 10^{19}$ Pa · s.

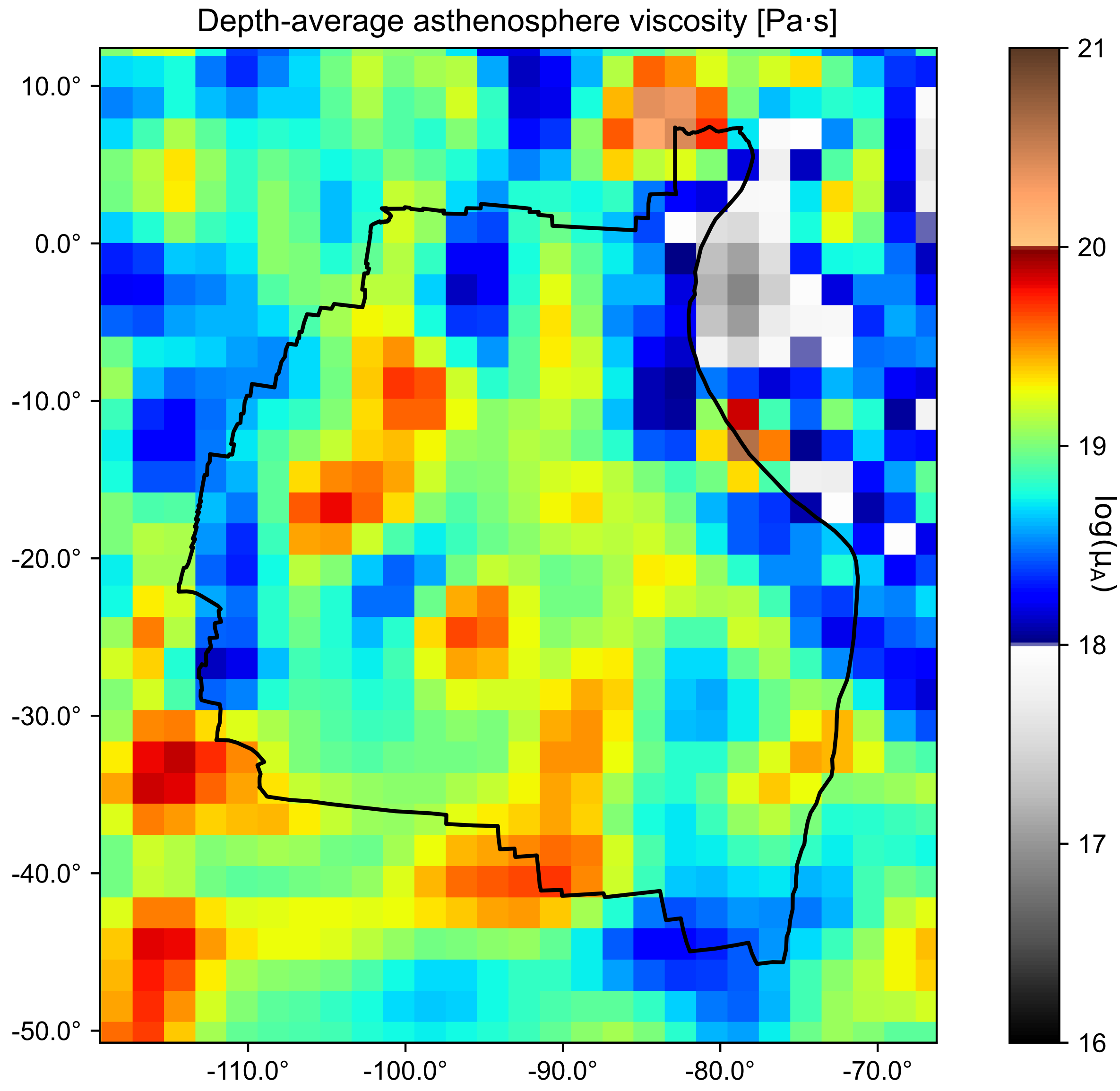

Supplementary Figure 3. As Supplementary Figure 1, for the case where the global average of μ_a is set to $3 \cdot 10^{19}$ Pa · s.

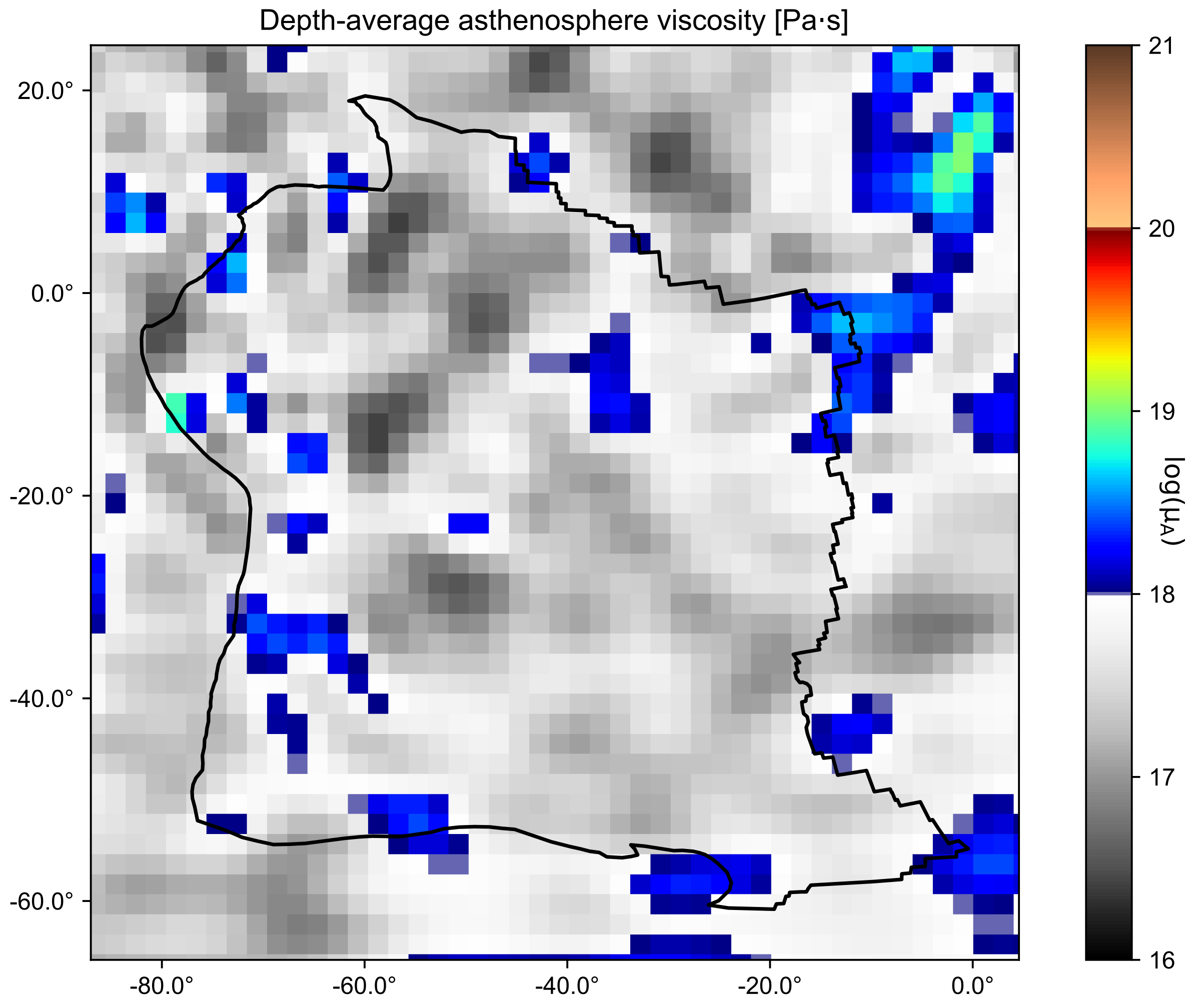

Supplementary Figure 4. Depth–averaged asthenosphere viscosity μ_a underneath SA, for the case where the global average of μ_a is set to $1 \cdot 10^{19}$ Pa·s. Lateral variations of μ_a are inferred from lateral temperature variations in the seismic tomography model of Priestly & McKenzie[1]. SA plate boundaries are in thick black.

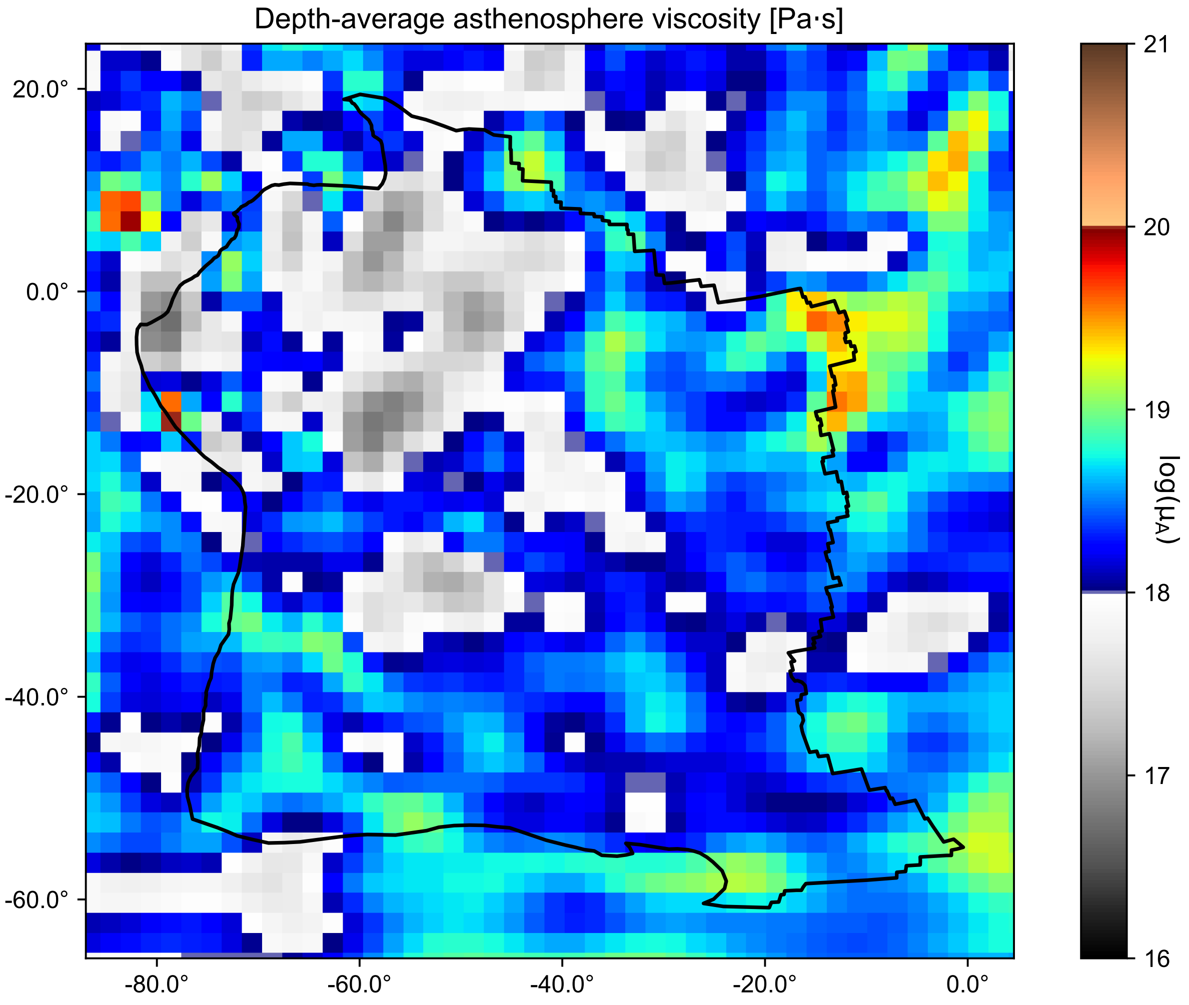

Supplementary Figure 5. As Supplementary Figure 4, for the case where the global average of μ_a is set to $2 \cdot 10^{19}$ Pa · s

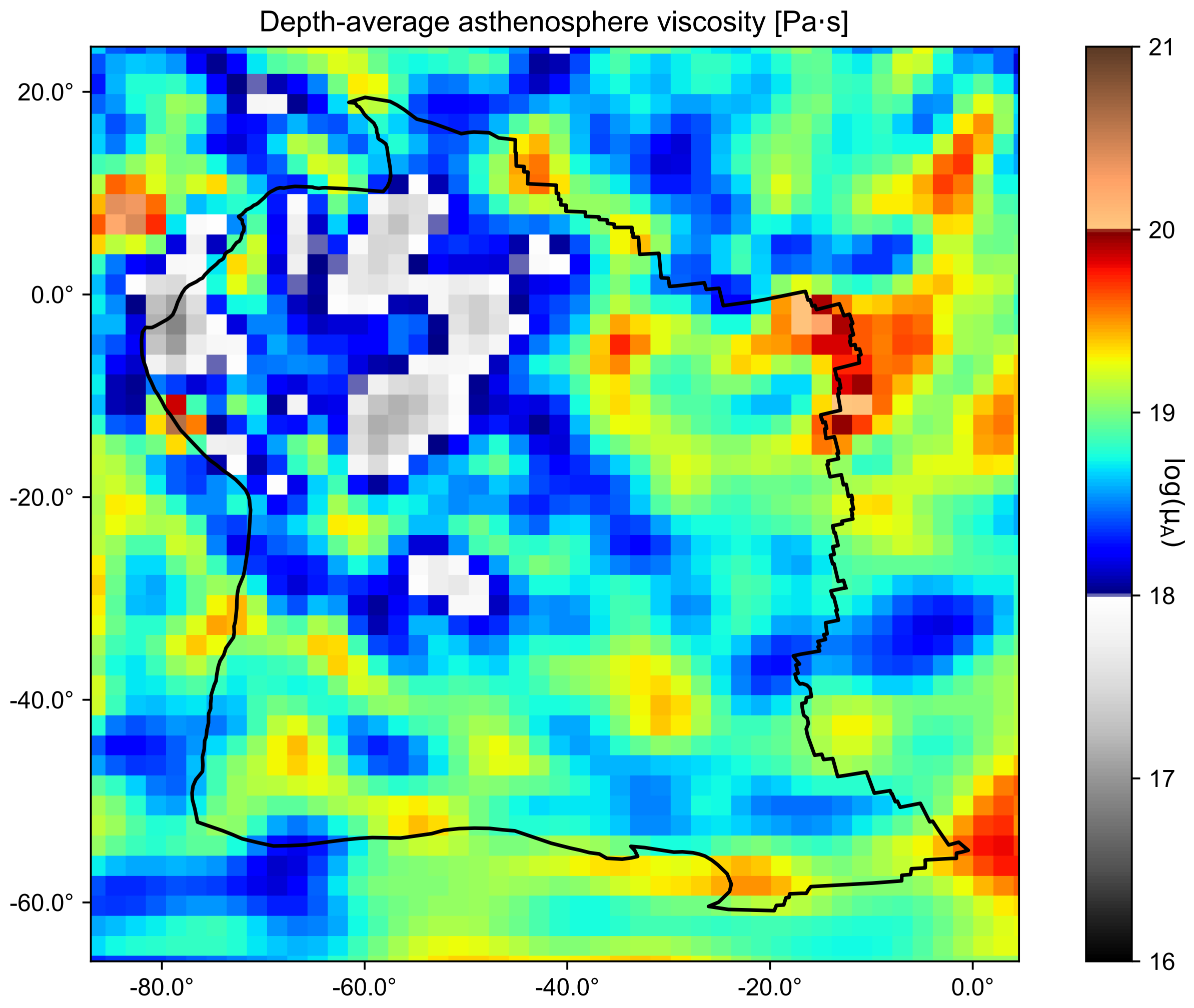

Supplementary Figure 6. As Supplementary Figure 4, for the case where the global average of μ_a is set to $3 \cdot 10^{19}$ Pa · s

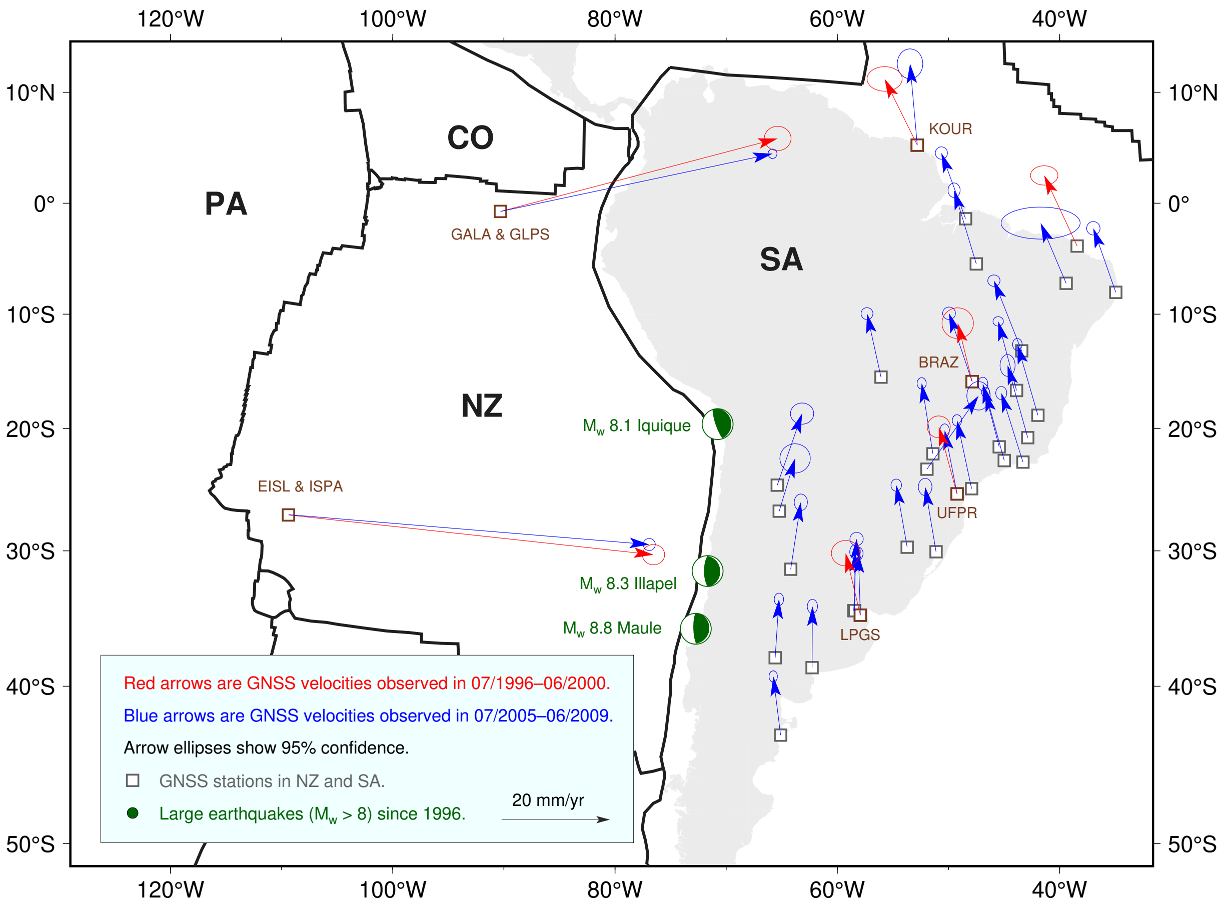

Supplementary Figure 7. Same as Figure 2, but showing the GNSS velocities available in NZ and SA for the periods July 1996—June 2000 and July 2005—June 2009. Please note that not all velocities have been used to constrain the Euler vectors for the latter period in SA, since some sites have been excluded on the basis of their velocity residuals (see Supplementary Figure 14).

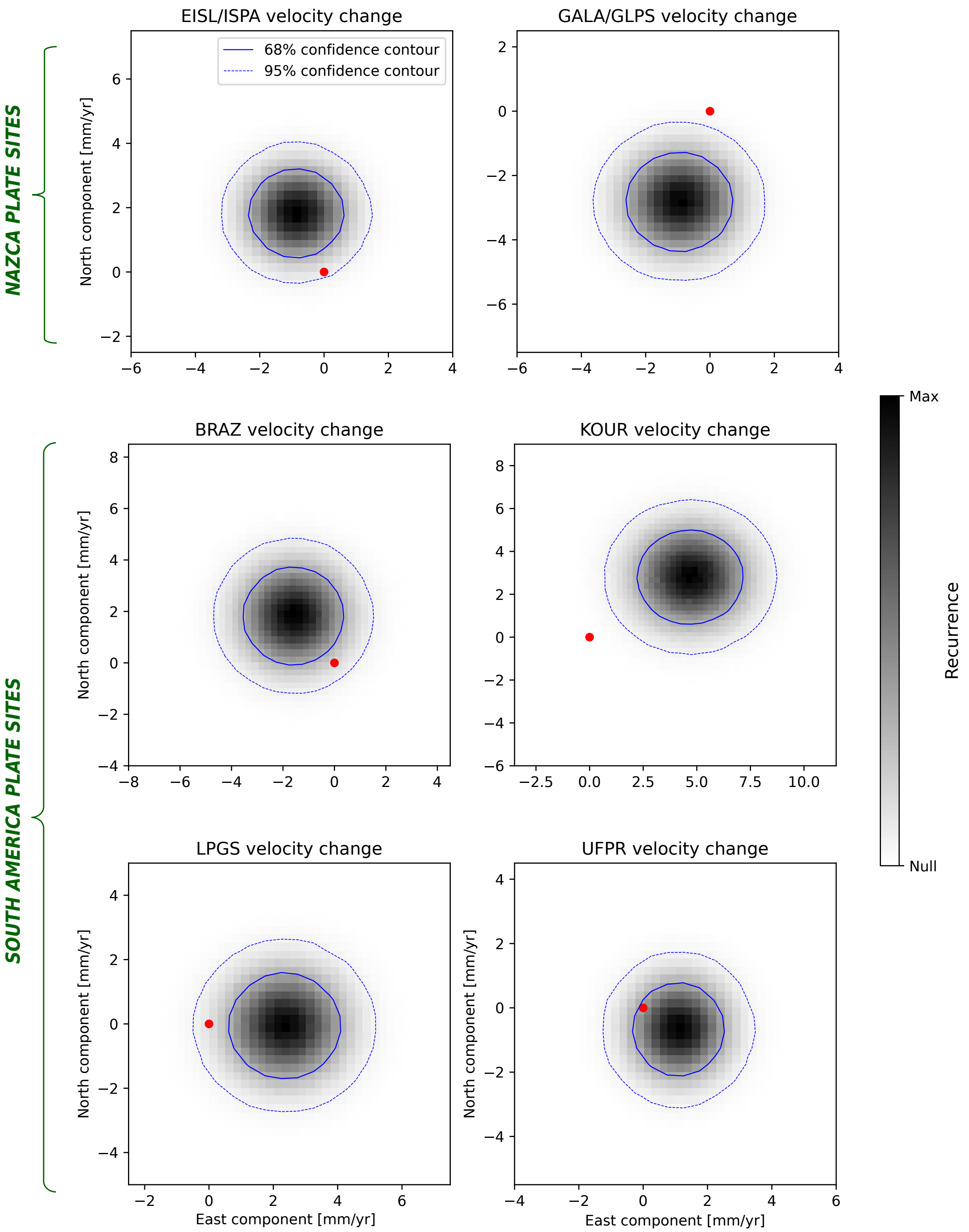

Supplementary Figure 8. Same as Figure 3, but for GNSS stations whose data cover periods spanning 4 years (July 1996—June 2000 and July 2005—June 2009).

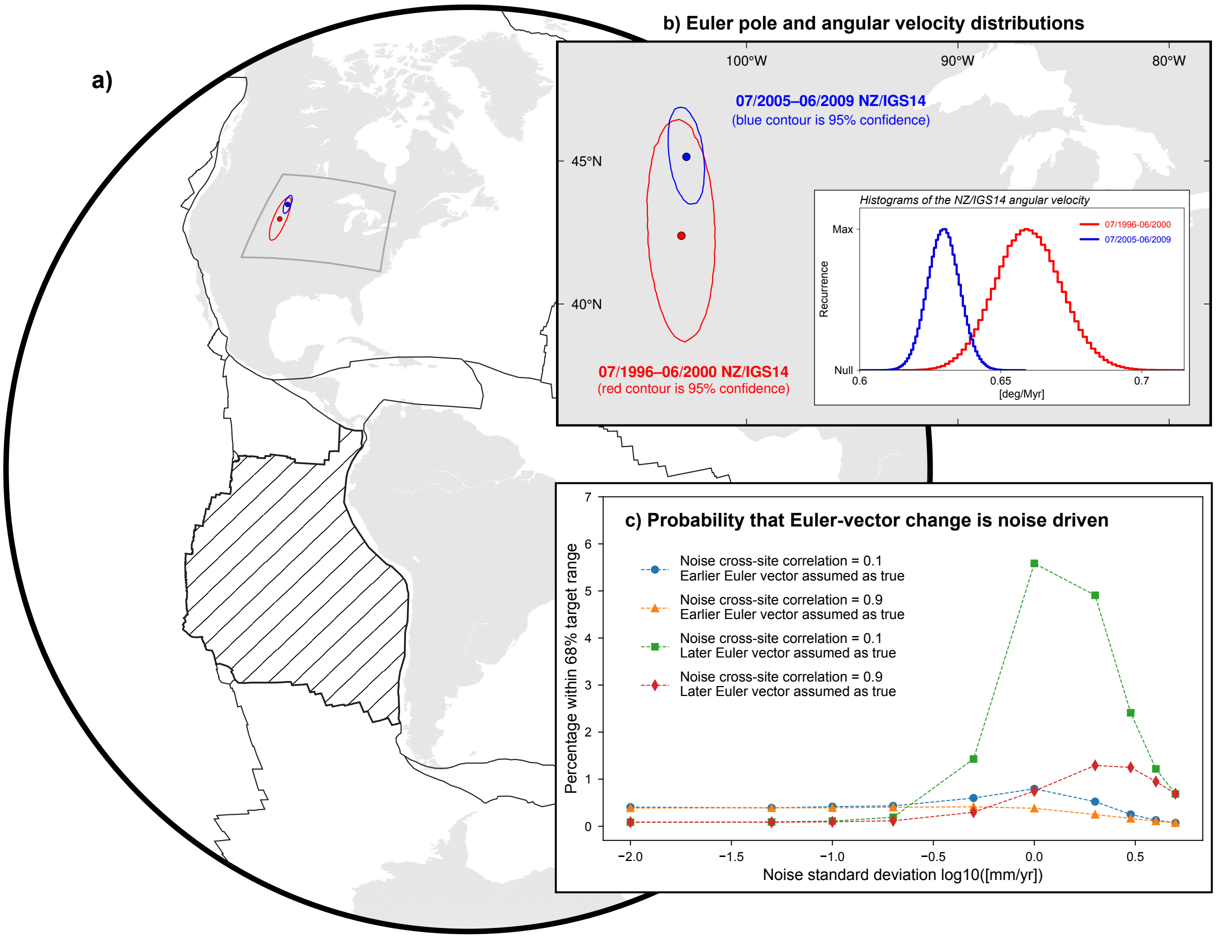

Supplementary Figure 9. Same as Figure 4, but showing the Euler-vector change of NZ relative to IGS14 for the 4-yr-long periods July 1996—June 2000 and July 2005—June 2009.

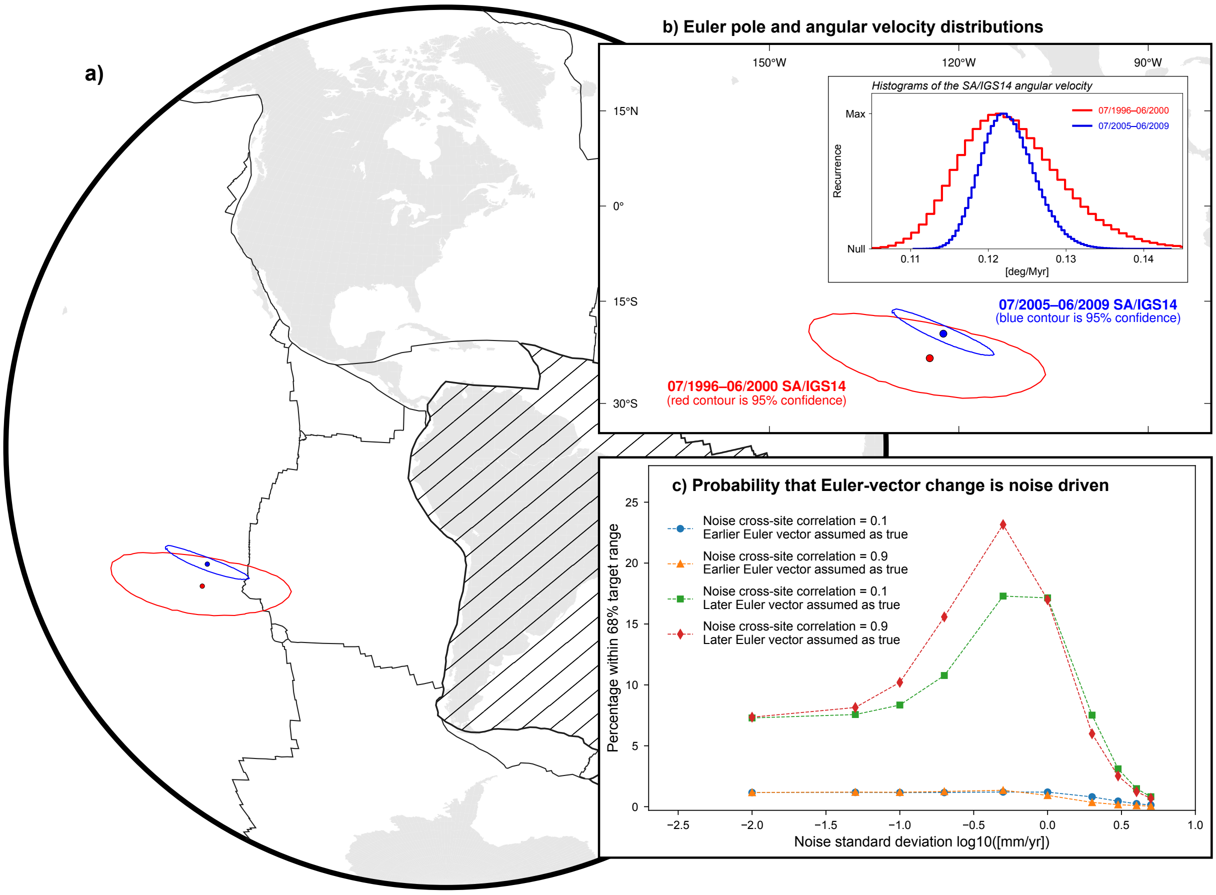

Supplementary Figure 10. Same as Figure 5, but showing the Euler-vector change of SA relative to IGS14 for the 4-yr-long periods July 1996—June 2000 and July 2005—June 2009.

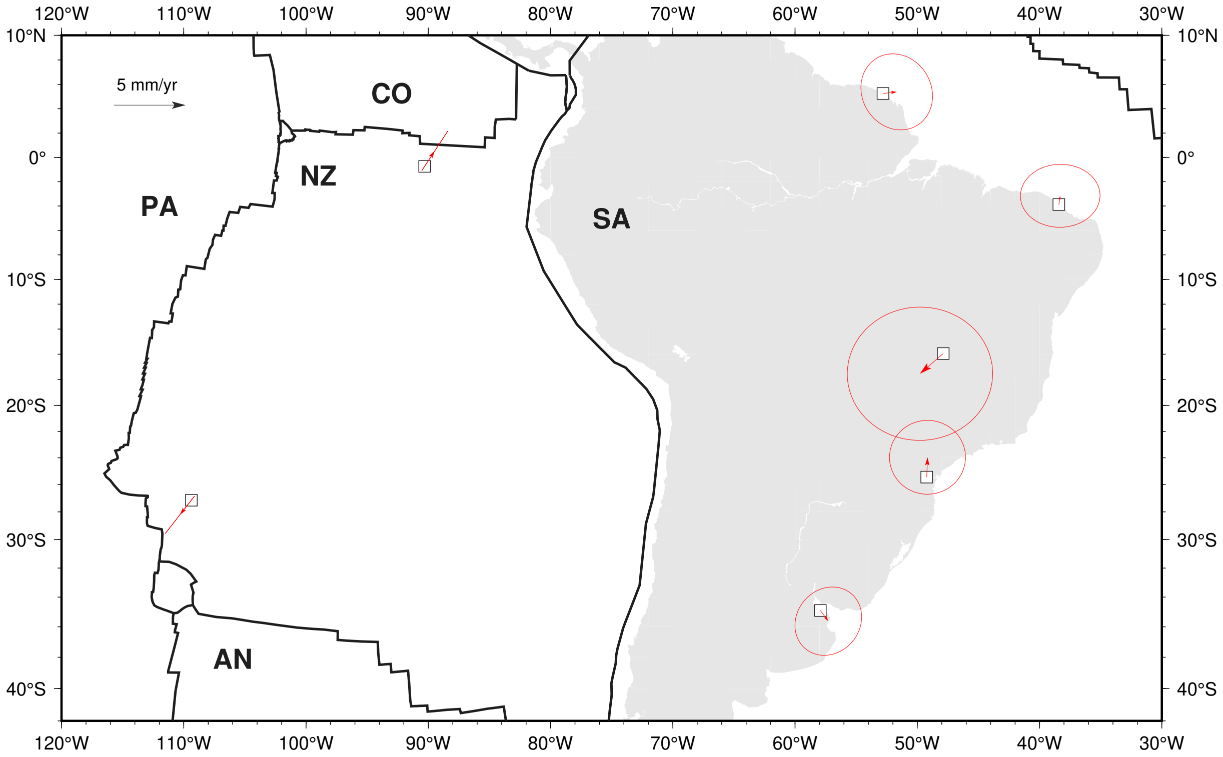

Supplementary Figure 11. Surface velocity residuals of GNSS sites used to infer the NZ/IGS14 and SA/IGS14 Euler vectors for the period from July 1996 to June 1999. Ellipses are 95% confidence.

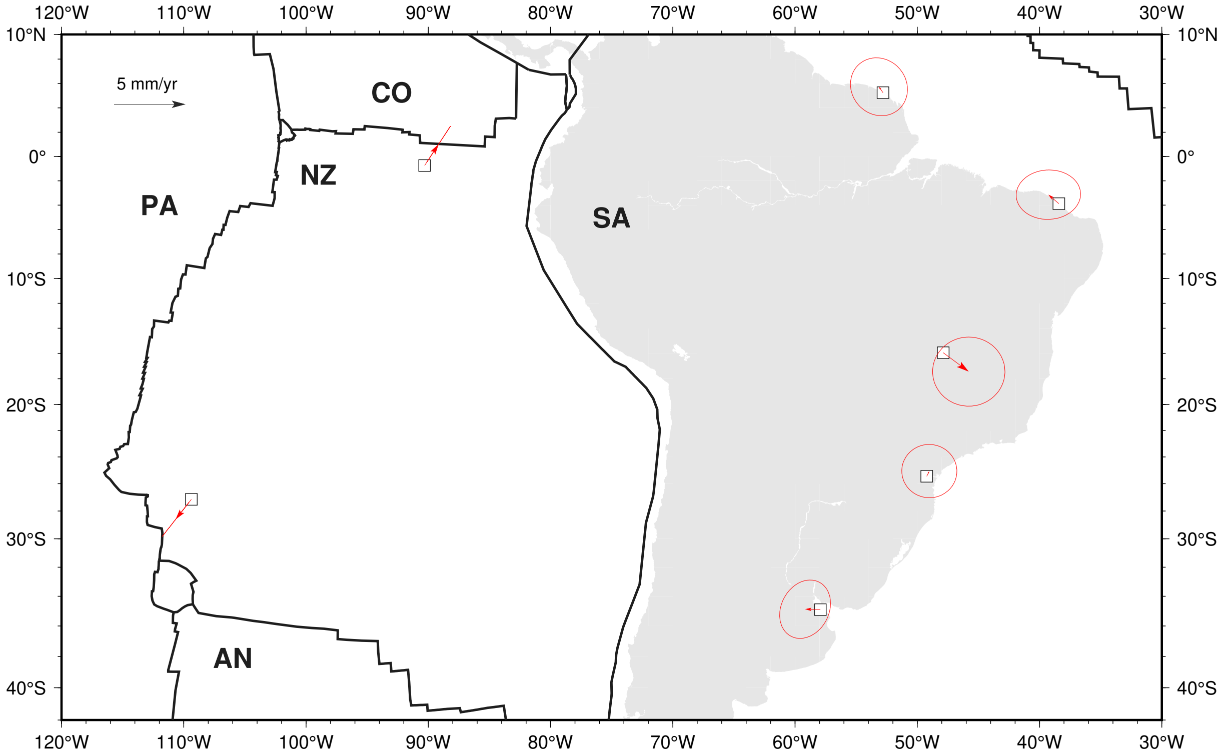

Supplementary Figure 12. Same as Supplementary Figure 11, but for for the period from July 1996 to June 2000.

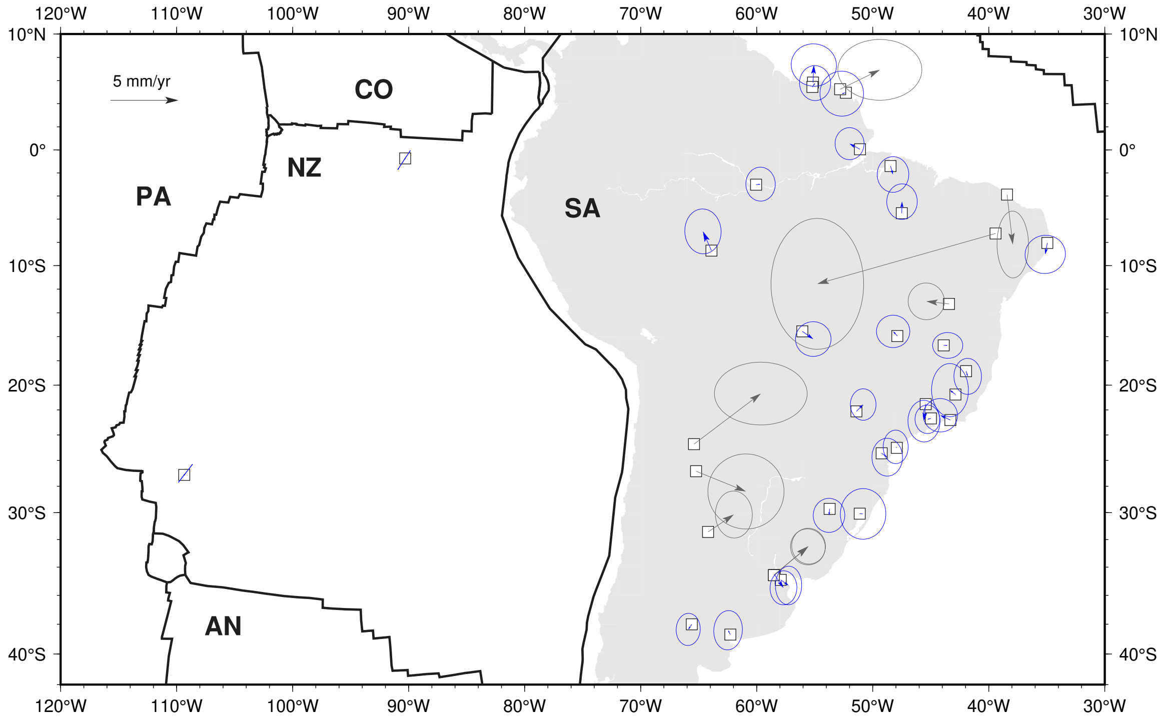

Supplementary Figure 13. Same as Supplementary Figure 11, but for for the period from July 2006 to June 2009. In grey are velocity residuals at GNSS sites within SA not used to constrain the Euler vector, since their velocity-residual values exceed their associated uncertainties.

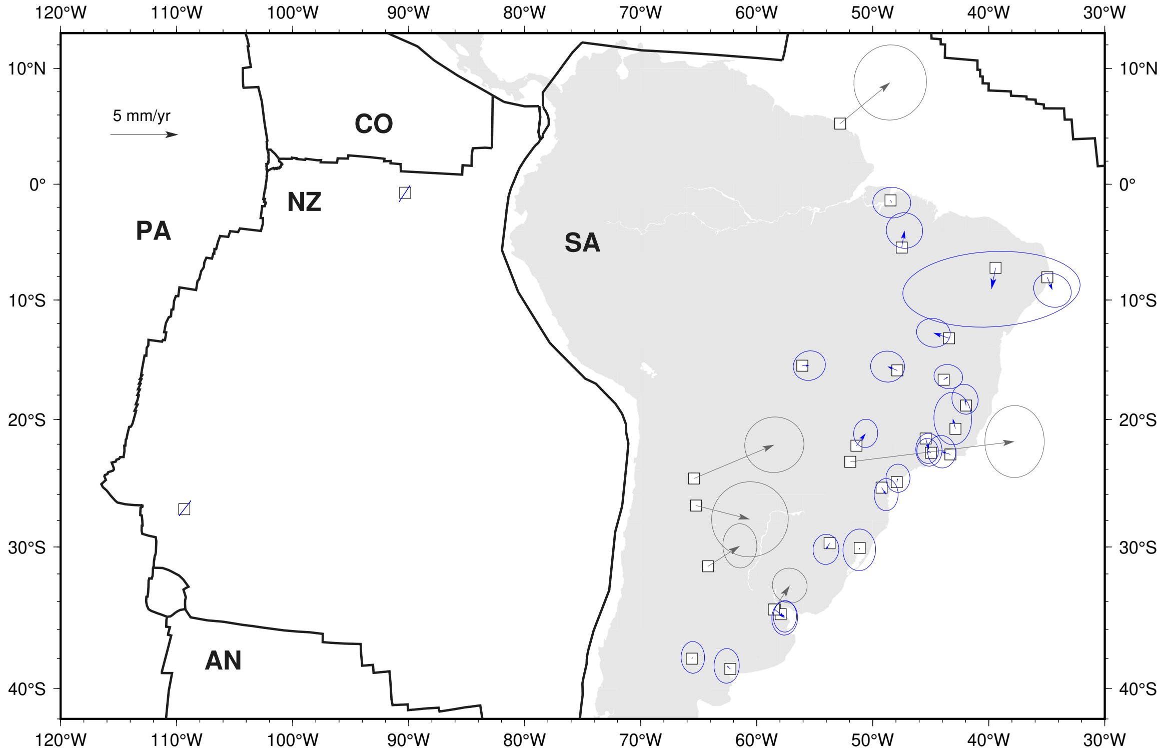

Supplementary Figure 14. Same as Supplementary Figure 11, but for the period from July 2005 to June 2009. In grey are velocity residuals at GNSS sites within SA not used to constrain the Euler vector, since their velocity-residual values exceed their associated uncertainties.

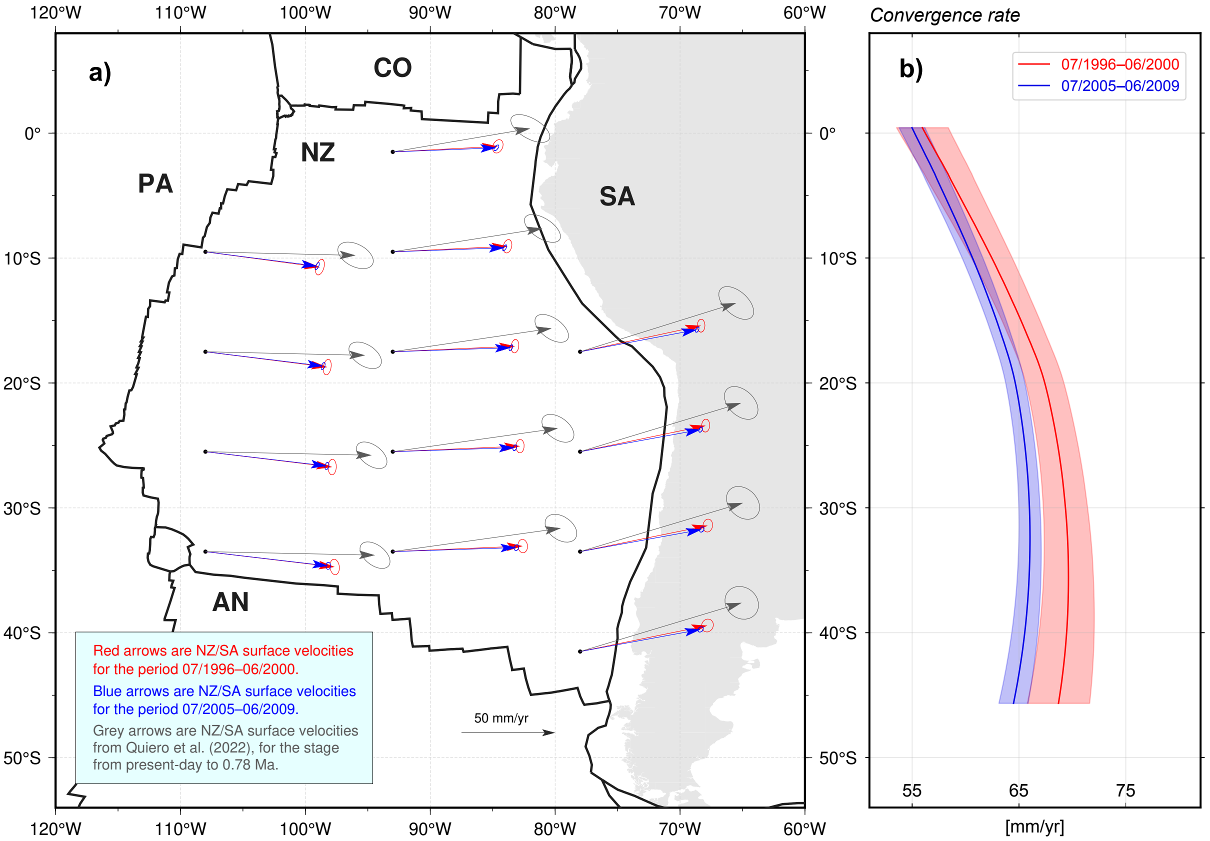

Supplementary Figure 15. As Figure 6, but for the case where the first and last time periods are 4 years long (i.e., from July 1996 to June 2000 and from July 2005 to June 2009).

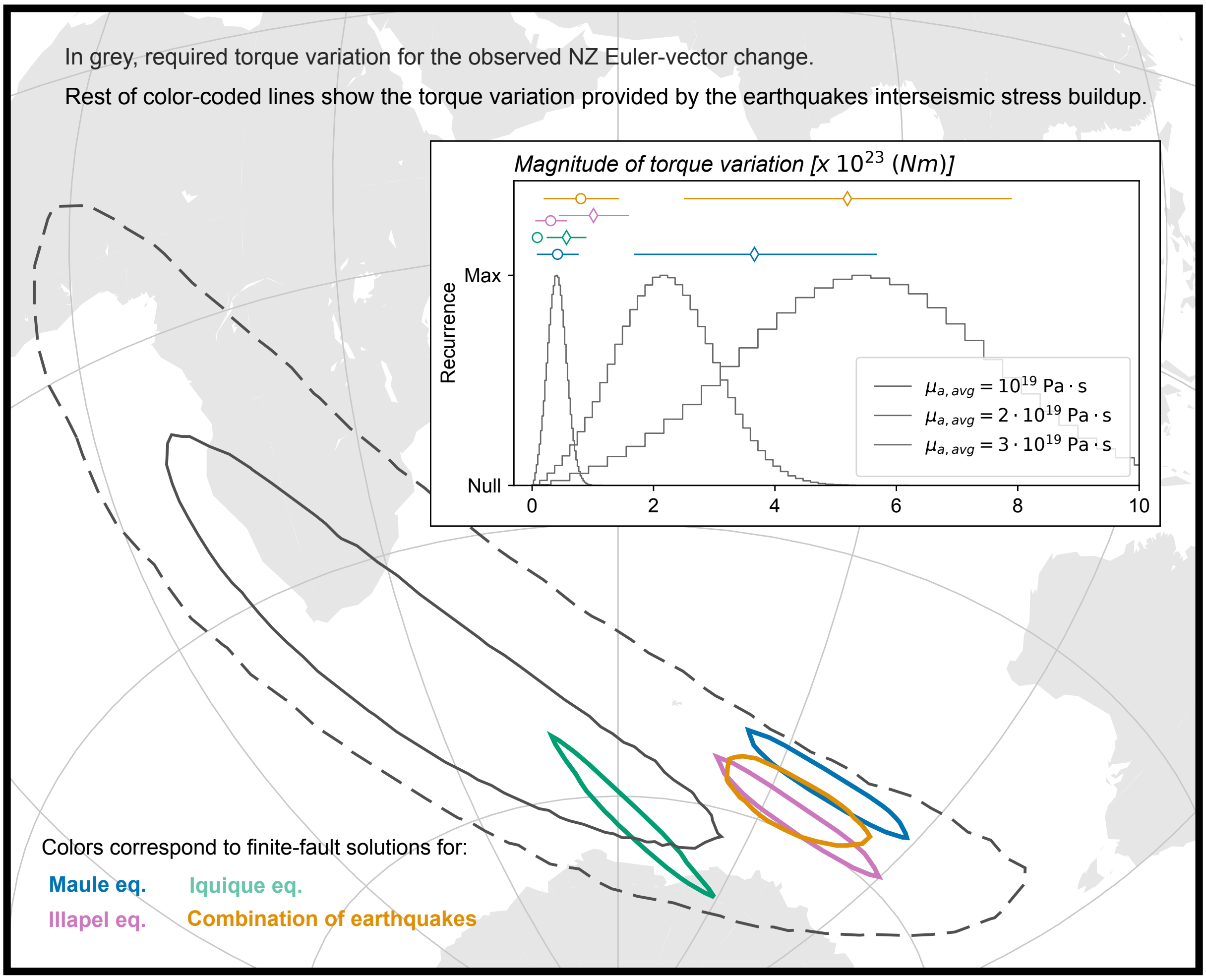

Supplementary Figure 16. As Figure 7, but for the case where the first and last time periods are 4 years long (i.e., from July 1996 to June 2000 and from July 2005 to June 2009).

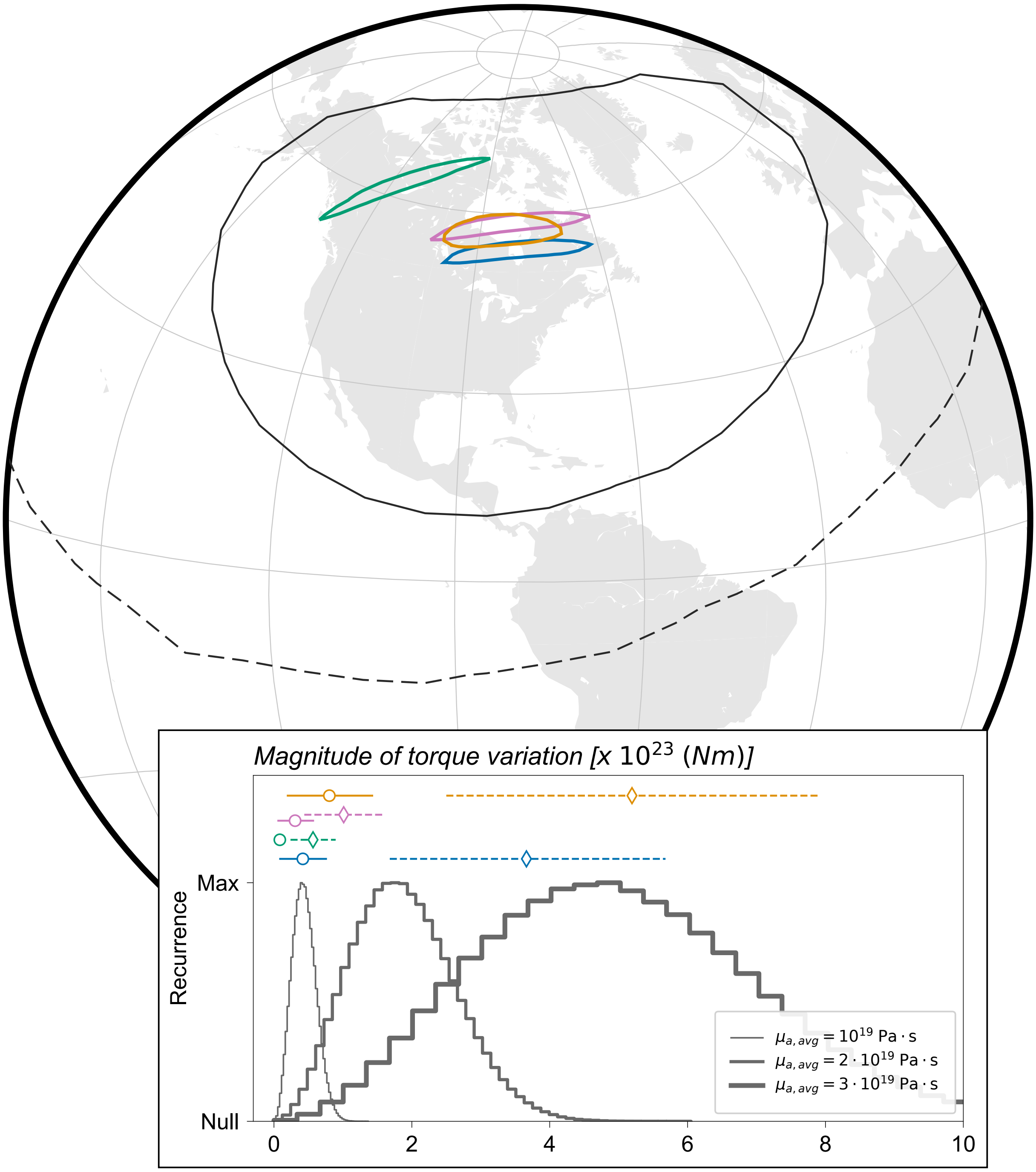

Supplementary Figure 17. As Figure 8, but for the case where the first and last time periods are 4 years long (i.e., from July 1996 to June 2000 and from July 2005 to June 2009).

FORT

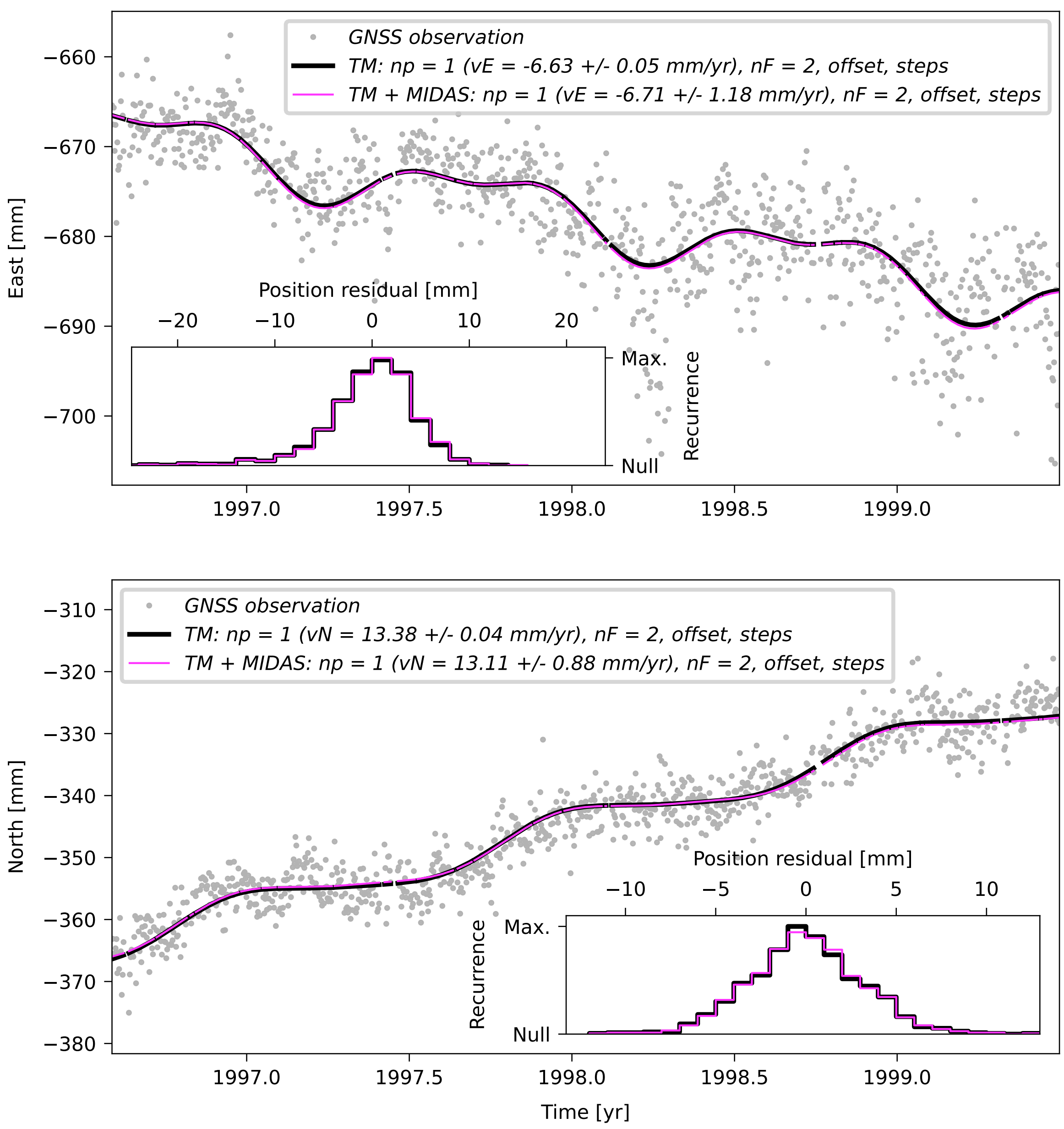

Supplementary Figure 18. East (upper panel) and North (lower panel) of the position time series for GNSS station FORT, for the period from July 1996 to June 1999. Grey dots are daily GNSS observations of the station position, while solid lines illustrate trajectory models (TMs, see main text for details) fitted to the observed time series. In black are TMs as defined by Bevis & Brown [2014][2], which incorporate reference position (offset), linear term (i.e., tectonic velocity, inferred value is reported in the legend), annual and semiannual terms corresponding to periodic signals, as well as the steps at known epochs. In magenta are TMs inferred from a hybrid approach where the station velocity output by MIDAS (value is reported in the legend) is taken as a known, fixed parameter of the TM, while the amplitude of other terms are free parameters of the inversion scheme. Insets show distributions of the residual position (difference between observations and TMs).

BRAZ

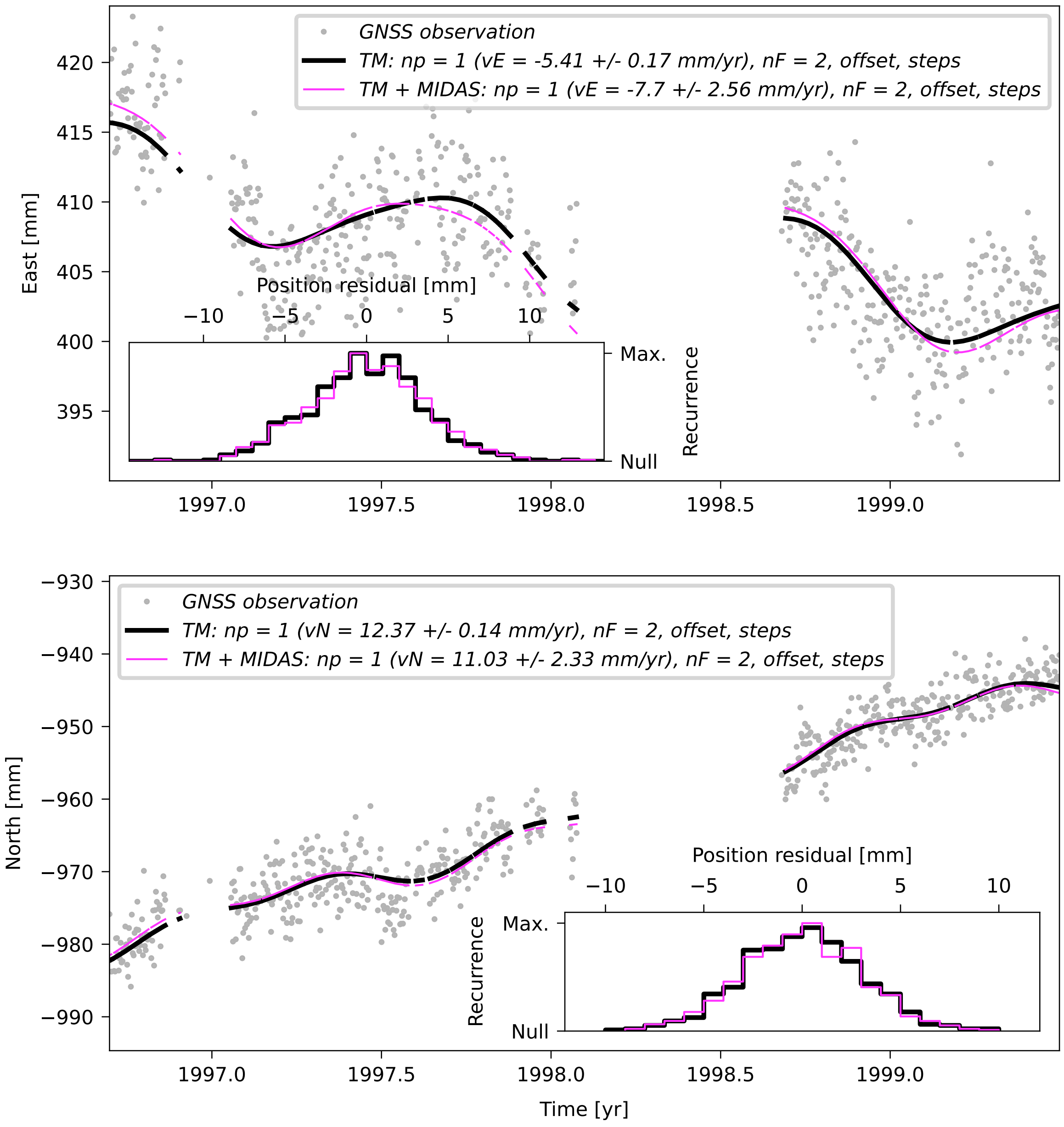

Supplementary Figure 19. Same as Supplementary Figure 18, but showing the time series and TMs of station BRAZ for the period from July 1996 to June 1999.

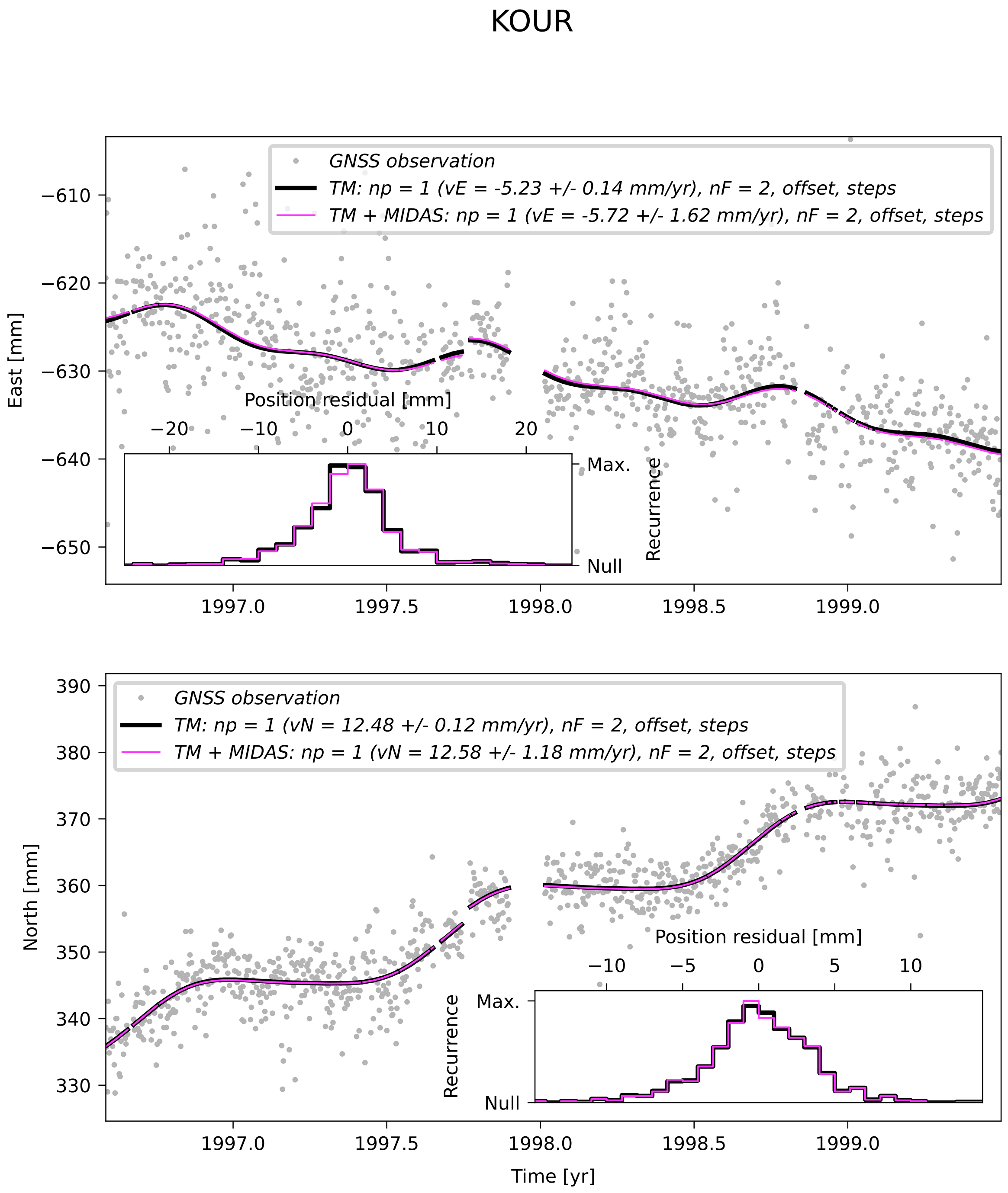

Supplementary Figure 20. Same as Supplementary Figure 18, but showing the time series and TMs of station KOUR for the period from July 1996 to June 1999.

LPGS

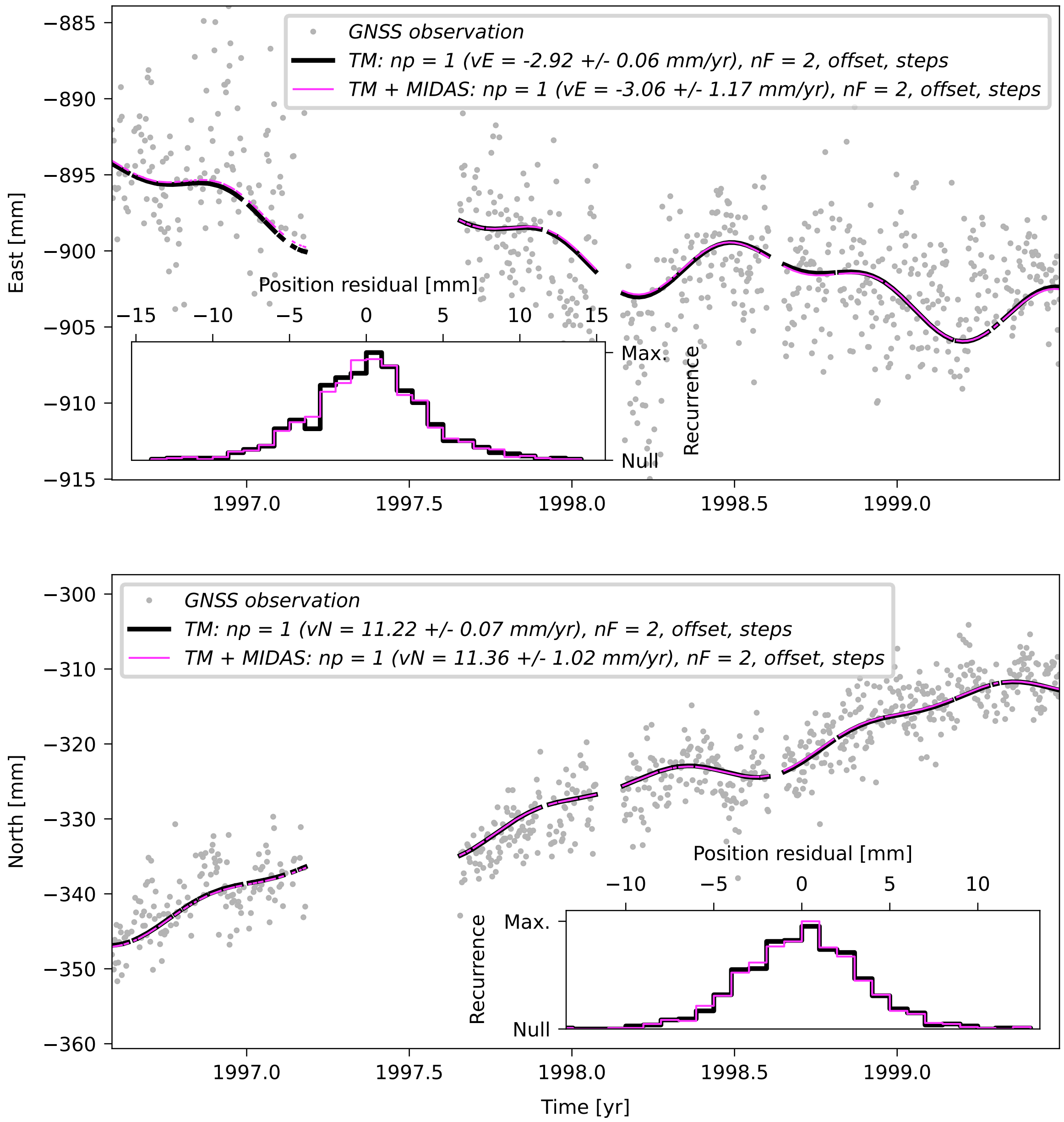

Supplementary Figure 21. Same as Supplementary Figure 18, but showing the time series and TMs of station LPGS for the period from July 1996 to June 1999.

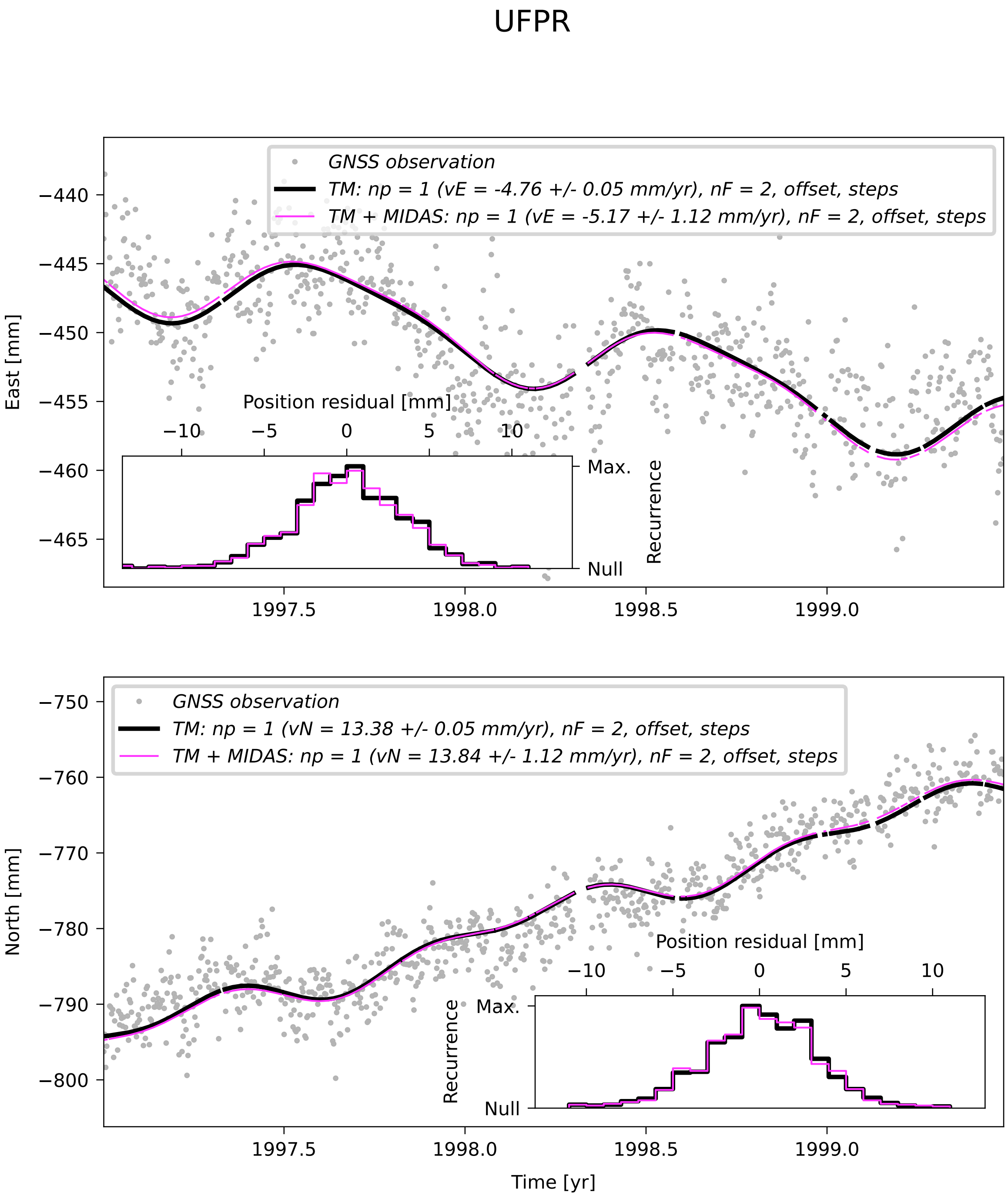

Supplementary Figure 22. Same as Supplementary Figure 18, but showing the time series and TMs of station UFPR for the period from July 1996 to June 1999.

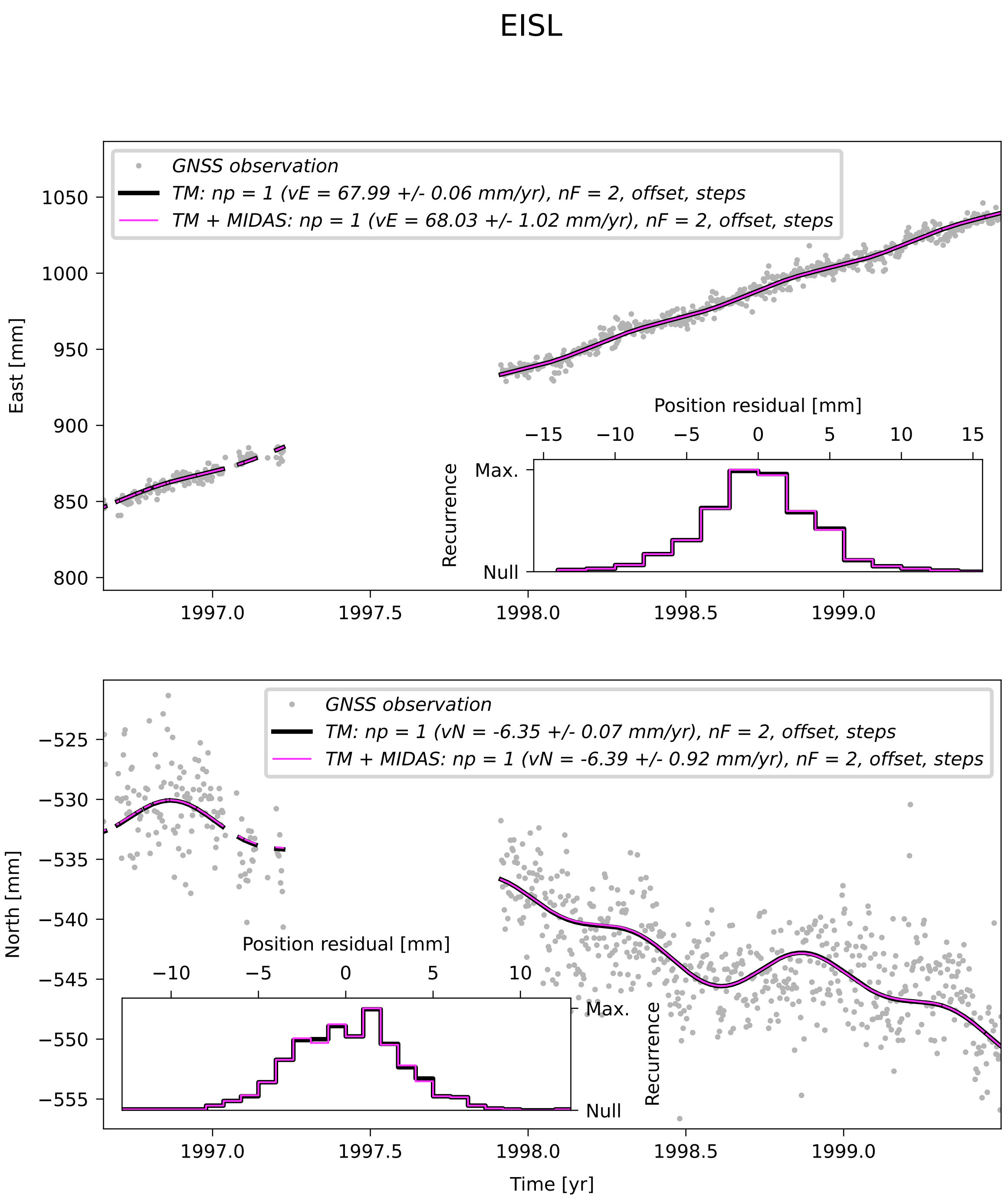

Supplementary Figure 23. Same as Supplementary Figure 18, but showing the time series and TMs of station EISL for the period from July 1996 to June 1999.

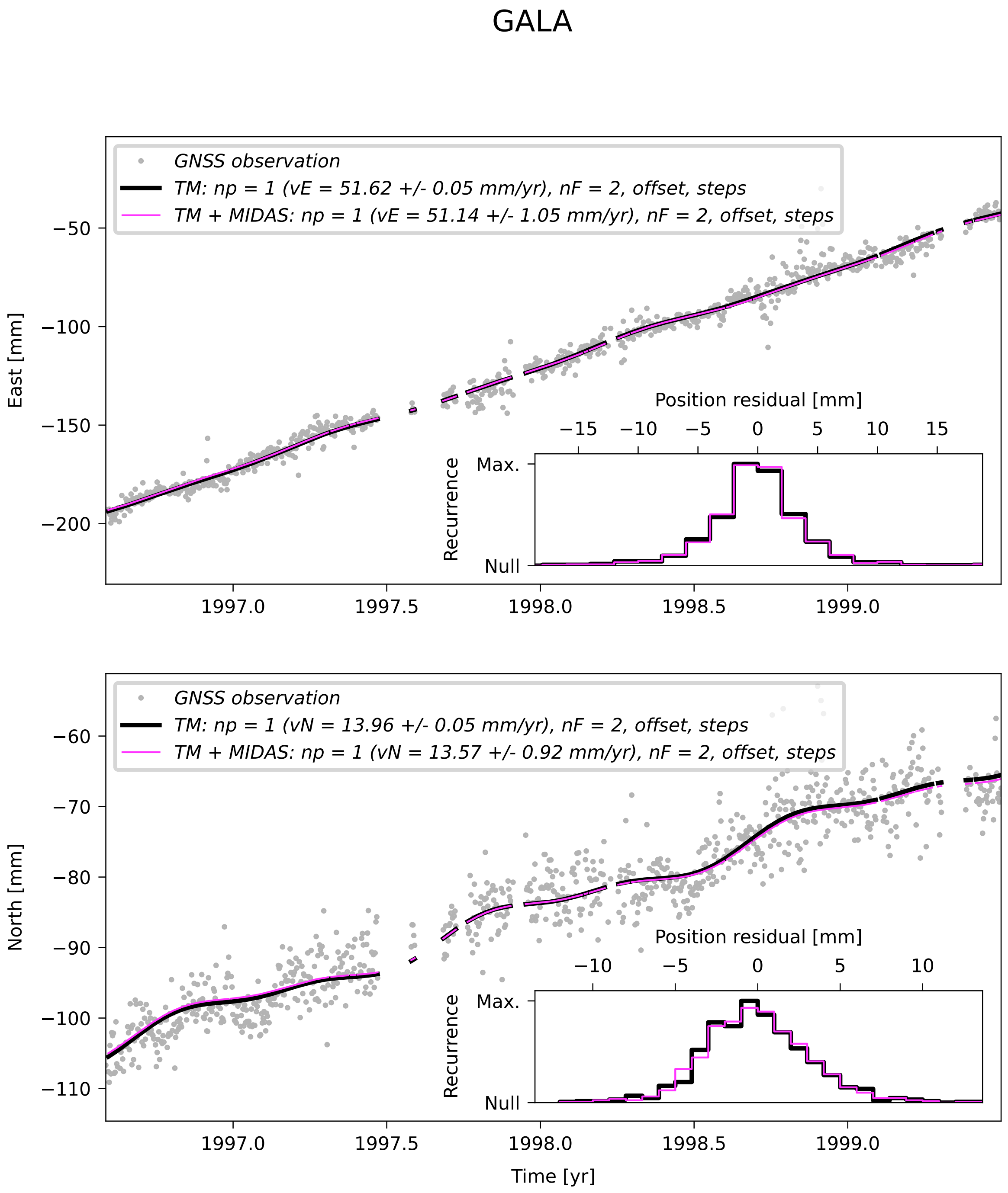

Supplementary Figure 24. Same as Supplementary Figure 18, but showing the time series and TMs of station GALA for the period from July 1996 to June 1999.

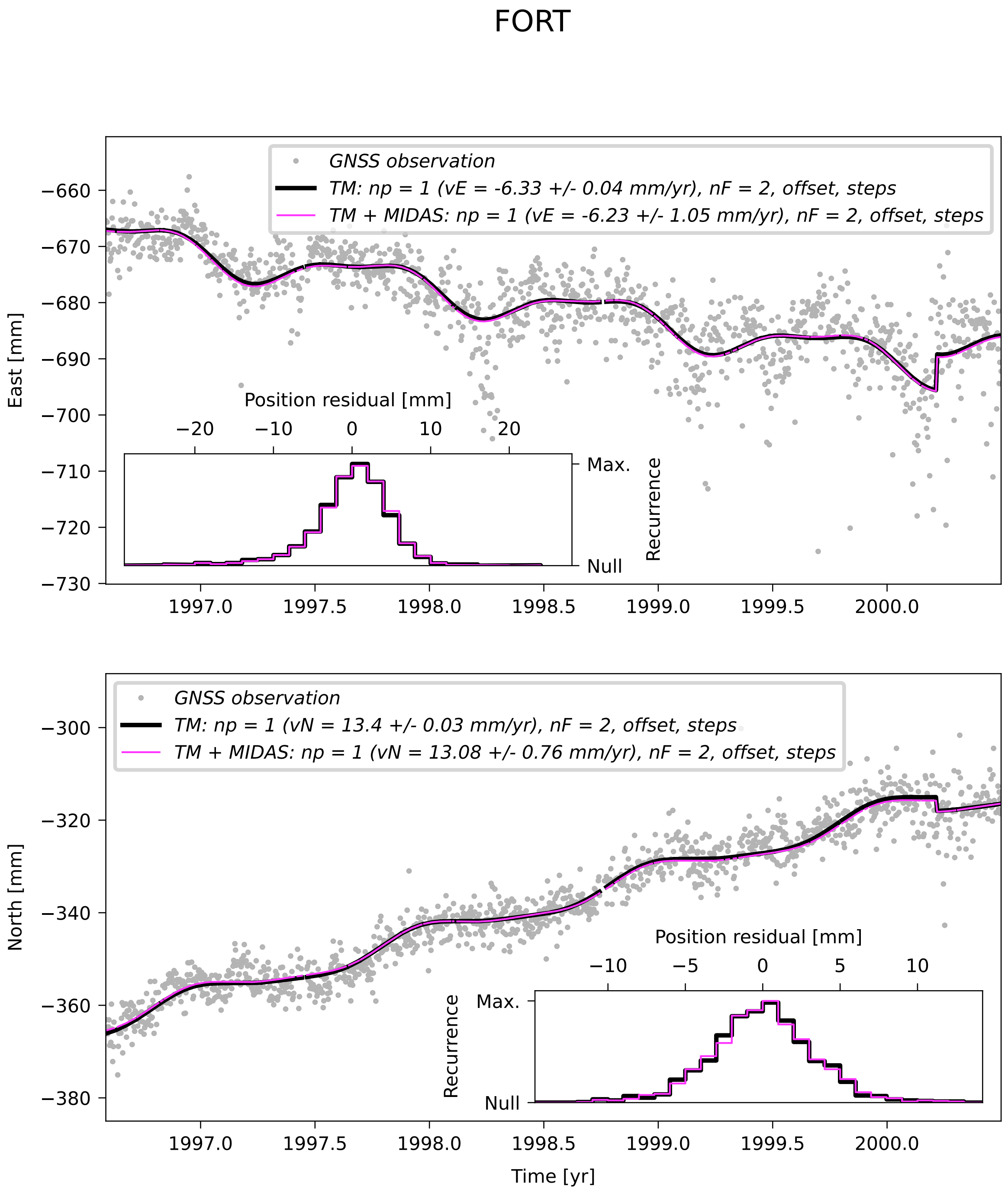

Supplementary Figure 25. Same as Supplementary Figure 18, but showing the time series and TMs of station FORT for the period from July 1996 to June 2000.

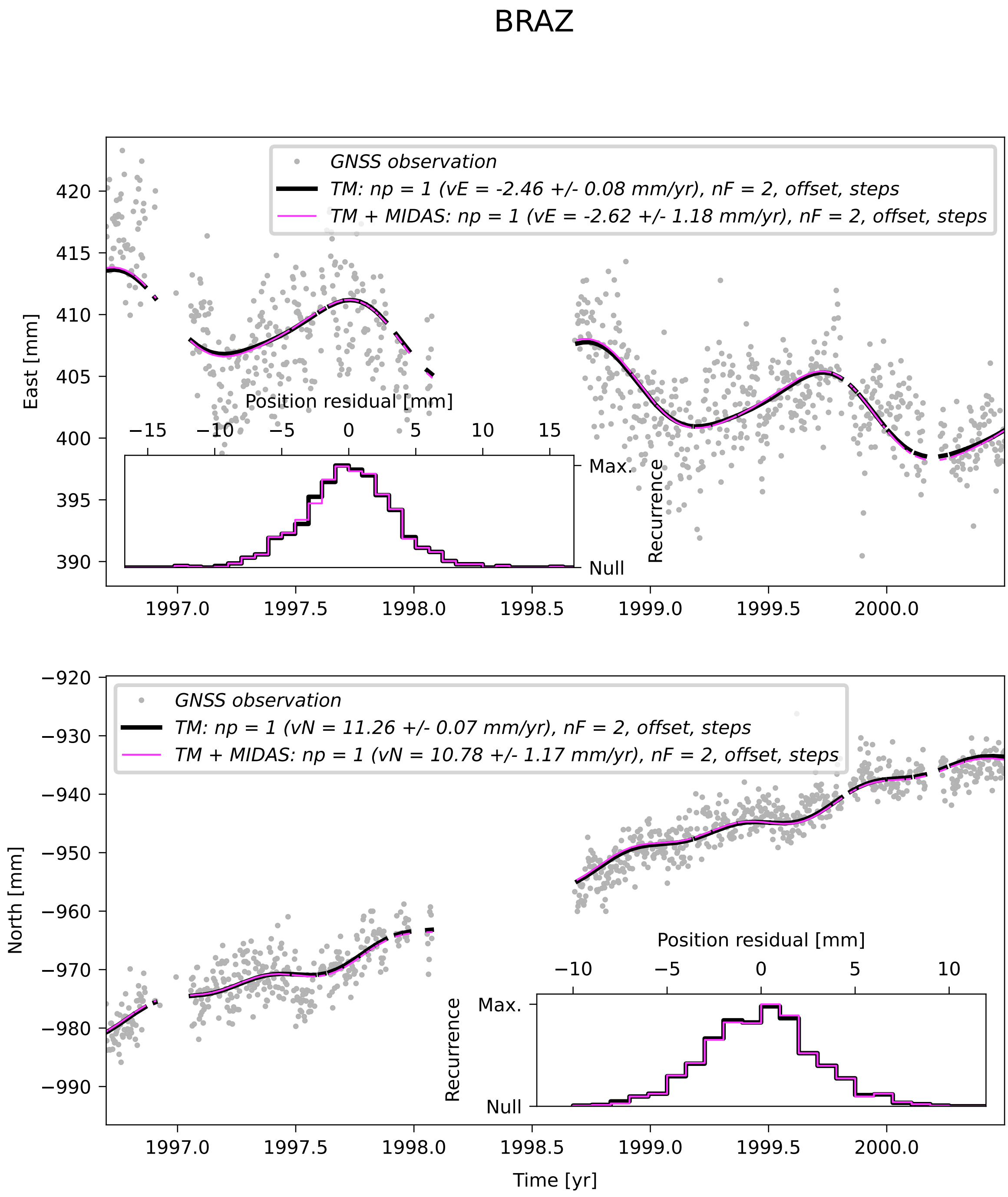

Supplementary Figure 26. Same as Supplementary Figure 18, but showing the time series and TMs of station BRAZ for the period from July 1996 to June 2000.

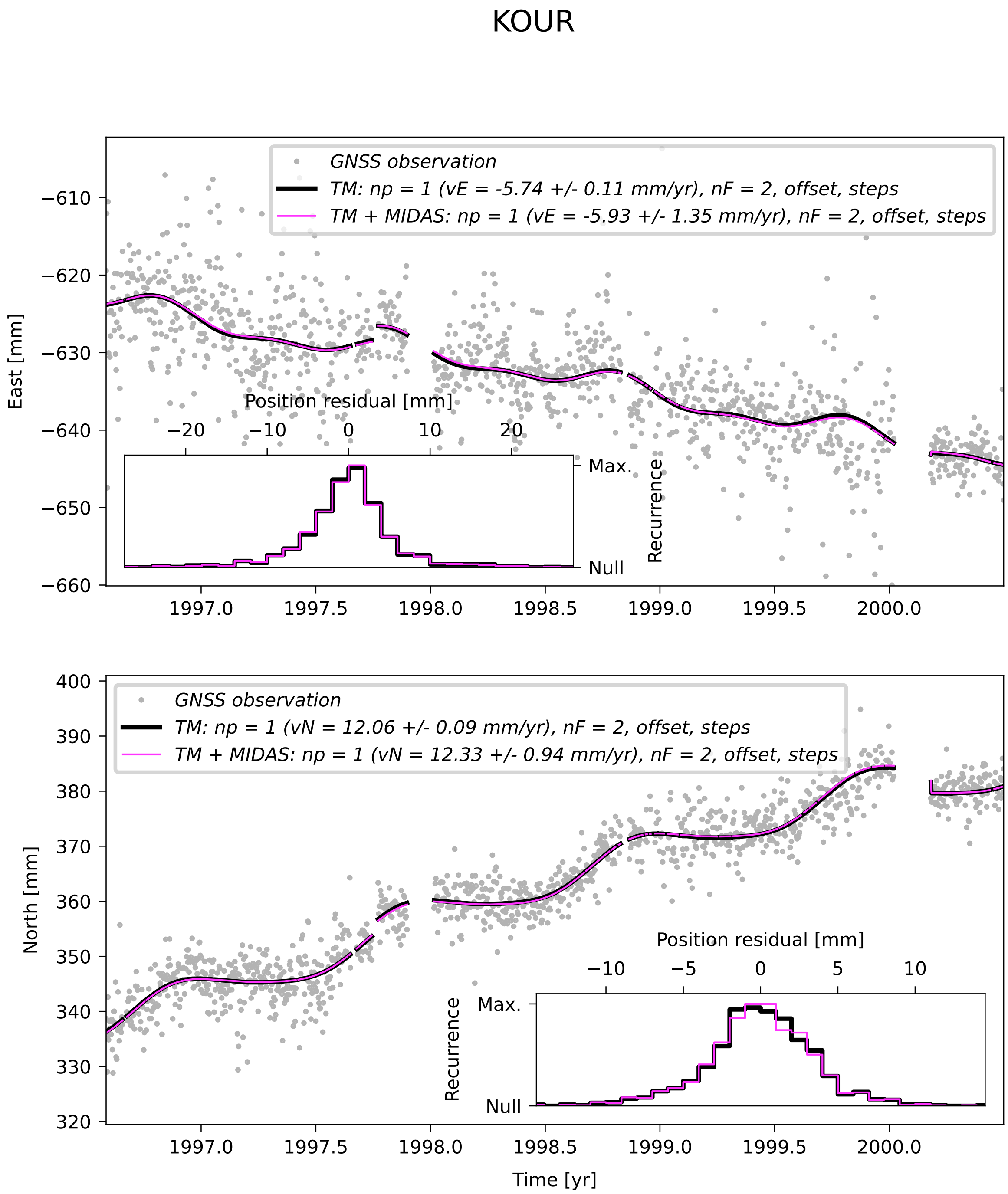

Supplementary Figure 27. Same as Supplementary Figure 18, but showing the time series and TMs of station KOUR for the period from July 1996 to June 2000.

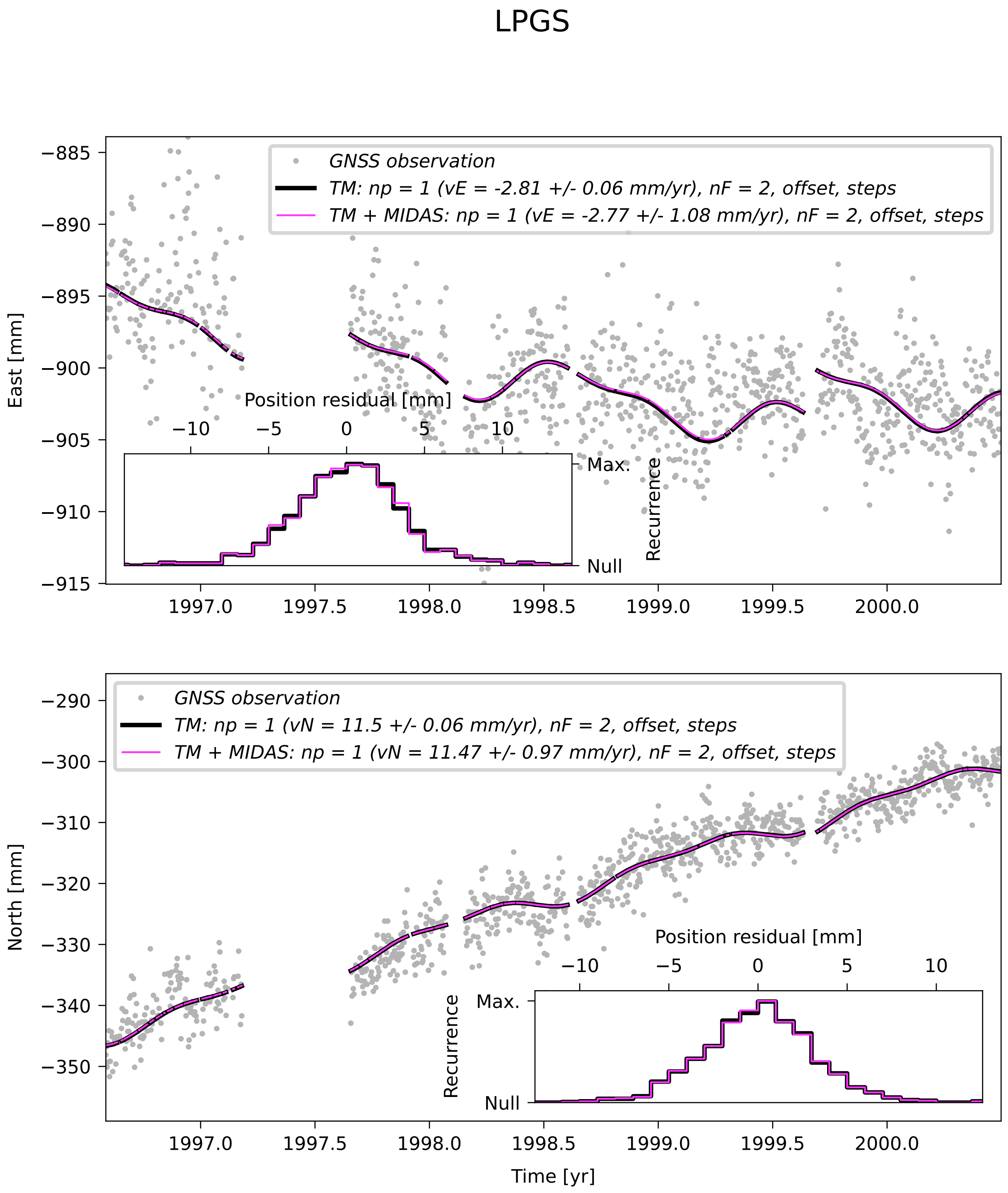

Supplementary Figure 28. Same as Supplementary Figure 18, but showing the time series and TMs of station LPGS for the period from July 1996 to June 2000.

UFPR

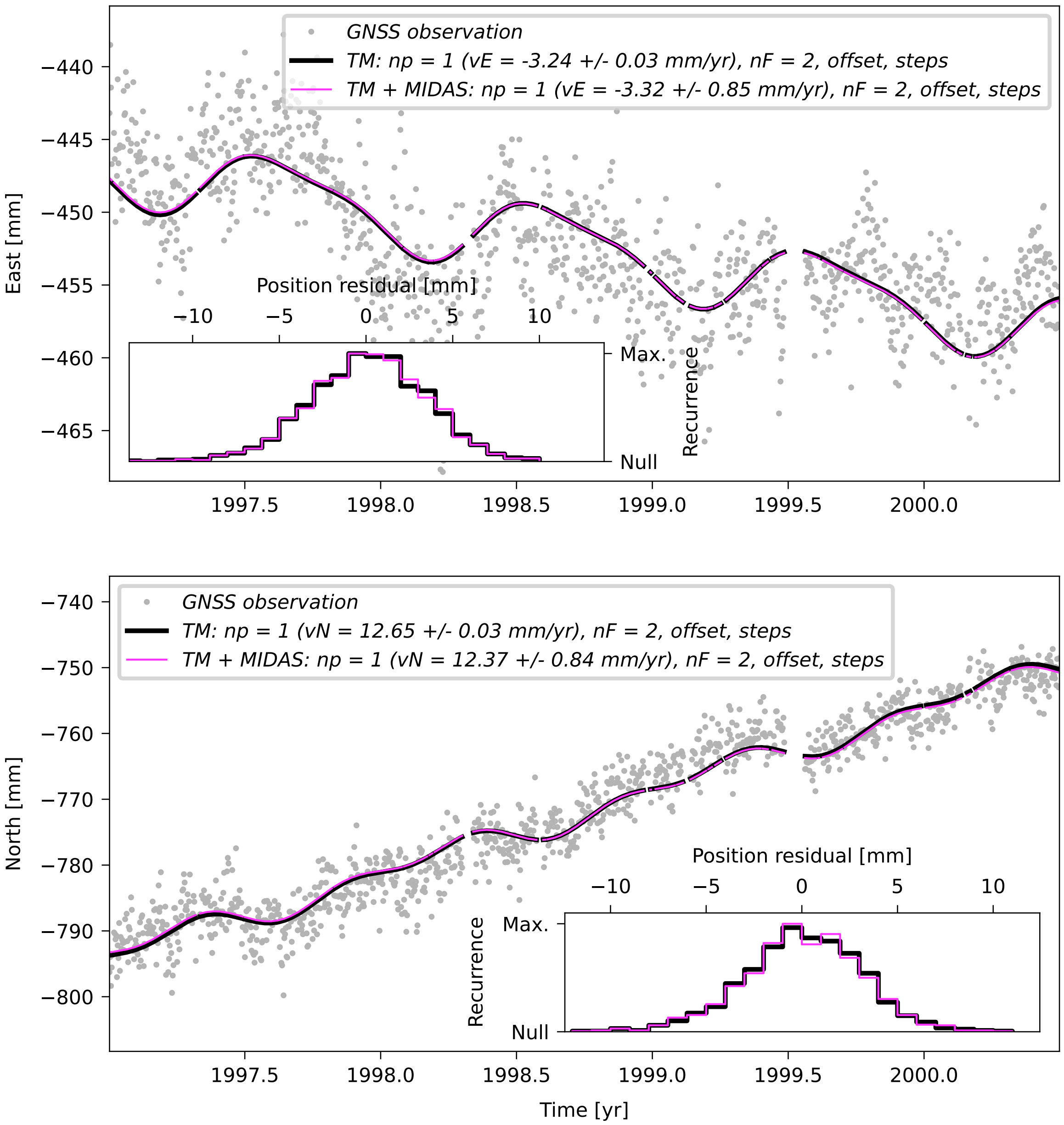

Supplementary Figure 29. Same as Supplementary Figure 18, but showing the time series and TMs of station UFPR for the period from July 1996 to June 2000.

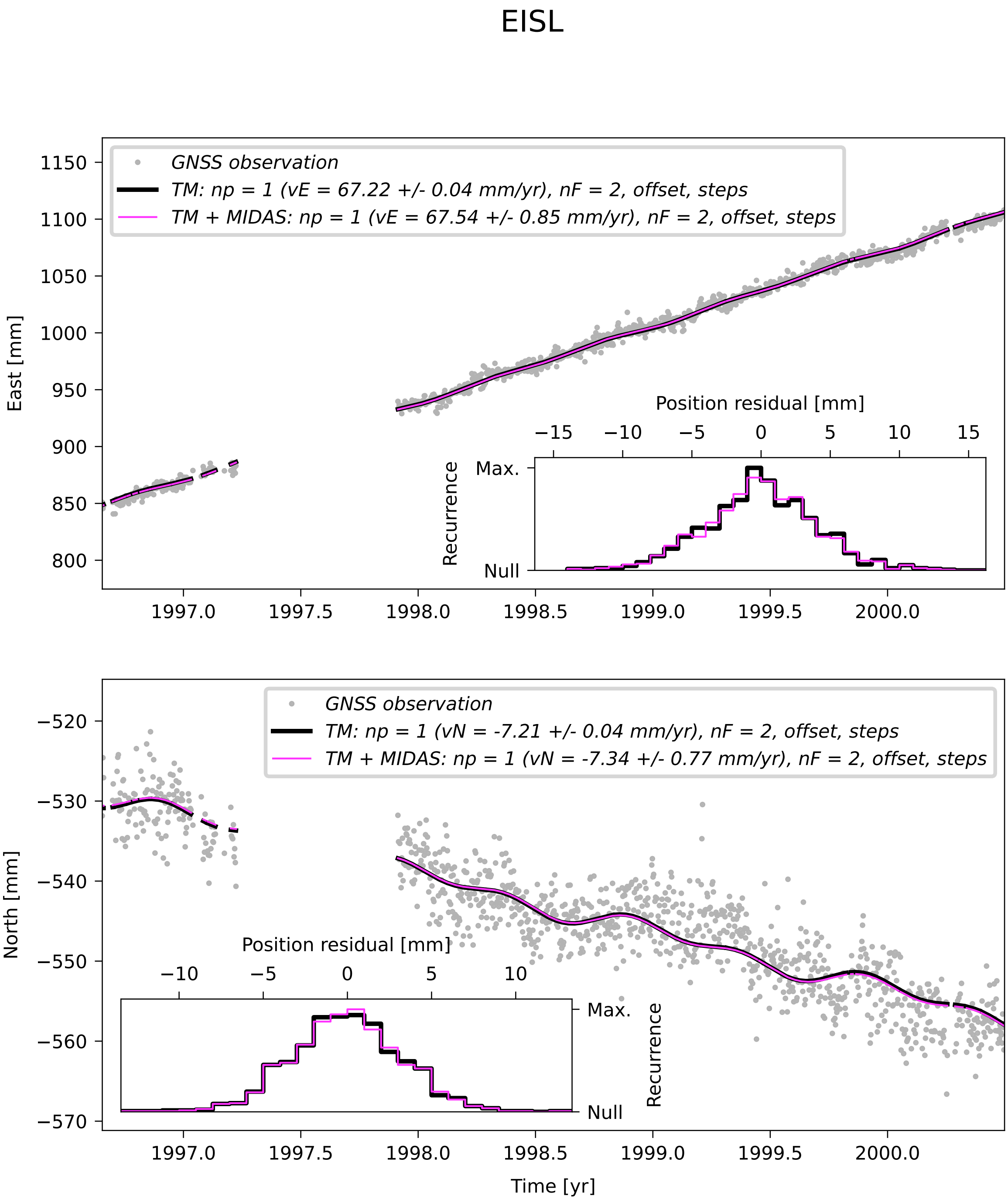

Supplementary Figure 30. Same as Supplementary Figure 18, but showing the time series and TMs of station EISL for the period from July 1996 to June 2000.

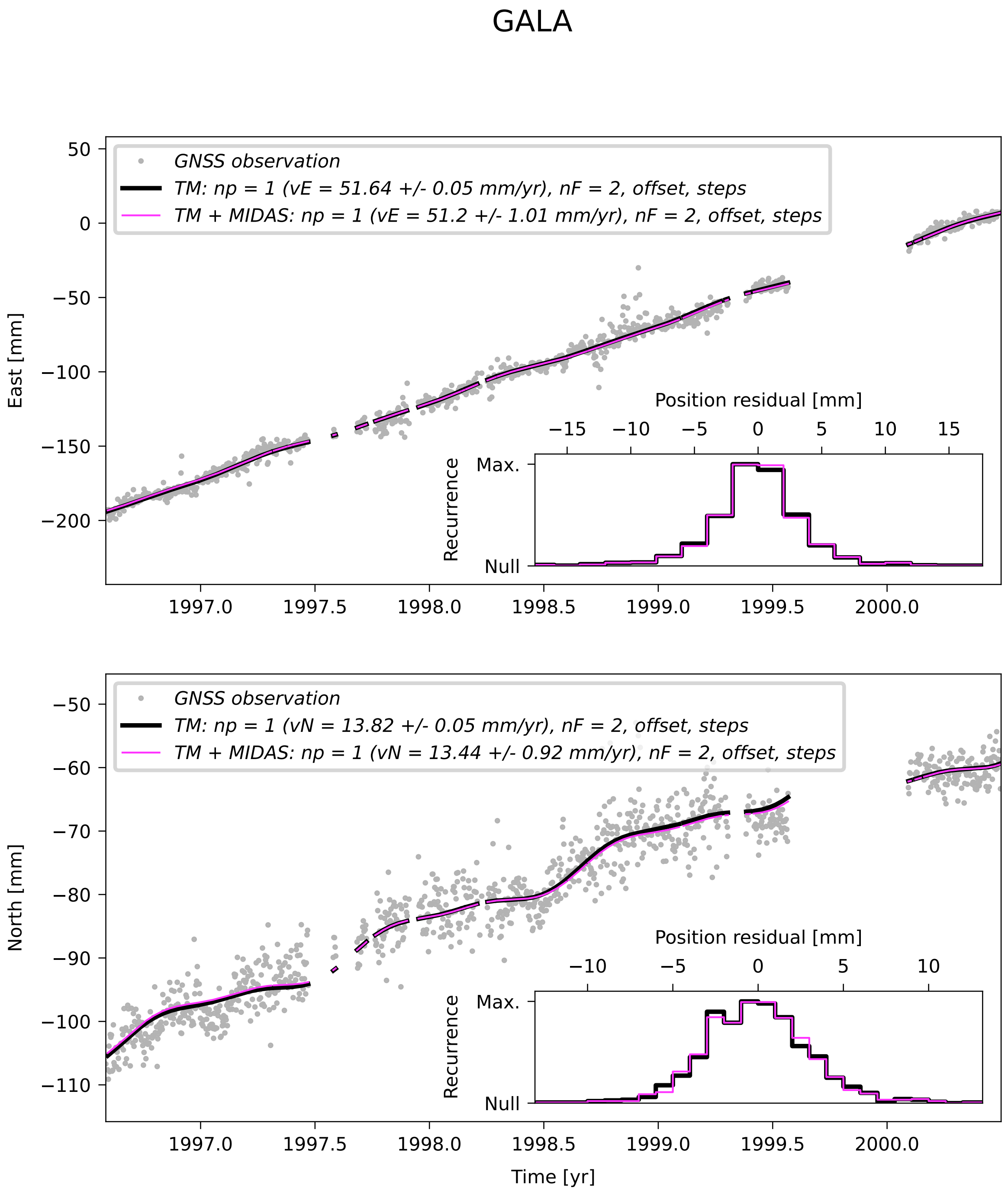

Supplementary Figure 31. Same as Supplementary Figure 18, but showing the time series and TMs of station GALA for the period from July 1996 to June 2000.

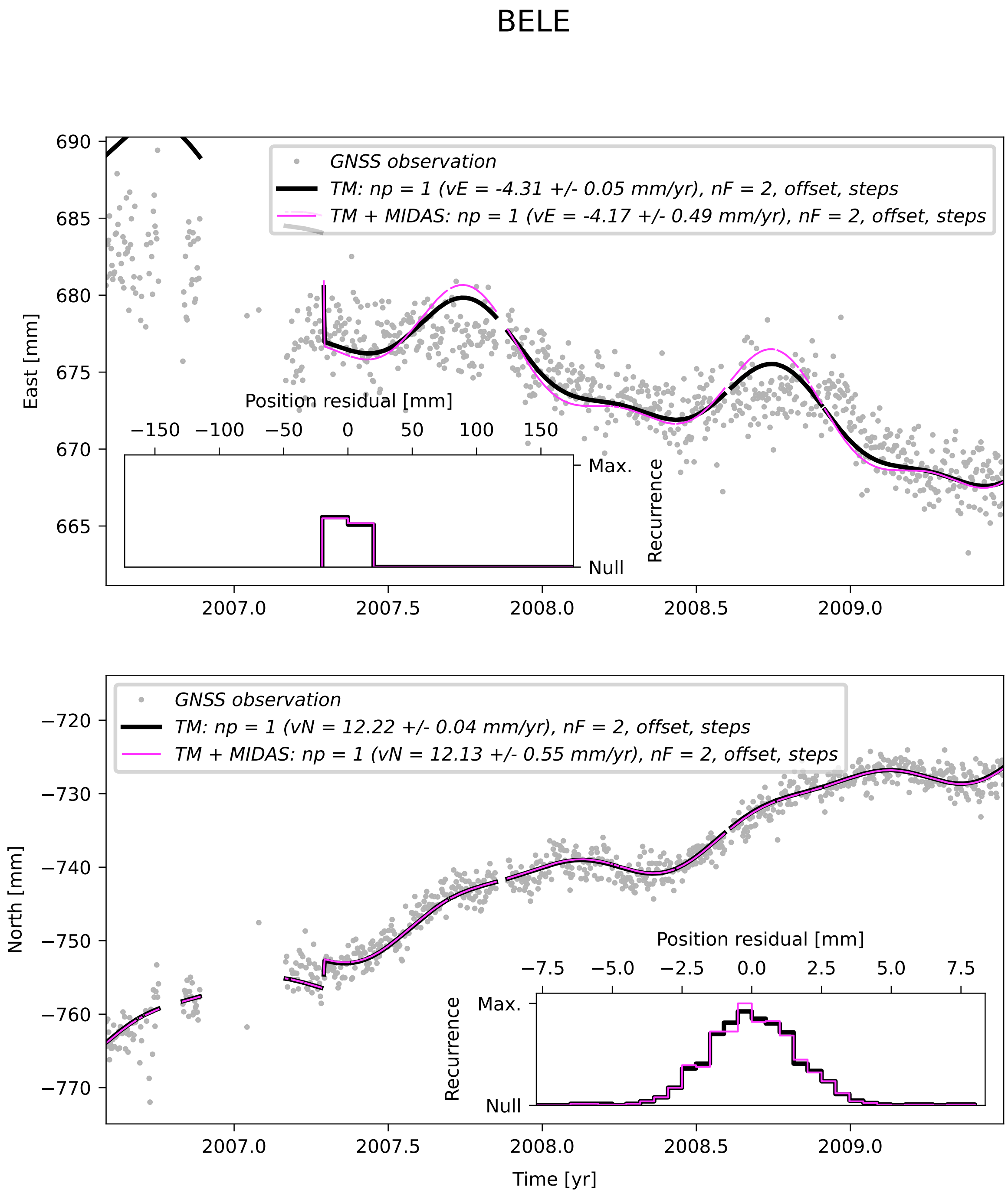

Supplementary Figure 32. Same as Supplementary Figure 18, but showing the time series and TMs of station BELE for the period from July 2006 to June 2009.

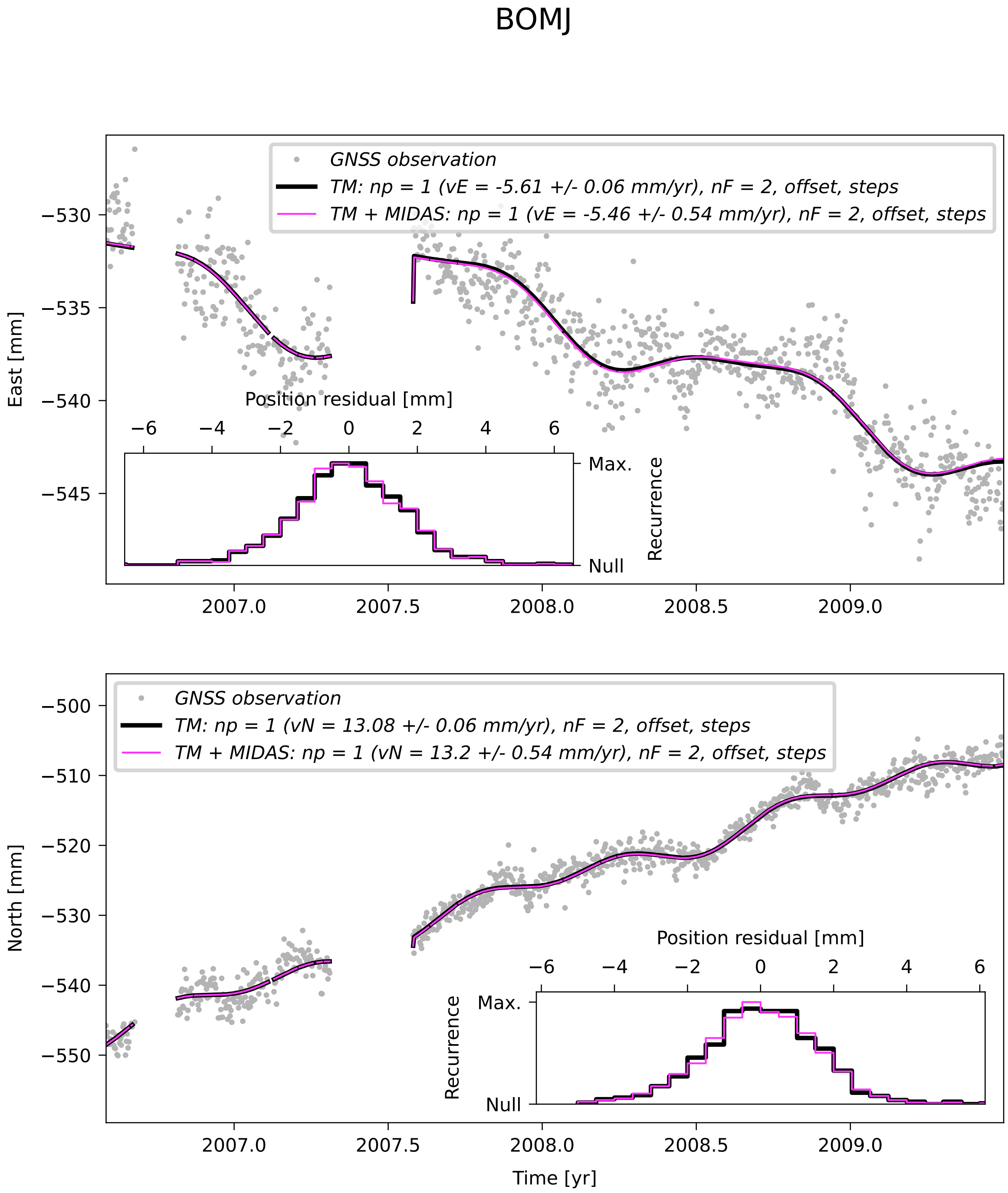

Supplementary Figure 33. Same as Supplementary Figure 18, but showing the time series and TMs of station BOMJ for the period from July 2006 to June 2009. Please note that this site has not been used to constrain the Euler vector for this time period. Instead, it has been excluded due to the *a posteriori* analysis of the velocity residuals (see Supp. Figure 13).

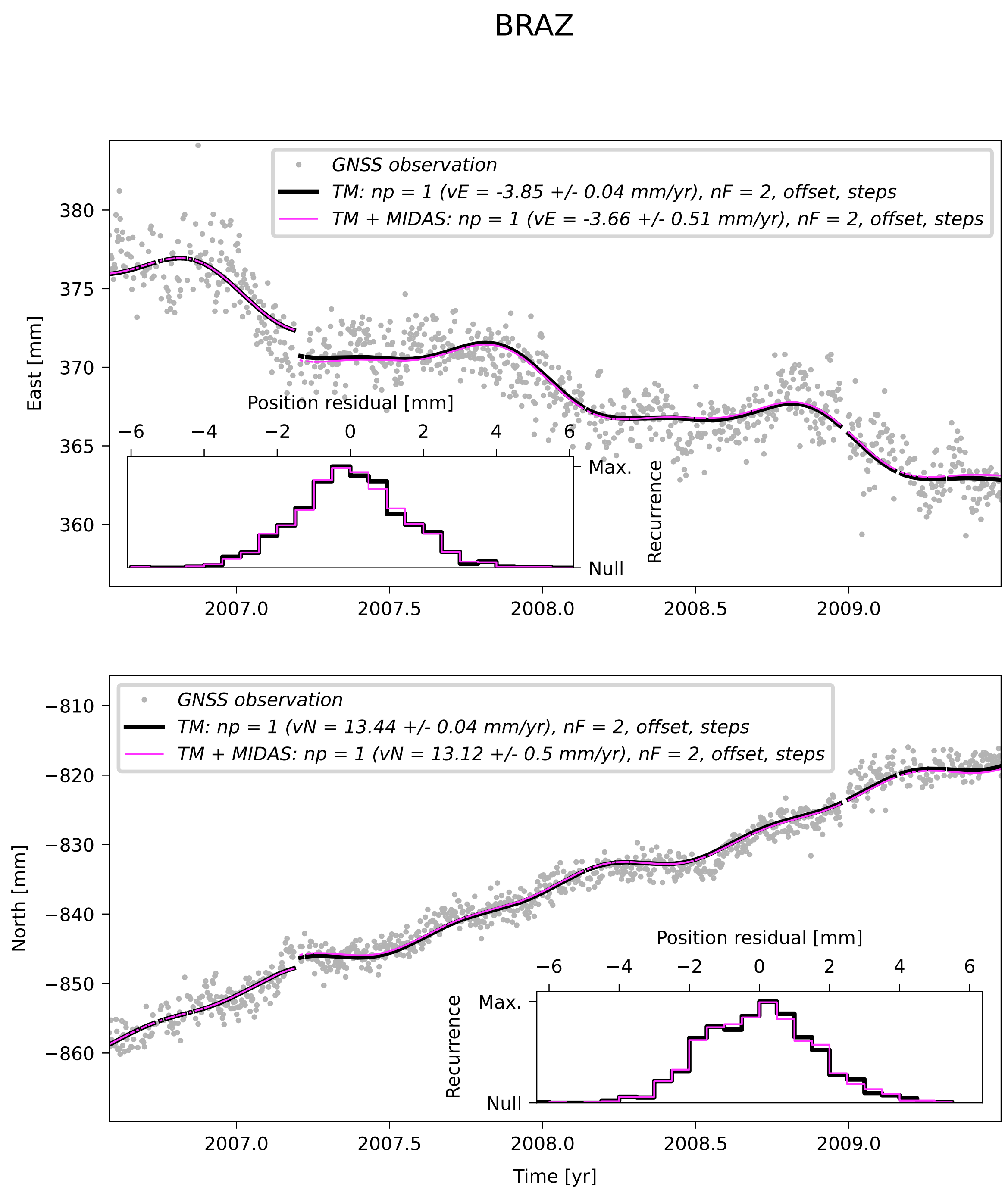

Supplementary Figure 34. Same as Supplementary Figure 18, but showing the time series and TMs of station BRAZ for the period from July 2006 to June 2009.

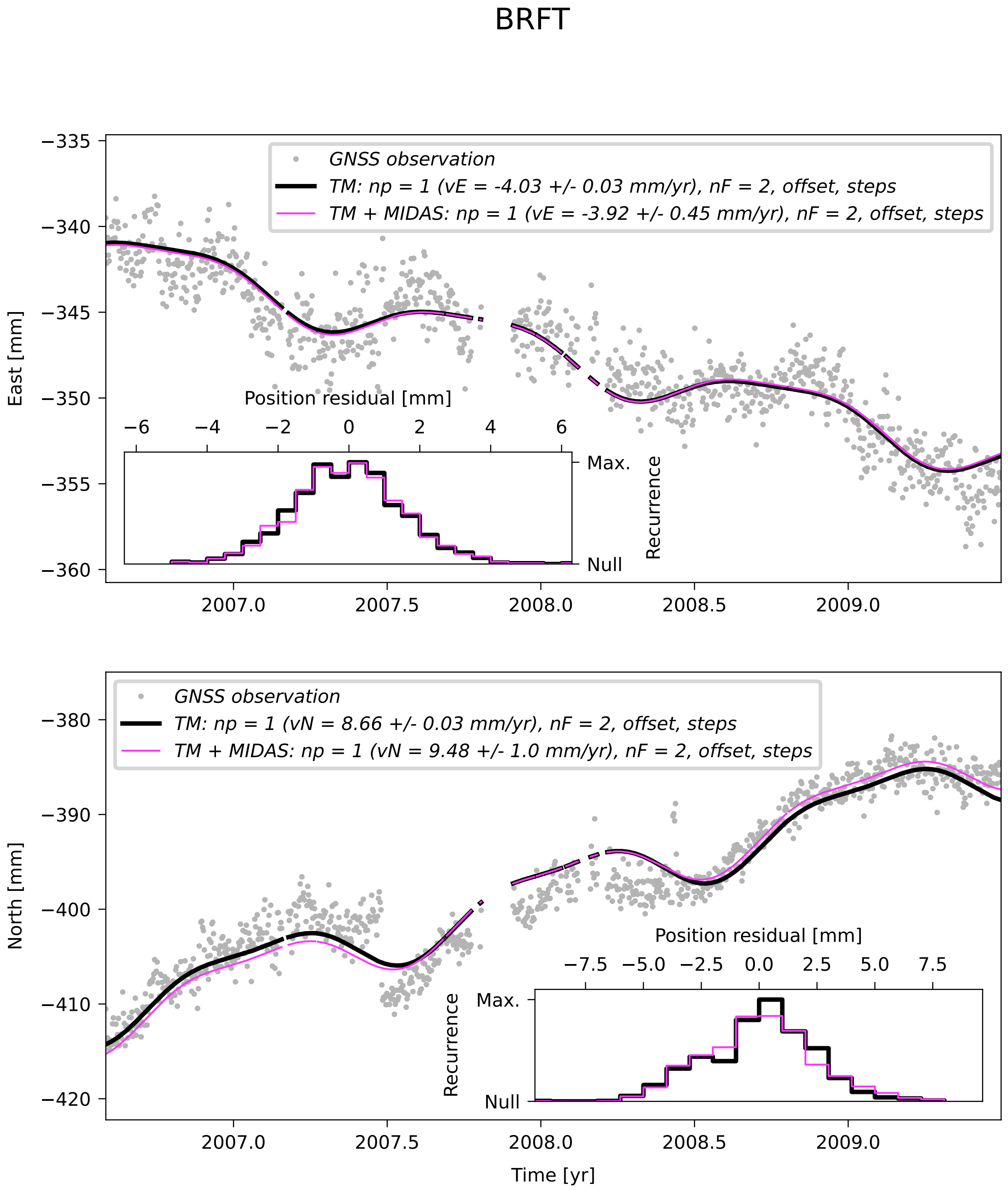

Supplementary Figure 35. Same as Supplementary Figure 18, but showing the time series and TMs of station BRFT for the period from July 2006 to June 2009. Please note that this site has not been used to constrain the Euler vector for this time period. Instead, it has been excluded due to the *a posteriori* analysis of the velocity residuals (see Supp. Figure 13).

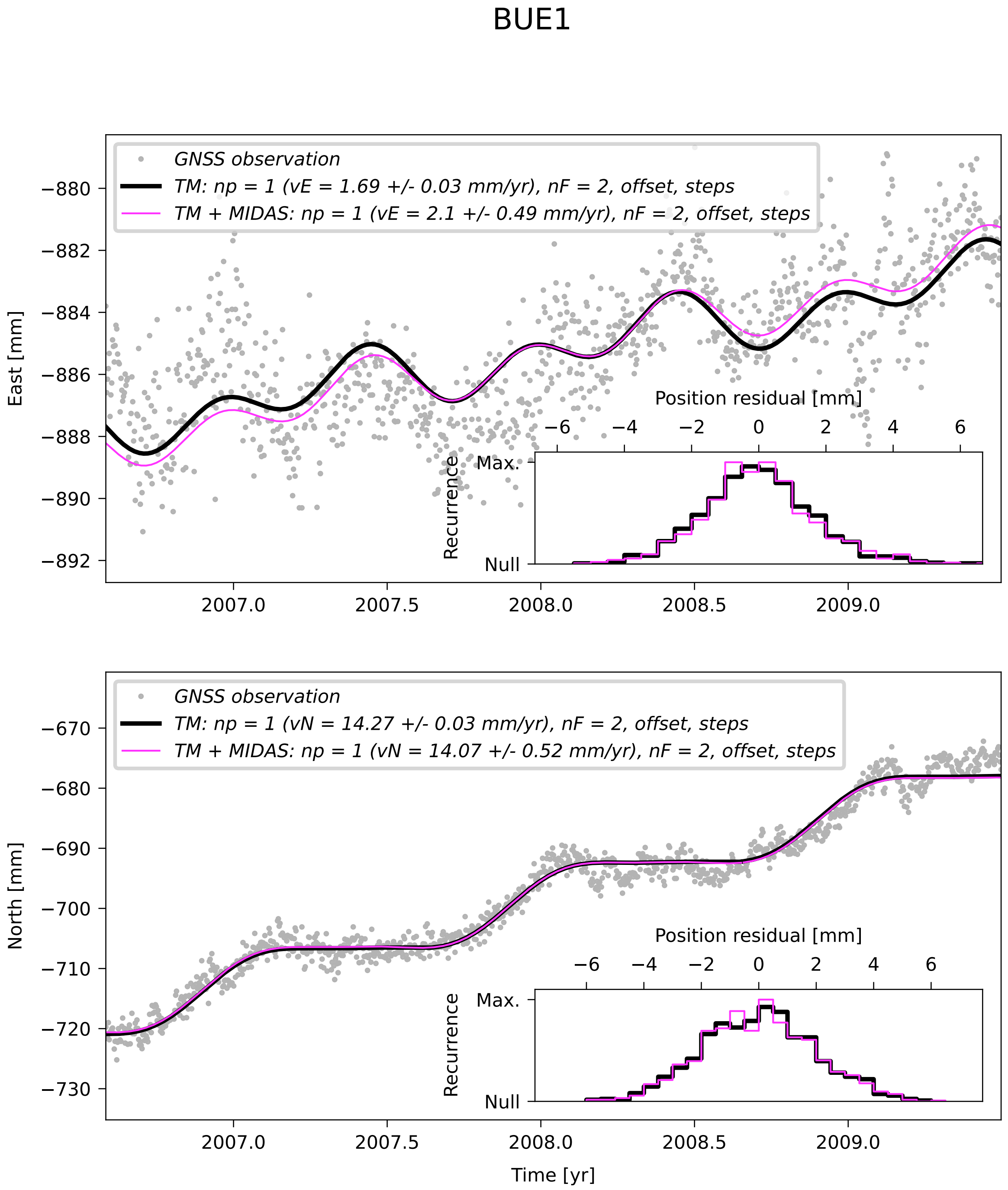

Supplementary Figure 36. Same as Supplementary Figure 18, but showing the time series and TMs of station BUE1 for the period from July 2006 to June 2009. Please note that this site has not been used to constrain the Euler vector for this time period. Instead, it has been excluded due to the *a posteriori* analysis of the velocity residuals (see Supp. Figure 13).

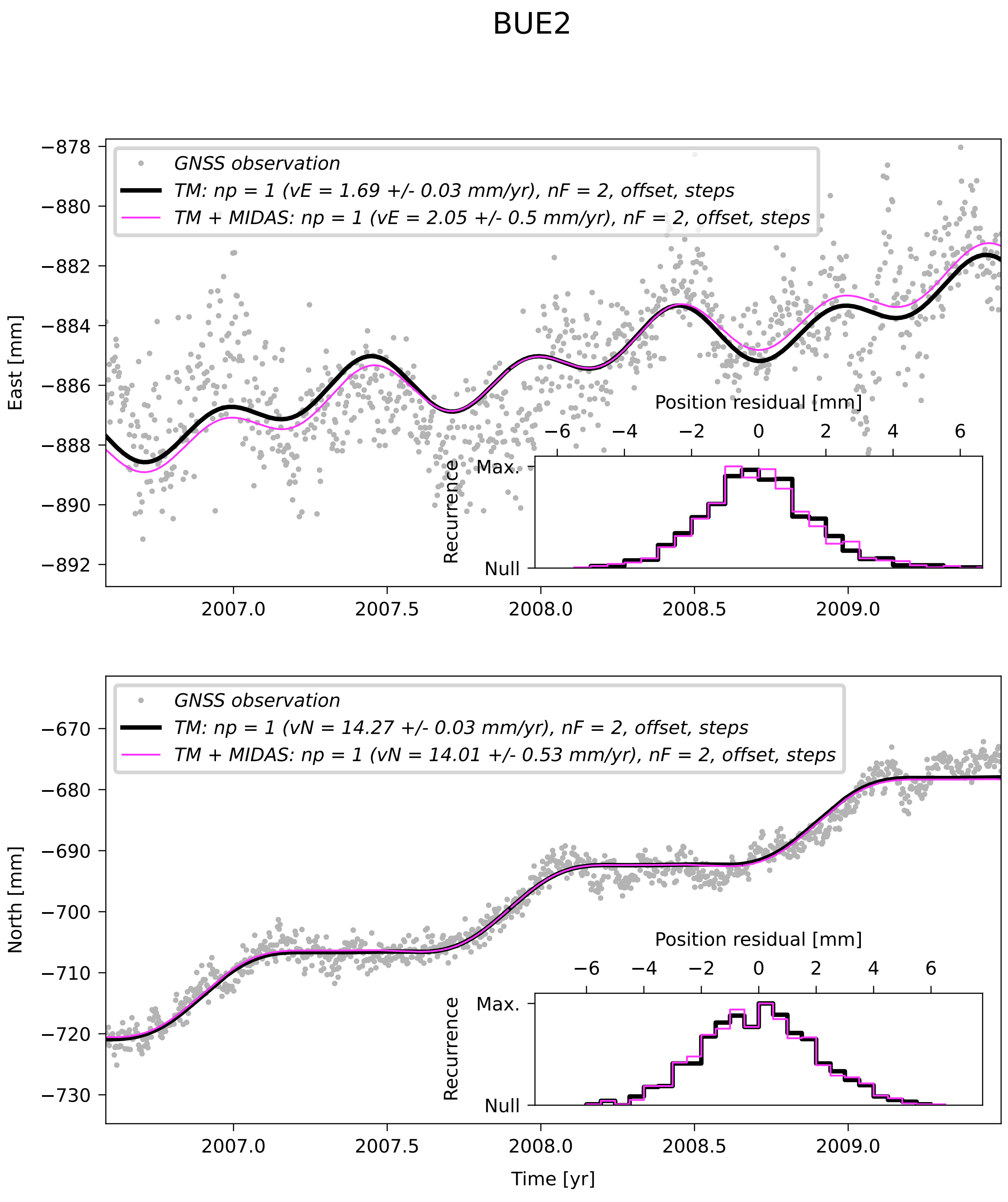

Supplementary Figure 37. Same as Supplementary Figure 18, but showing the time series and TMs of station BUE2 for the period from July 2006 to June 2009. Please note that this site has not been used to constrain the Euler vector for this time period. Instead, it has been excluded due to the *a posteriori* analysis of the velocity residuals (see Supp. Figure 13).

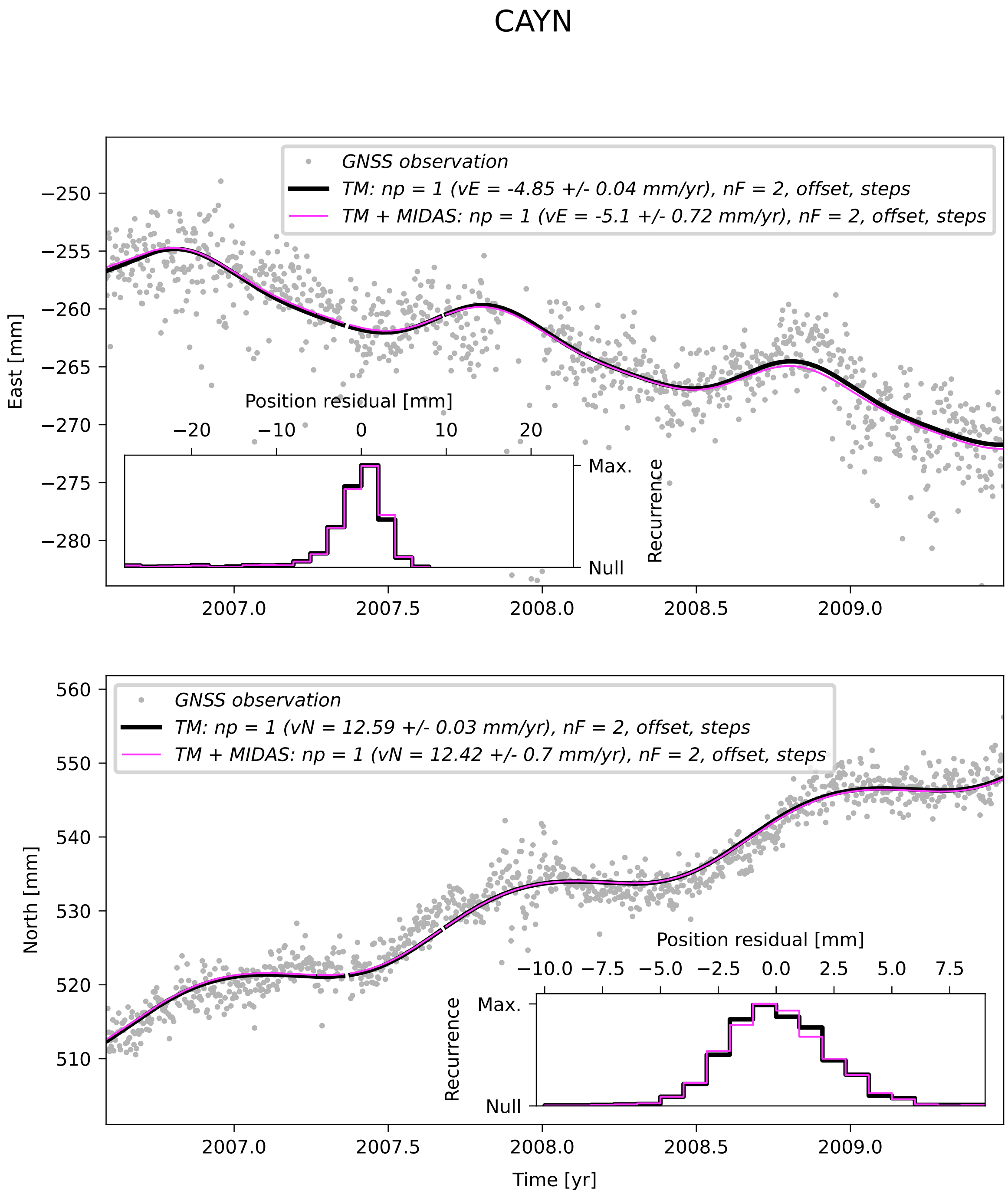

Supplementary Figure 38. Same as Supplementary Figure 18, but showing the time series and TMs of station CAYN for the period from July 2006 to June 2009.

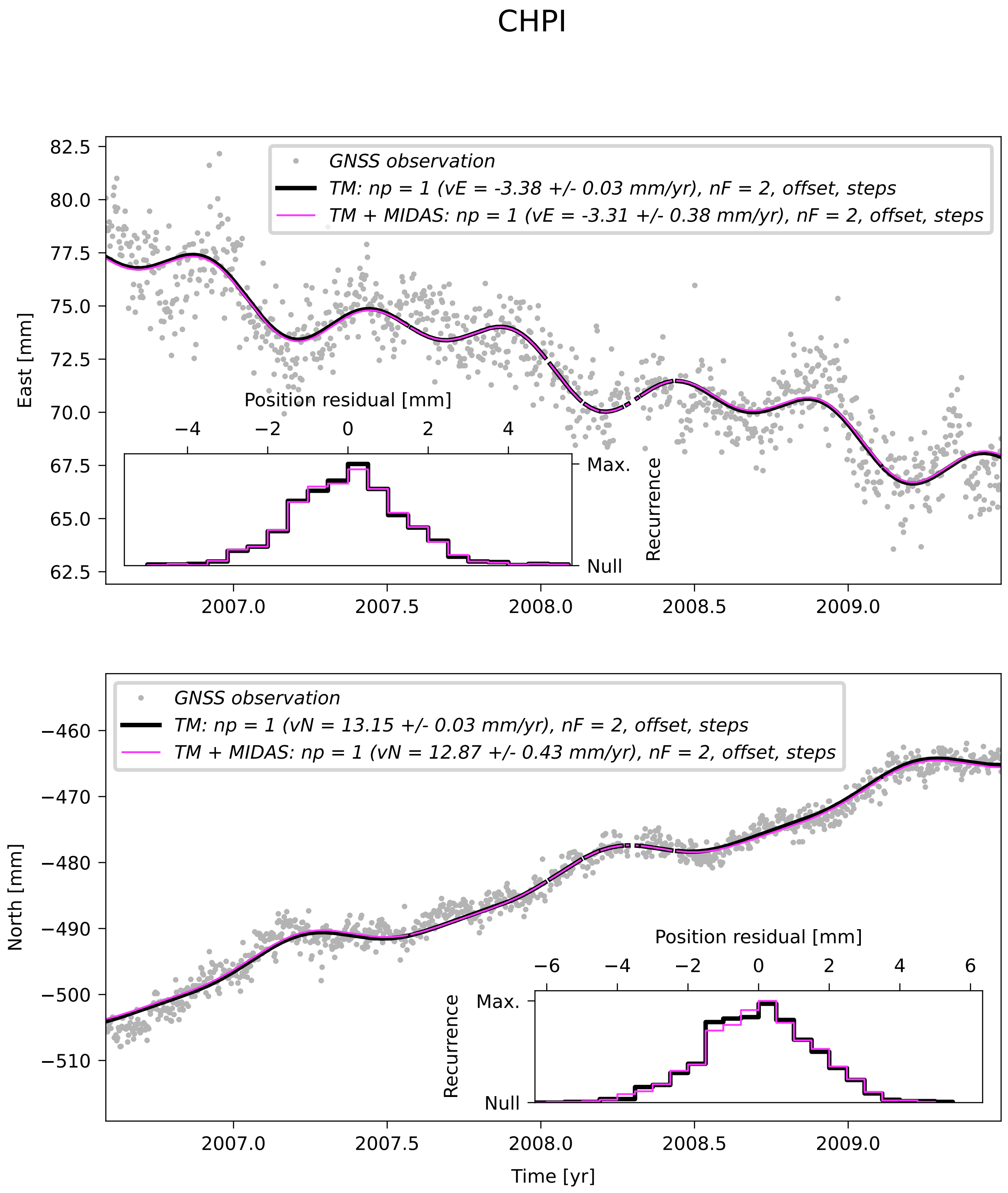

Supplementary Figure 39. Same as Supplementary Figure 18, but showing the time series and TMs of station CHPI for the period from July 2006 to June 2009.

CRA1

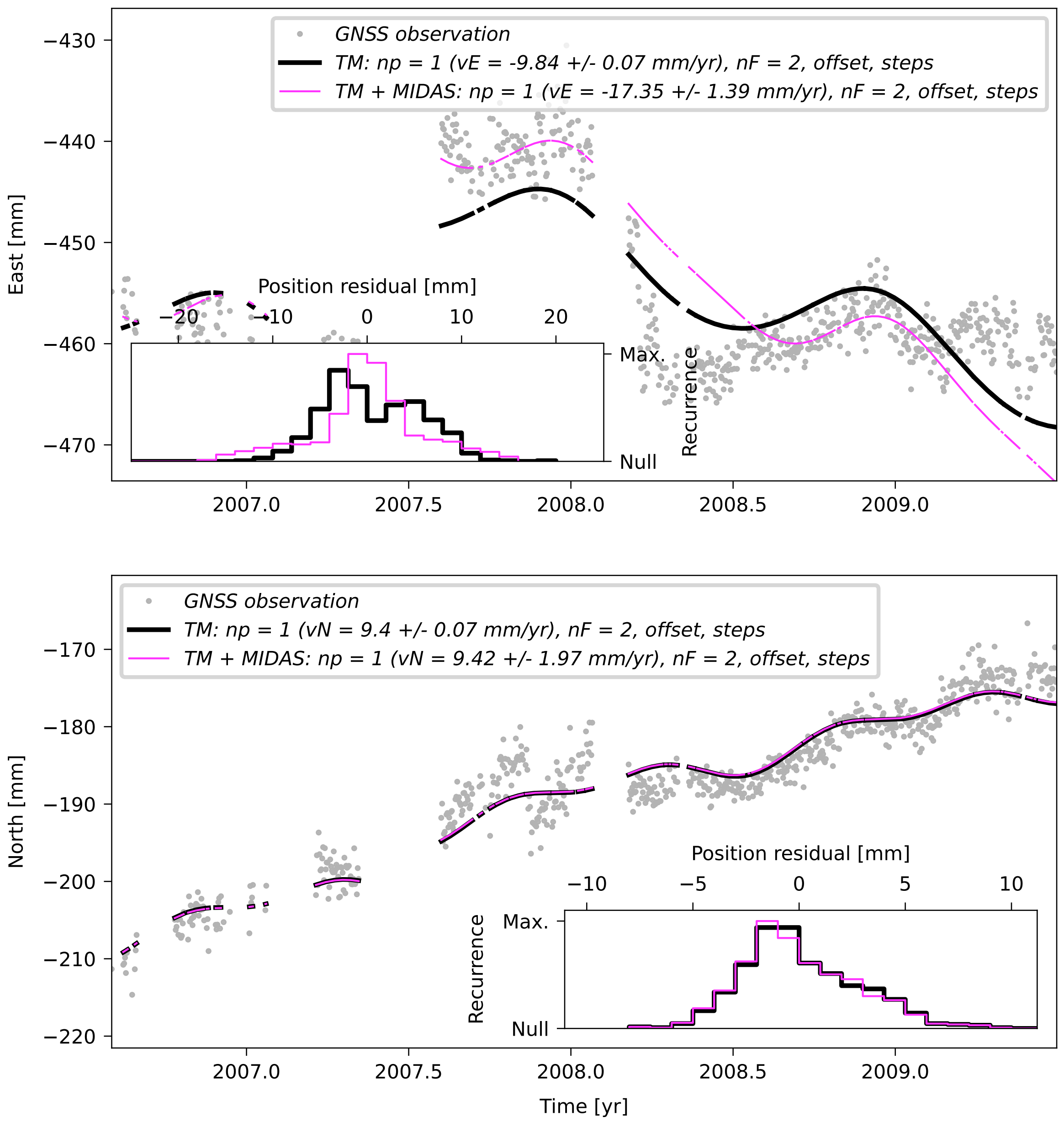

Supplementary Figure 40. Same as Supplementary Figure 18, but showing the time series and TMs of station CRA1 for the period from July 2006 to June 2009. Please note that this site has not been used to constrain the Euler vector for this time period. Instead, it has been excluded due to the *a posteriori* analysis of the velocity residuals (see Supp. Figure 13).

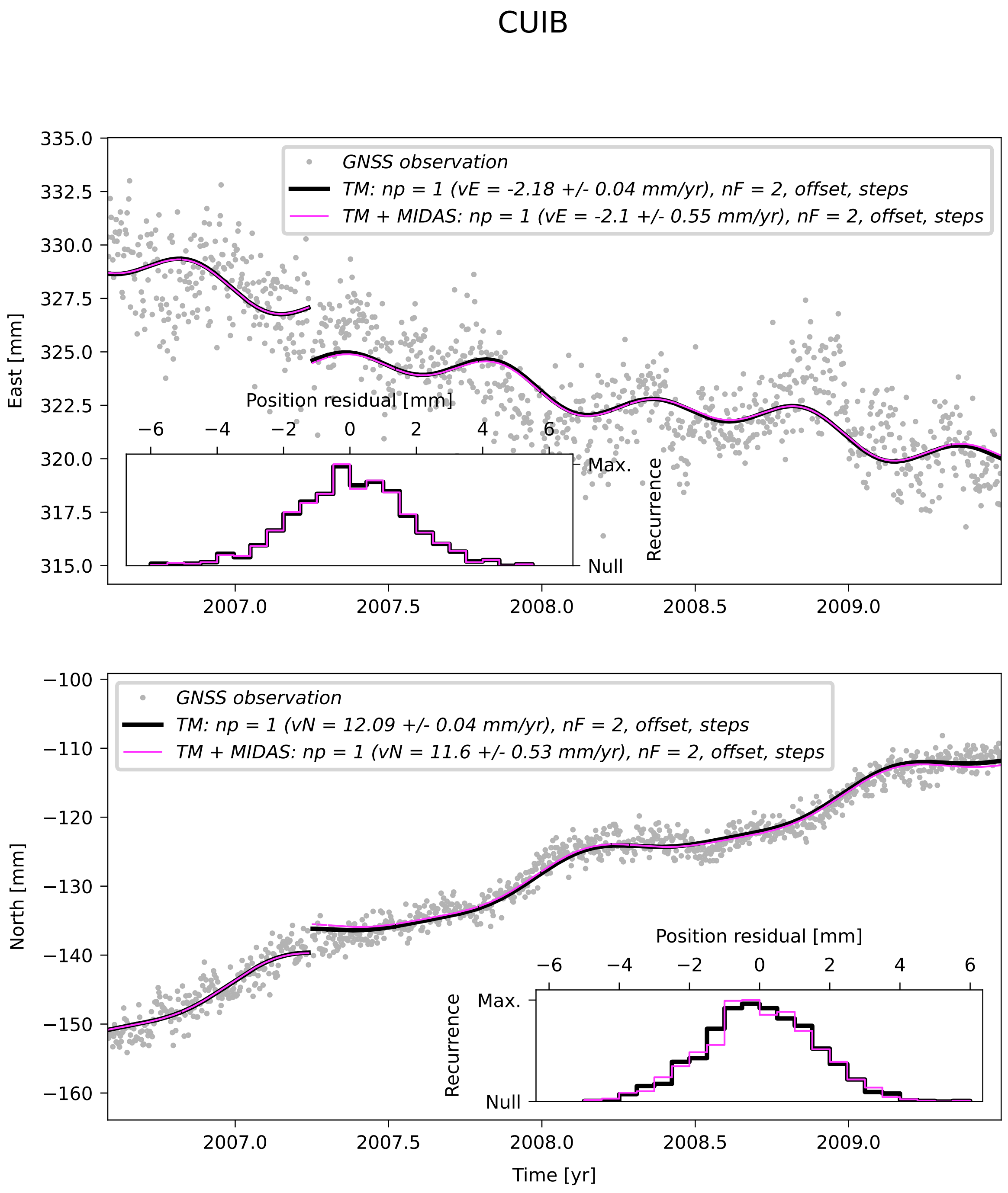

Supplementary Figure 41. Same as Supplementary Figure 18, but showing the time series and TMs of station CUIB for the period from July 2006 to June 2009.

GVA1

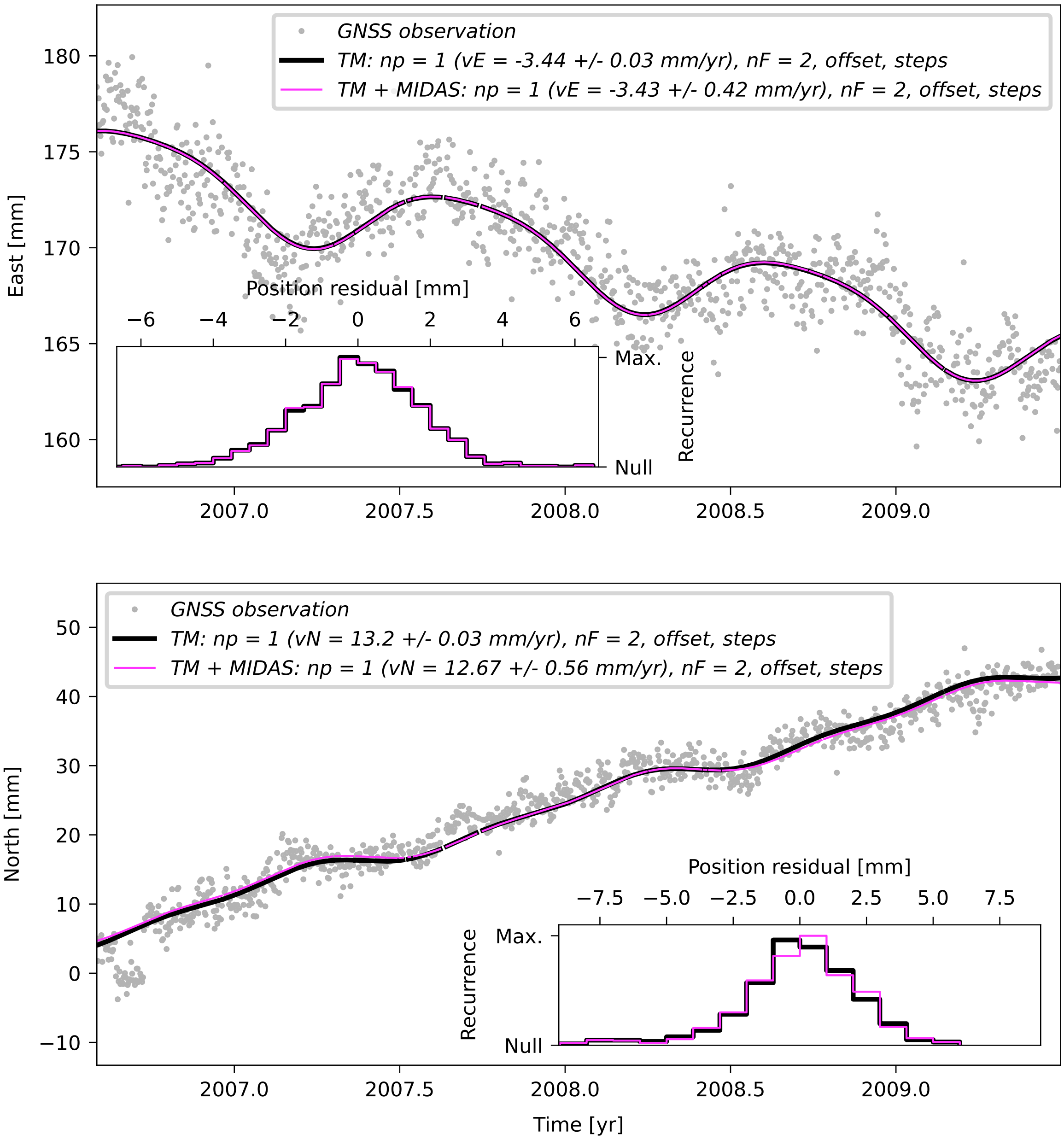

Supplementary Figure 42. Same as Supplementary Figure 18, but showing the time series and TMs of station GVA1 for the period from July 2006 to June 2009.

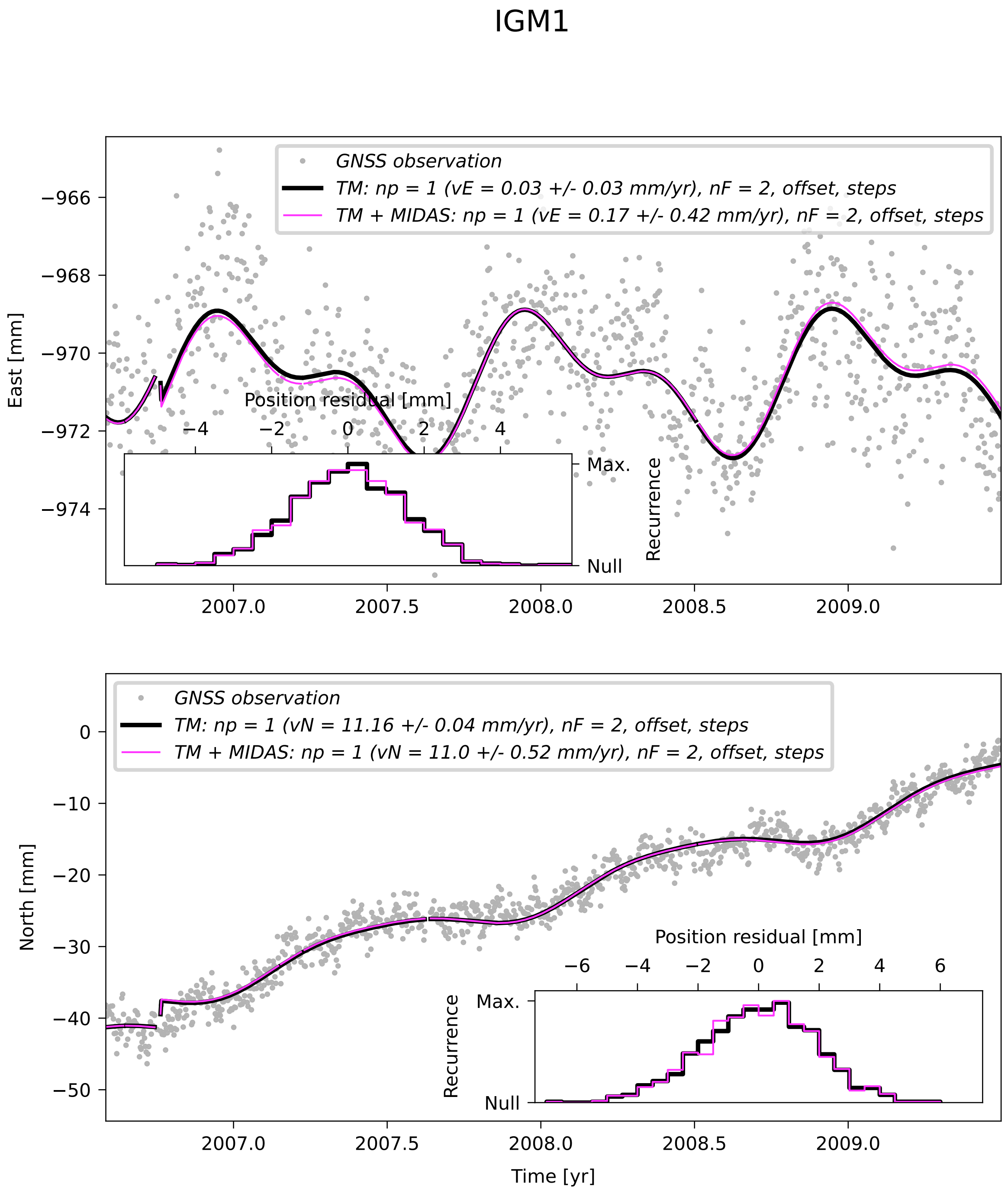

Supplementary Figure 43. Same as Supplementary Figure 18, but showing the time series and TMs of station IGM1 for the period from July 2006 to June 2009.

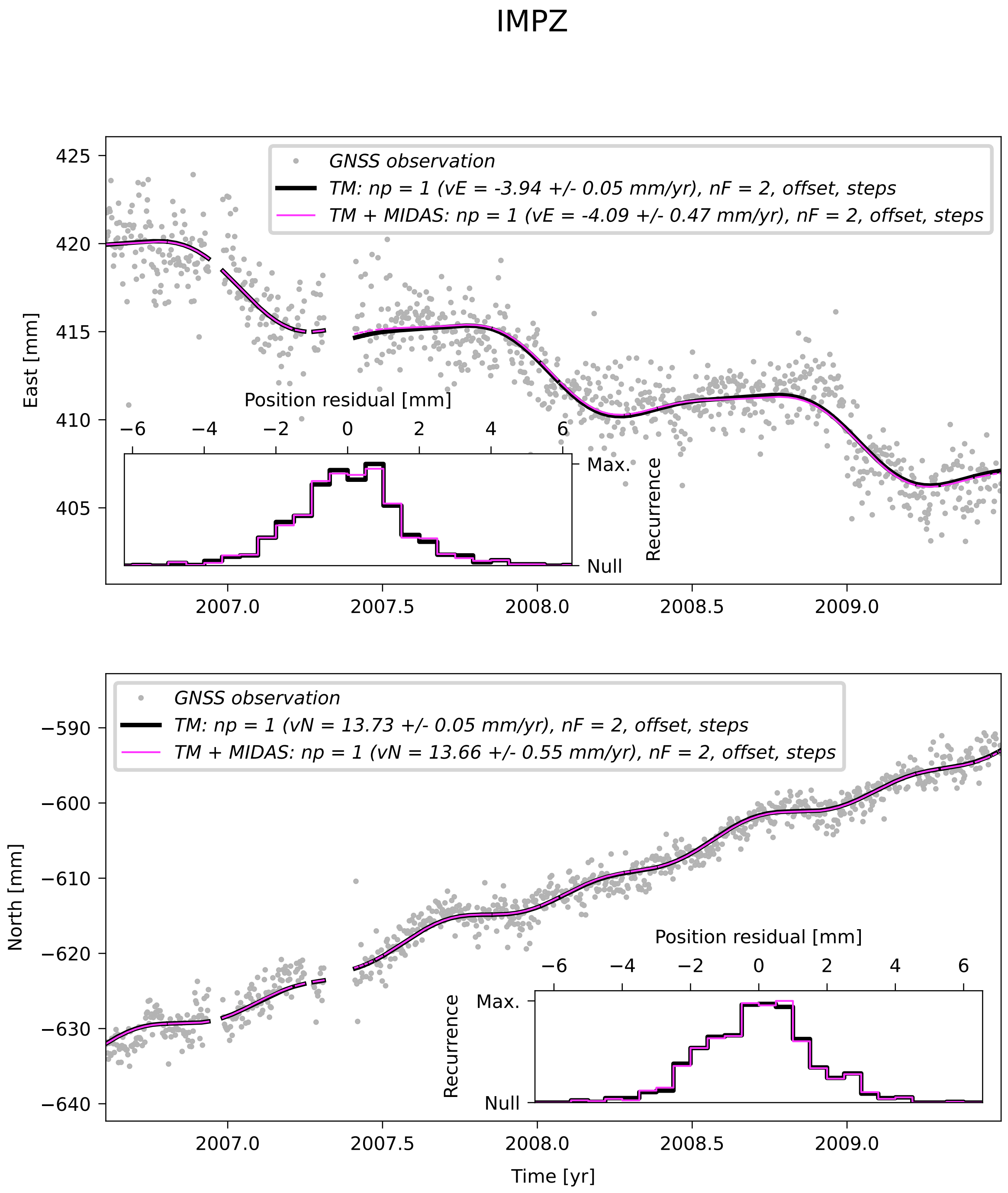

Supplementary Figure 44. Same as Supplementary Figure 18, but showing the time series and TMs of station IMPZ for the period from July 2006 to June 2009.

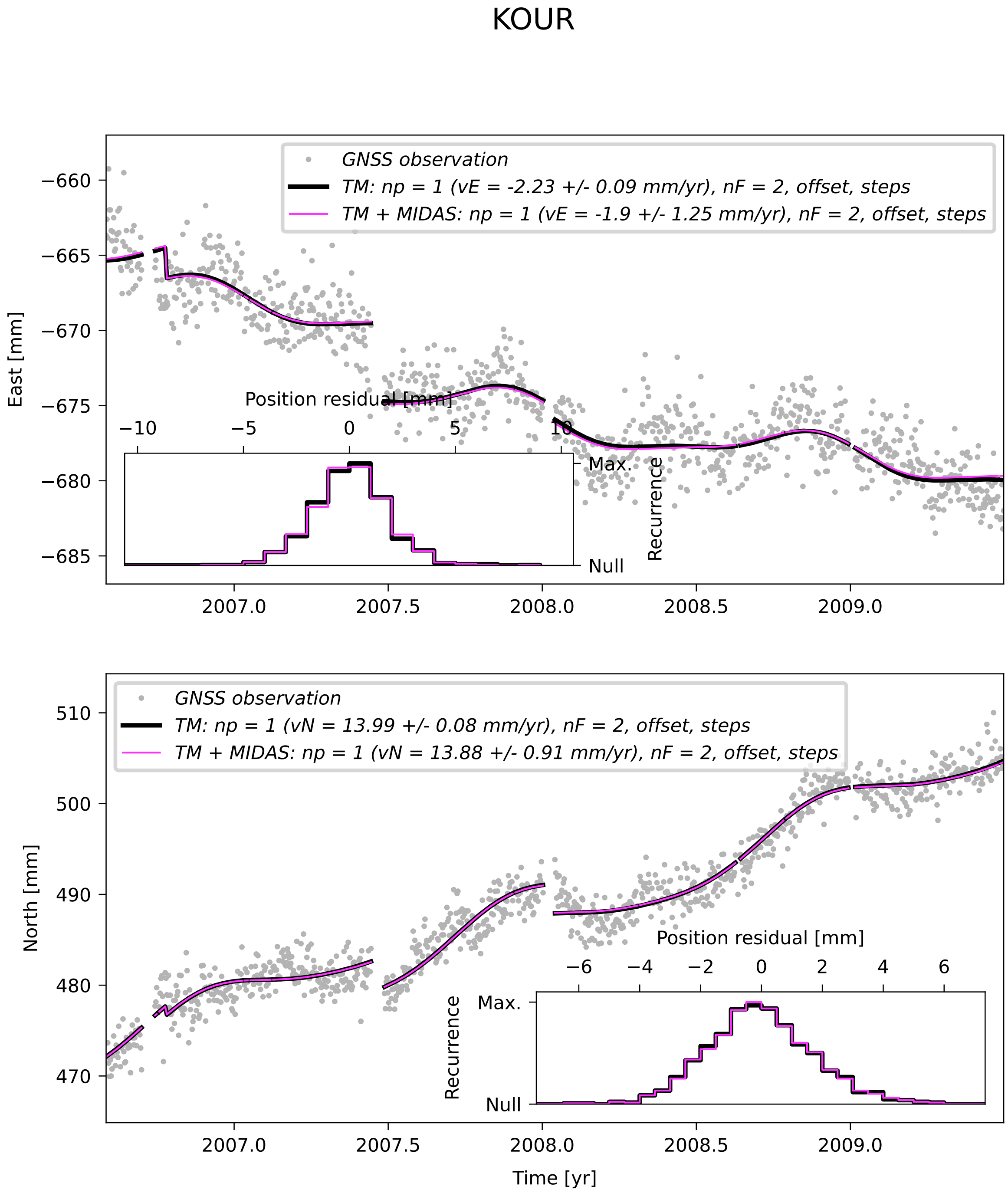

Supplementary Figure 45. Same as Supplementary Figure 18, but showing the time series and TMs of station KOUR for the period from July 2006 to June 2009. Please note that this site has not been used to constrain the Euler vector for this time period. Instead, it has been excluded due to the *a posteriori* analysis of the velocity residuals (see Supp. Figure 13).

LHCL

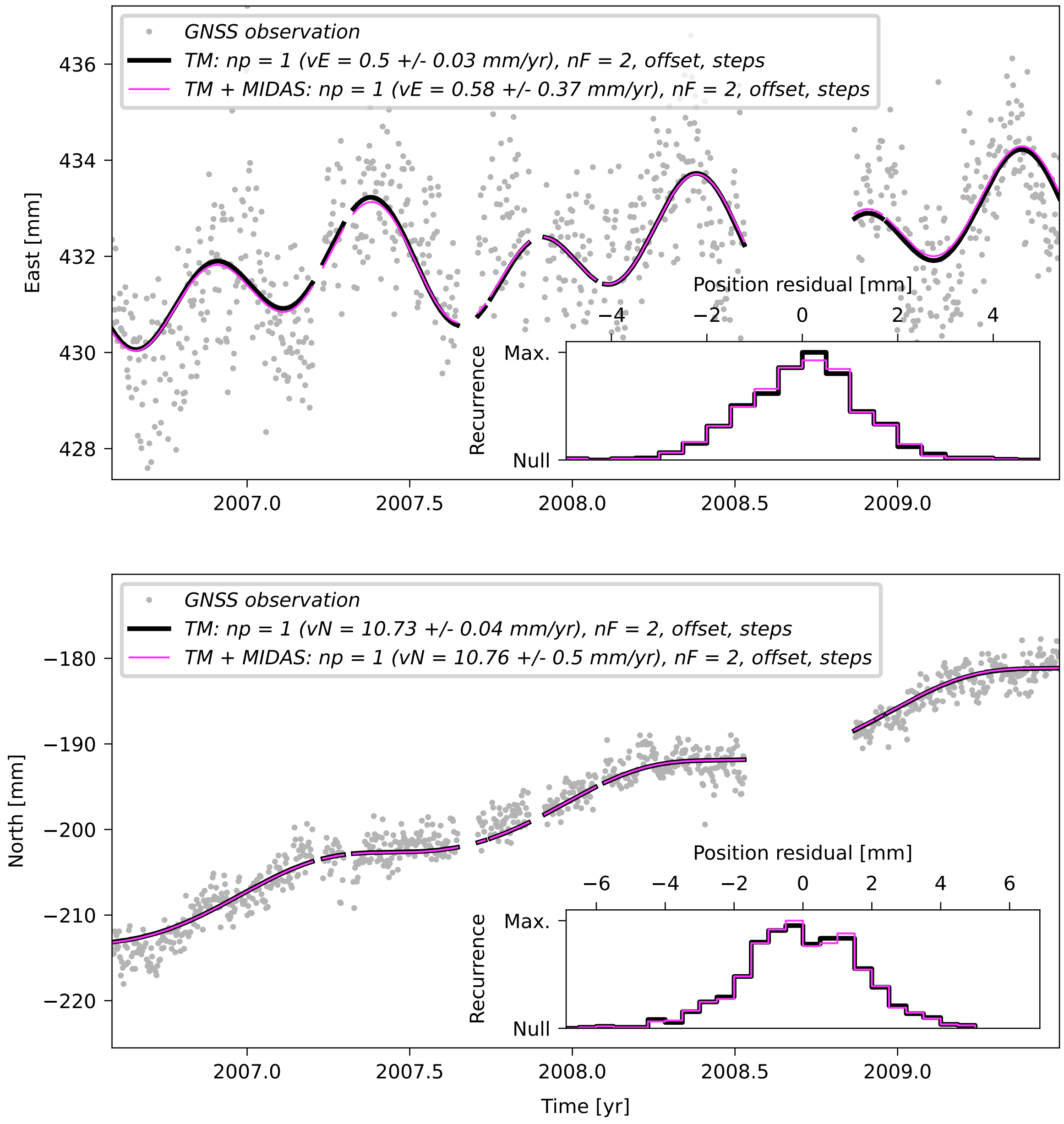

Supplementary Figure 46. Same as Supplementary Figure 18, but showing the time series and TMs of station LHCL for the period from July 2006 to June 2009.

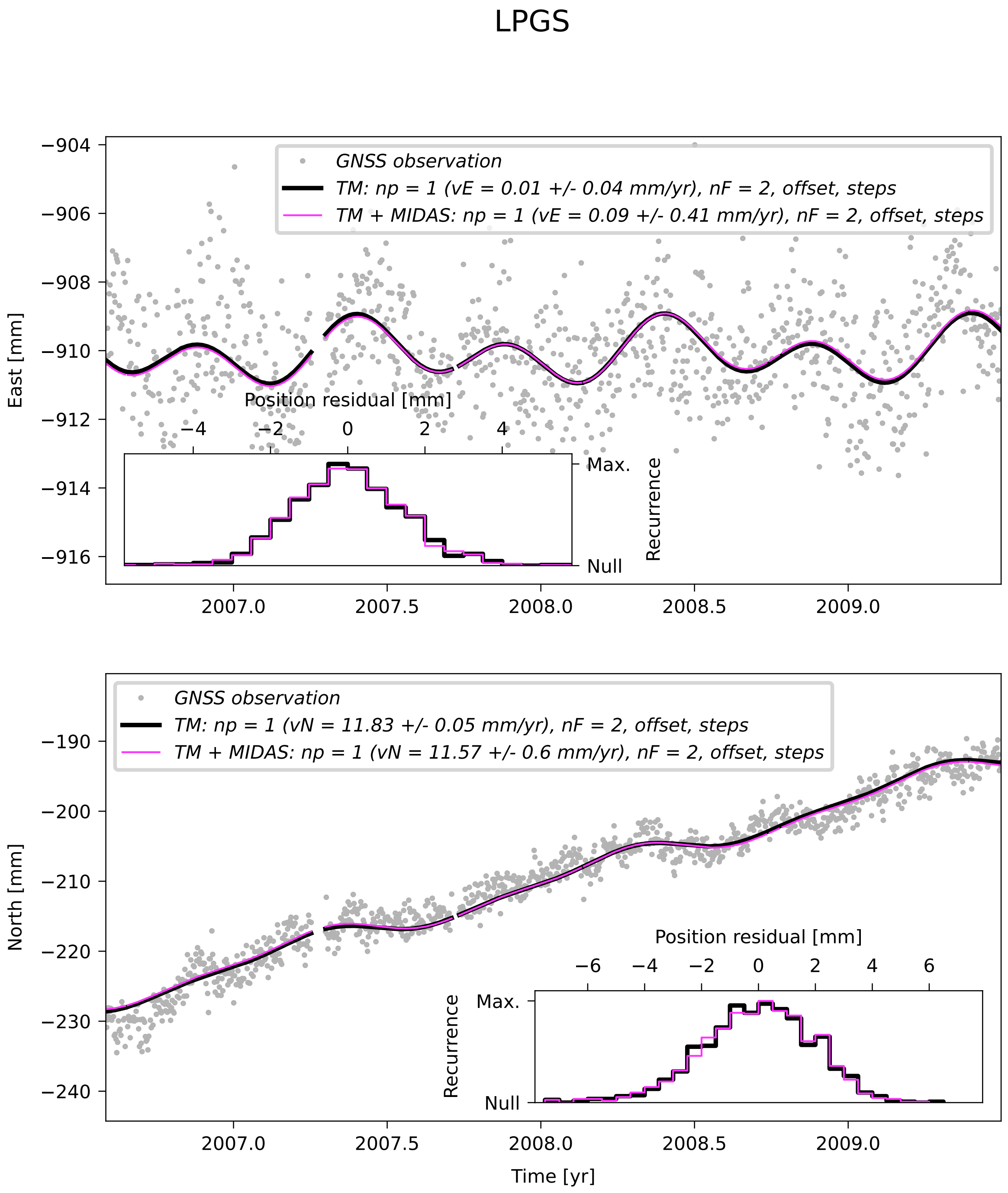

Supplementary Figure 47. Same as Supplementary Figure 18, but showing the time series and TMs of station LPGS for the period from July 2006 to June 2009.

MAPA

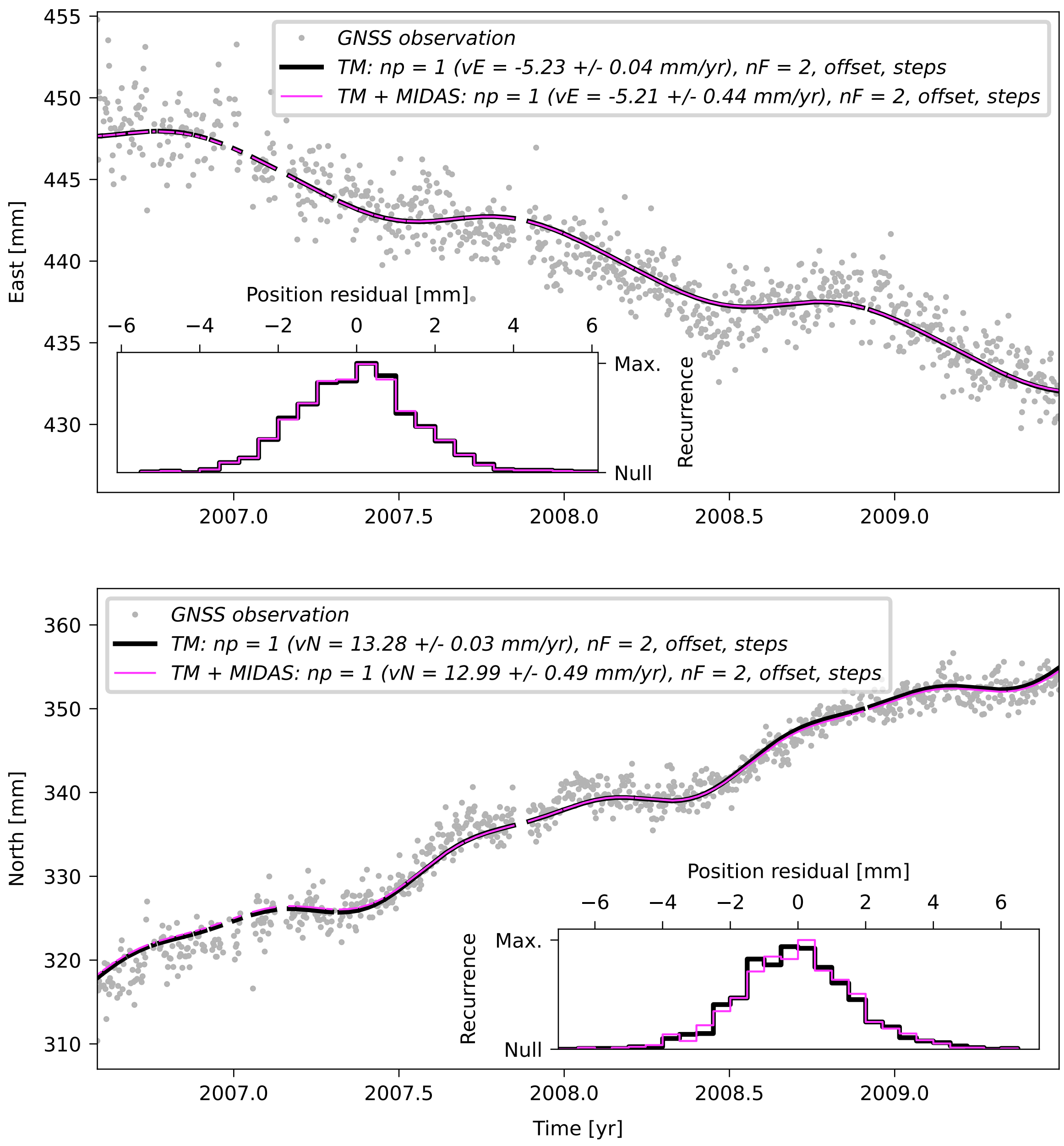

Supplementary Figure 48. Same as Supplementary Figure 18, but showing the time series and TMs of station MAPA for the period from July 2006 to June 2009.

MCLA

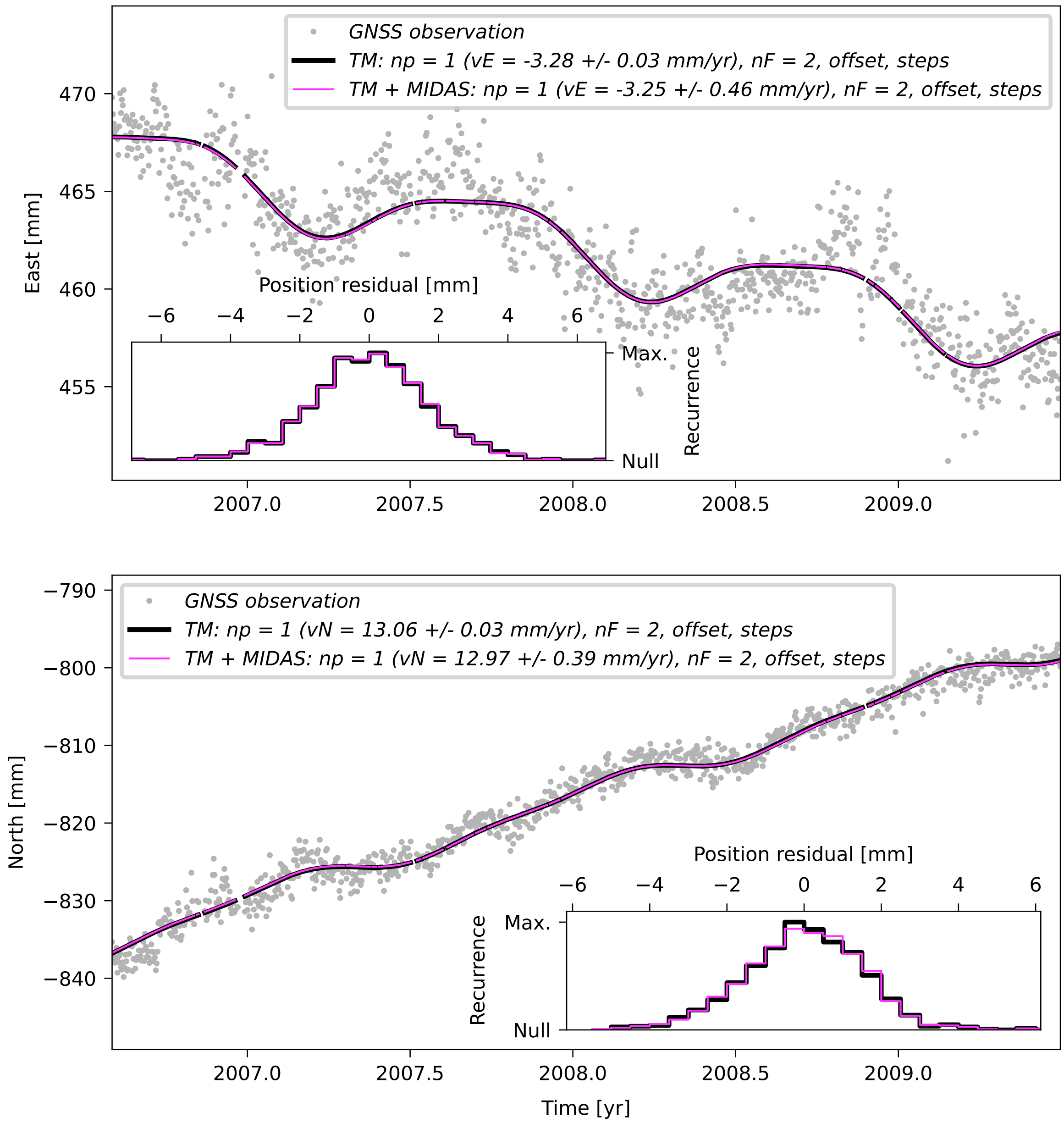

Supplementary Figure 49. Same as Supplementary Figure 18, but showing the time series and TMs of station MCLA for the period from July 2006 to June 2009.

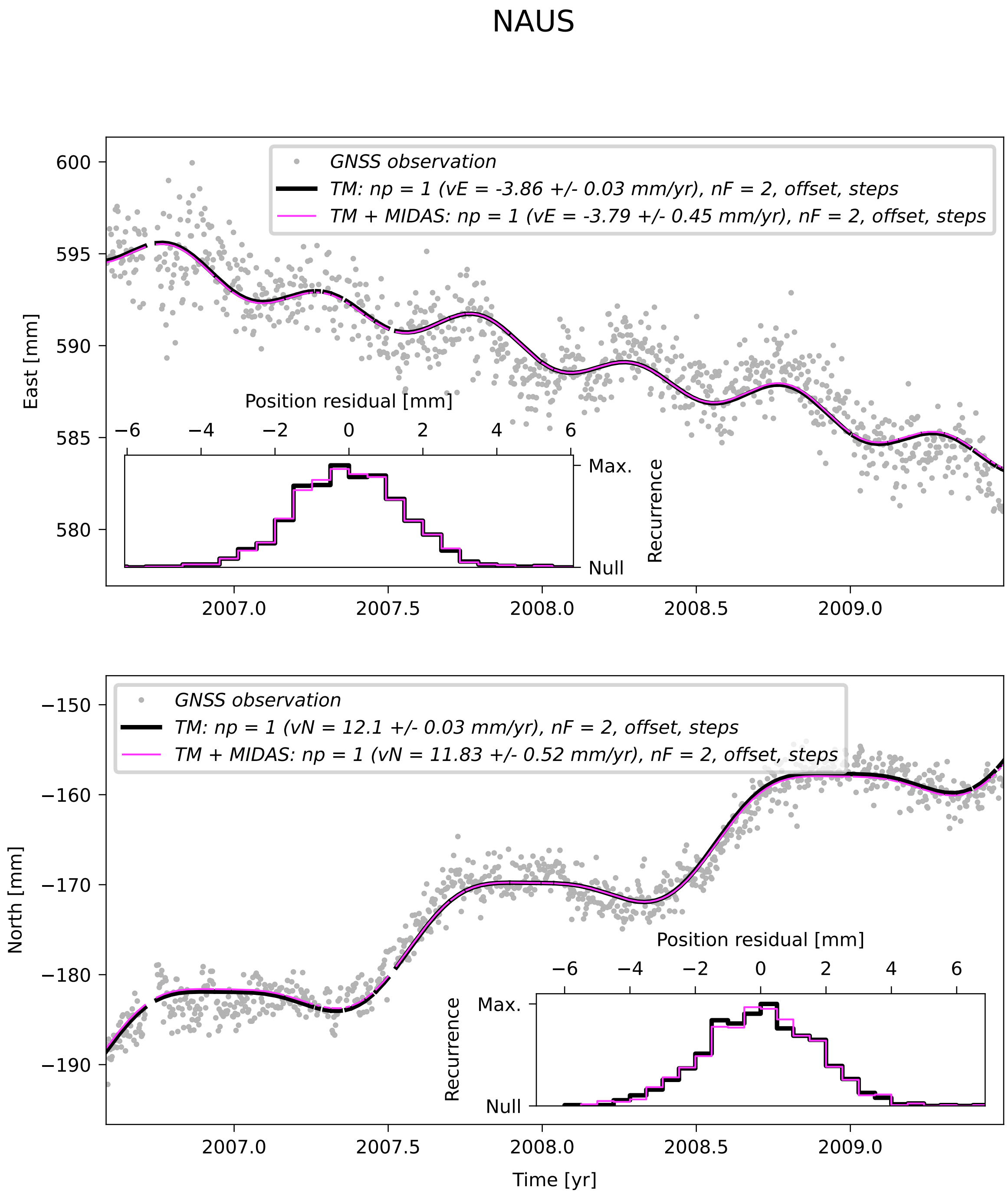

Supplementary Figure 50. Same as Supplementary Figure 18, but showing the time series and TMs of station NAUS for the period from July 2006 to June 2009.

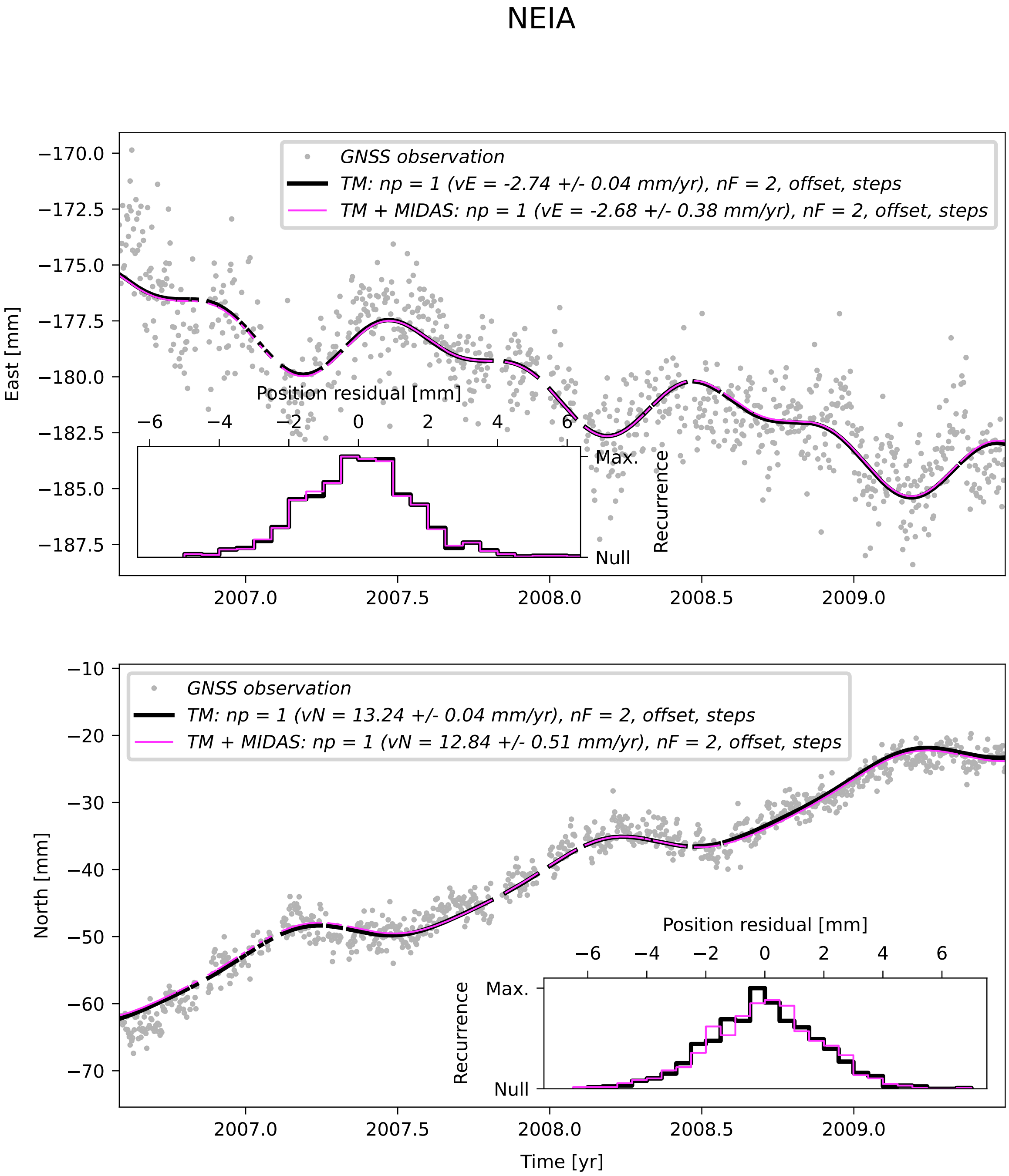

Supplementary Figure 51. Same as Supplementary Figure 18, but showing the time series and TMs of station NEIA for the period from July 2006 to June 2009.

PMB1

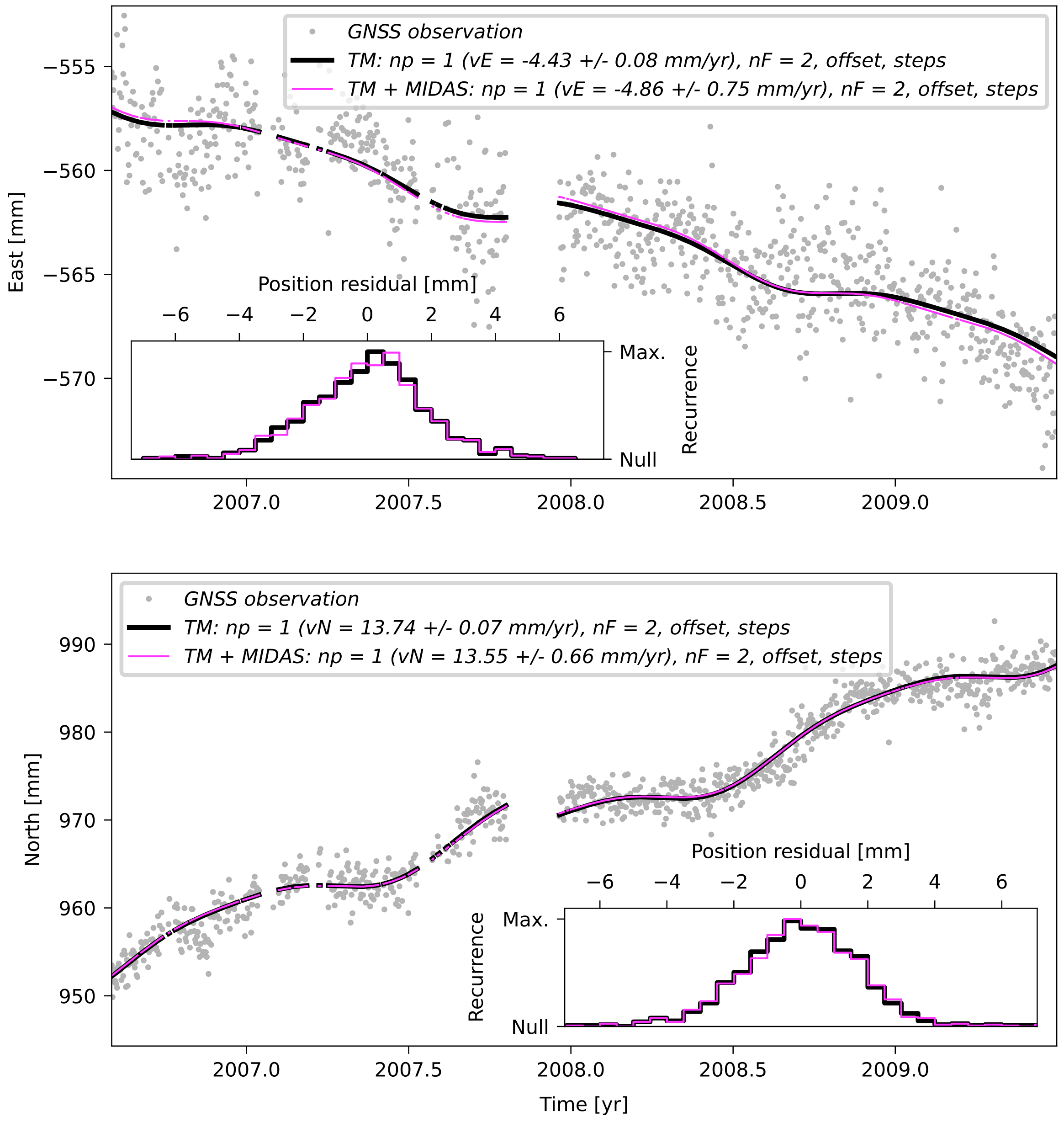

Supplementary Figure 52. Same as Supplementary Figure 18, but showing the time series and TMs of station PMB1 for the period from July 2006 to June 2009.

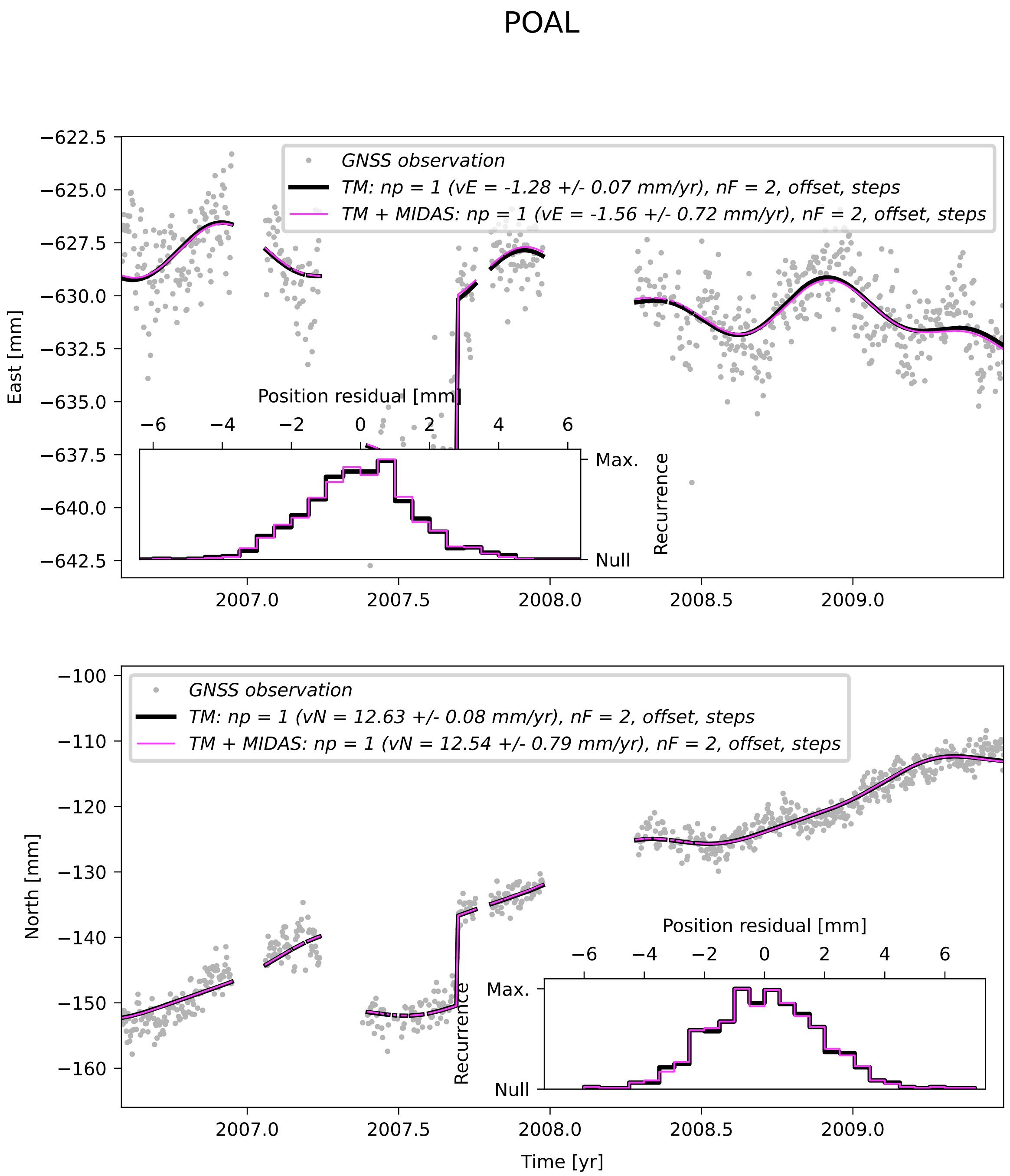

Supplementary Figure 53. Same as Supplementary Figure 18, but showing the time series and TMs of station POAL for the period from July 2006 to June 2009.

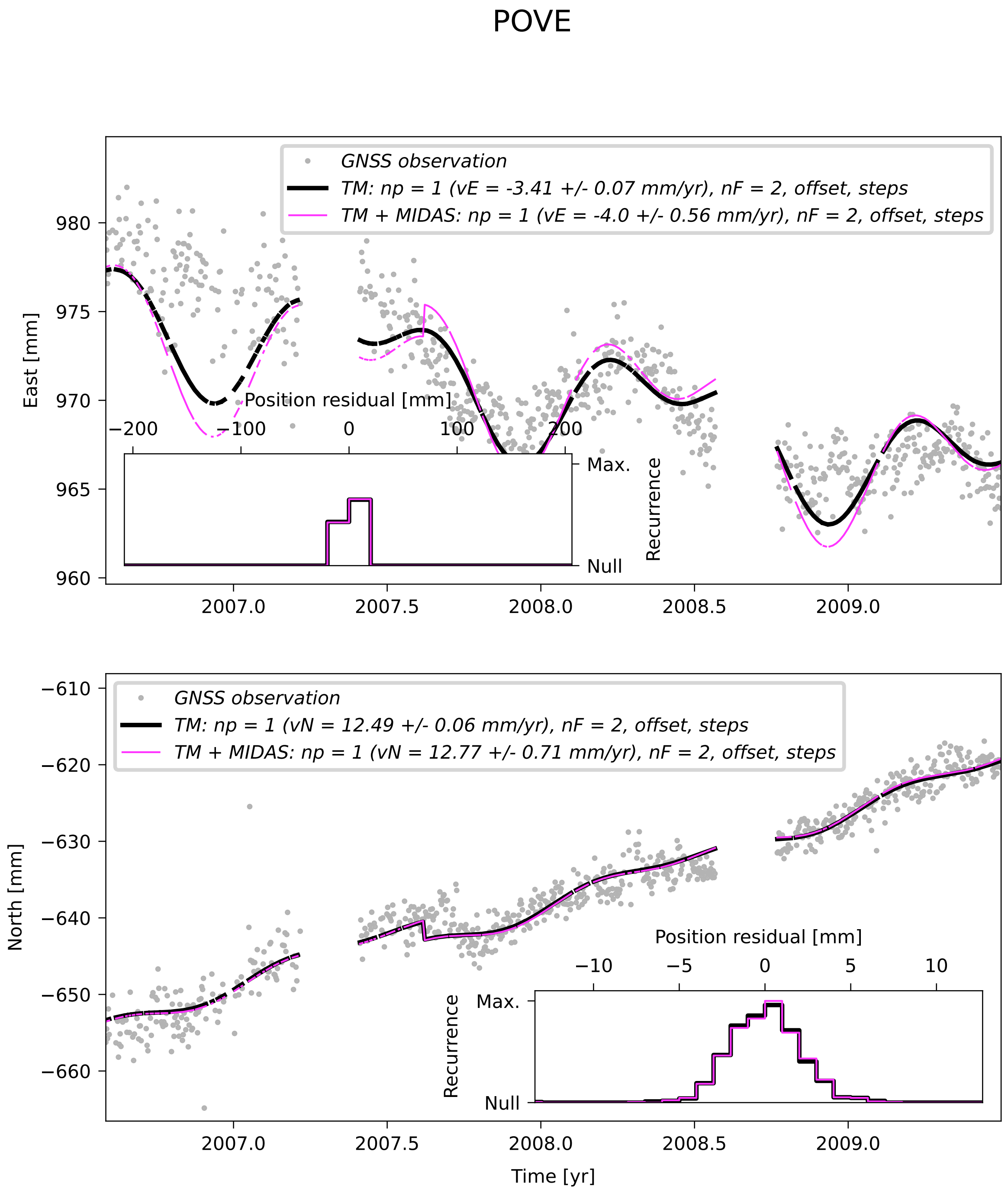

Supplementary Figure 54. Same as Supplementary Figure 18, but showing the time series and TMs of station POVE for the period from July 2006 to June 2009.

PPTE

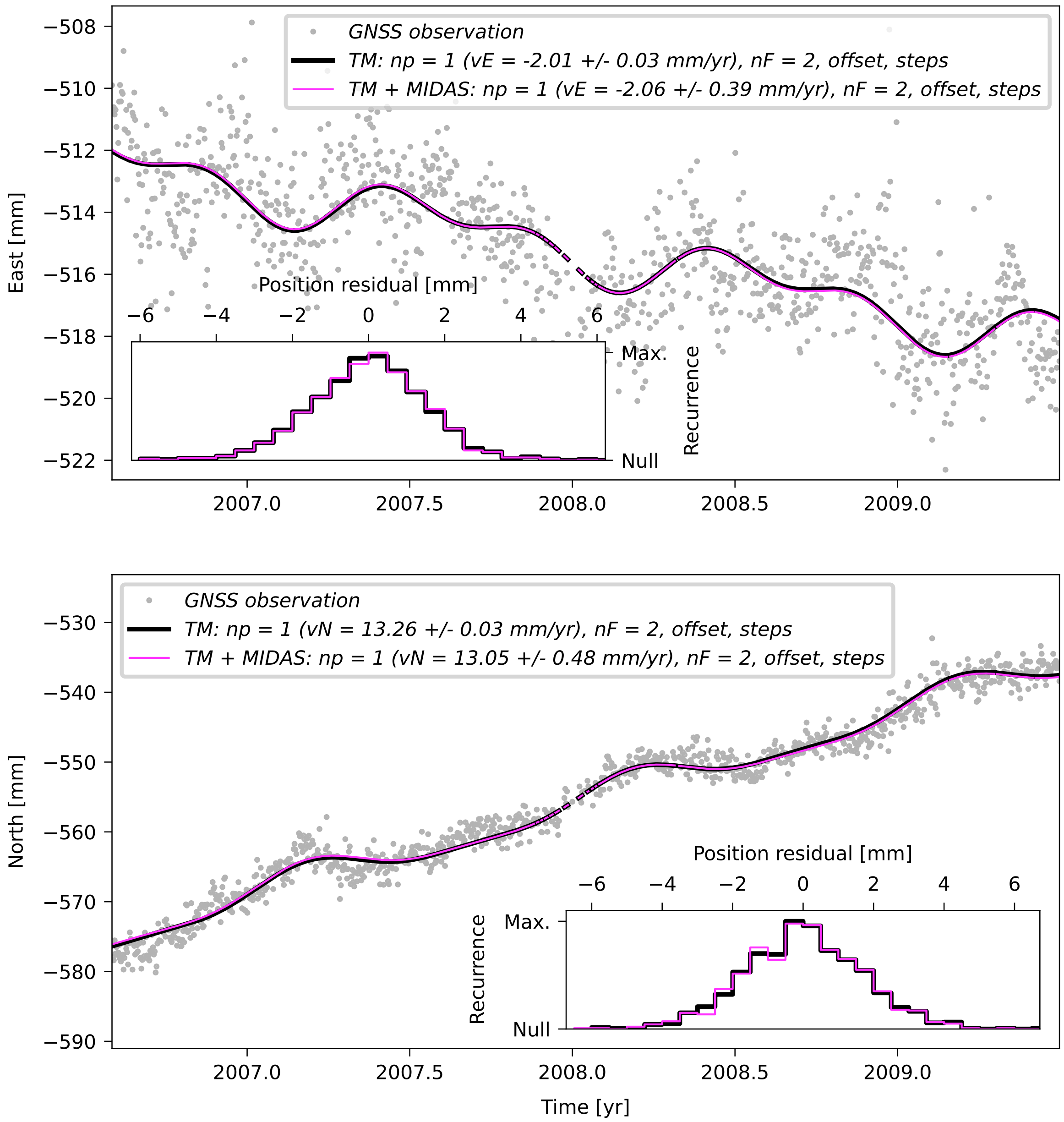

Supplementary Figure 55. Same as Supplementary Figure 18, but showing the time series and TMs of station PPTE for the period from July 2006 to June 2009.

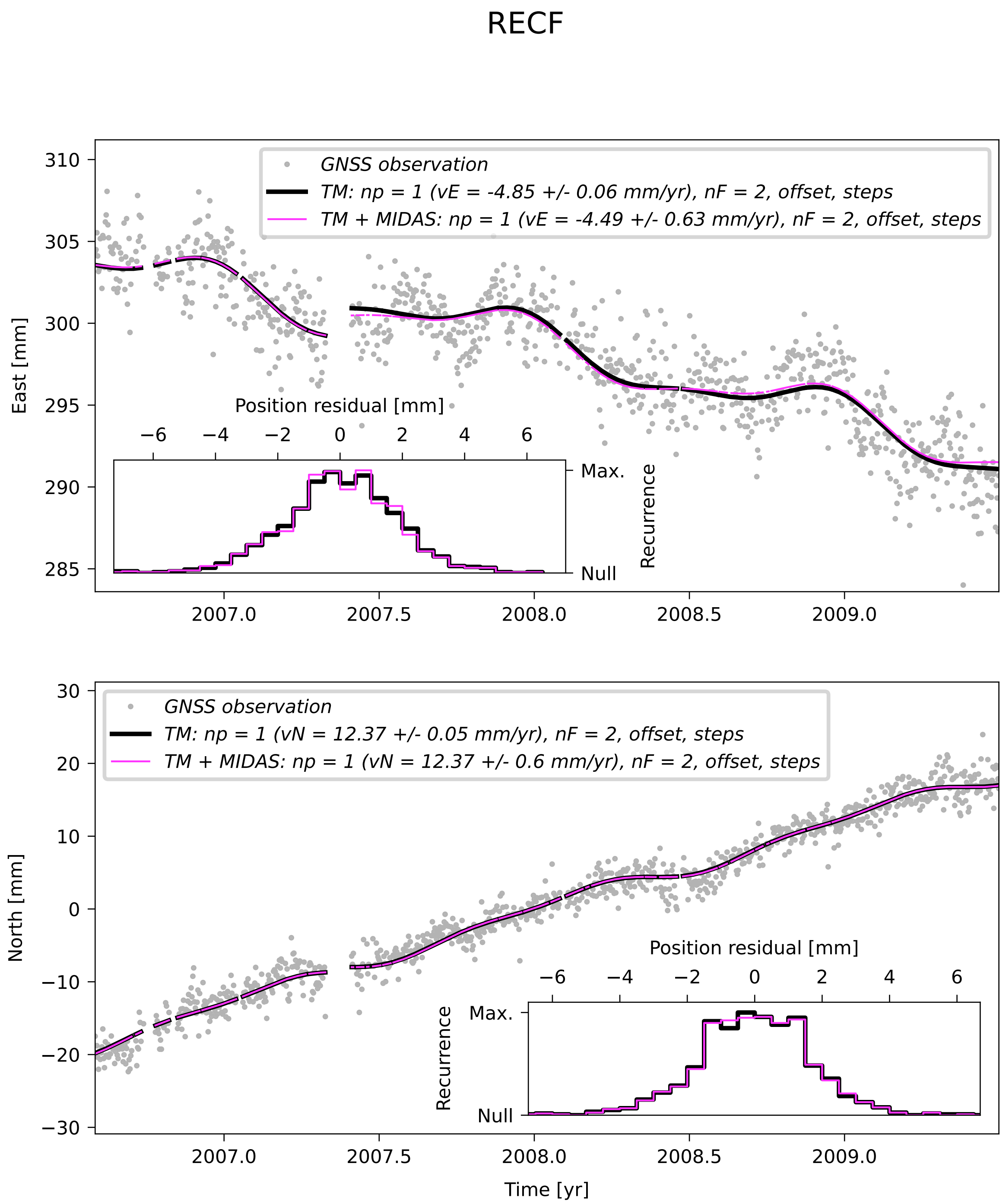

Supplementary Figure 56. Same as Supplementary Figure 18, but showing the time series and TMs of station RECF for the period from July 2006 to June 2009.

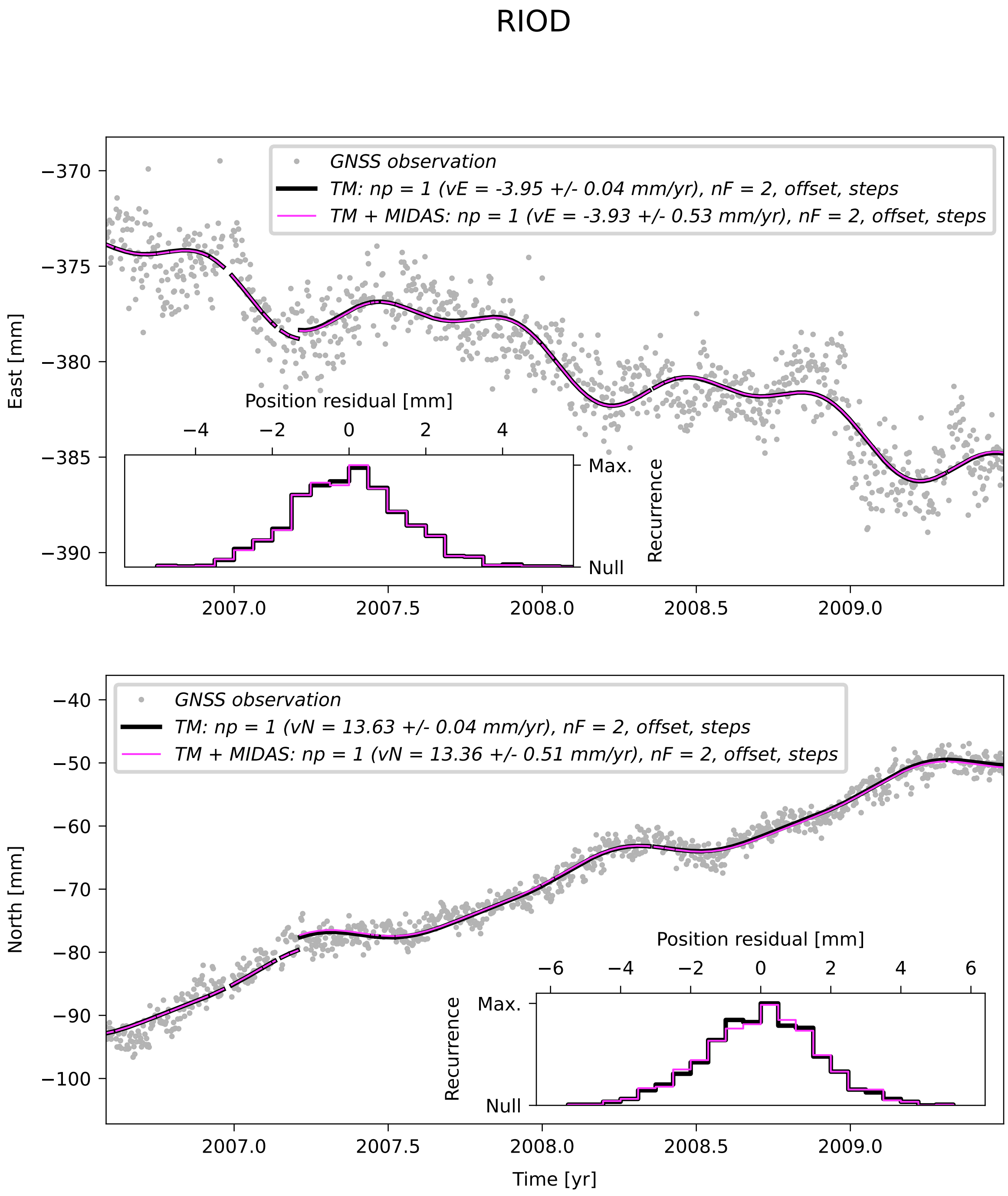

Supplementary Figure 57. Same as Supplementary Figure 18, but showing the time series and TMs of station RIOD for the period from July 2006 to June 2009.

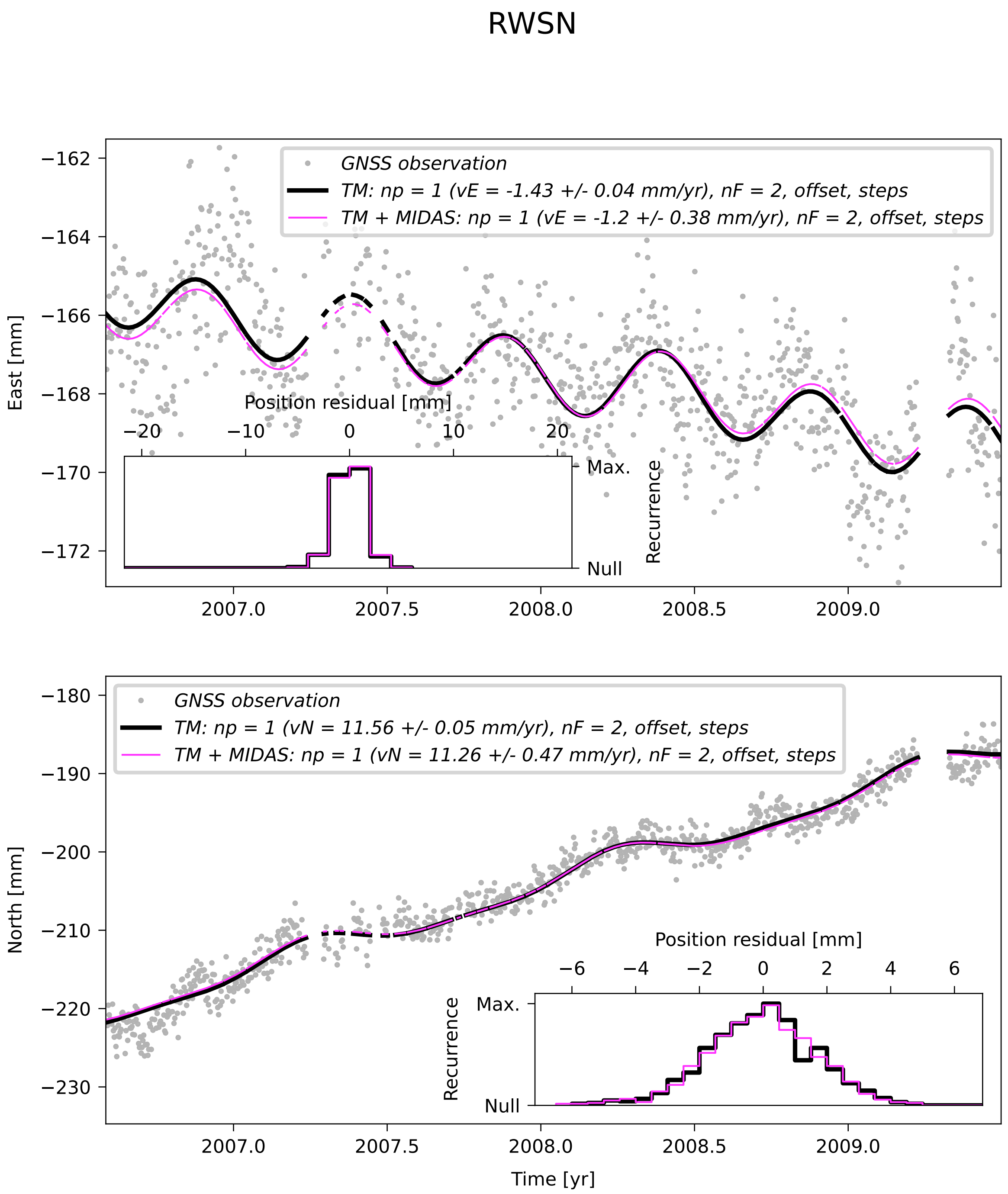

Supplementary Figure 58. Same as Supplementary Figure 18, but showing the time series and TMs of station RWSN for the period from July 2006 to June 2009. Please note that this site has not been used to constrain the Euler vector for this time period. Instead, it has been excluded due to the *a posteriori* analysis of the velocity residuals (see Supp. Figure 13).

SMAR

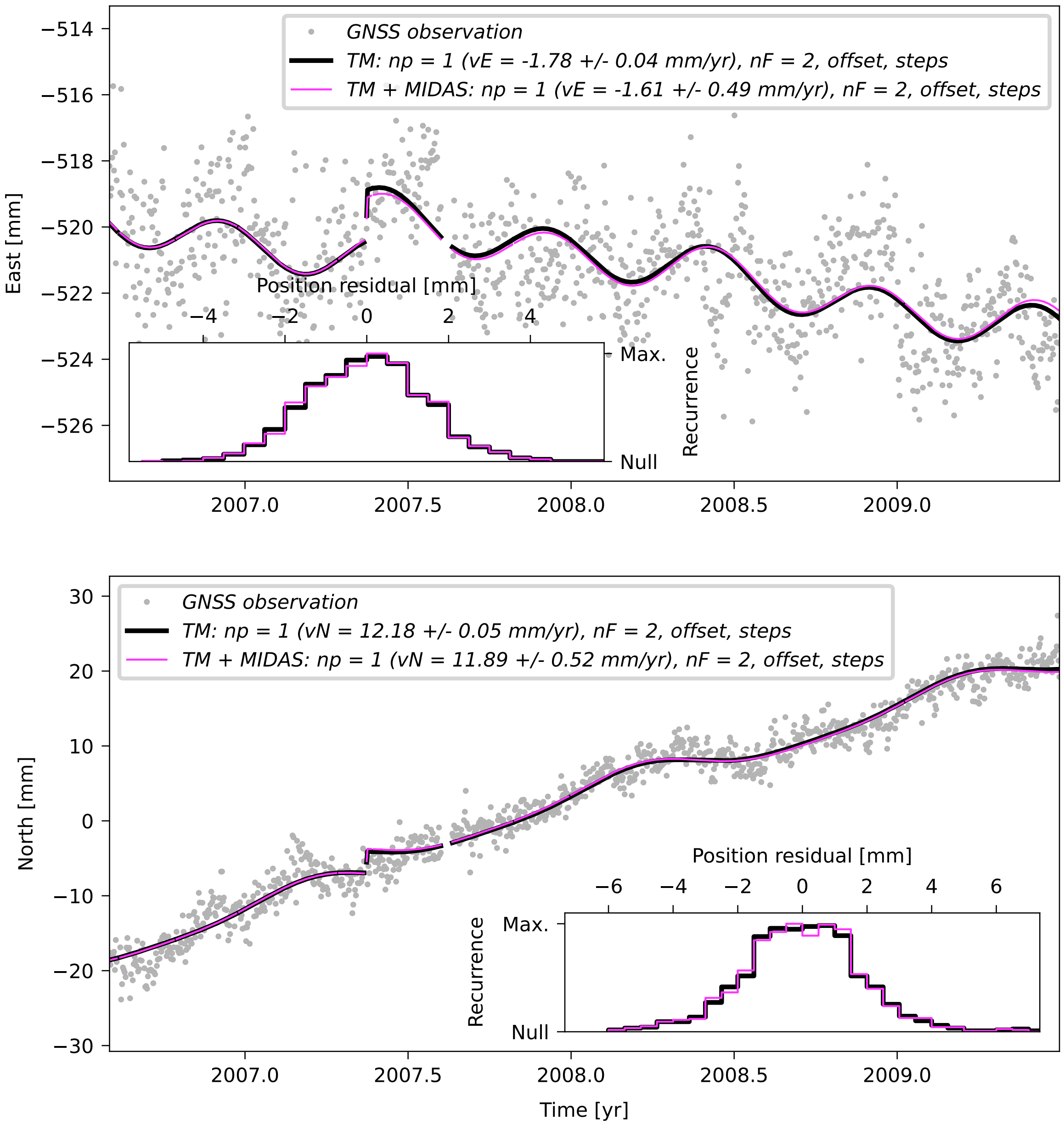

Supplementary Figure 59. Same as Supplementary Figure 18, but showing the time series and TMs of station SMAR for the period from July 2006 to June 2009.

SRZN

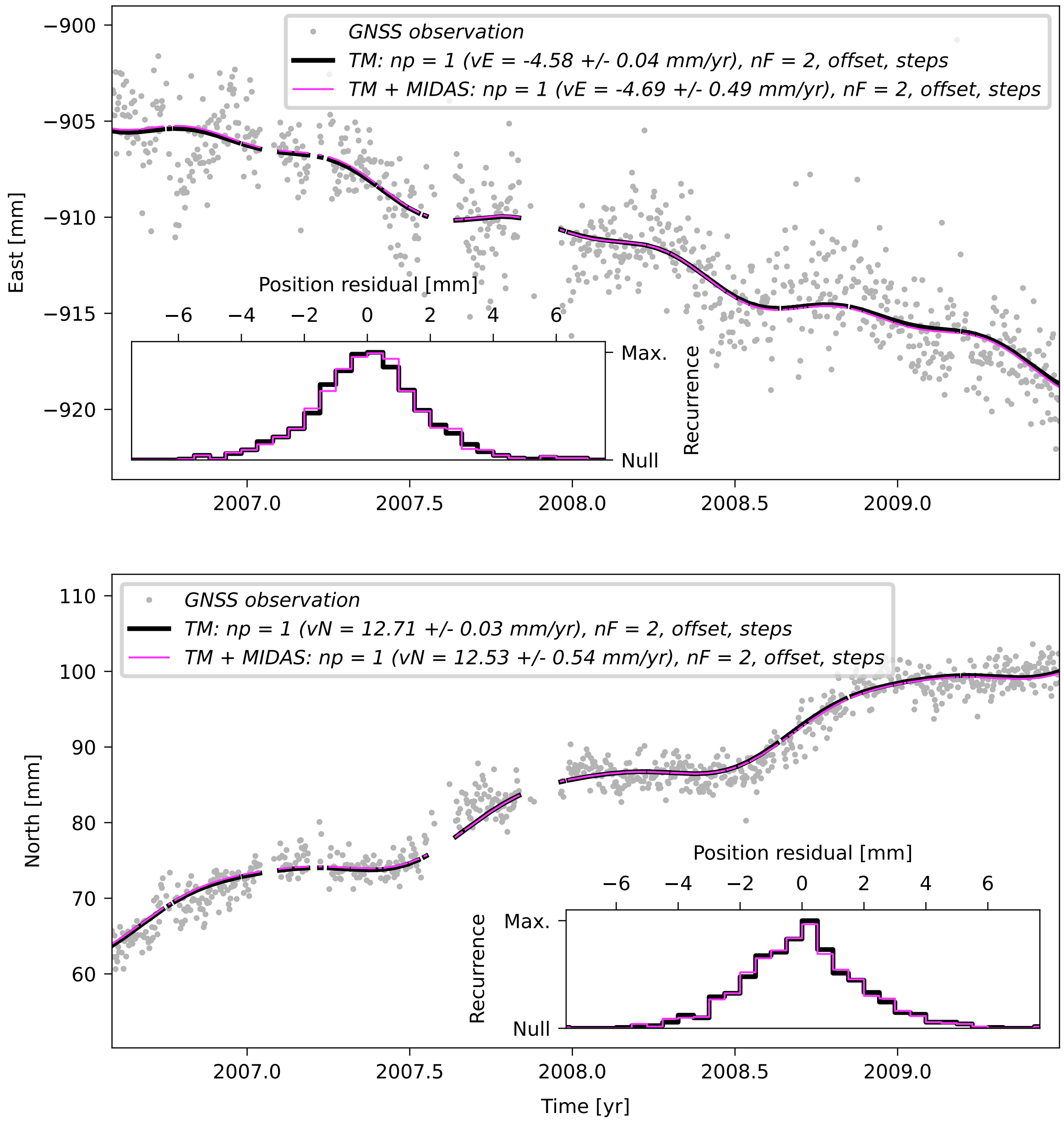

Supplementary Figure 60. Same as Supplementary Figure 18, but showing the time series and TMs of station SRZN for the period from July 2006 to June 2009.

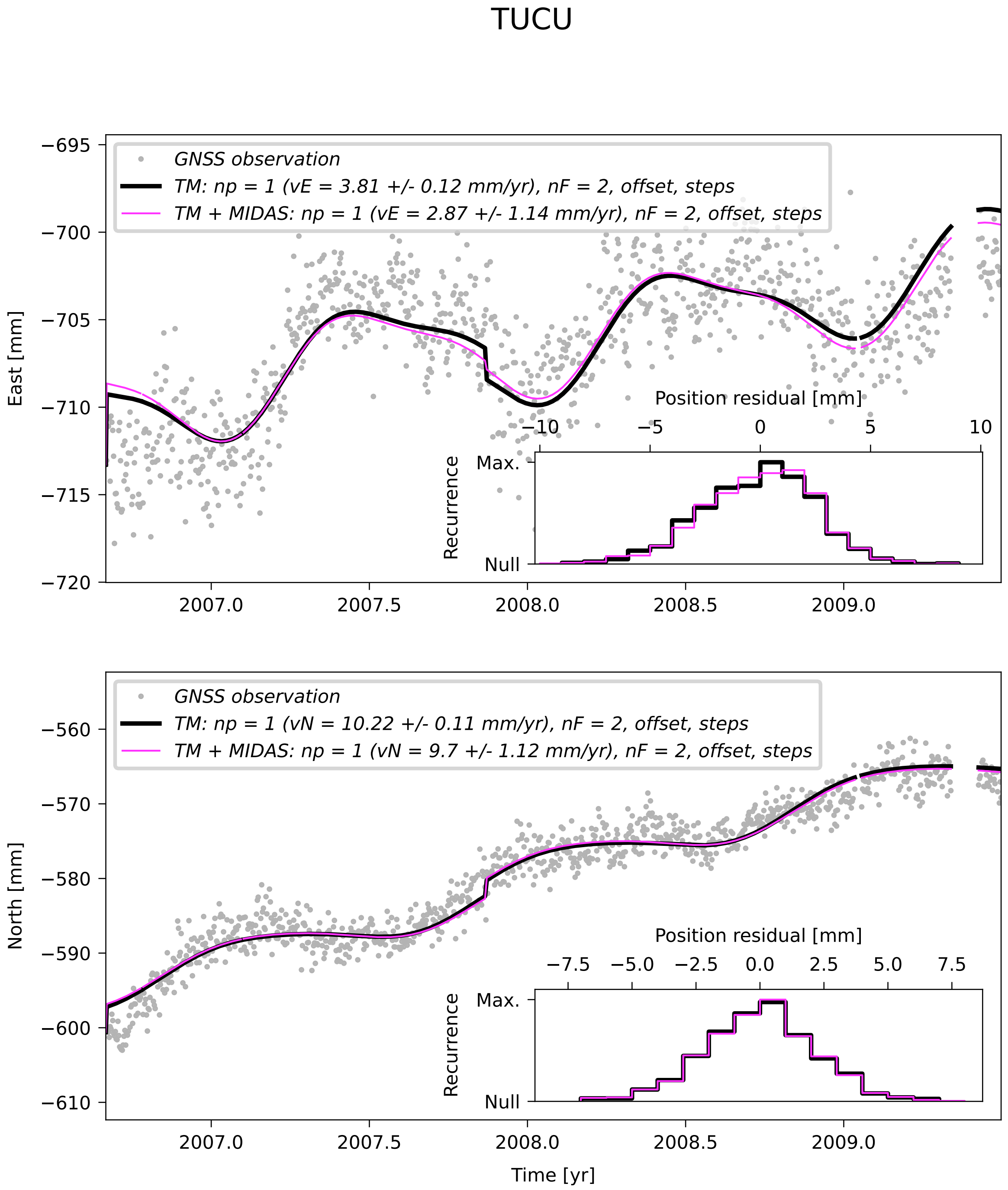

Supplementary Figure 61. Same as Supplementary Figure 18, but showing the time series and TMs of station TUCU for the period from July 2006 to June 2009. Please note that this site has not been used to constrain the Euler vector for this time period. Instead, it has been excluded due to the *a posteriori* analysis of the velocity residuals (see Supp. Figure 13).

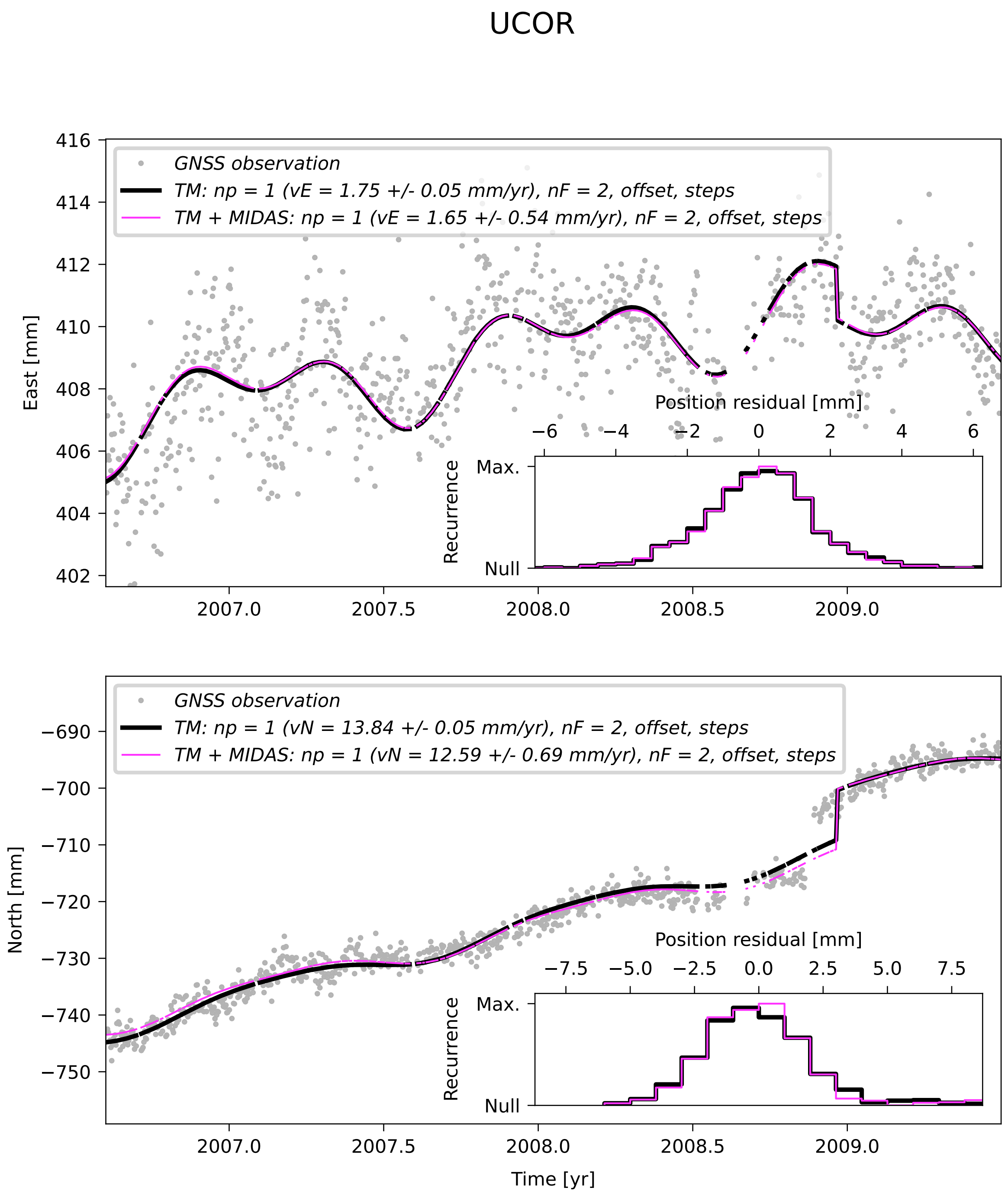

Supplementary Figure 62. Same as Supplementary Figure 18, but showing the time series and TMs of station UCOR for the period from July 2006 to June 2009. Please note that this site has not been used to constrain the Euler vector for this time period. Instead, it has been excluded due to the *a posteriori* analysis of the velocity residuals (see Supp. Figure 13).

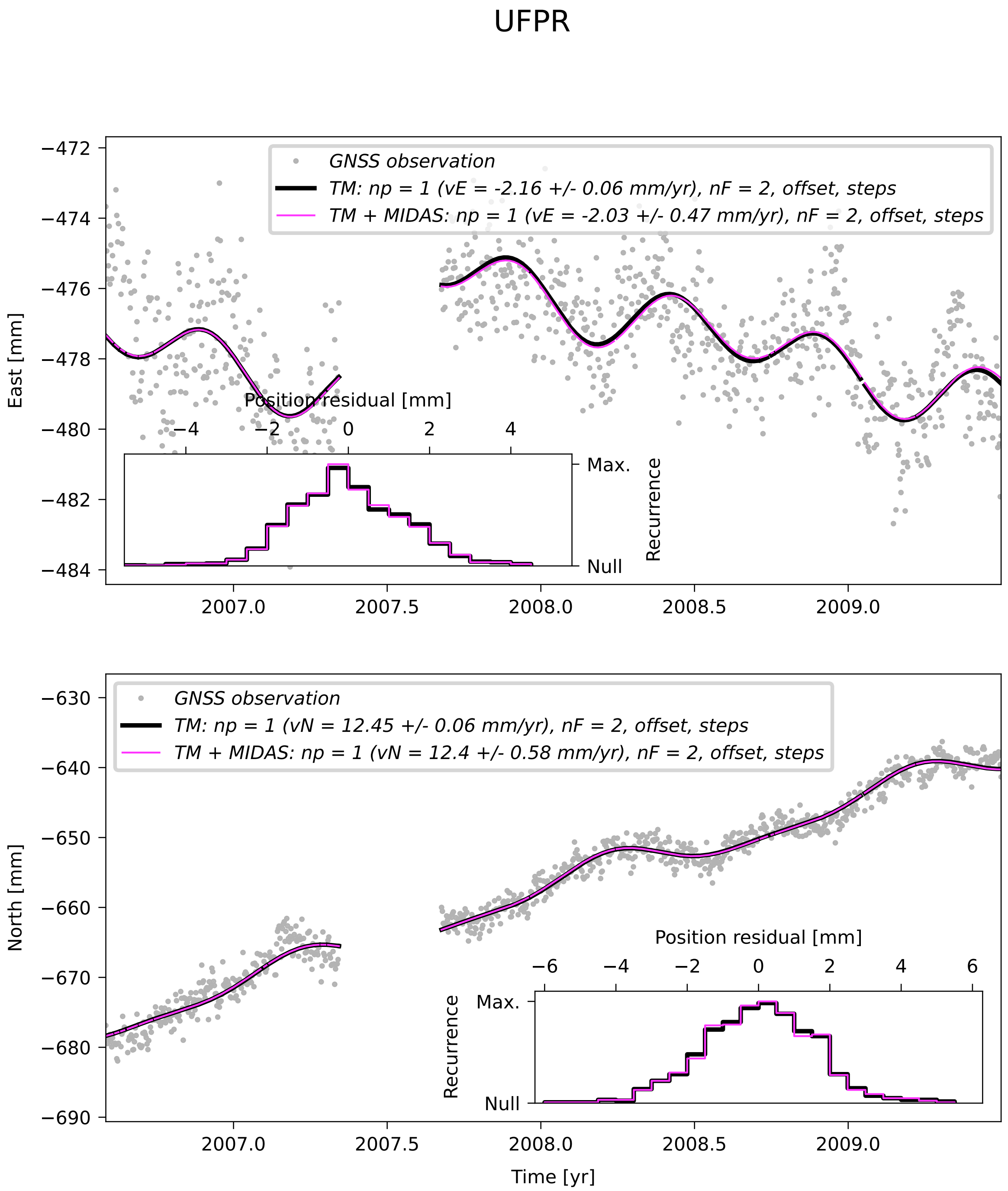

Supplementary Figure 63. Same as Supplementary Figure 18, but showing the time series and TMs of station UFPR for the period from July 2006 to June 2009.

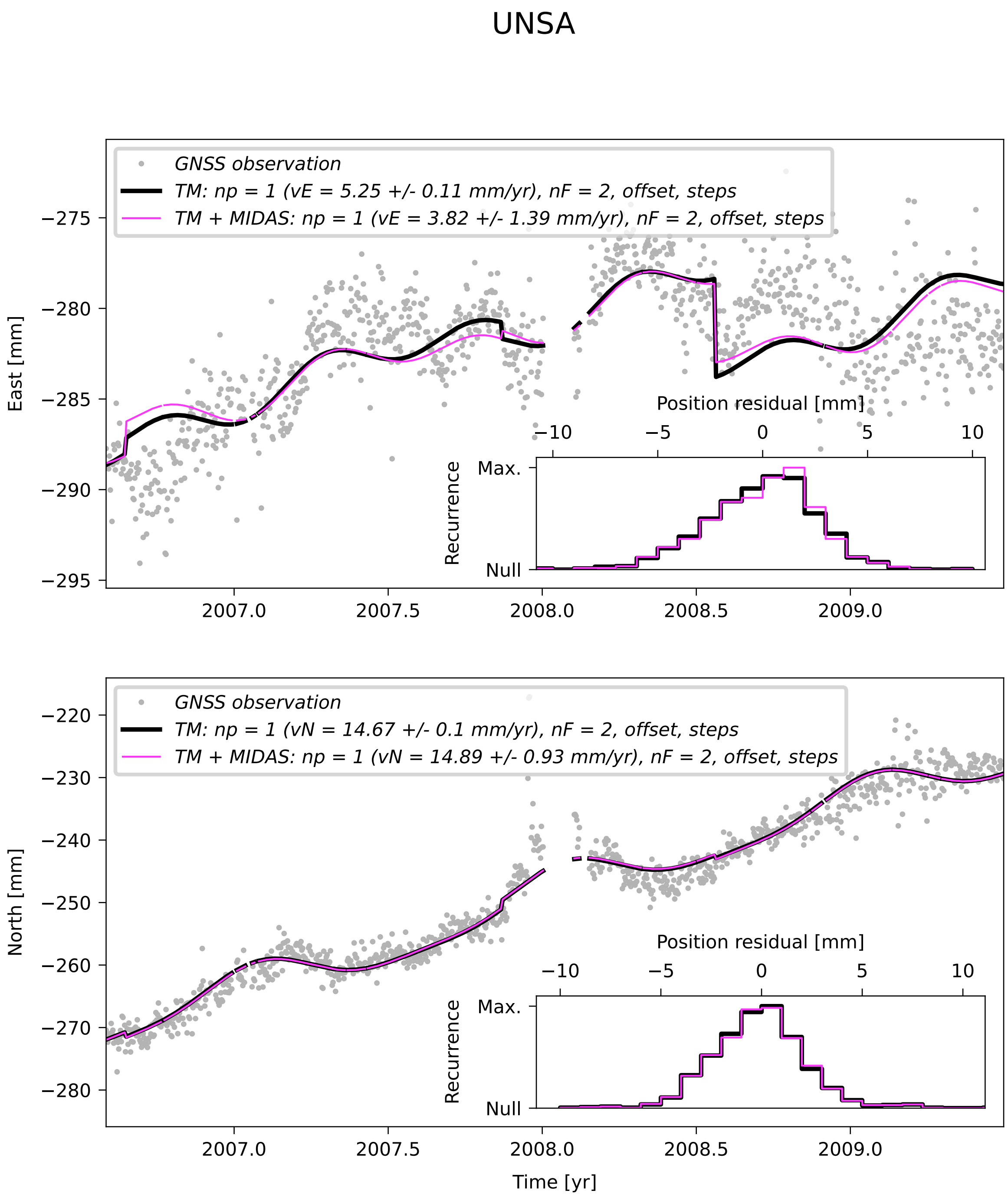

Supplementary Figure 64. Same as Supplementary Figure 18, but showing the time series and TMs of station UNSA for the period from July 2006 to June 2009. Please note that this site has not been used to constrain the Euler vector for this time period. Instead, it has been excluded due to the *a posteriori* analysis of the velocity residuals (see Supp. Figure 13).

VARG

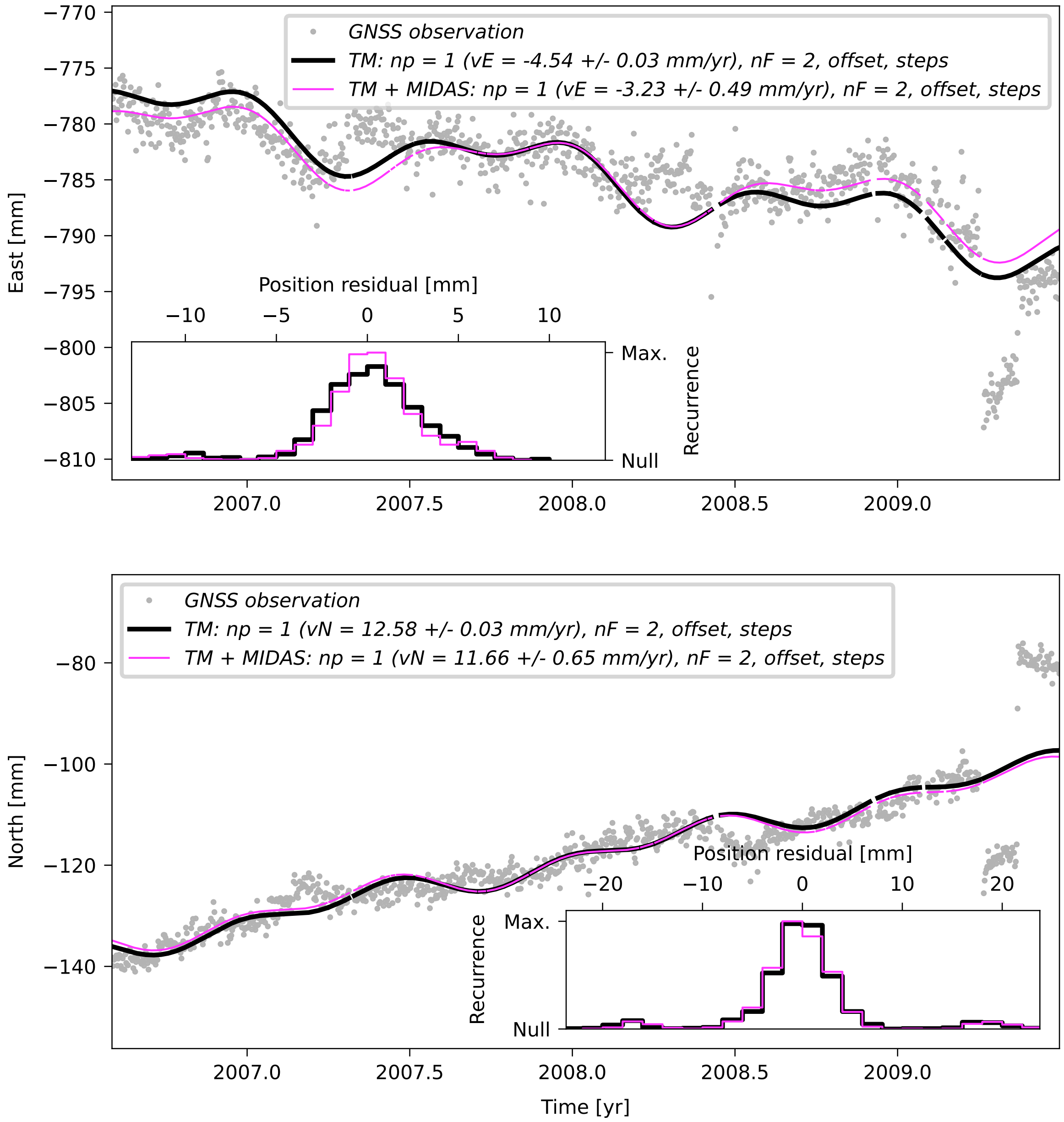

Supplementary Figure 65. Same as Supplementary Figure 18, but showing the time series and TMs of station VARG for the period from July 2006 to June 2009.

VBCA

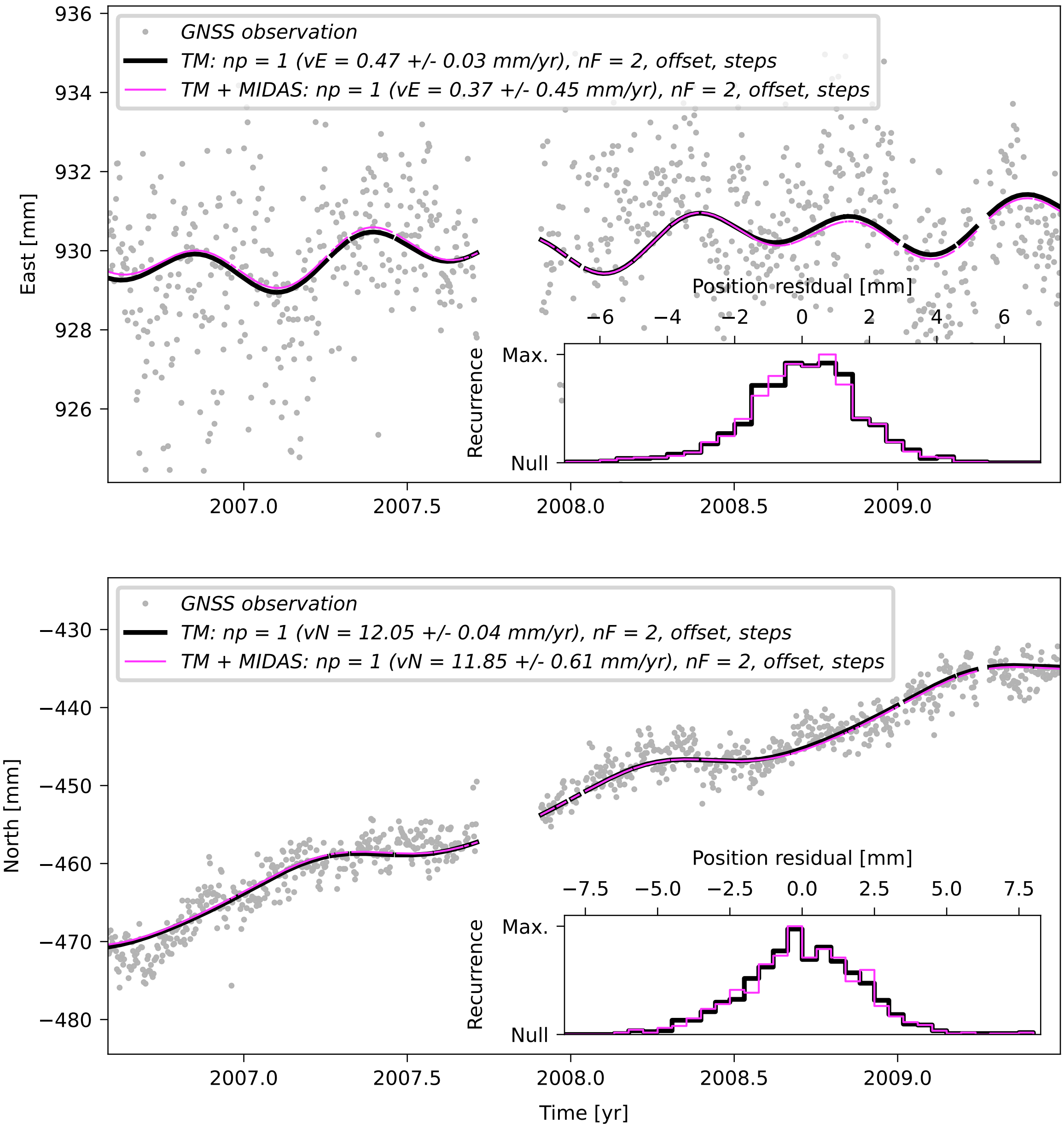

Supplementary Figure 66. Same as Supplementary Figure 18, but showing the time series and TMs of station VBCA for the period from July 2006 to June 2009.

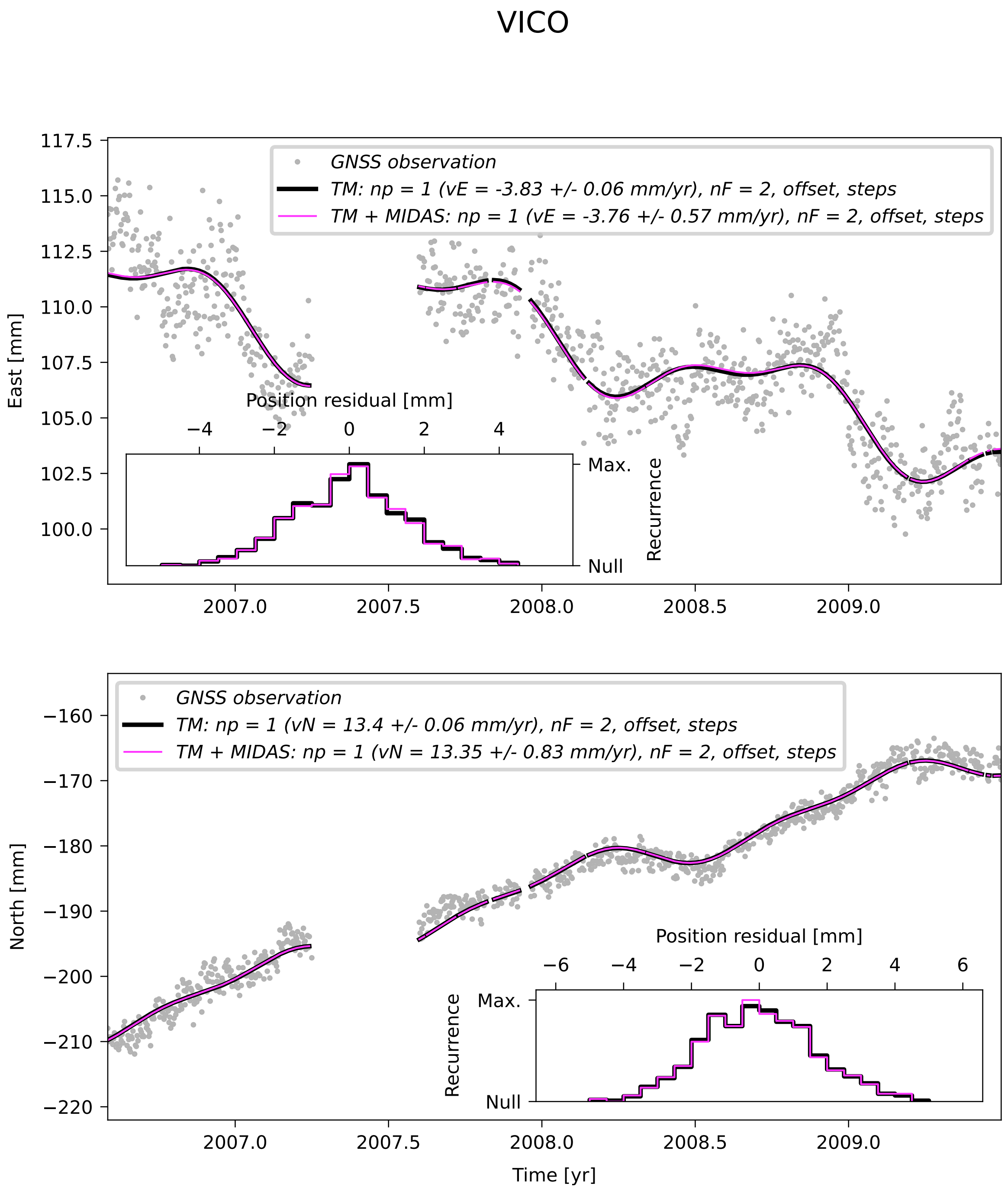

Supplementary Figure 67. Same as Supplementary Figure 18, but showing the time series and TMs of station VICO for the period from July 2006 to June 2009.

GLPS

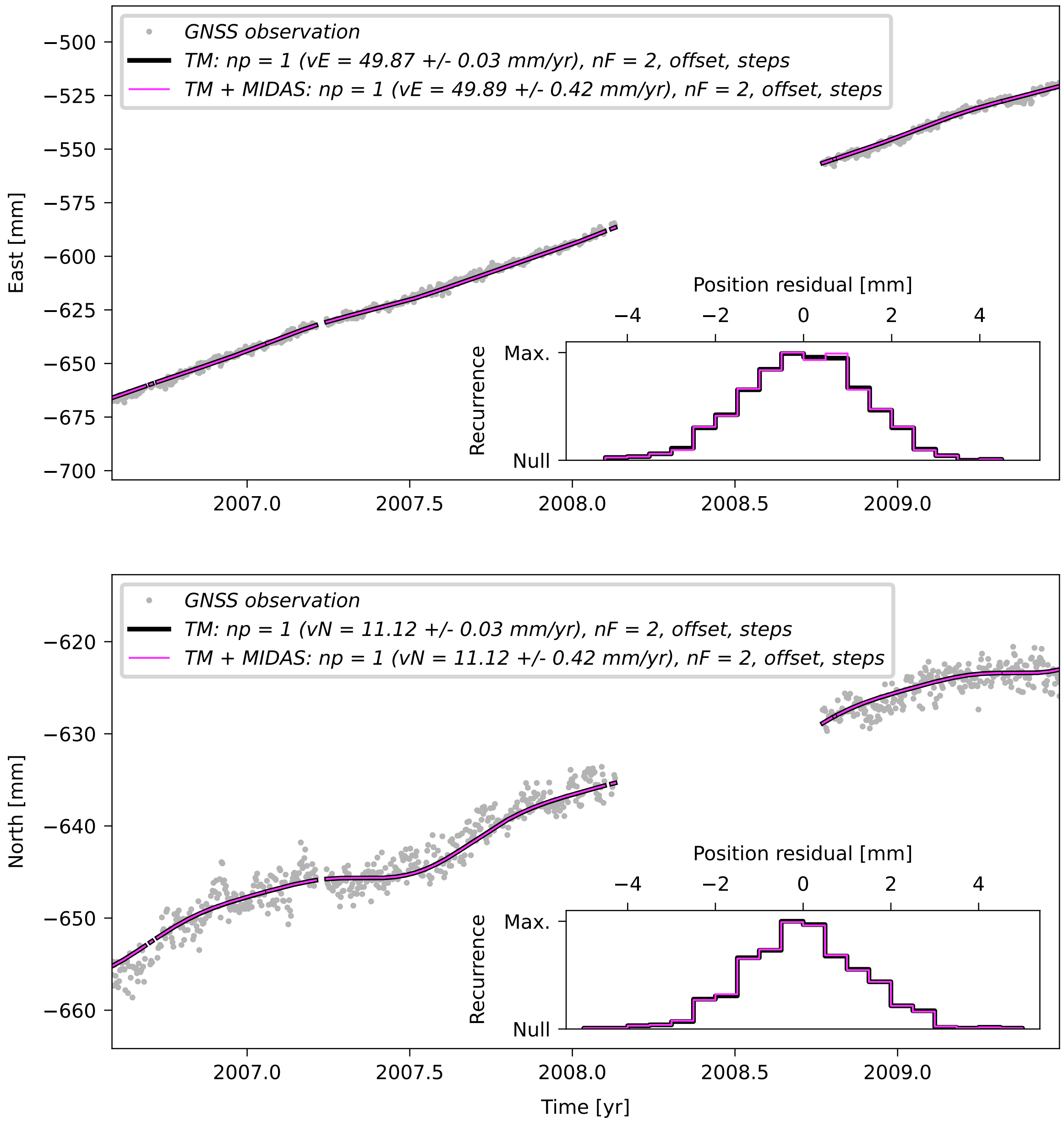

Supplementary Figure 68. Same as Supplementary Figure 18, but showing the time series and TMs of station GLPS for the period from July 2006 to June 2009.

ISPA

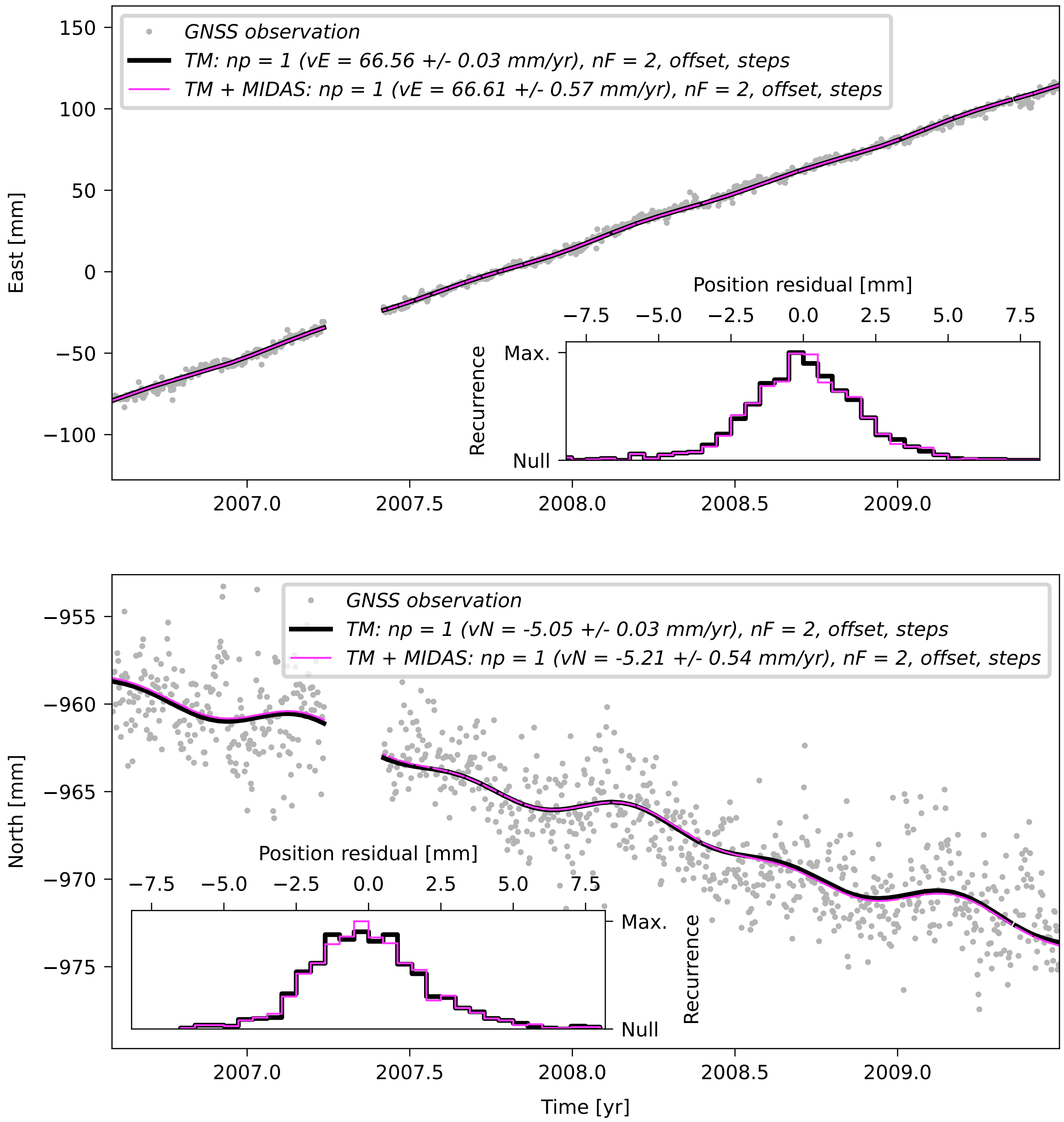

Supplementary Figure 69. Same as Supplementary Figure 18, but showing the time series and TMs of station ISPA for the period from July 2006 to June 2009.

BELE

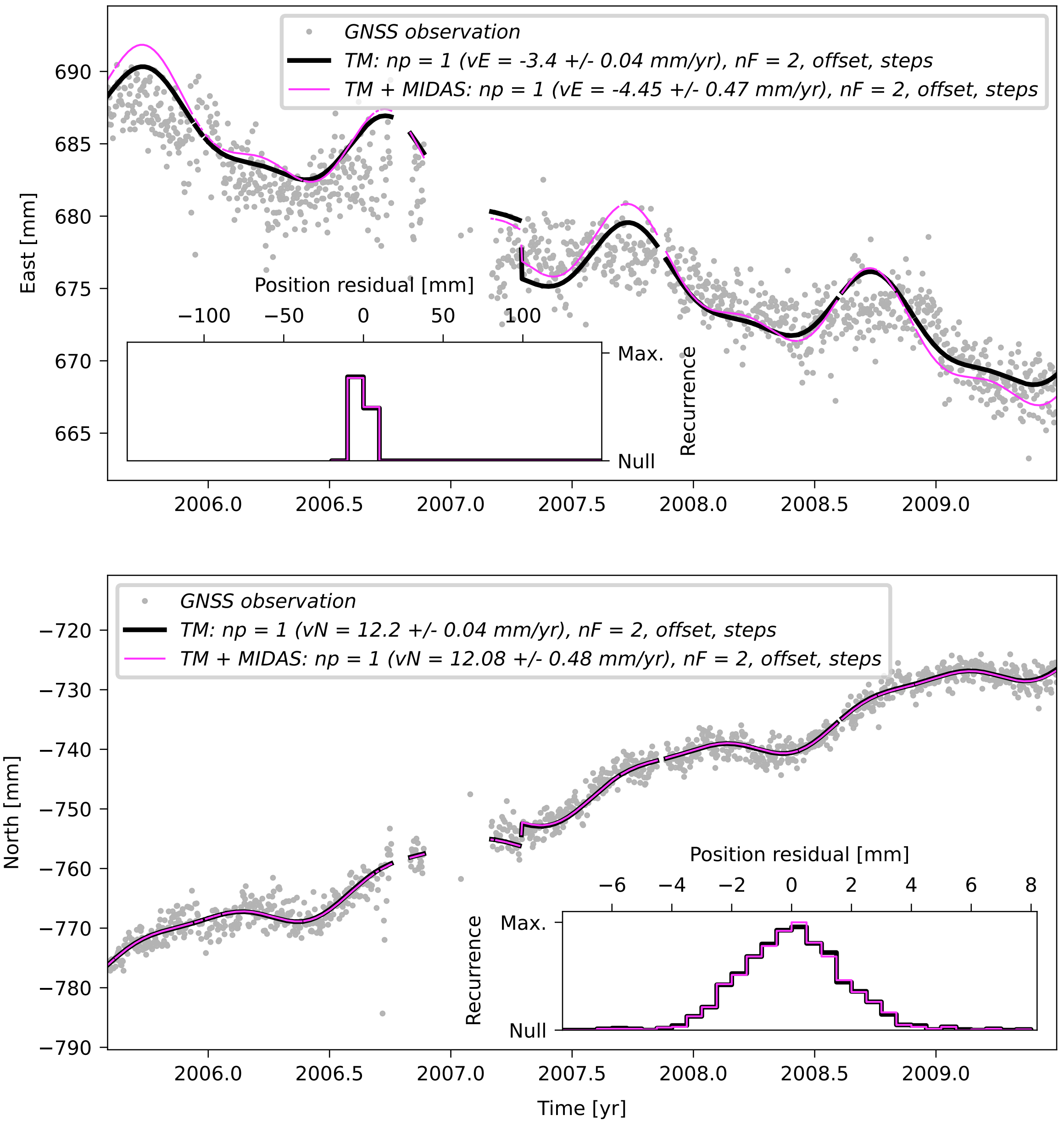

Supplementary Figure 70. Same as Supplementary Figure 18, but showing the time series and TMs of station BELE for the period from July 2005 to June 2009.

BOMJ

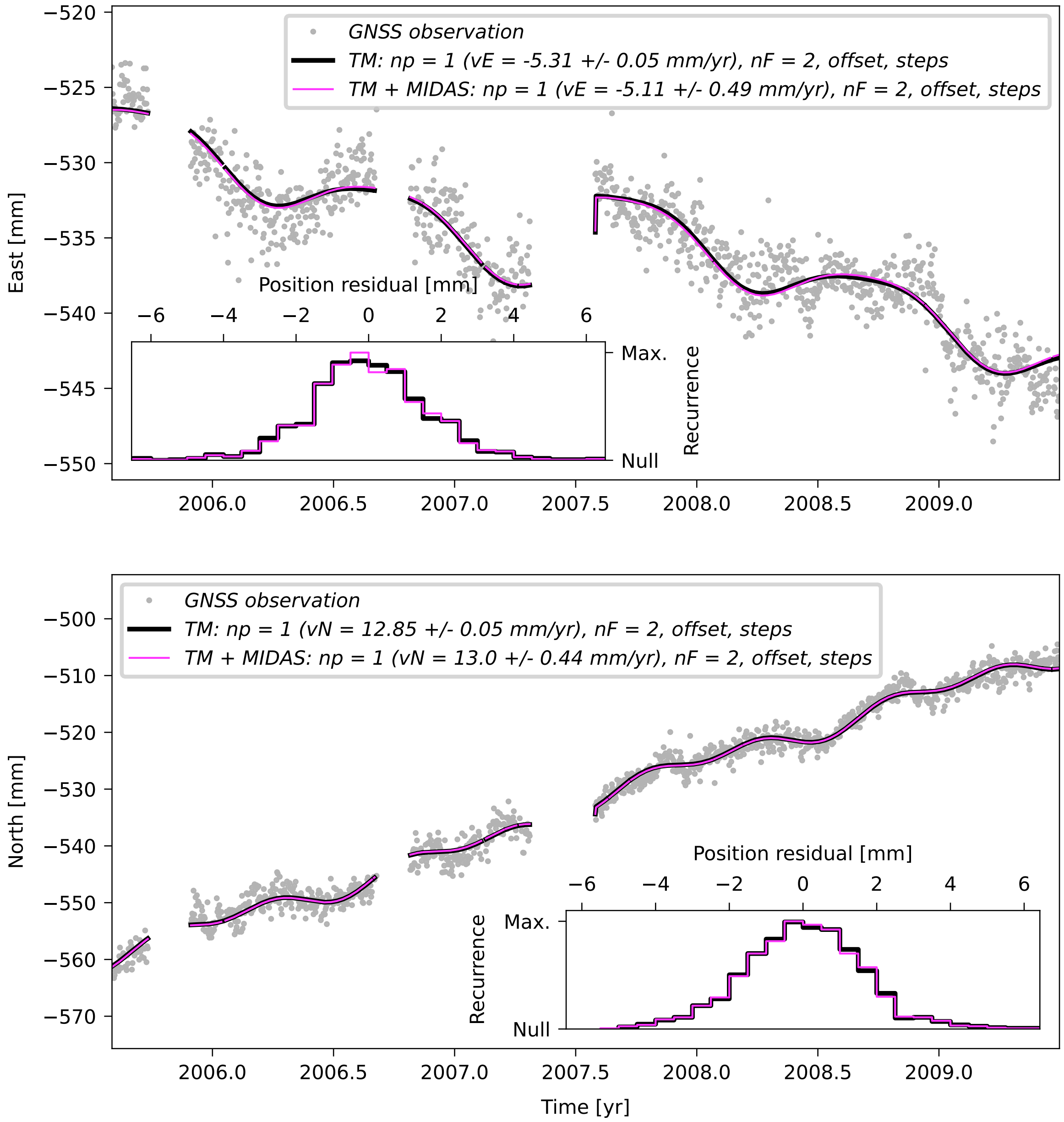

Supplementary Figure 71. Same as Supplementary Figure 18, but showing the time series and TMs of station BOMJ for the period from July 2005 to June 2009.

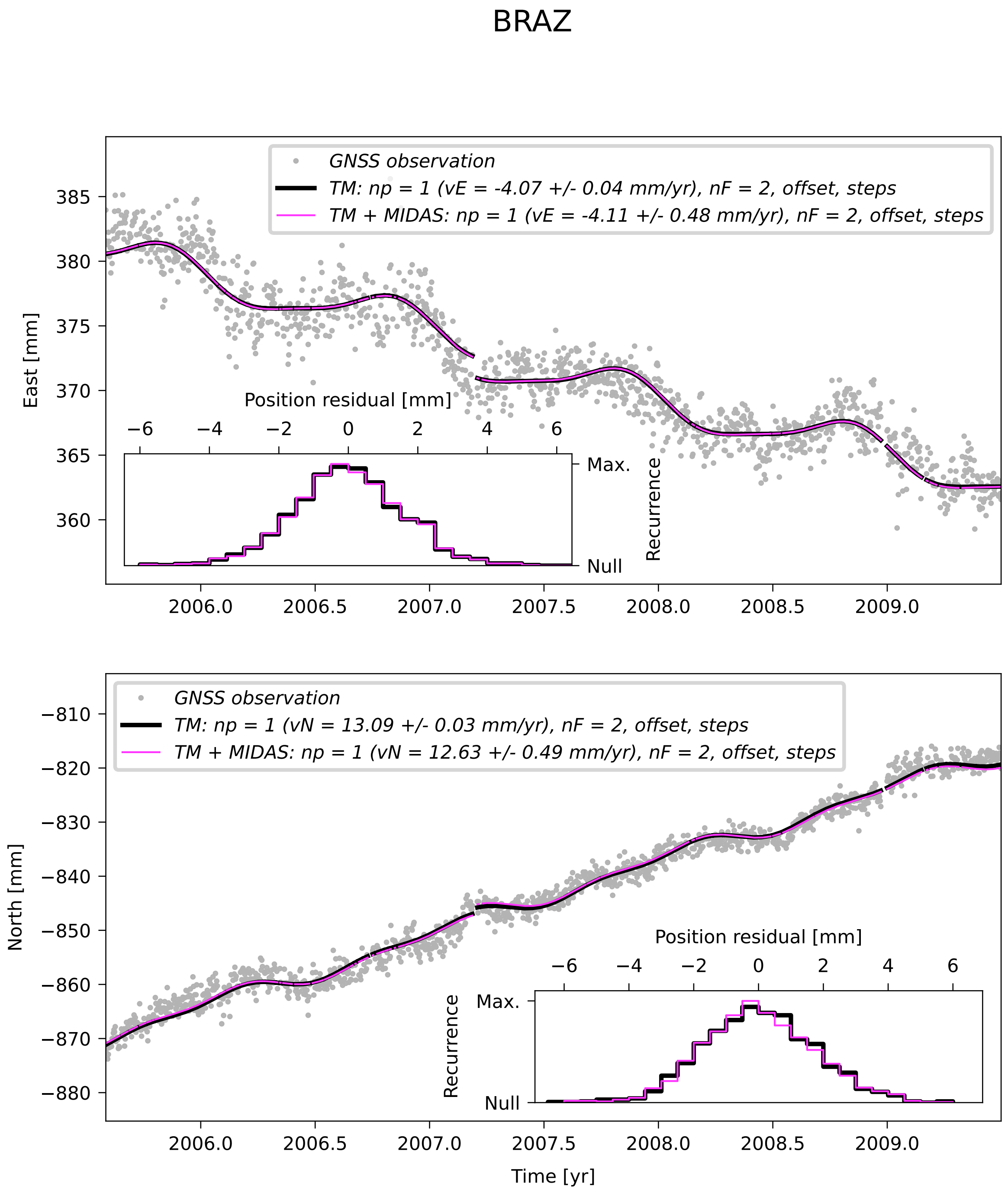

Supplementary Figure 72. Same as Supplementary Figure 18, but showing the time series and TMs of station BRAZ for the period from July 2005 to June 2009.

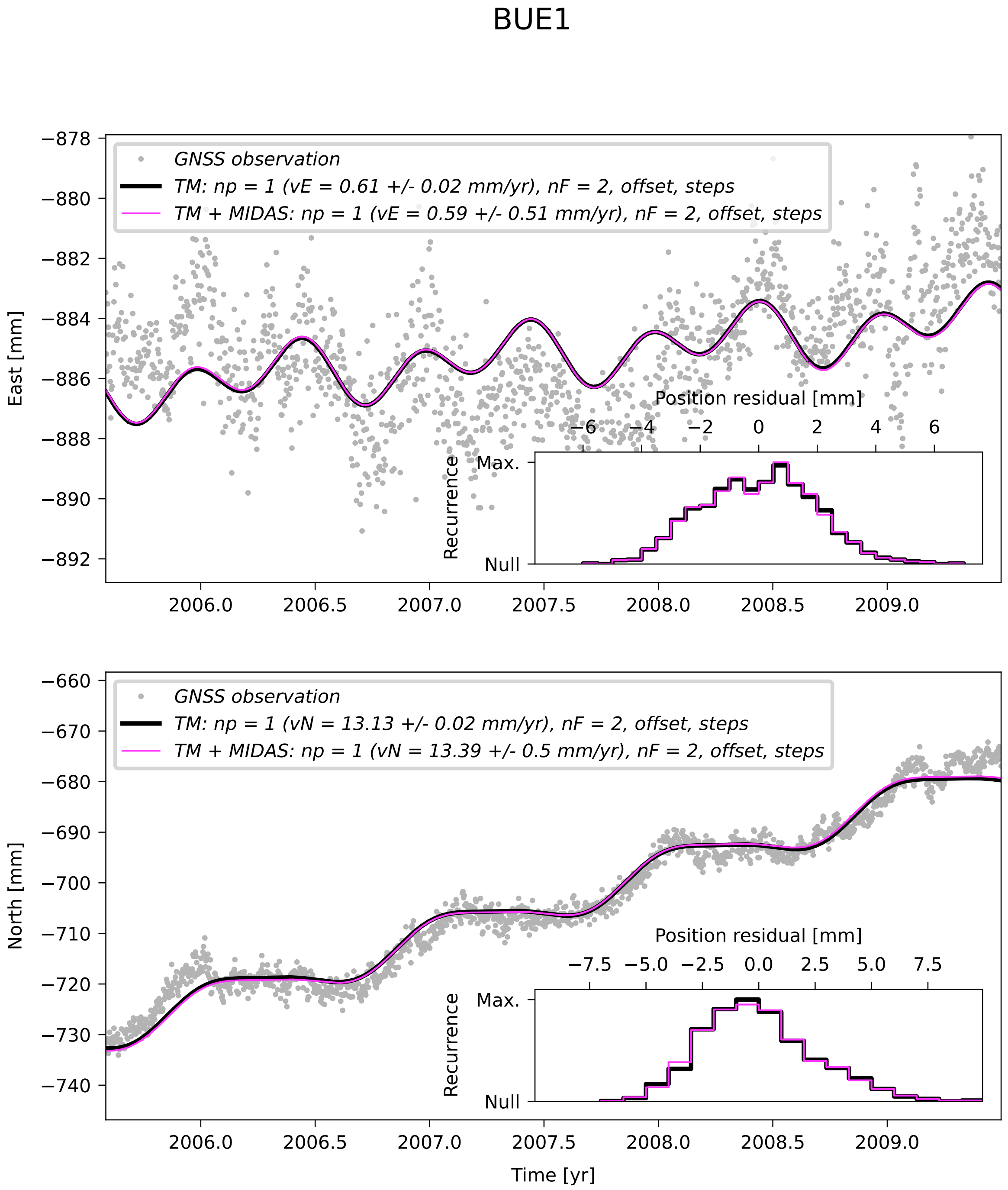

Supplementary Figure 73. Same as Supplementary Figure 18, but showing the time series and TMs of station BUE1 for the period from July 2005 to June 2009. Please note that this site has not been used to constrain the Euler vector for this time period. Instead, it has been excluded due to the *a posteriori* analysis of the velocity residuals (see Supp. Figure 14).

CHPI

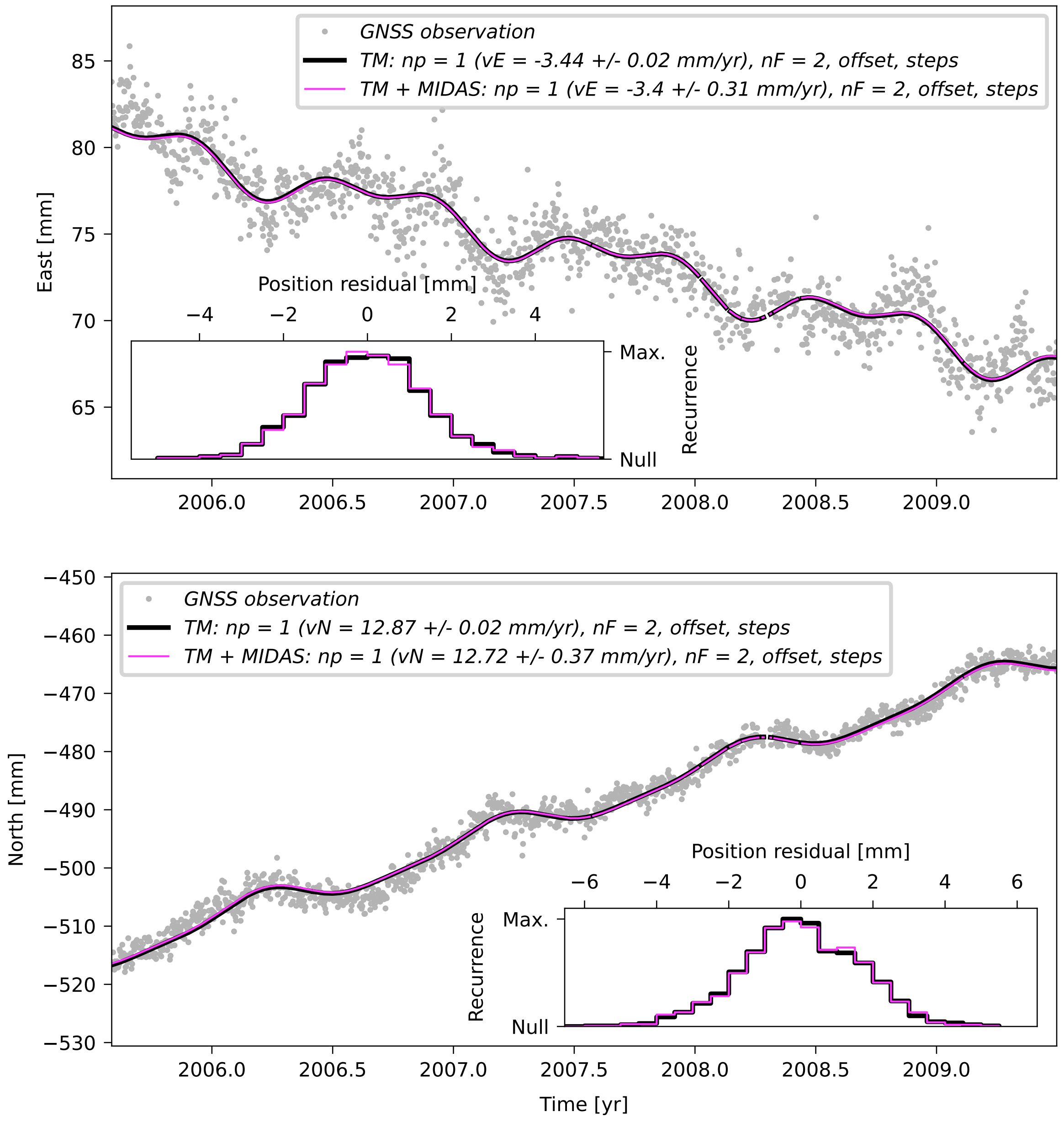

Supplementary Figure 74. Same as Supplementary Figure 18, but showing the time series and TMs of station CHPI for the period from July 2005 to June 2009.

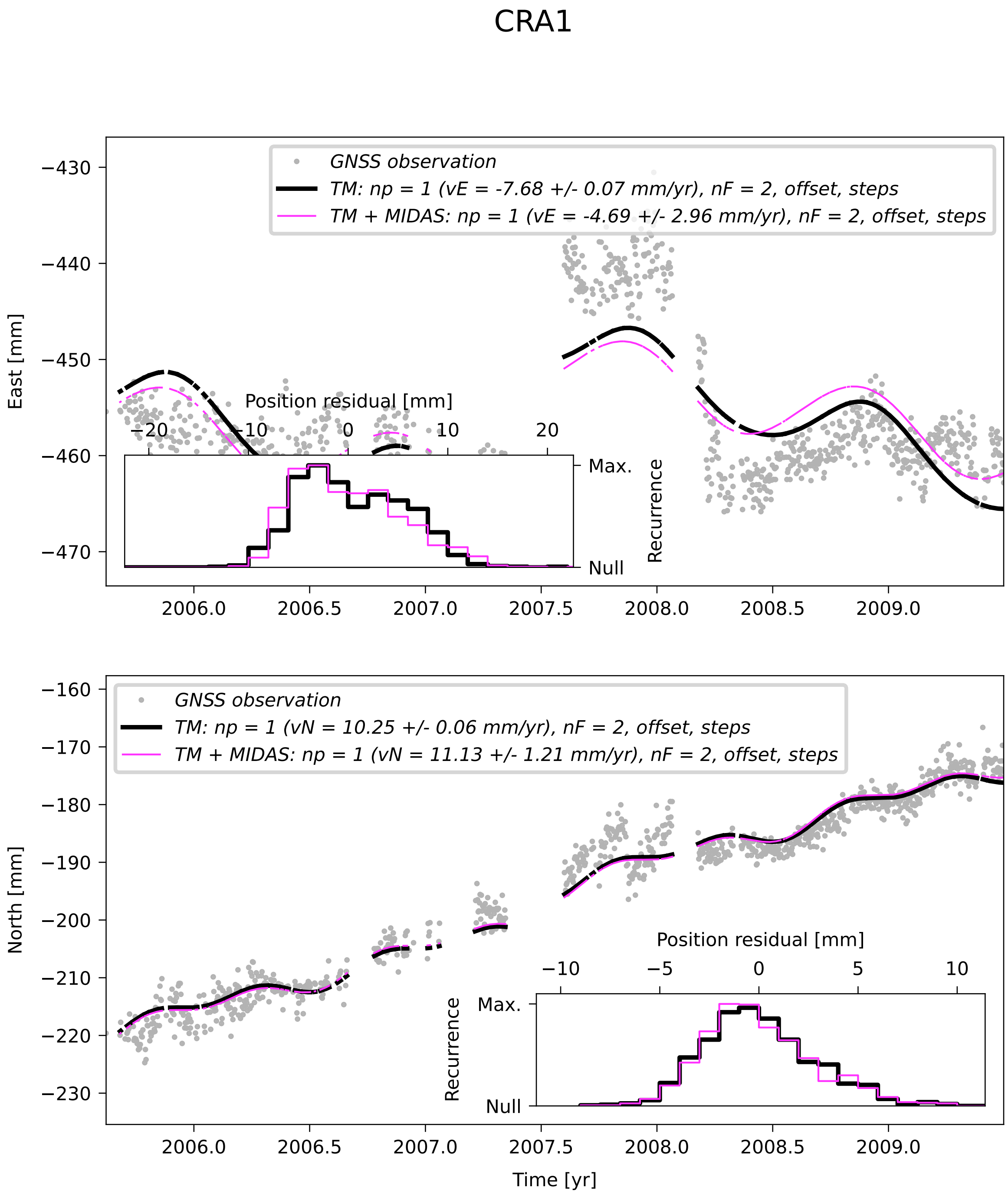

Supplementary Figure 75. Same as Supplementary Figure 18, but showing the time series and TMs of station CRA1 for the period from July 2005 to June 2009.

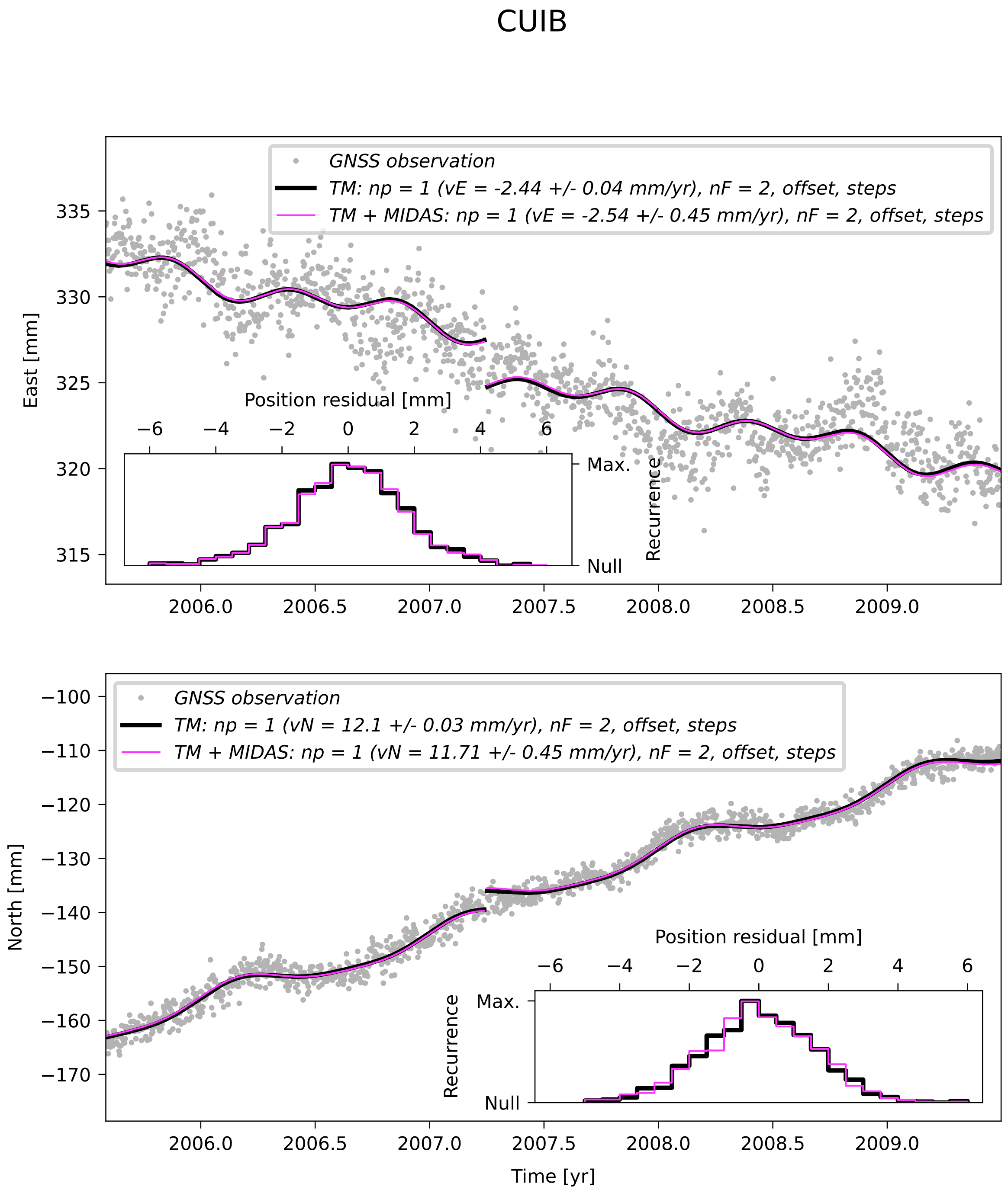

Supplementary Figure 76. Same as Supplementary Figure 18, but showing the time series and TMs of station CUIB for the period from July 2005 to June 2009.

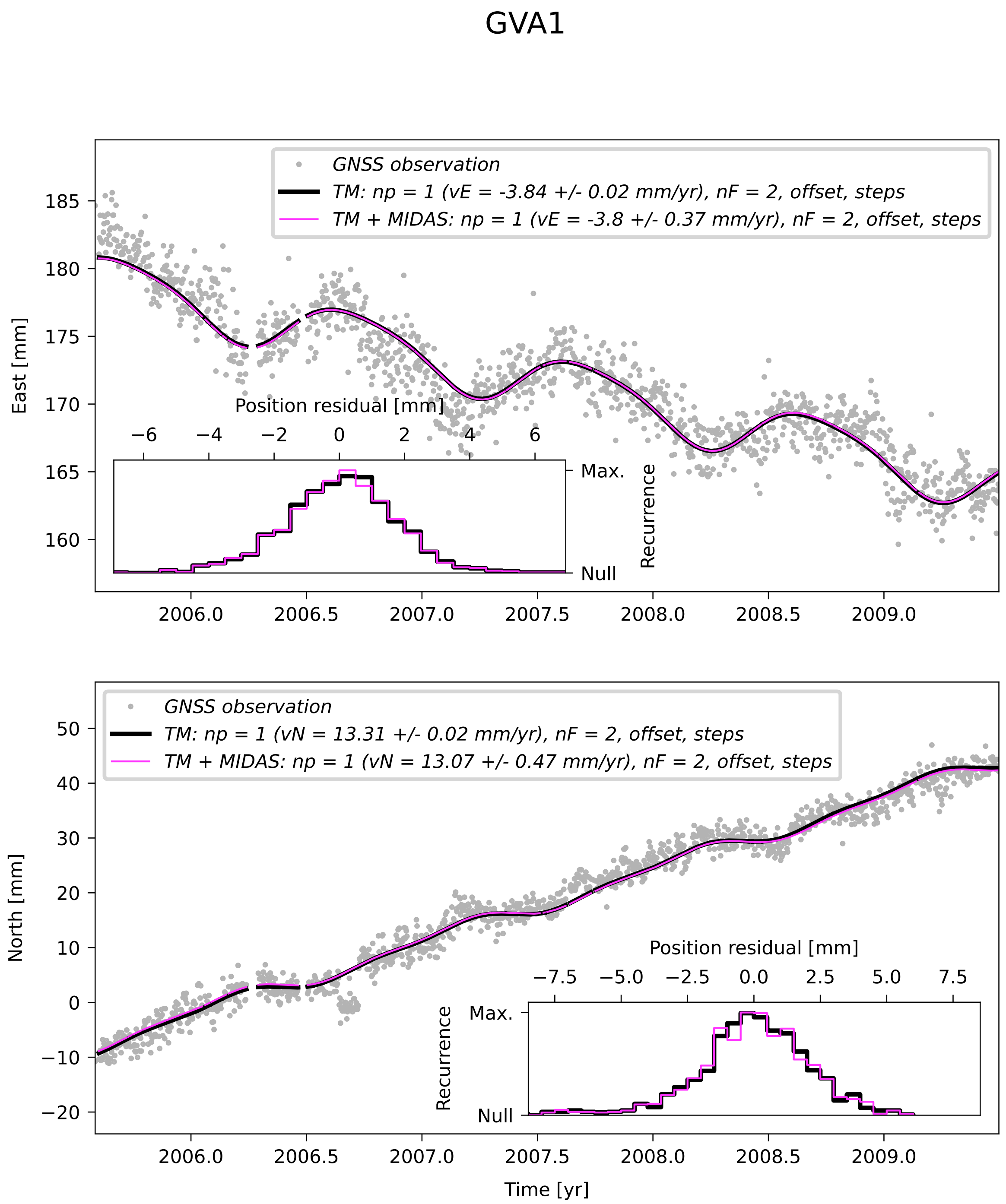

Supplementary Figure 77. Same as Supplementary Figure 18, but showing the time series and TMs of station GVA1 for the period from July 2005 to June 2009.

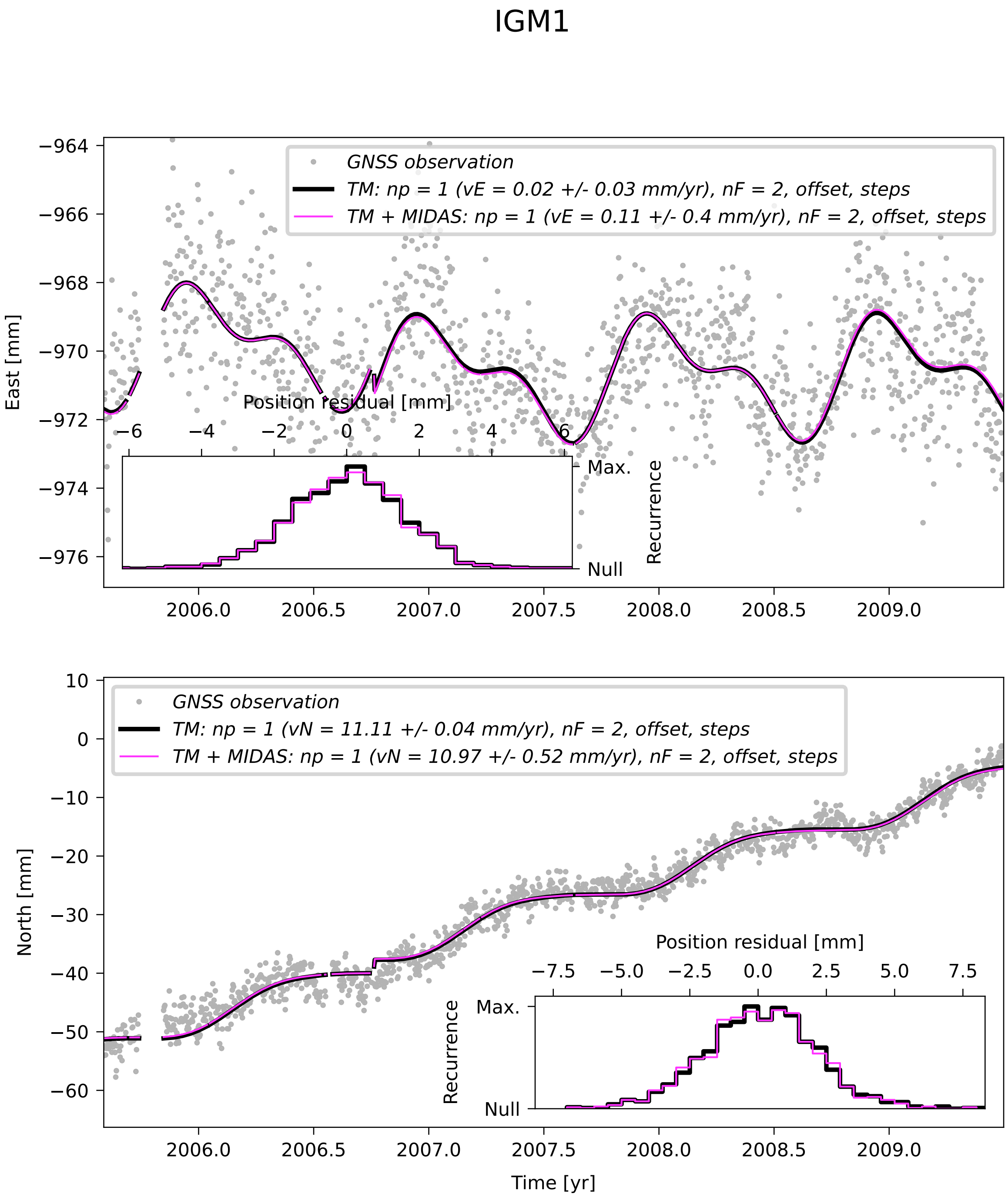

Supplementary Figure 78. Same as Supplementary Figure 18, but showing the time series and TMs of station IGM1 for the period from July 2005 to June 2009.

IMPZ

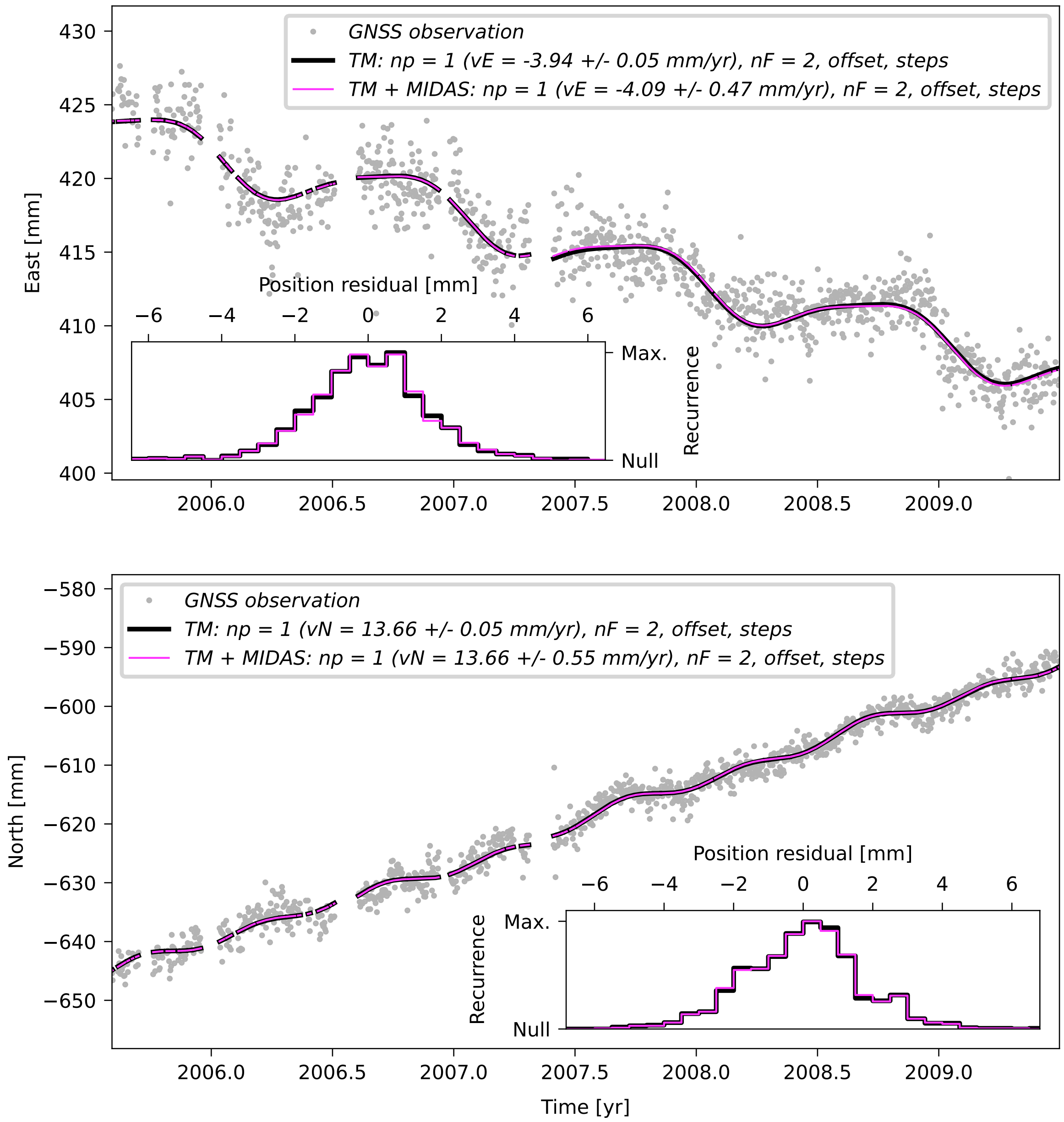

Supplementary Figure 79. Same as Supplementary Figure 18, but showing the time series and TMs of station IMPZ for the period from July 2005 to June 2009.

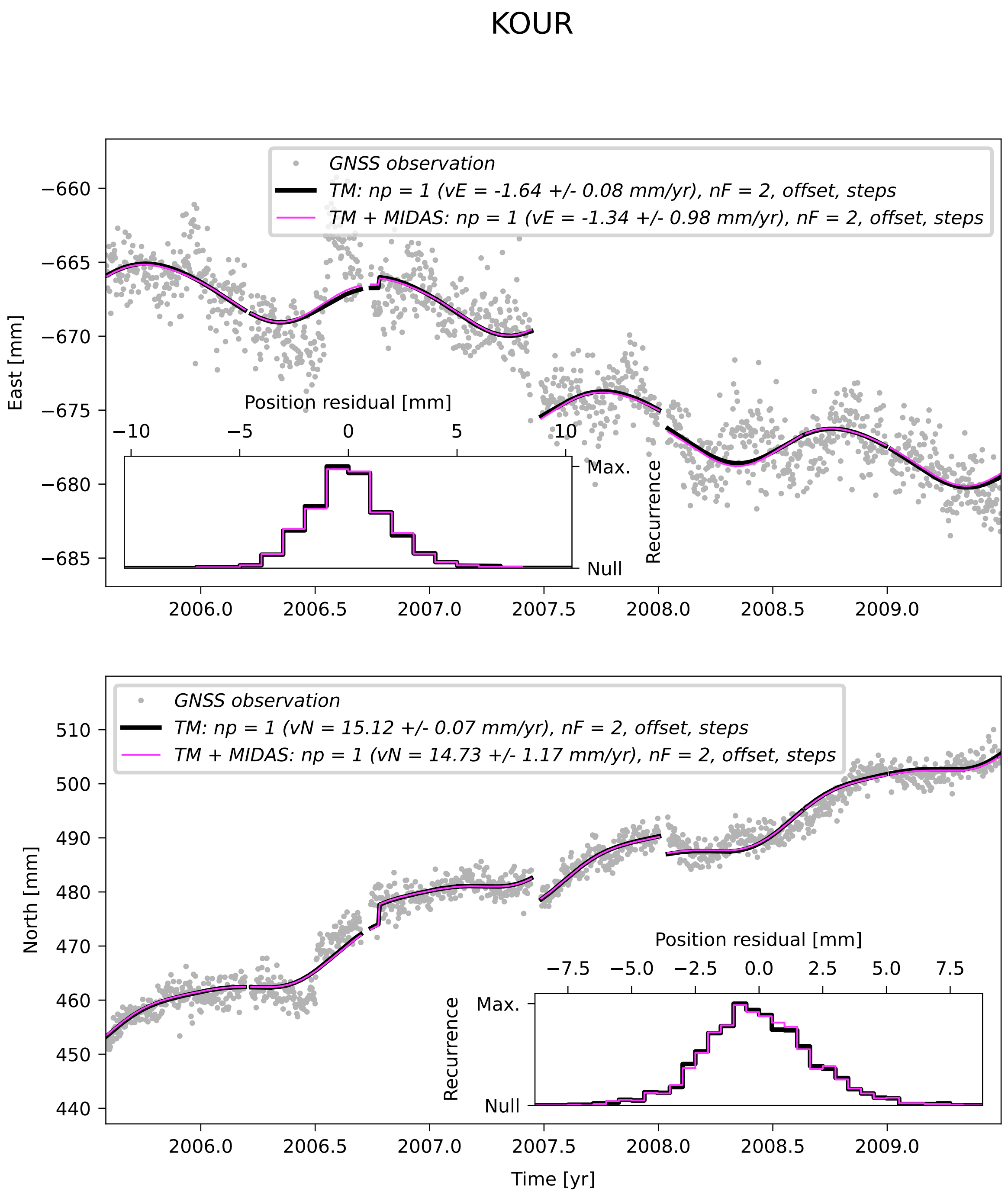

Supplementary Figure 80. Same as Supplementary Figure 18, but showing the time series and TMs of station KOUR for the period from July 2005 to June 2009. Please note that this site has not been used to constrain the Euler vector for this time period. Instead, it has been excluded due to the *a posteriori* analysis of the velocity residuals (see Supp. Figure 14).

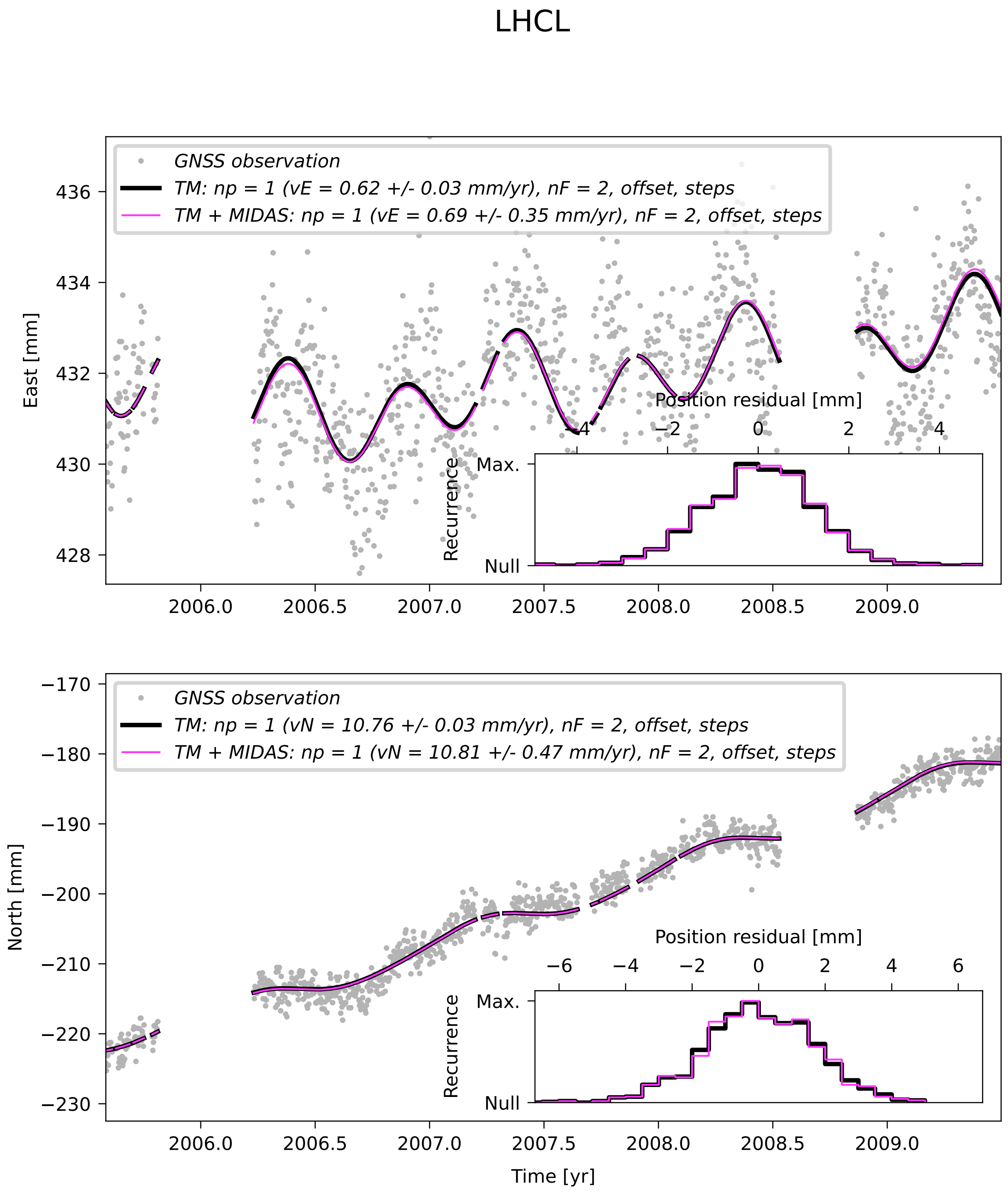

Supplementary Figure 81. Same as Supplementary Figure 18, but showing the time series and TMs of station LHCL for the period from July 2005 to June 2009.

LPGS

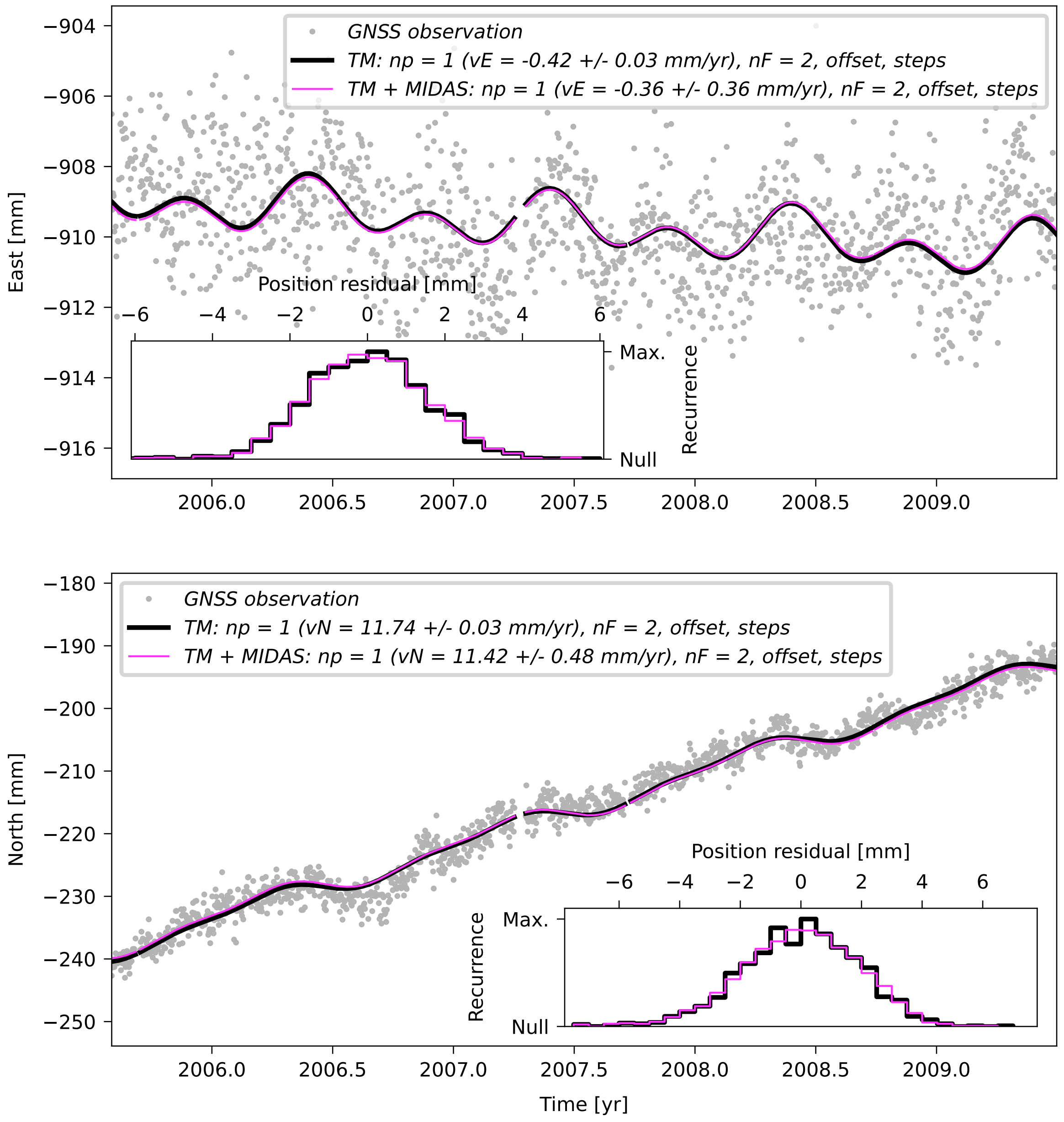

Supplementary Figure 82. Same as Supplementary Figure 18, but showing the time series and TMs of station LPGS for the period from July 2005 to June 2009.

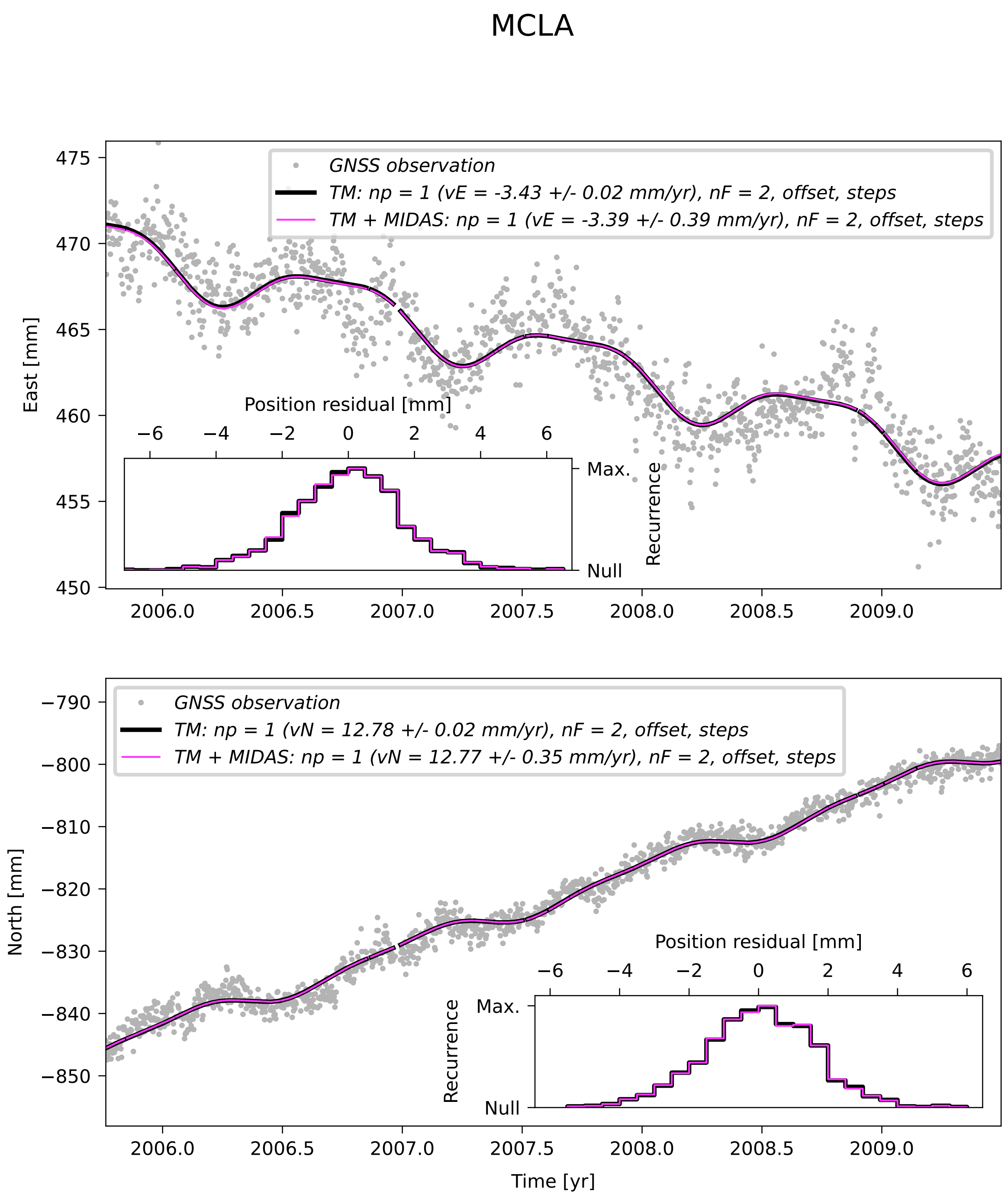

Supplementary Figure 83. Same as Supplementary Figure 18, but showing the time series and TMs of station MCLA for the period from July 2005 to June 2009.

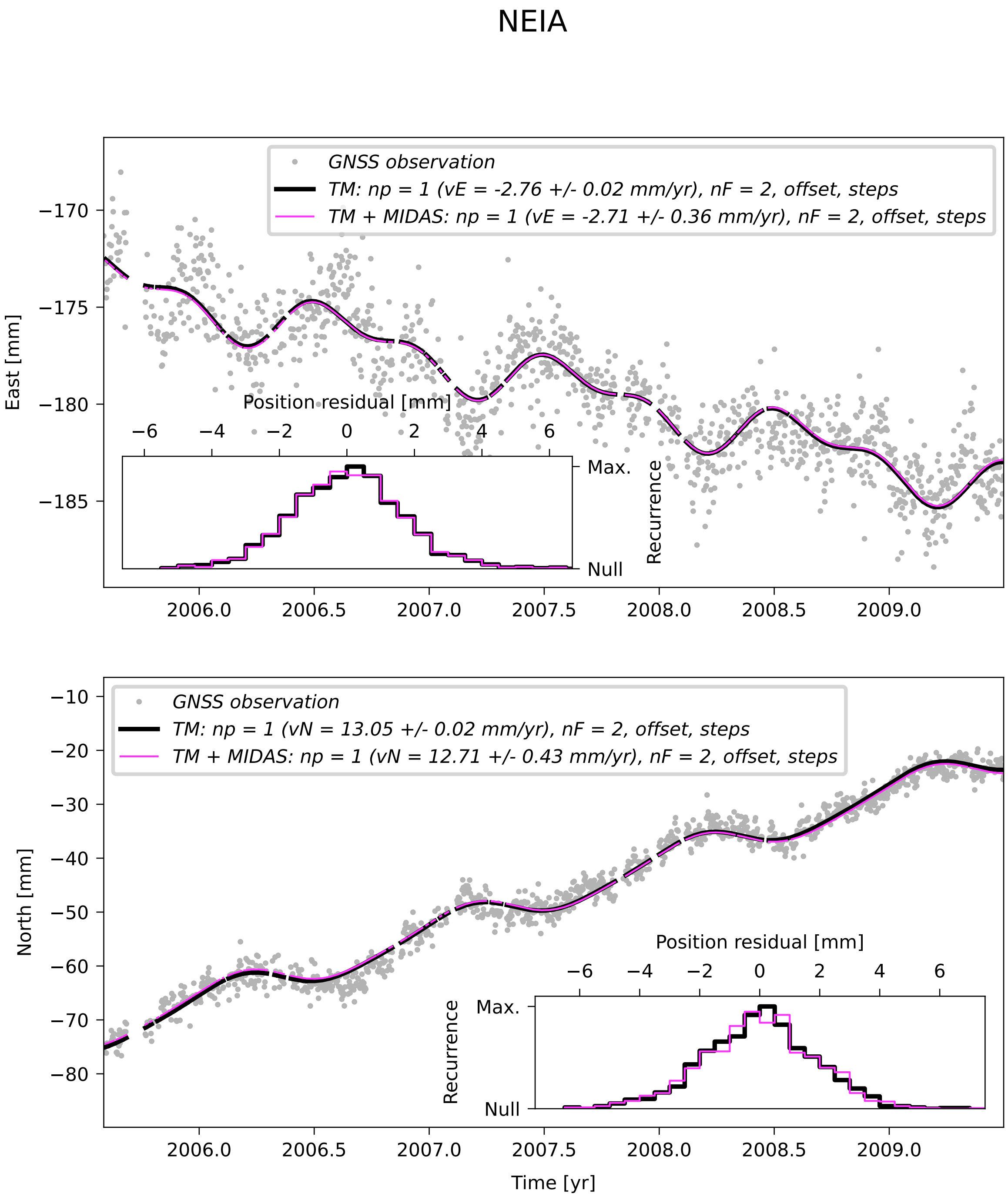

Supplementary Figure 84. Same as Supplementary Figure 18, but showing the time series and TMs of station NEIA for the period from July 2005 to June 2009.

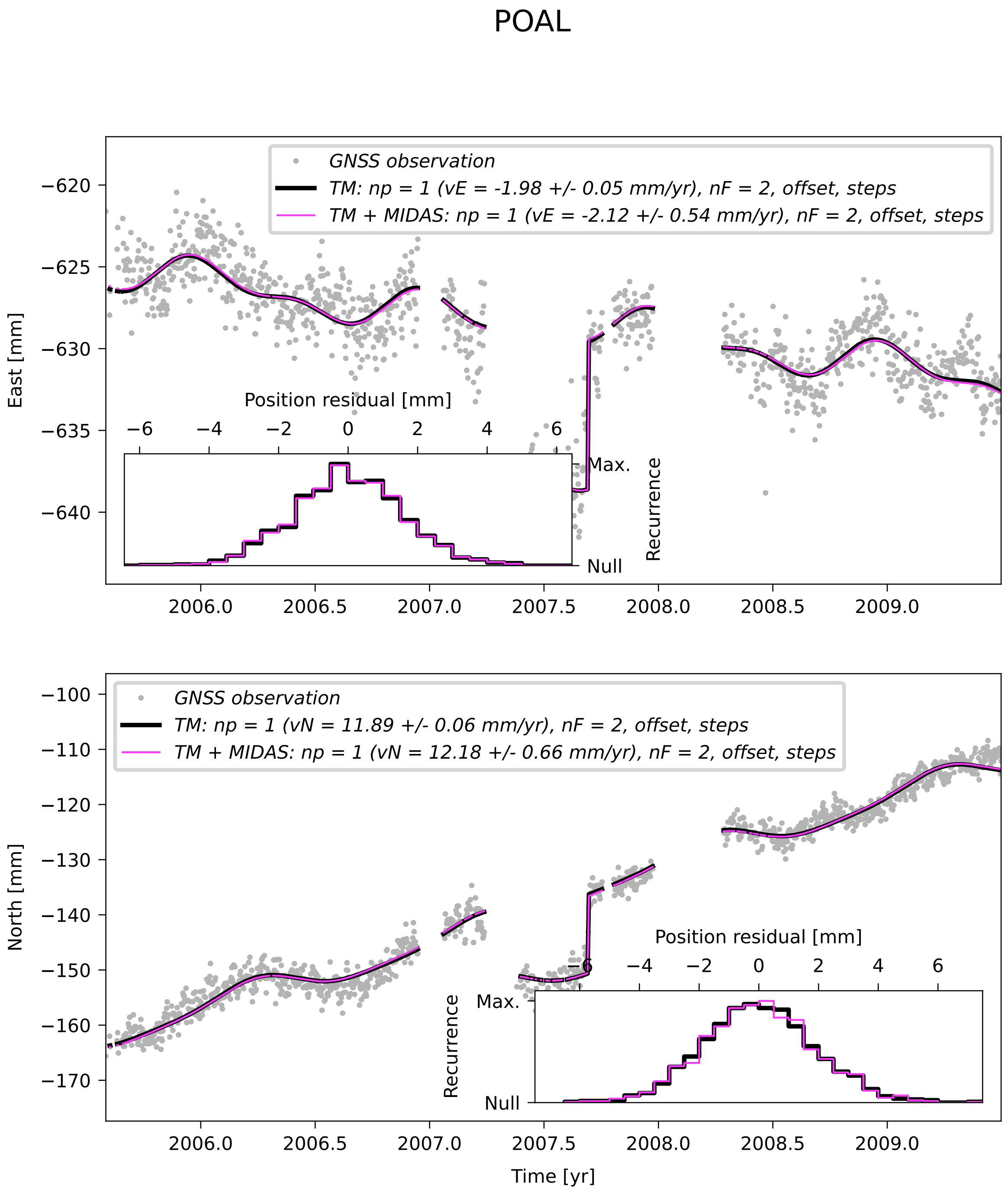

Supplementary Figure 85. Same as Supplementary Figure 18, but showing the time series and TMs of station POAL for the period from July 2005 to June 2009.

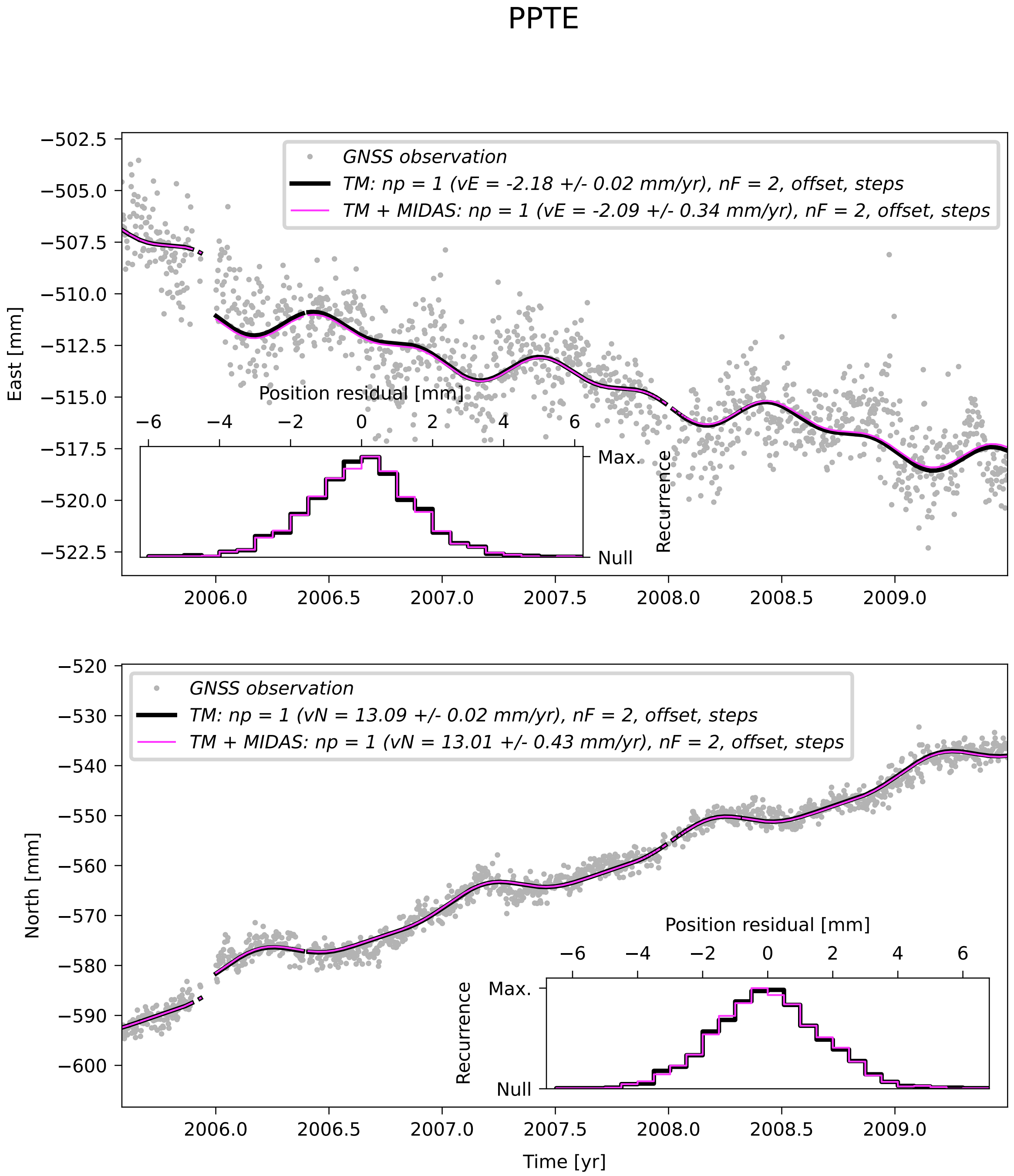

Supplementary Figure 86. Same as Supplementary Figure 18, but showing the time series and TMs of station PPTE for the period from July 2005 to June 2009.

PRMA

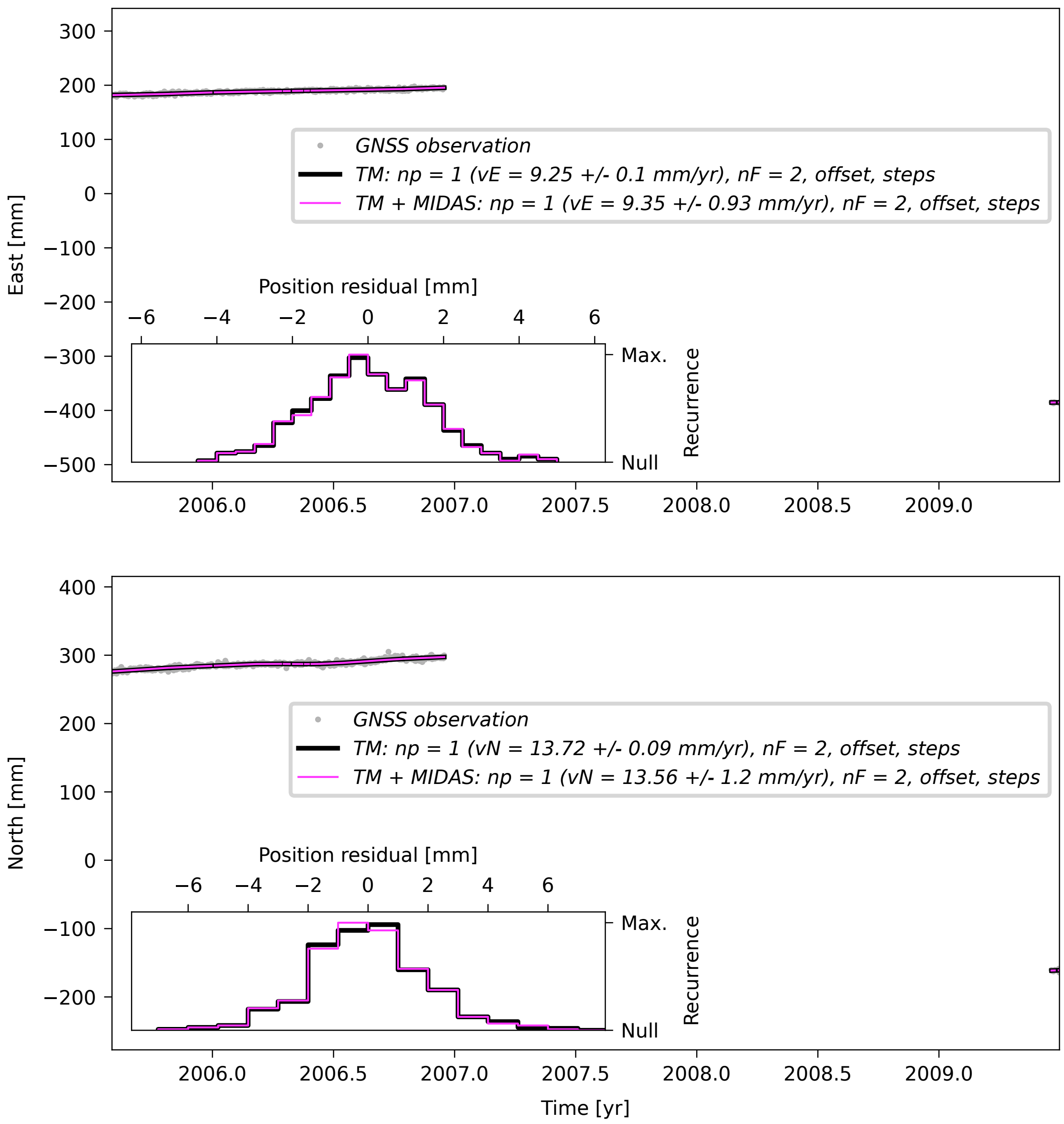

Supplementary Figure 87. Same as Supplementary Figure 18, but showing the time series and TMs of station PRMA for the period from July 2005 to June 2009. Please note that this site has not been used to constrain the Euler vector for this time period. Instead, it has been excluded due to the *a posteriori* analysis of the velocity residuals (see Supp. Figure 14).

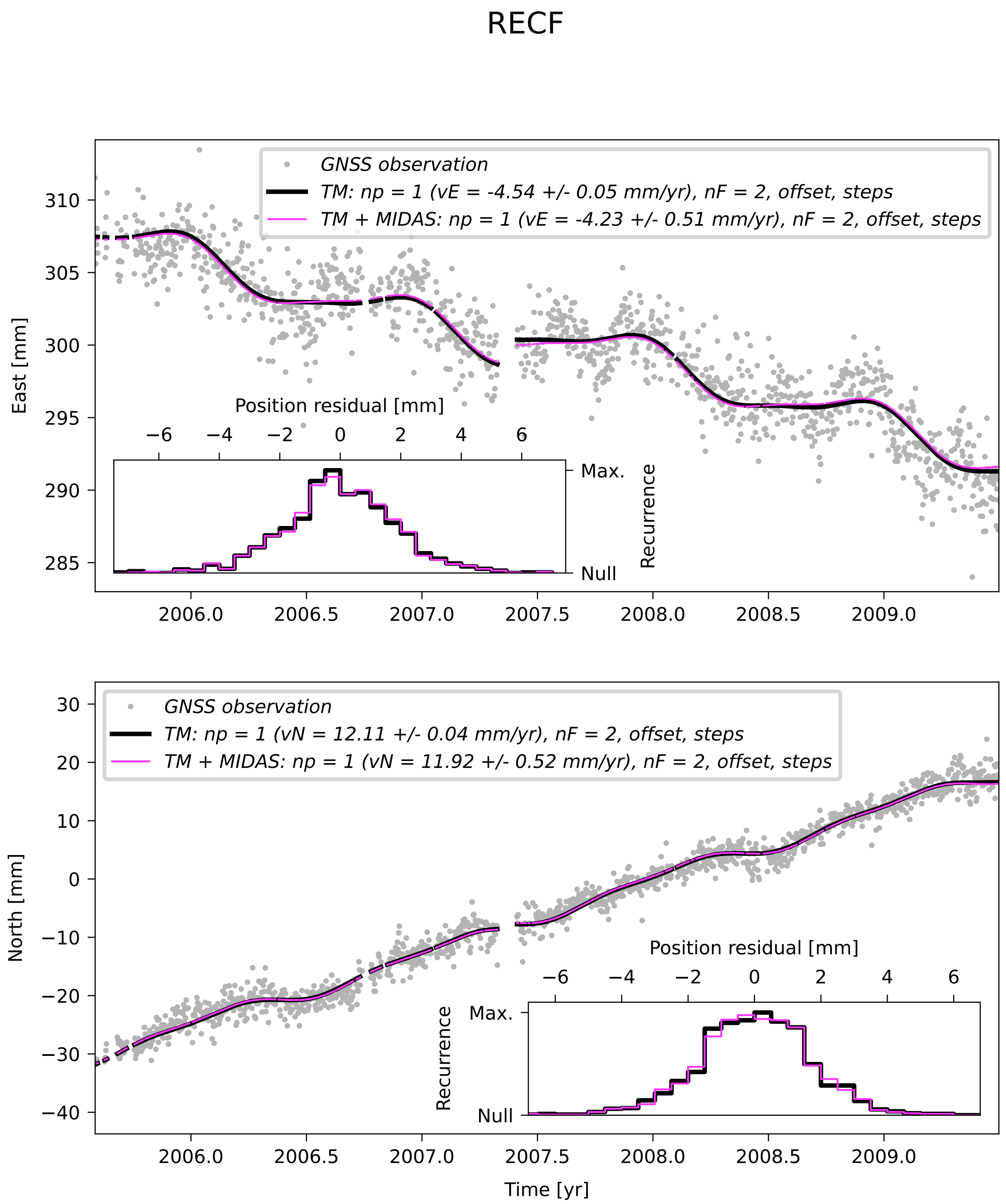

Supplementary Figure 88. Same as Supplementary Figure 18, but showing the time series and TMs of station RECF for the period from July 2005 to June 2009.

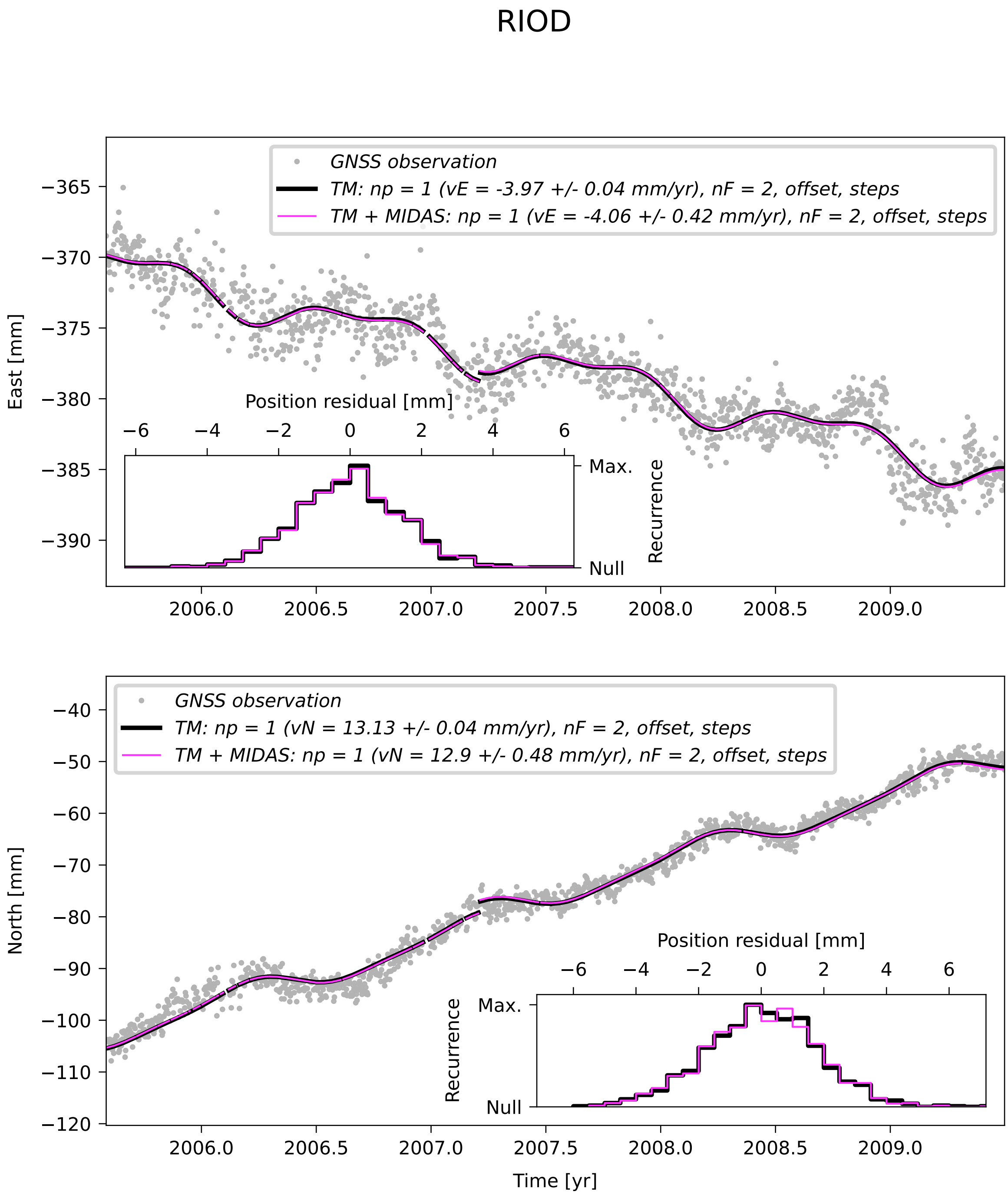

Supplementary Figure 89. Same as Supplementary Figure 18, but showing the time series and TMs of station RIOD for the period from July 2005 to June 2009.

RWSN

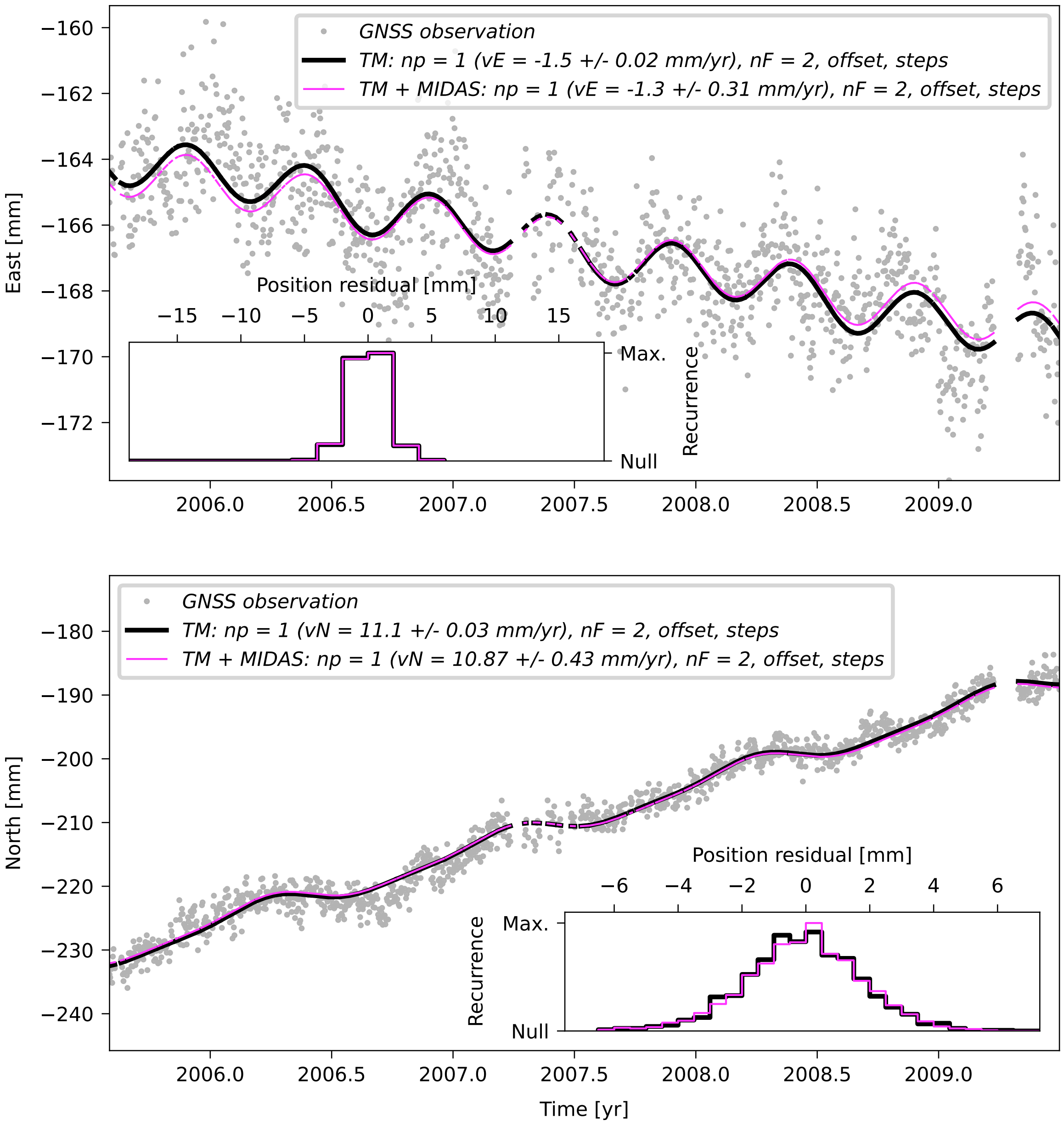

Supplementary Figure 90. Same as Supplementary Figure 18, but showing the time series and TMs of station RWSN for the period from July 2005 to June 2009. Please note that this site has not been used to constrain the Euler vector for this time period. Instead, it has been excluded due to the *a posteriori* analysis of the velocity residuals (see Supp. Figure 14).

SMAR

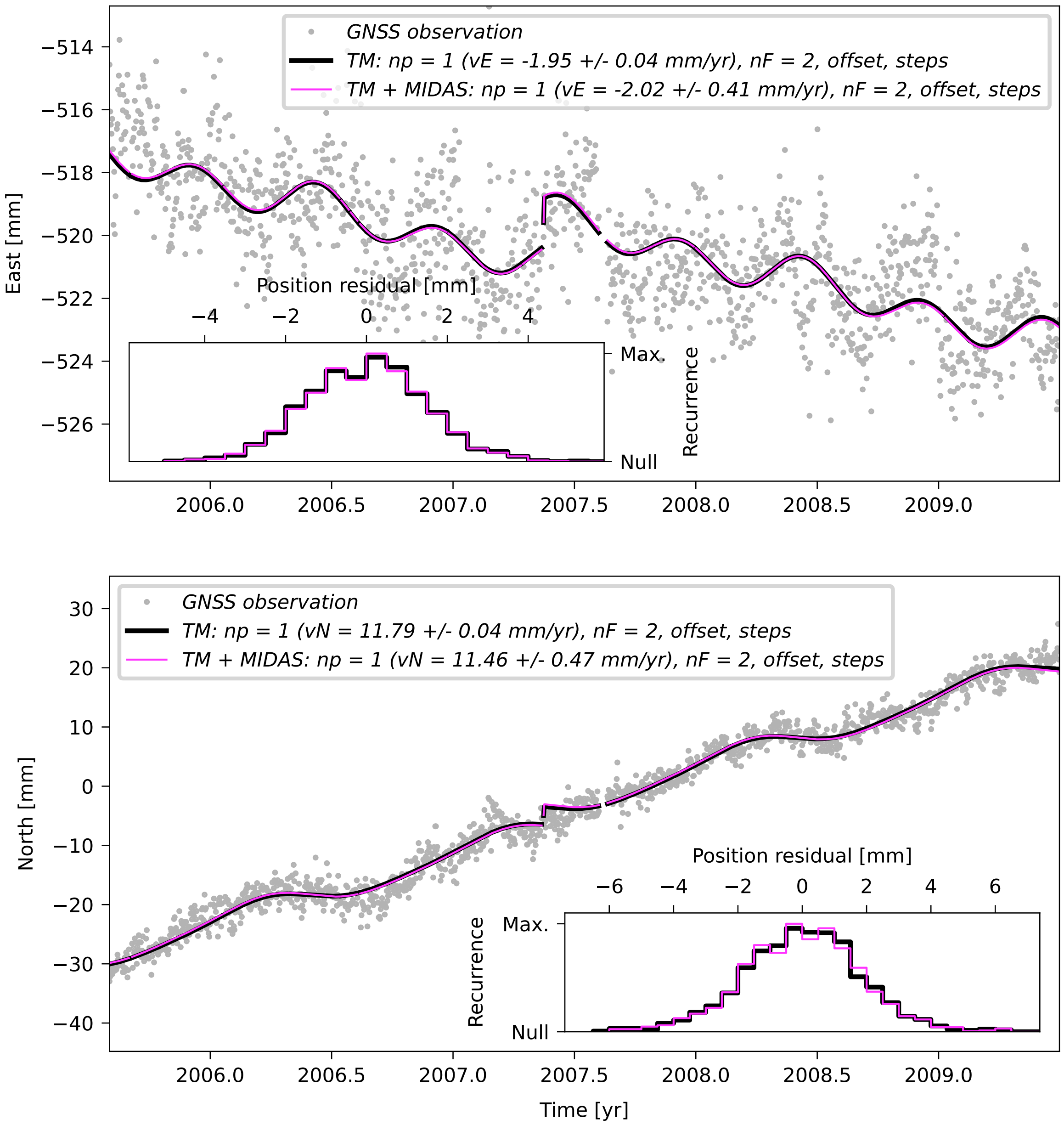

Supplementary Figure 91. Same as Supplementary Figure 18, but showing the time series and TMs of station SMAR for the period from July 2005 to June 2009.

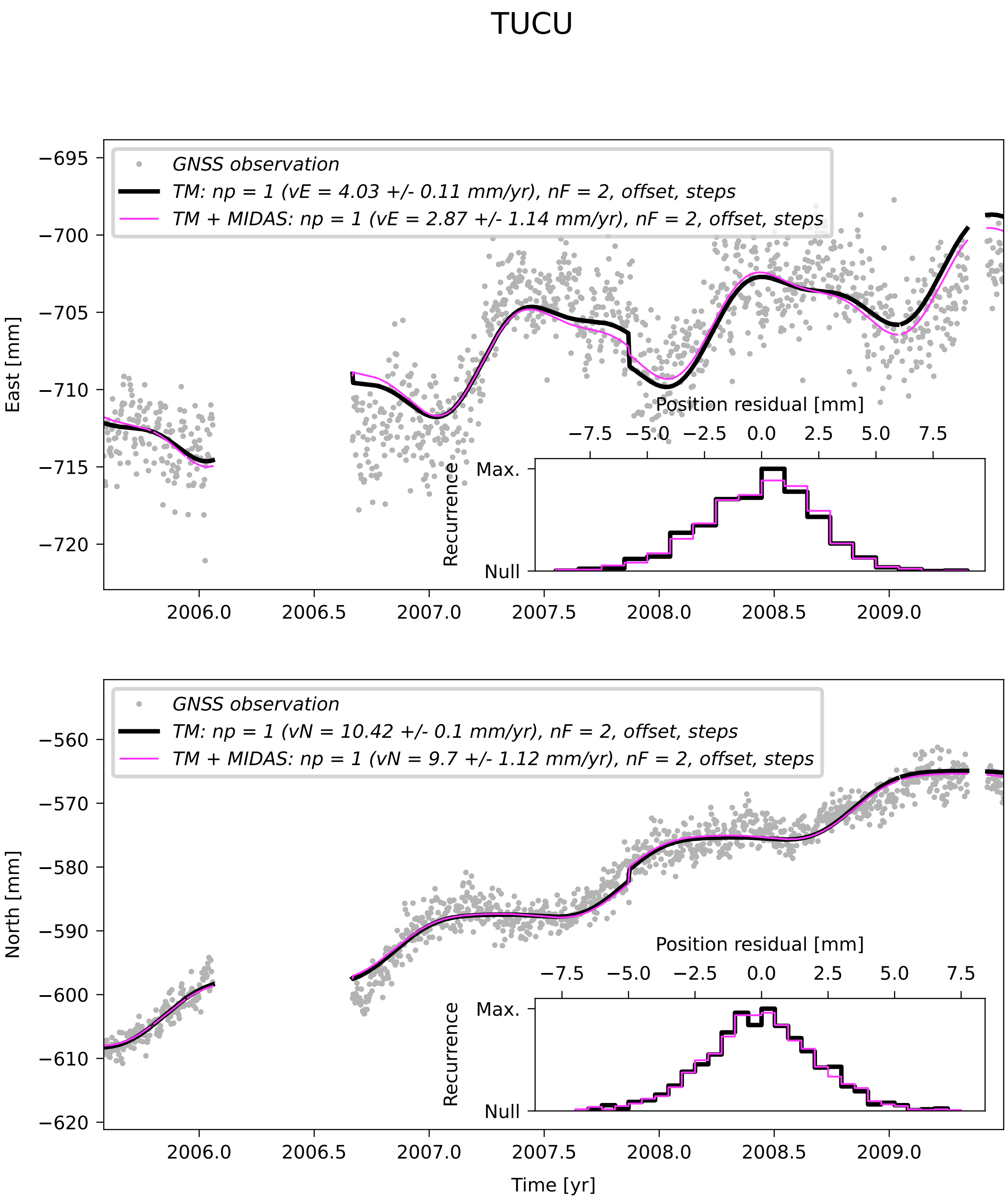

Supplementary Figure 92. Same as Supplementary Figure 18, but showing the time series and TMs of station TUCU for the period from July 2005 to June 2009. Please note that this site has not been used to constrain the Euler vector for this time period. Instead, it has been excluded due to the *a posteriori* analysis of the velocity residuals (see Supp. Figure 14).

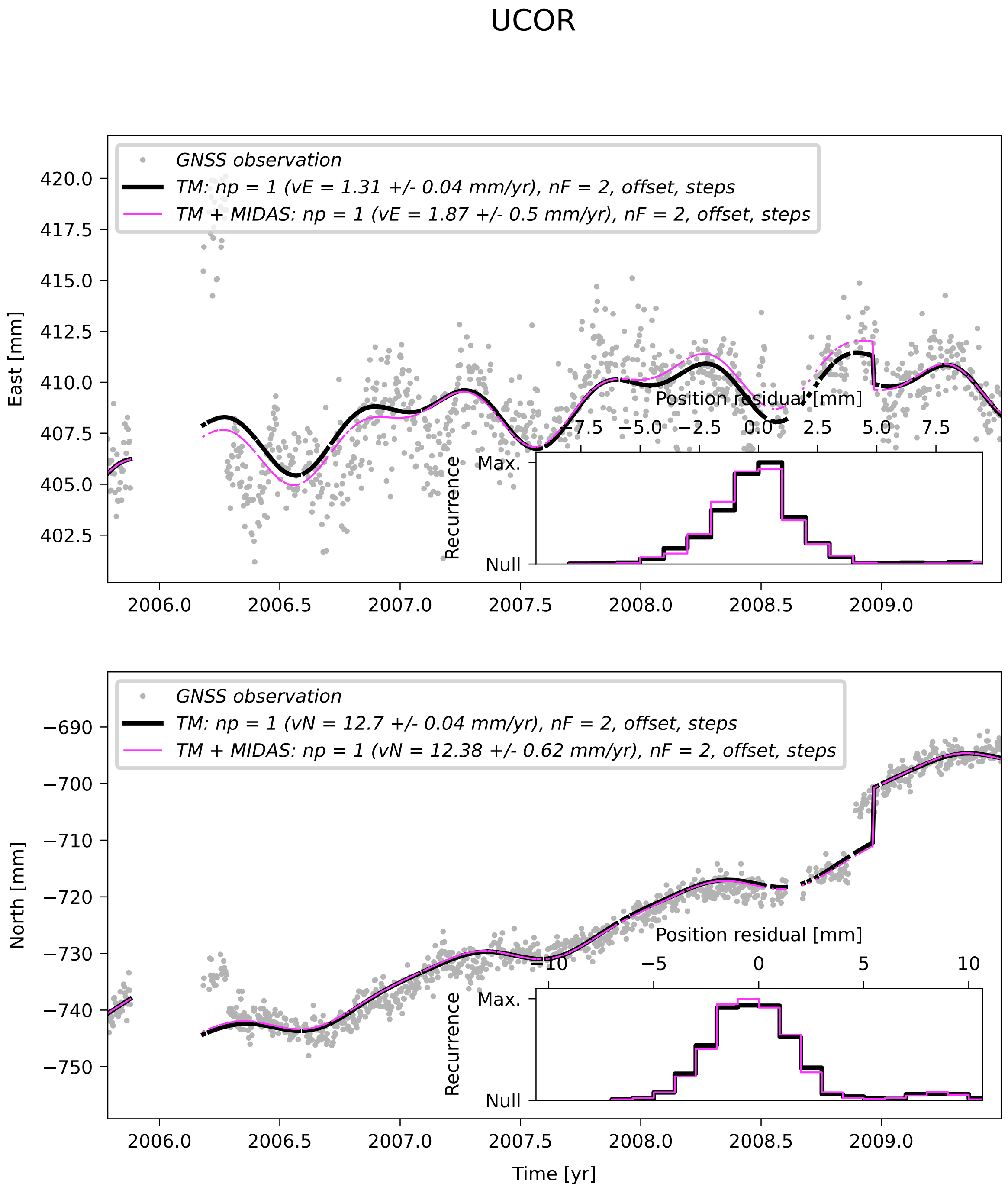

Supplementary Figure 93. Same as Supplementary Figure 18, but showing the time series and TMs of station UCOR for the period from July 2005 to June 2009. Please note that this site has not been used to constrain the Euler vector for this time period. Instead, it has been excluded due to the *a posteriori* analysis of the velocity residuals (see Supp. Figure 14).

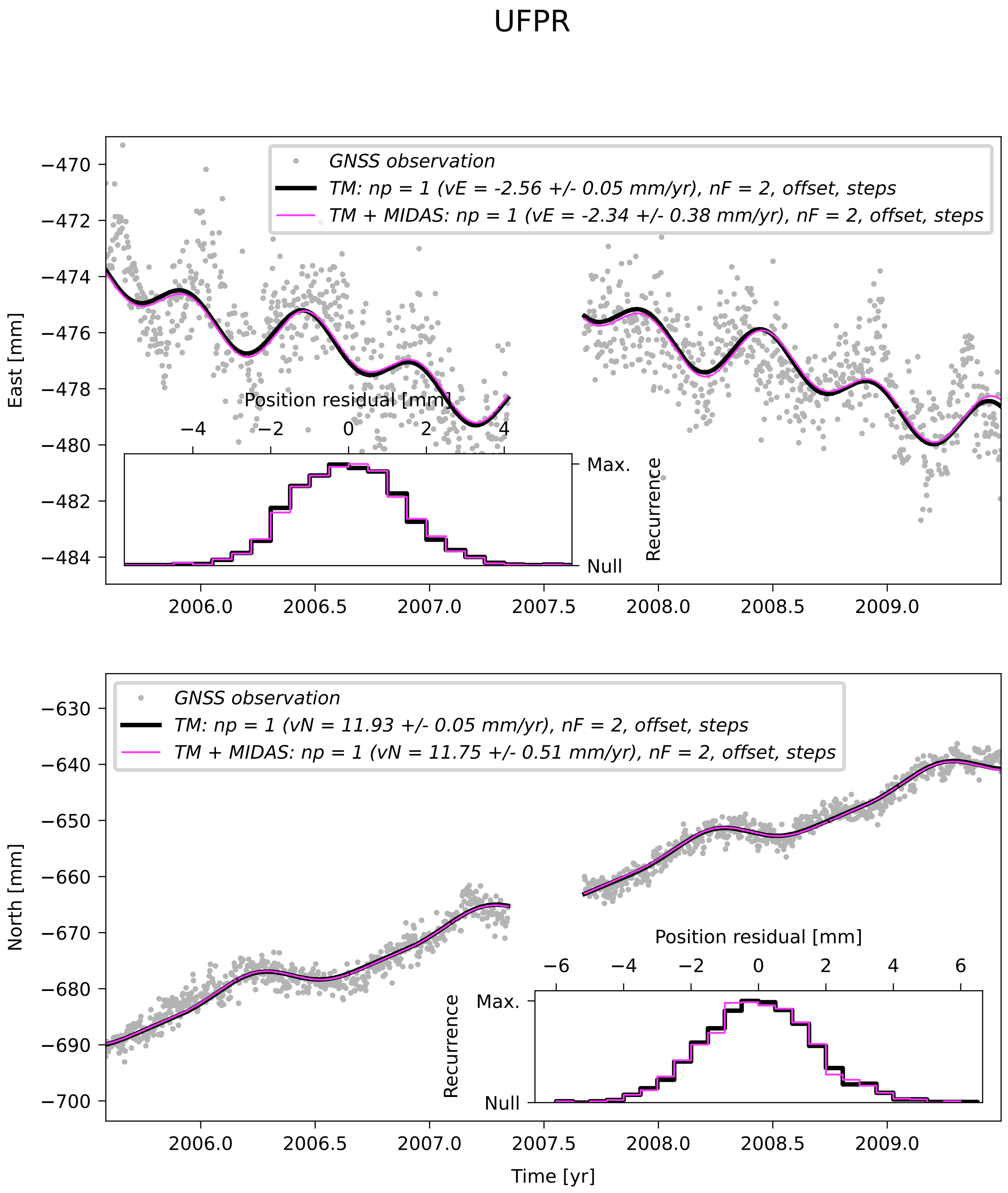

Supplementary Figure 94. Same as Supplementary Figure 18, but showing the time series and TMs of station UFPR for the period from July 2005 to June 2009.

UNSA

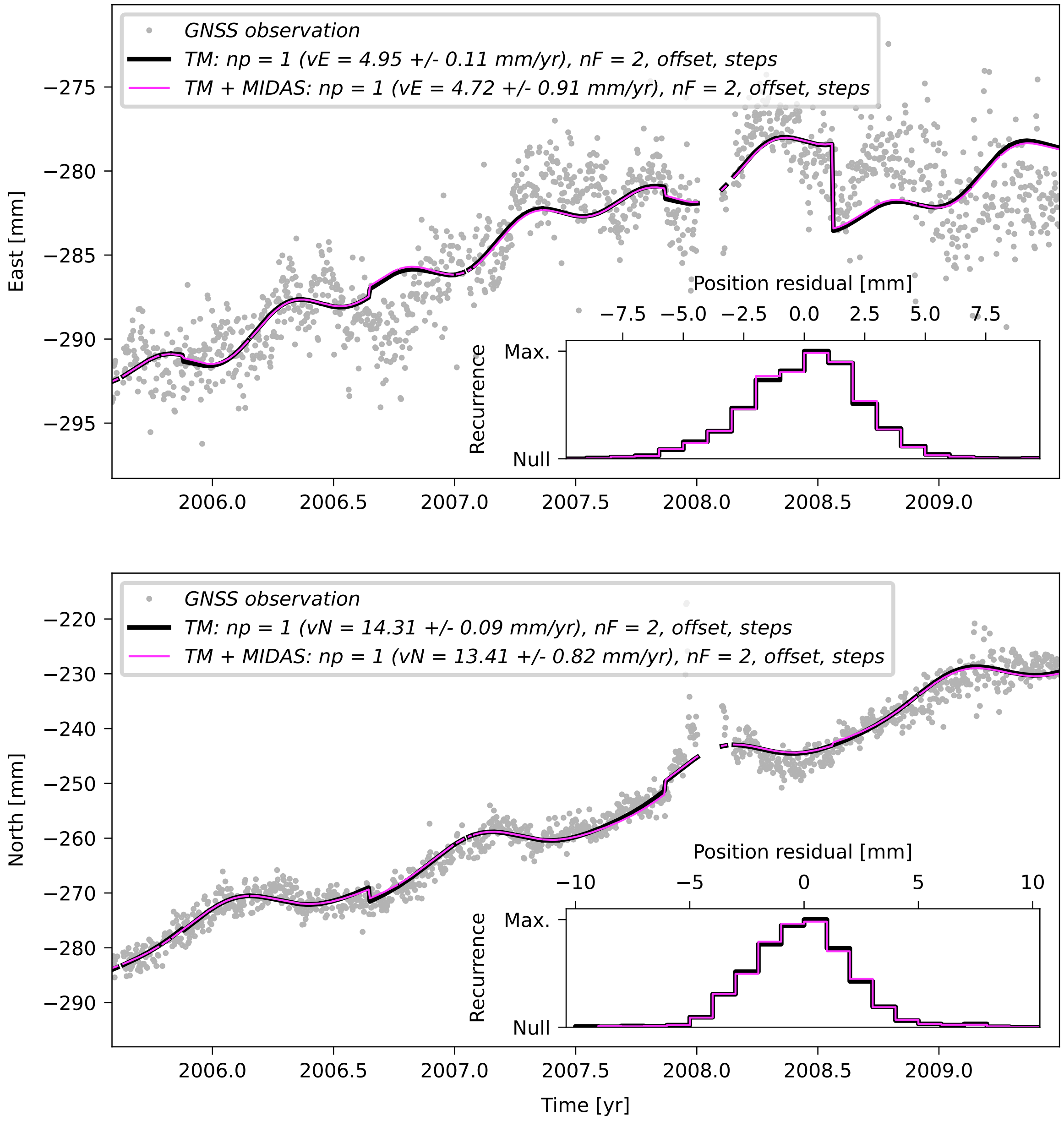

Supplementary Figure 95. Same as Supplementary Figure 18, but showing the time series and TMs of station UNSA for the period from July 2005 to June 2009. Please note that this site has not been used to constrain the Euler vector for this time period. Instead, it has been excluded due to the *a posteriori* analysis of the velocity residuals (see Supp. Figure 14).

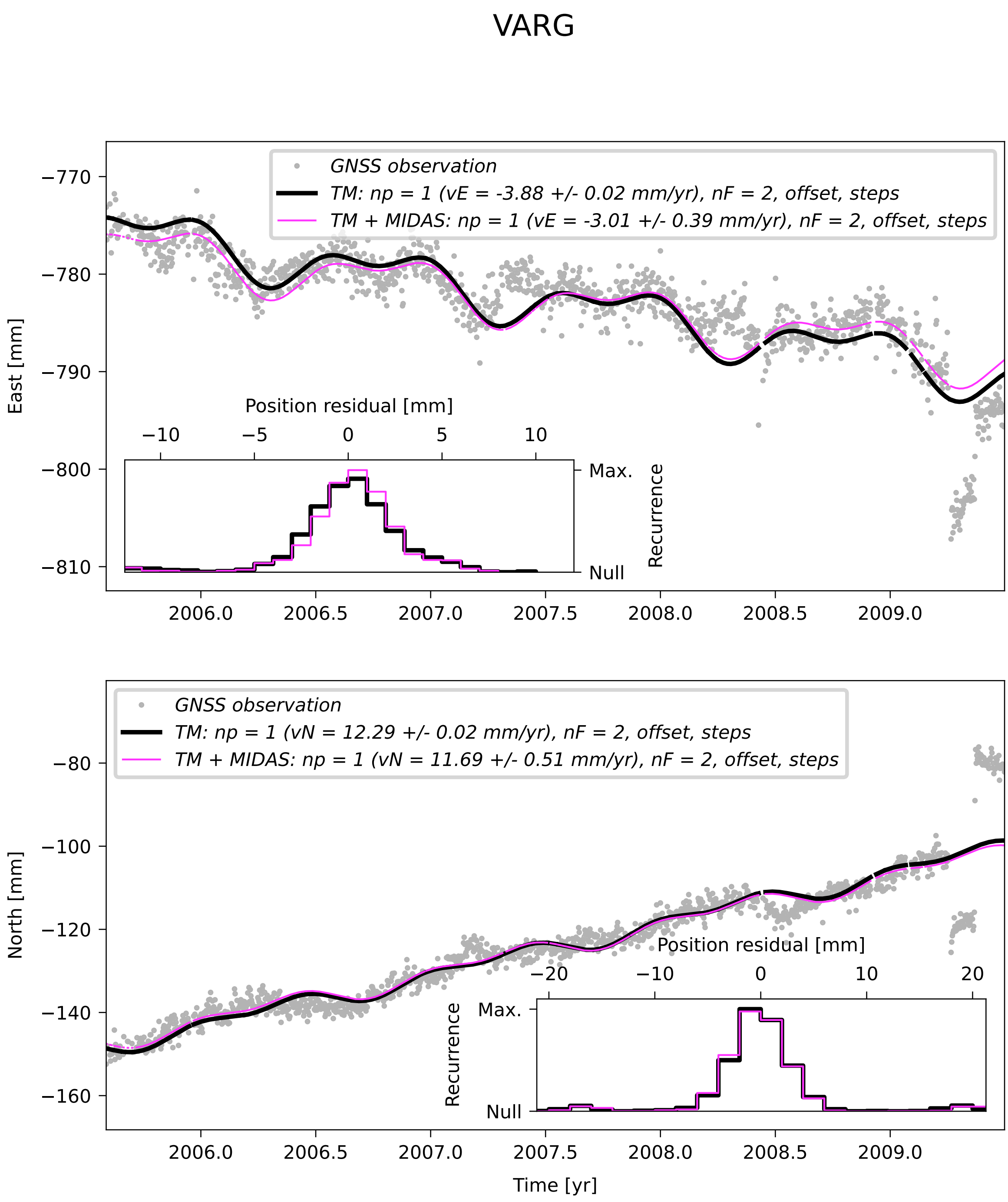

Supplementary Figure 96. Same as Supplementary Figure 18, but showing the time series and TMs of station VARG for the period from July 2005 to June 2009.

VBCA

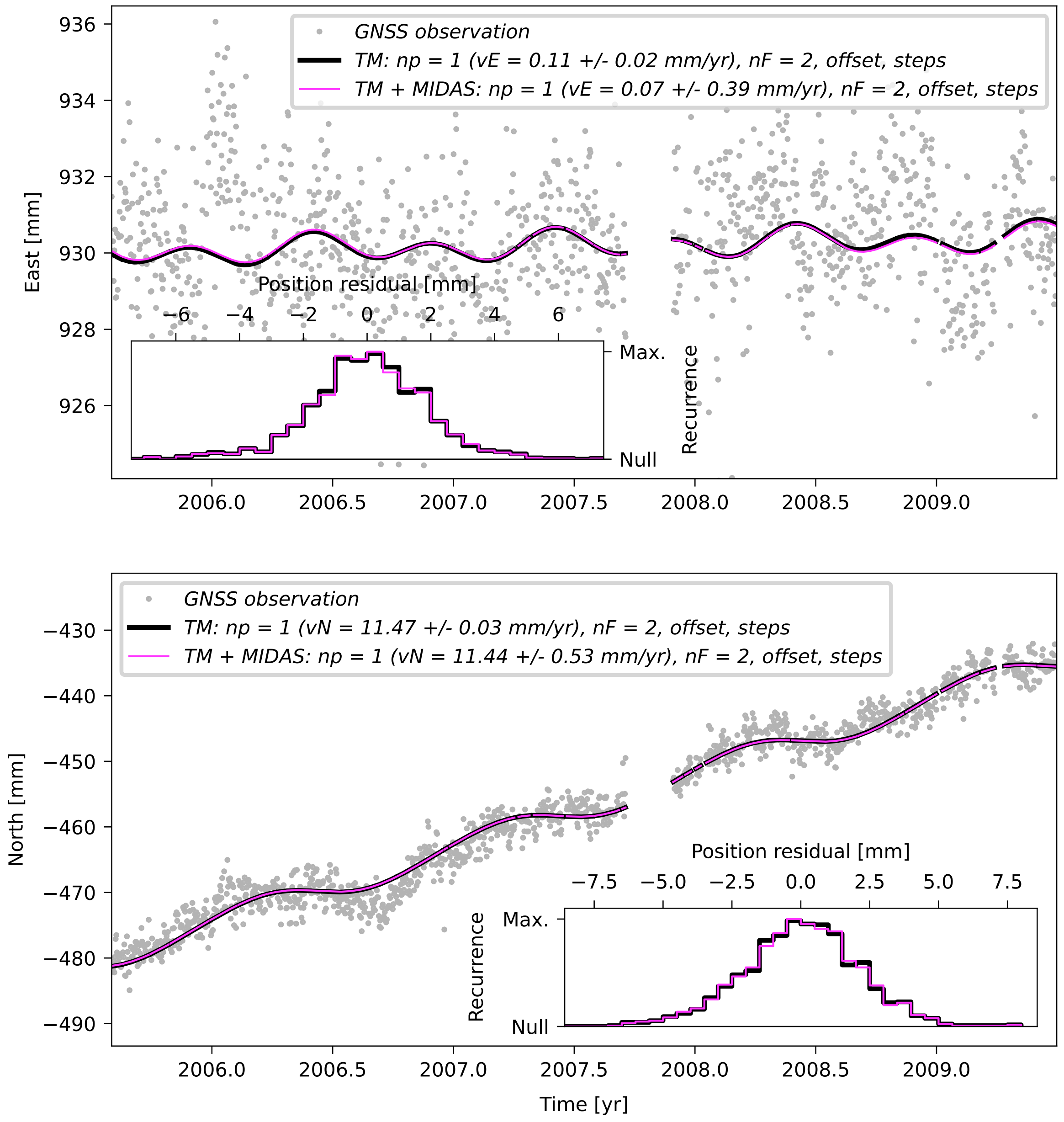

Supplementary Figure 97. Same as Supplementary Figure 18, but showing the time series and TMs of station VBCA for the period from July 2005 to June 2009.

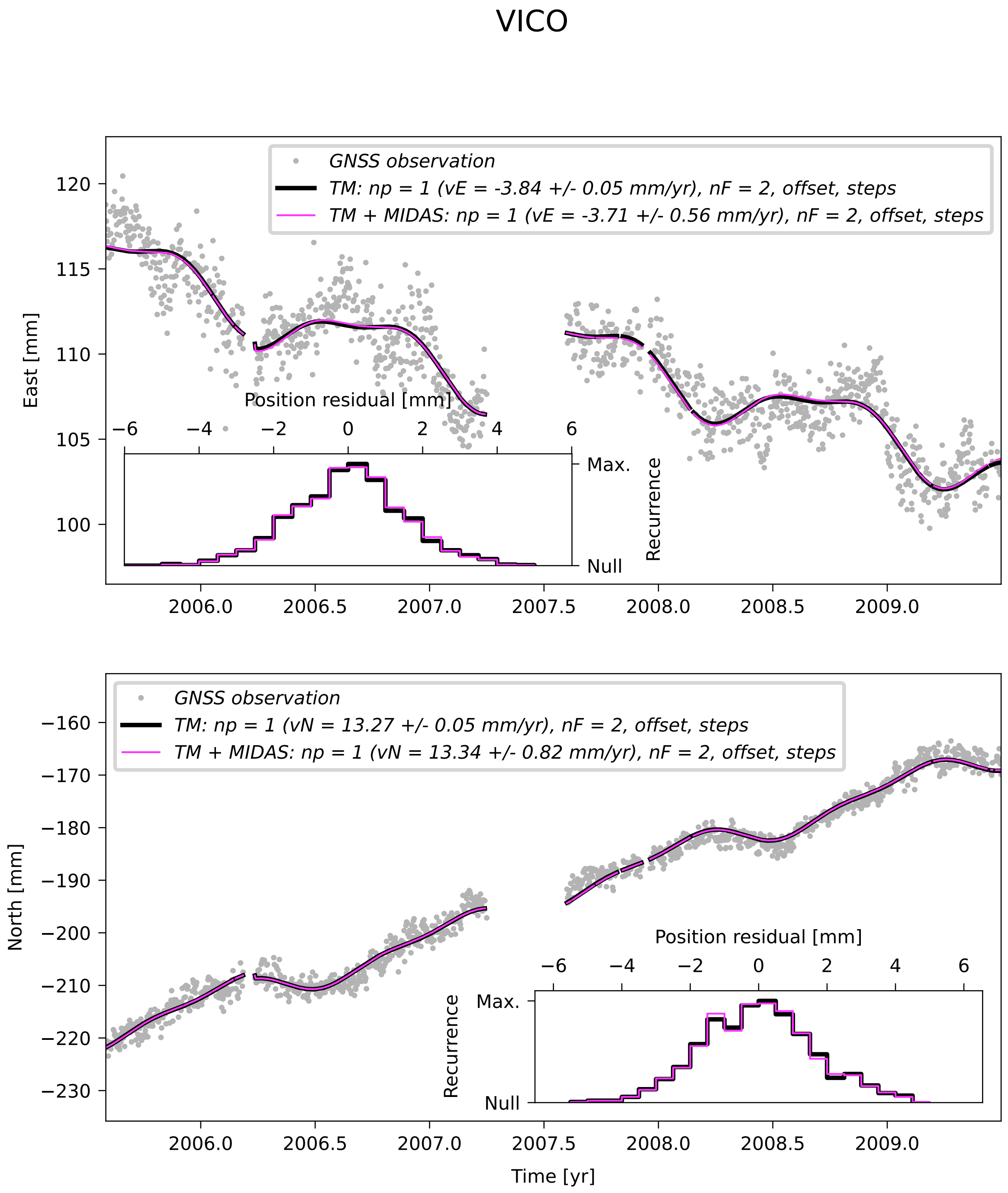

Supplementary Figure 98. Same as Supplementary Figure 18, but showing the time series and TMs of station VICO for the period from July 2005 to June 2009.

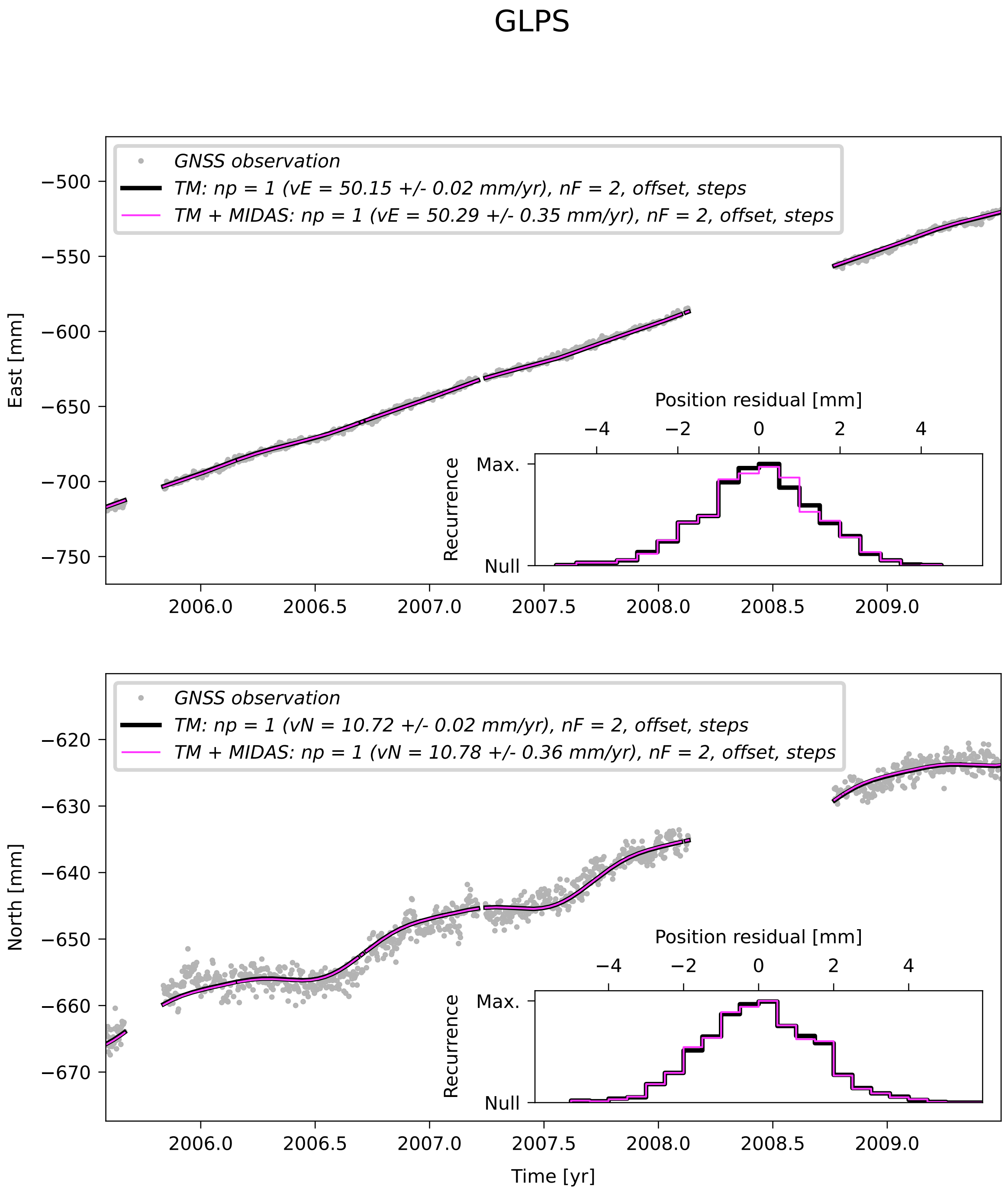

Supplementary Figure 99. Same as Supplementary Figure 18, but showing the time series and TMs of station GLPS for the period from July 2005 to June 2009.

ISPA

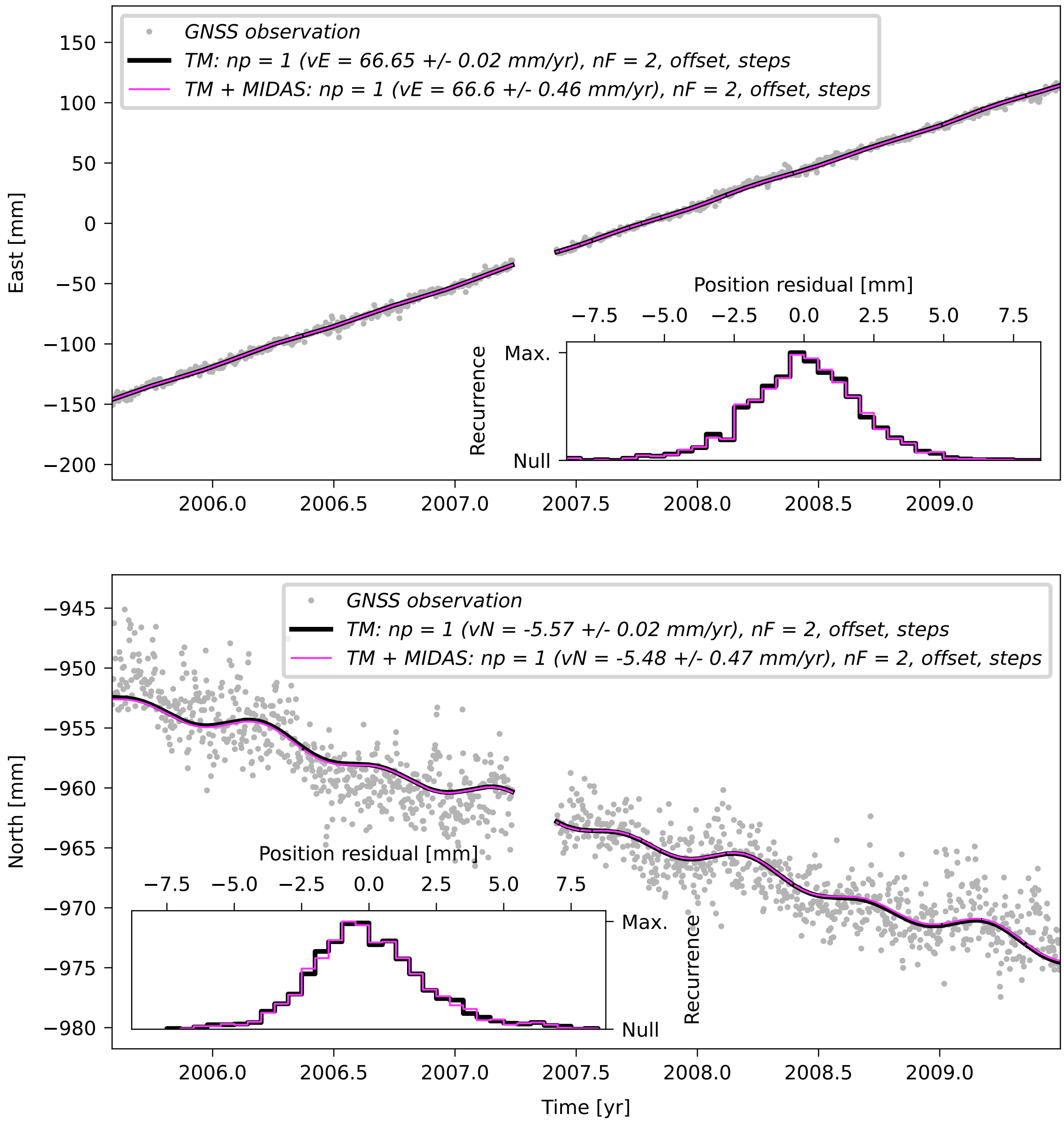

Supplementary Figure 100. Same as Supplementary Figure 18, but showing the time series and TMs of station ISPA for the period from July 2005 to June 2009.

References

1. Priestley, K. & McKenzie, D. The relationship between shear wave velocity, temperature, attenuation and viscosity in the shallow part of the mantle. *Earth Planet. Sci. Lett.* **381**, 78–91, DOI: 10.1016/j.epsl.2013.08.022 (2013).
2. Bevis, M. & Brown, A. Trajectory models and reference frames for crustal motion geodesy. *J. Geod.* **88**, 283–311, DOI: 10.1007/s00190-013-0685-5 (2014).
